# Candidate selective sweeps in US wheat populations

**DOI:** 10.1002/tpg2.20513

**Published:** 2024-09-25

**Authors:** Sajal R. Sthapit, Travis M. Ruff, Marcus A. Hooker, Bosen Zhang, Xianran Li, Deven R. See

**Affiliations:** ^1^ Department of Plant Pathology Washington State University Pullman Washington USA; ^2^ The Land Institute Salina Kansas USA; ^3^ USDA‐ARS Wheat Health, Genetics, and Quality Research Unit Washington State University Pullman Washington USA; ^4^ Department of Crop and Soil Sciences Washington State University Pullman Washington USA; ^5^ Department of Biological Sciences College of Southern Nevada Henderson Nevada USA; ^6^ Washington State University Pullman Washington USA

## Abstract

Exploration of novel alleles from ex situ collection is still limited in modern plant breeding as these alleles exist in genetic backgrounds of landraces that are not adapted to modern production environments. The practice of backcross breeding results in preservation of the adapted background of elite parents but leaves little room for novel alleles from landraces to be incorporated. Selection of adaptation‐associated linkage blocks instead of the entire adapted background may allow breeders to incorporate more of the landrace's genetic background and to observe and evaluate novel alleles. Important adaptation‐associated linkage blocks would have been selected over multiple cycles of breeding and hence are likely to exhibit signatures of positive selection or selective sweeps. We conducted genome‐wide scan for candidate selective sweeps (CSS) using F_st_, Rsb, and xpEHH in state, regional, spring, winter, and market‐class population pairs and reported 446 CSS in 19 population pairs over time and 1033 CSS in 44 population pairs across geography and class. Further validation of these CSS in specific breeding programs may lead to identification of sets of loci that can be selected to restore population‐specific adaptation in pre‐breeding germplasms.

## Core Ideas

- Selective sweeps were reported in 63 US wheat populations over time and across regions, states, and market classes.
- Exploration of candidate selective sweeps may aid breeders in maintaining adaptation while incorporating novel alleles.
- Newer varieties were most differentiated from older varieties within the Pacific and soft white spring populations.
- The Pacific, hard red spring, and South Carolina populations were most differentiated from other regions and classes.

**Abbreviations**
CNV: copy number variation
CSS: candidate selective sweeps
EHH: extended haplotype homozygosity
HRS: hard red spring
HRW: hard red winter
PIC: polymorphic information content
pnw: Pacific Northwest
SNP: single nucleotide polymorphism
SRW: soft red winter
SWS: soft white spring
SWW: soft white winter
xpEHH: cross‐population extended haplotype homozygosity

## 1 INTRODUCTION

Genetic bottlenecks, first during crop domestication and then with plant breeding, have affected all crop species (Louwaars, 2018; Tanksley & McCouch, 1997). Selection by early farmers for mutants, interspecific hybrids, and polyploids with domestication syndrome traits increased the progeny populations of a small subset of plants that became the crops we grow today. It is unlikely that domestication syndrome traits appeared in the plants that also had the most agronomically valuable alleles at other loci (Charmet, 2011). A wealth of favorable alleles may still be found in ex situ collections and landraces on farms that have yet to be explored in the genetic background of elite modern wheat varieties (Sehgal et al., 2015). Limited characterization and evaluation information on germplasm and the sheer time and effort it takes to incorporate useful genes from germplasm into adapted backgrounds can deter plant breeders from using these resources (Byrne et al., 2018). Breeding programs often rely on crossing elite by elite lines to maintain adaptedness (Baenziger & Depauw, 2009). A wide cross between a landrace and an elite line is typically followed by a series of backcrosses to the elite line to restore its genetic background. Backcross breeding is effective at preserving favorable linkage blocks from elite lines but is conservative in terms of broadening the genetic base (Sneep, 1979). Screening for selective sweeps or signatures of positive selection in specific populations can be a way to identify linkage blocks that are favorable for regional adaptation. If breeders apply forward selection only on specific linkage blocks associated with adaptation rather than the elite parent's entire genetic background, then even experimental lines with broader genetic bases may have adequate fitness to be included in variety testing and be evaluated for potentially useful novel alleles. By tracking adaptation‐associated linkage blocks during pre‐breeding, adapted germplasm with broader genetic base can be developed. Broadening the genetic base may be necessary to make continual gains in complex traits such as yield. In this study, we use three statistics to scan for candidate selective sweeps (CSS) in different market class, regional, and state wheat populations of the United States.

## 2 METHODS

### 2.1 Wheat variety panel

**FIGURE 1:**
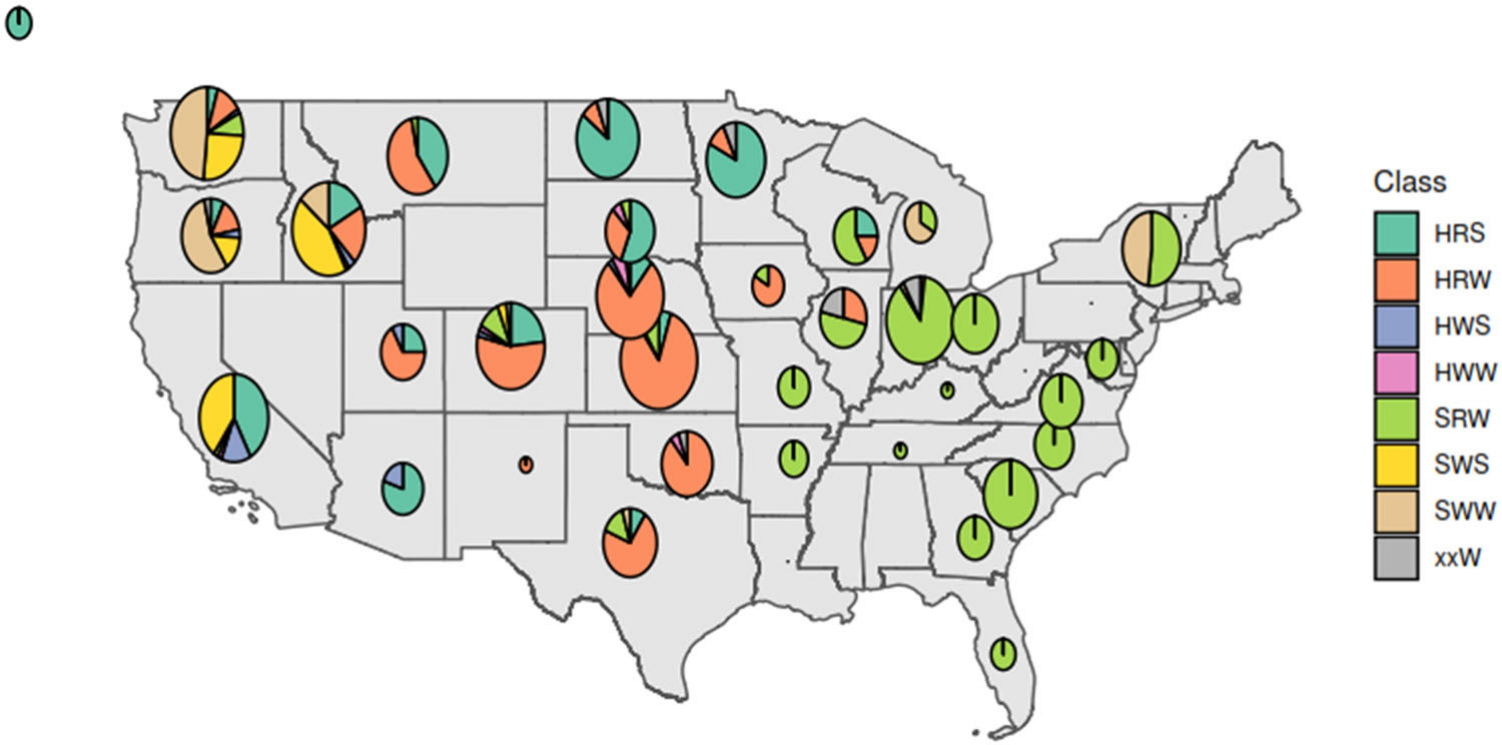
Distribution of 753 US wheat varieties used in the study by state. Four varieties from Alaska are shown on the top left outside the continental map. The pie chart diameter is proportional to the base 10 logarithm of the number of varieties. HRS, hard red spring; HRW, hard red winter; HWS, hard white spring; HWW, hard white winter; SRW, soft red winter; SWS, soft white spring; SWW, soft white winter; xxW, winter varieties of unknown market class.

The study panel included 753 US wheat varieties, 236 spring and 517 winter growth habits, from 1858 to 2014 (Figure 1; Table S1). We assigned varieties to five production regions. The Eastern region extends from the Atlantic coast to the states of Missouri and Arkansas and specializes in the soft red winter (SRW) market class. The Great Plains region extends from northern Texas to Nebraska and specializes in hard red winter (HRW). North of the Great Plains is the Northern region where hard red spring (HRS) is the main market class. To the west of the Northern region is the Pacific Northwest that has greater diversity of market classes but is known for soft white spring (SWS) and winter (SWW) wheat. The fifth region is the Pacific with California and Arizona that grow mostly HRS and SWS wheat. These regions roughly correspond to the four production regions defined by the National Association of Wheat Growers (n.d.) with a few differences. We separated California and Arizona from the greater Pacific Northwest into their own region called the Pacific because the production areas are separated by the southern half of Oregon and the Pacific varieties are mostly spring wheat. Varieties from Utah were added to the Pacific Northwest because of the proximity of Utah and Idaho wheat‐growing areas compared to any other wheat‐producing state.

### 2.2 Genotyping

The study uses the single nucleotide polymorphism (SNP) genotyping data generated from a previous study (Sthapit et al., 2022) that described the detailed methodology. In short, seeds from the US National Plant Germplasm System in Aberdeen, ID, were used to grow green leaf tissue for genomic DNA extraction using a large‐scale DNA extraction protocol as described in Faris et al. (2000). The Illumina Infinium iSelect 90K SNP array platform (Illumina Inc.) was used for genotyping at the USDA‐ARS Small Grains Genotyping Center in Fargo, ND. The default GenTrain algorithm in GenomeStudio ver. 2.0.3 (Illumina Inc.) was used to calculate call frequencies for all markers and samples. The 31,230 SNPs with call frequency ≥90% that were also previously mapped by Wang et al. (2014) were manually called in the diploid module of GenomeStudio. Only the SNPs that behaved like diploid markers (two or three distinct clusters) with small heterozygote clusters were used resulting in a total of 24,033 SNP markers with less than 10% missing calls (average 0.43% missing per marker). Missing values were imputed using LinkImpute (Money et al., 2015). Sequences for the 24,033 markers were downloaded from the Triticeae Toolbox at: https://triticeaetoolbox.org/wheat/genotyping/marker_selection.php. We queried the marker sequences against the respective chromosomes of the Chinese Spring reference sequence ver. 2.1 (IWGSC et al., 2018) to obtain their base pair positions. Of the 24,033 markers, 747 did not return a physical position on the chromosome queried, 560 were mapped at two or more locations, and 259 had different marker names but mapped to the same position. After removing these markers, we had 21,714 loci with unique base pair positions. By applying a filter of minor allele frequency ≥5%, we obtained a final dataset of 13,995 polymorphic SNP markers (5529, 7149, and 1317 on the A, B, and D genomes, respectively). The GenomeStudio genotype calls in AB format were converted to nucleotide (ACGT) format using the 90K SNP array wheat manifest file that employs conversion rules described at: https://malt.pw.usda.gov/t3/sandbox/wheat/snps.php.

### 2.3 Data analysis

Three methods, the Weir and Cockerham's fixation index (F_st_) (Weir & Cockerham, 1984), the ratio of site‐specific haplotype homozygosity (Rsb) (Tang et al., 2007), and cross‐population extended haplotype homozygosity (xpEHH) (Sabeti et al., 2007), were used to conduct genome‐wide scans for selective sweeps in various US wheat populations. Selection of an allele can leave different types of signatures of selection in the genome. Loci that are selected can be highly differentiated between populations, which can be detected by outlier values of F_st_. Selection can also result in the selected allele occurring in longer conserved haplotypes with high extended haplotype homozygosity (EHH) values (Sabeti et al., 2002), due to the hitch‐hiking effect of alleles closer to the favored allele being inherited together (Smith & Haigh, 1974). The EHH‐based statistics Rsb and xpEHH compare the EHH of different alleles in the two populations to determine if selection is occurring in one population compared to another. Extreme values for Rsb or xpEHH may indicate possible recent selection.

**Core Ideas**

- Selective sweeps were reported in 63 US wheat populations over time and across regions, states, and market classes.
- Exploration of candidate selective sweeps may aid breeders in maintaining adaptation while incorporating novel alleles.
- Newer varieties were most differentiated from older varieties within the Pacific and soft white spring populations.
- The Pacific, hard red spring, and South Carolina populations were most differentiated from other regions and classes.

Various functions in the R package “tidyverse” were used to organize the data for analysis and interpretation (R Core Team, 2018; Wickham et al., 2019). Polymorphic information content (PIC) or gene diversity was calculated with the formula, PIC = 1 − Σ*p_i_* ^2^, where *p_i_* is the frequency of the *i*th allele in the population (Weir, 1996). F_st_ was calculated using the “Fst” function in the R package “pegas” (Paradis, 2010), while Rsb and xpEHH were calculated using the functions “scan_hh,” “ines2rsb,” and “ies2xpehh” in the R package “rehh” (Gautier et al., 2017). As large gaps between consecutive markers can incorrectly inflate EHH values, we capped large gaps between consecutive markers down to 2500 kb with the scalegap parameter.

To identify selective sweeps due to breeding over time, we split a population into one half of older and one half of newer varieties and calculated F_st_, Rsb, and xpEHH using the population pair consisting of the older or reference and newer or target halves. For example, the HRS population of 150 varieties was split into HRS1 with 75 older varieties from 1895 to 1978 and HRS2 with 75 newer varieties from 1978 to 2009. Varieties released in the same year were sorted alphabetically by their accession numbers (Table S2). A population pair was not analyzed if one population in the pair had less than 20 varieties. We had one pair for the halves of the entire panel, one pair for all spring varieties, one pair for all winter varieties, five pairs for regional varieties with both spring and winter habit, three pairs for regional spring varieties, three pairs for regional winter varieties, and five pairs by market classes for a total of 19 population pairs for over time comparisons.

To identify selective sweeps due to regional or geographic adaptation, we defined reference population as varieties from other regions. For example, to estimate selective sweeps in the Pacific spring wheat, the reference population included spring varieties from regions other than the Pacific. To identify selective sweeps in state populations, the reference population had varieties from other states with the exclusion of states from the same region as the target state or the states that share a border with the target state. For example, for California spring wheat population, the reference population included spring varieties from the states in the Eastern, the Northern, the Great Plains, and the Pacific Northwest regions except for Oregon because it borders California. A population pair was not analyzed if one population in the pair had less than 20 varieties. We had five pairs of regional populations with both spring and winter habit, four pairs of regional spring populations, and four pairs of regional winter populations for a subtotal of 13 regional‐level population pairs. Likewise, there were 12 pairs of state (vs. others) populations with both spring and winter habit, five pairs of state spring populations, and nine pairs of state winter populations for a subtotal of 26 state‐level population pairs. Finally, there were five pairs of market‐class population pairs, for a total of 44 pairs for across‐region comparisons.

The EHH‐based statistics, Rsb and xpEHH, have a normal distribution with mean of 0 and standard deviation of 1 under the null hypothesis of neutrality. Therefore, above the absolute value of 2 are the extreme 5% of the observations. The null distribution was compared with the empirical distribution of Rsb and xpEHH using the “rehh” package function “distribplot” (Gautier et al., 2017). For all 63 population pairs scanned, markers with Rsb and xpEHH values between −2 and +2 were close to normally distributed (Figures S1–S2), suggesting that the markers with extreme values can be plausible candidates under selection. Cluster of markers with extreme values are more indicative of selection than an isolated marker with a very extreme value (Gautier et al., 2017; Voight et al., 2006). CSS were identified for each statistic using the “rehh” package function “calc_candidate_regions” with a window size of 5000 kb, a minimum of five markers in the window, and at least 90% of markers in the window exceeding the threshold of +2. A positive value of Rsb or xpEHH indicates that the average length of shared haplotypes is longer, a sign of selection, in the target population (designated by scan_pop1 in the “ines2rsb” and the “ies2xpehh” functions), while a negative value indicates they are longer in the reference population (Klassmann et al., 2021). Consecutive windows that are less than 1000 kb apart were merged into one CSS. CSS based on F_st_ values were also calculated using the “calc_candidate_regions” function from the “rehh” package using the same settings as Rsb and xpEHH, except for the threshold value. The 95th percentile F_st_ value was calculated for each population pair and used as the threshold for identifying CSS.

We obtained the physical positions of 45 known informative markers for various adaptation and quality traits in wheat by searching the published probe sequences (Table S3) against the respective chromosomes of the Chinese Spring wheat reference genome ver. 2.1 using the basic local alignment search tool (BLAST) implemented at the Wheat@URGI portal (Alaux et al., 2018). The candidate regions with selective sweeps identified by F_st_, Rsb, or xpEHH and the known informative markers were visualized in a selection map using the software MapChart ver. 2.32 (Voorrips, 2002). Details about all the CSS were saved in an html file using R Markdown. Every CSS was given a serial number (css1 to css1479) that can be used to locate information about it in the selection map, html R Markdown output, and the CSV output file for more details. An online database that breeders and researchers can browse for their specific population of interest has also been made publicly available at www.wheatselectivesweeps.org. The data, R Markdown files, and outputs from the analysis are available at https://doi.org/10.5061/dryad.ghx3ffbx0.

## 3 RESULTS

**FIGURE 2:**
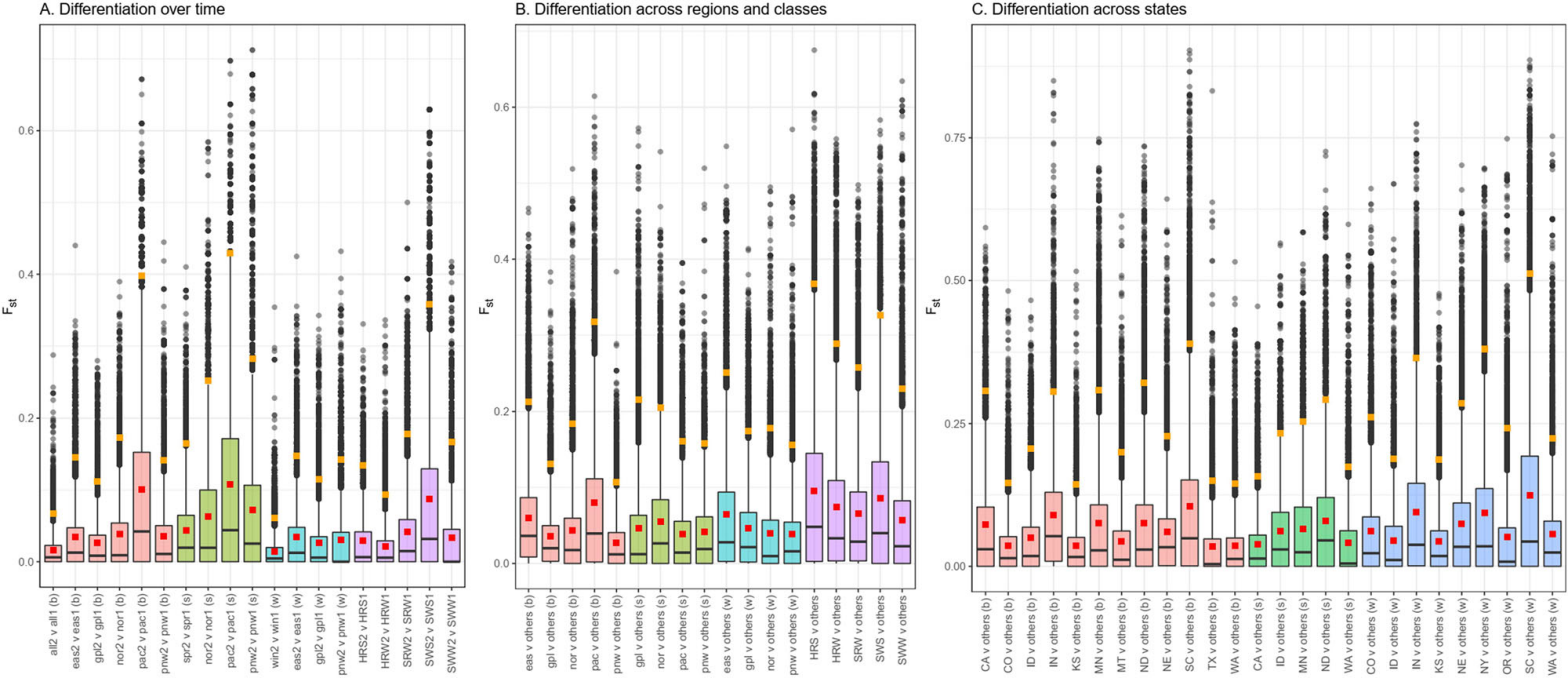
F_st_ differentiation between wheat population pairs in the United States. The red squares indicate mean F_st_ values. The orange squares are the 95th percentile F_st_ values, which were used as the population pair‐specific threshold for identifying candidate selective sweeps. The whiskers atop the boxplots represent 1.5 times the interquartile range, and the black dots are the outlying F_st_ values that exceed 1.5 times the interquartile range. The letters in the parenthesis (b, s, and w) refer to populations with both spring and winter varieties, populations with only spring varieties, and populations with only winter varieties, respectively. (A) Differentiation over time was computed by dividing a population into two halves of newer (suffix 2) and older (suffix 1) varieties and using the pair to calculate F_st_. (B) Differentiation of regional wheat populations compared to rest of the US population, that is, others. (C) Differentiation of state wheat populations compared to varieties from other regions, excluding neighbor states that share a border. The population legends are: all, entire study panel; eas, the Eastern; gpl, the Great Plains; nor, the Northern; pac, the Pacific; pnw, the Pacific Northwest; HRS, hard red spring; HRW, hard red winter; SRW, soft red winter; SWS, soft white spring; SWW, soft white winter.

Selective sweeps were scanned in 63 population pairs using F_st_, Rsb, and xpEHH. The median and 95th percentile of F_st_ values for the 63 population pairs ranged from 0 to 0.05 and 0.06 to 0.51, respectively. The distribution of F_st_ values indicated that the Pacific spring population had differentiated the most from the older varieties in these respective populations (Figure 2A). The differentiation over time within spring populations was more pronounced than within winter populations (Figure 2A). Across regions too, the Pacific population was most differentiated from the rest (spring and winter combined) followed by the Eastern population (Figure 2B). However, the Northern and the Great Plains spring populations were more differentiated than the rest of the spring varieties, while the Eastern winter population was more differentiated than the rest of the winter varieties (Figure 2B). Among state populations, South Carolina, Indiana, and California were most differentiated from the rest (Figure 2C). The ND spring population was most differentiated from the rest of the spring varieties, while the South Carolina, New York, and Indiana winter population was most differentiated from the rest of the winter varieties (Figure 2C). Over time periods, the SWS market class was most differentiated from older SWS varieties (Figure 2A). Across market classes, HRS and SWS were most differentiated from others (Figure 2B).

The outlier approach to F_st_ (setting the 95th percentile F_st_ value as the threshold) reported 555 CSS, while Rsb and xpEHH reported 400 and 524 CSS, respectively, for a total of 1479 CSS (Table 1; Table S4; Supporting Information S1). The 1479 CSS are not all unique as CSS from different populations pairs and different statistics often overlap. For example, the chromosome segment 1A:23–54 Mbp has 31 CSS reported by either F_st_, Rsb, or xpEHH in 18 population pairs. The median correlation between F_st_ and |Rsb|, Fst and |xpEHH|, and Rsb and xpEHH were 0.10 (range of −0.02–0.33), 0.20 (0.02–0.38), and 0.81 (0.74–0.89), respectively. The largest CSS were 39, 69, and 71 Mbp, respectively, for F_st_, Rsb, and xpEHH.

**TABLE 1:** Number of candidate selective sweeps (CSS) detected on different chromosomes.

Chromosome	1A	1B	1D	2A	2B	2D	3A	3B	3D	4A	4B	4D	5A	5B	5D	6A	6B	6D	7A	7B	7D	All
**All CSS (in 63 population pairs)**																						
Fst	40	22	34	29	49	27	25	36	8	13	34	1	46	27	5	74	32	0	16	32	5	555
Rsb	45	12	3	42	21	1	7	59	0	2	1	0	19	25	0	16	122	0	8	17	0	400
xpEHH	68	5	3	55	33	2	14	48	0	12	3	0	33	59	0	2	152	0	15	20	0	524
Total	153	39	40	126	103	30	46	143	8	27	38	1	98	111	5	92	306	0	39	69	5	1479
**CSS in a population over time (in 19 population pairs)**																						
Fst	6	10	18	5	17	15	1	5	1	2	5	0	11	3	0	43	22	0	4	14	0	182
Rsb	5	1	3	3	12	1	0	0	0	1	0	0	5	0	0	6	71	0	0	3	0	111
xpEHH	8	0	3	6	21	2	0	0	0	4	3	0	11	6	0	1	82	0	0	6	0	153
Total	19	11	24	14	50	18	1	5	1	7	8	0	27	9	0	50	175	0	4	23	0	446
**CSS across populations (in 44 population pairs)**																						
Fst	34	12	16	24	32	12	24	31	7	11	29	1	35	24	5	31	10	0	12	18	5	373
Rsb	40	11	0	39	9	0	7	59	0	1	1	0	14	25	0	10	51	0	8	14	0	289
xpEHH	60	5	0	49	12	0	14	48	0	8	0	0	22	53	0	1	70	0	15	14	0	371
Total	134	28	16	112	53	12	45	138	7	20	30	1	71	102	5	42	131	0	35	46	5	1033
**CSS across state‐level populations (in 26 population pairs)**																						
Fst	16	7	6	17	19	9	17	12	4	5	13	1	18	15	2	22	7	0	8	10	4	212
Rsb	28	7	0	23	2	0	5	37	0	0	0	0	9	14	0	6	36	0	7	7	0	181
xpEHH	38	5	0	31	4	0	12	31	0	5	0	0	11	26	0	1	54	0	11	11	0	240
Total	82	19	6	71	25	9	34	80	4	10	13	1	38	55	2	29	97	0	26	28	4	633
**CSS across regional‐level populations (in 13 population pairs)**																						
Fst	6	5	8	4	8	3	4	14	3	5	11	0	11	8	2	9	1	0	1	7	1	111
Rsb	7	4	0	10	7	0	2	15	0	1	1	0	4	8	0	3	13	0	1	5	0	81
xpEHH	14	0	0	12	7	0	1	12	0	2	0	0	7	21	0	0	15	0	4	3	0	98
Total	27	9	8	26	22	3	7	41	3	8	12	0	22	37	2	12	29	0	6	15	1	290
**CSS across market classes (in five population pairs)**																						
Fst	12	0	2	3	5	0	3	5	0	1	5	0	6	1	1	0	2	0	3	1	0	50
Rsb	5	0	0	6	0	0	0	7	0	0	0	0	1	3	0	1	2	0	0	2	0	27
xpEHH	8	0	0	6	1	0	1	5	0	1	0	0	4	6	0	0	1	0	0	0	0	33
Total	25	0	2	15	6	0	4	17	0	2	5	0	11	10	1	1	5	0	3	3	0	110

**FIGURE 3:**
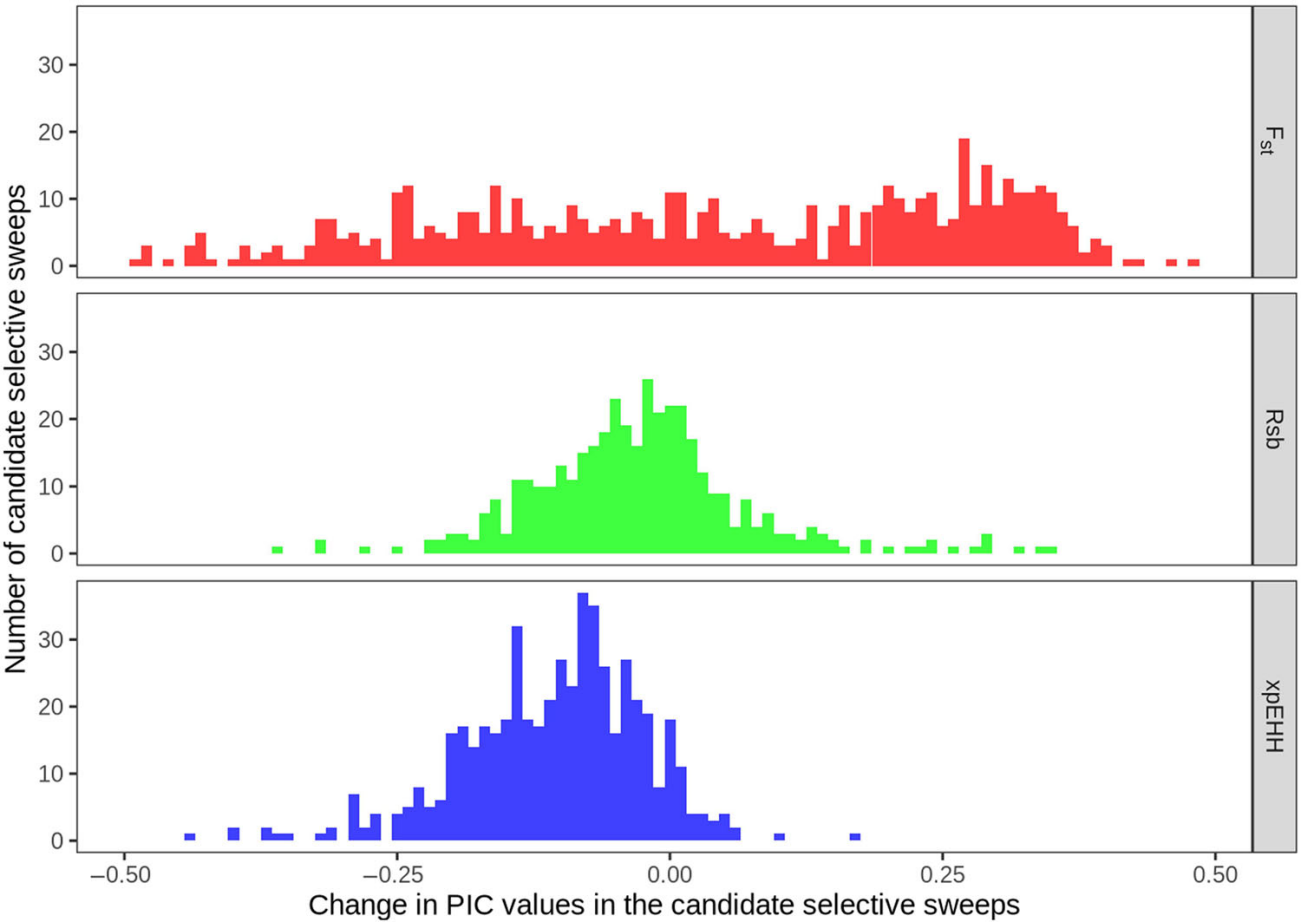
Change in polymorphic information content (PIC) values (∆PIC) of single nucleotide polymorphisms (SNPs) in the candidate selective sweeps detected by F_st_, Rsb, and xpEHH.

In 980 CSS, genetic diversity (measured as PIC) decreased in the target population and genetic diversity increased in the remaining 499. However, the change in genetic diversity of CSS was different based on the statistic used to detect the CSS. The EHH‐based statistics, Rsb and xpEHH, reported more CSS with loss of genetic diversity (Figure 3). Forty‐two percent of the F_st_ CSS had lower genetic diversity, while 65% and 92% of the Rsb and xpEHH CSS had lower genetic diversity. CSS detected by F_st_ had a ∆PIC range of −0.49–0.48. The Rsb and xpEHH had smaller spread of ∆PIC (−0.36–0.35 and −0.44–0.17, respectively). The mean ∆PIC for all 1479 CSS was −0.028. For F_st_, Rsb, and xpEHH CSS, the mean ∆PIC were 0.047, −0.028, and −0.106, respectively.

The genomes A, B, and D had 581, 809, and 89 CSS, respectively. The EHH‐based statistics, Rsb and xpEHH, reported only nine CSS on the D genome chromosomes, with the rest detected by F_st_. The number of CSS detected in a chromosome was positively correlated with the number of loci for all three statistics (F_st_: *r*(19) = 0.61, *p* = 0.002; Rsb: *r*(19) = 0.54, *p* = 0.006; xpEHH: *r*(19) = 0.59, *p* = 0.003). The CSS detected by EHH‐based statistics also tended to be located closer to the ends of the chromosomes, while the F_st_ outlier CSS were also found around the centromeric region of the chromosome (Figures S3–S4). Chromosome 6B had the most CSS (306) followed by 1A (153) and 3B (143), while chromosome 4D had only one CSS across all 63 population pairs compared.

### 3.1 Selective sweeps over time

To assess CSS due to selection over time, we split a population into halves of newer and older varieties and computed F_st_, Rsb, and xpEHH using the pairs of new (target population for selection) and old (reference population) halves. In total, 19 population pairs were scanned, and a total of 446 CSS (182, 111, and 153 reported by F_st_, Rsb, and xpEHH, respectively) were found (Table 1; Figure S3). CSS were not found in chromosomes 4D, 5D, 6D, and 7D. Chromosome 6B had the most CSS (175) but only 25 were in population pairs of only winter. The Northern population had the most CSS (21) over time on chromosome 2B, while the other four regions all had the most CSS on 6B.

The short arms of chromosomes 1A and 1B had only five CSS despite being the site for wheat–rye translocations. The chromosome region 1A:474–519 Mbp, which also encompasses the Glu‐B1 locus, had 12 CSS, mostly in the Eastern and SRW populations. Chromosome 1D had 24 CSS, with 17 of them in the 1D:26–58 Mbp segment indicating selection in all five regions. However, in the Pacific population (css202, css203, and css209) selection over time resulted in reduced diversity and an increase in major allele frequency, while in the Northern (css201) and the Eastern (css208) populations, there was an increase in diversity. The remaining five selective sweeps on 1D were found in the 406–422 Mbp segment that also encompasses the Glu‐D1 locus.

Chromosome 2A had 14 CSS with nine of them clustered in the 2A:691–718 Mbp segment representing selection in the Great Plains and HRW populations. This cluster also encompassed the polyphenol oxidase locus. Chromosome 2B had 50 CSS with 48 of them clustered within two large clusters. One with 16 CSS, all reported by F_st_ and mostly selected in the Northern population, were within the 2B:388–458 Mbp region. The second cluster had 32 CSS within the 2B:597–762 Mbp, which also includes the Yr5 stripe rust resistance gene. Most of the CSS in this cluster were found in the HRS and the Northern population. The chromosome region 2D:59–85 Mbp had 13 CSS, which were about 22 Mbp proximal from the known photoperiod genes. Two dense CSS with over 70 SNPs were found in the Pacific population (css483 and css484) with an increase in diversity over time in the region 2D:569–583 Mbp that also encompasses the polyphenol oxidase gene.

No CSS were detected over time near the reduced height genes Rht‐B1 and Rht‐D1 on chromosomes 4B and 4D, respectively.

Most of the CSS on 5A were in a cluster within the 5A:434–510 Mbp segment, representing selection over time mostly in the Pacific Northwest winter wheat populations. However, CSS in the Great Plains and SWW populations over time were flanking the vernalization gene Vrn‐A1, while CSS in HRW were closest to the Vrn‐B1 gene on 5B.

Chromosome 6A had 50 CSS and 43 of them were reported by F_st_. Two clusters of CSS, 6A:103–112 Mbp and 6A:409–446 Mbp, exhibit large increases in genetic diversity over time (especially in the Eastern and the Pacific Northwest populations) going from minor allele frequencies range of 0–0.12 to 0.22–0.50. Chromosome 6B had the most CSS at 175. Only 22 of these CSS were reported by F_st_ and only 25 were in strictly winter population pairs, indicating major genetic changes mostly in the spring population over time. Among the spring wheat populations, 29 CSS were found in the Pacific Northwest (PNW) population, 14 in the Pacific population, and 12 in SWS.

### 3.2 Selective sweeps across populations

Scanning of selective sweeps by comparing populations from two different regions can identify linkage blocks in the genome that may be key to regional adaptation of wheat varieties. To identify these linkage blocks, we conducted scans for selective sweeps in 13 pairs of regional populations, 26 pairs of state populations, and five pairs of market class populations and found 1033 CSS with 373, 289, and 371 reported by F_st_, Rsb, and xpEHH, respectively (Table 1; Table S4; Figure S4). No CSS were found on chromosome 6D. Chromosomes 3B, 1A, 6B, 2A, and 5B had the most CSS for across population comparisons. Only F_st_ detected CSS on the D genome when comparing across populations.

The Eastern population had the least number of CSS (24) with nine on 5B and five on 5A. The Great Plains population had 70 CSS with 19 on 2B and 13 on 1A. The Northern population had the most CSS (77) with 12 each on 5A and 5B. The Pacific population had 46 CSS with 12 on 6B and nine on 2A. And the Pacific Northwest population had 73 CSS with 26 on 3B and eight on 5B. The SRW, a market class grown mostly in the Eastern region, also had the least number of CSS at 14 when compared to other classes. The HRS class had 24 CSS with 13 on 1A, HRW had 21 CSS with eight on 2A, SWS had 20 CSS, and SWW had 31 CSS with 13 on 3B.

Chromosome 1A had 134 CSS, with the Rsb and xpEHH reported CSS clustered toward the ends of the chromosomes within 1A:23–54 Mbp and 1A:462–582 Mbp. Between these two clusters were CSS reported by F_st_. Chromosome 1B had 28 CSS. California and the Pacific spring population had CSS encompassing or nearby the Glu_B1 gene, while North Dakota populations had CSS clustered in the 1B:626–675 Mbp region. Chromosome 1D had 16 CSS, all reported by F_st_. Ten CSS were either clustered nearby or encompassed (css221 to css225 in the Northern, PNW, and SWS populations) the Glu_D1 gene.

Chromosome 2A had 112 CSS, with 108 of them in two clusters toward the ends of the chromosome in the regions 2A:48–123 Mbp and 2A:568–725 Mbp. No CSS were detected in spring populations on the short arm of 2A, which also encompasses the photoperiod genes. Chromosome 2B had 53 CSS with 19 in the Great Plains spring population, mostly on the long arm.

Chromosome 3B had 138 CSS, with 119 reported in population pairs with winter varieties. The chromosome seems especially important for wheat populations from the Pacific Northwest as 92 CSS were shared among PNW, SWW, Idaho, Oregon, and Washington populations. Notably, 3B had only five CSS when selection over time was compared within the same population.

Just as in scan over time, no CSS were found near the Rht‐B1 gene on 4B, but one CSS in NY winter wheat was detected near the Rht‐D1 gene on 4D.

Chromosome 5A had 71 CSS, nine on the short arm within 5A:22–48 Mbp and the rest on the long arm. North Dakota and the Northern regional populations had 27 CSS on 5A. There were two clusters on the long arm, one at 5A:434–493 Mbp with 35 CSS reported in North Dakota and Northern spring populations and mostly in HRW and SRW populations. The next cluster of 23 CSS was within the 5A:568–624 Mbp segment that also encompasses the VRN_A1a gene, with only two CSS reported in winter population pairs.

Chromosome 6A had 42 CSS, and 31 of them were reported by F_st_. California, Minnesota, and North Dakota had multiple CSS on the long arm within 6A:524–599 Mbp. Chromosome 6B had 131 CSS, the second most after chromosome 3B in the across population comparisons. Of the 131, 97 were from state‐level populations, 29 were in regional populations, and only five were in market class populations. Indiana had the most CSS (19) on 6B followed by Idaho (14), South Carolina (12), and Minnesota (11).

Chromosome 7A had 35 CSS with 26 of them reported in state populations and only one in spring only population. Kansas had 14 CSS and Montana had four. Chromosome 7B had 46 CSS with 10 coming from Texas, but only two (css1442 and css1443) of them overlapped with a CSS detected in the Great Plains population that also contains Texas wheat. Chromosome 7D had five CSS, all reported by F_st_. One of them in the Pacific Northwest population (css1475 at 7D:5–10 Mbp) was in a segment that also encompassed a 1000‐grain weight gene.

## 4 DISCUSSION

CSS have previously been reported in global (Cavanagh et al., 2013), Chinese (Zhou et al., 2017), Australian (Joukhadar et al., 2019), and Canadian spring (Semagn et al., 2021) wheat populations. Two recent studies in the United States have scanned for selection in the hard winter varieties (Ayalew et al., 2020) and the SRW (Gaire et al., 2020) populations. Our study population represents all wheat‐growing regions of the continental United States. We conducted scans for selective sweeps using F_st_, Rsb, and xpEHH on 63 pairs of wheat populations, including 19 pairs to assess selection over time within regional and market class populations, and 44 pairs to assess selection across regional, state, and market class populations.

Clustering of CSS from different populations may be indicative of the importance of different allelic variants in specific populations, while clustering of CSS from the same or related populations may indicate the relative importance of the genomic region for those populations. Further exploration of these CSS may lead to the identification of linkage blocks that confer adaptedness to varieties within specific populations. For instance, a plant breeder from Kansas may find CSS detected in Kansas, Great Plains, and HRW populations to be of particular interest.

Selection maps were prepared for the 446 CSS for selection over time within populations (Figure S3) and 1033 CSS for selection across populations (Figure S4). These selection maps provide the physical location of CSS in different populations. The labels provide summary information including the population name, statistics for selective sweep scans, PIC in target and reference populations, major allele frequencies in target and reference populations, and the CSS serial number. The same information is also available in a comma separated value (/output/cr_pic_freq_consolidated.csv at https://doi.org/10.5061/dryad.ghx3ffbx0) file, which can be filtered for the populations or chromosomes of interest (Table S1). Additional details for every CSS, including the SNP names, chromosome, base pair positions, PIC, allele frequencies, F_st_, Rsb, and xpEHH values for all the SNPs in the sweep, are provided in an html document (05_view_details_of_candidate_regions.html) in the dryad repository for this article (https://doi.org/10.5061/dryad.ghx3ffbx0). The CSS serial number (e.g., css1) can be used to search and locate further details in the html file or Table S1. An interactive web database with visualizations for the same information is available at https://feline.pw.usda.gov/wss/. Researchers can use the selection maps and the online database to browse for detailed information on CSS that may be useful in pre‐breeding to create diverse but adapted germplasm.

CSS were found to occur in clusters. Previously, Gao et al. (2017) reported candidate selection SNPs from the 90K wheat SNP chip to occur in clusters unevenly across the genome when they compared wild accessions with landraces and landraces with modern varieties. They reported more clusters near the centromeric regions, while we found CSS reported by EHH‐based statistics (Rsb and xpEHH) to be common toward the telomeric ends of the chromosomes. However, CSS reported by F_st_ tended to be closer to the centromeric regions. The difference in clustering patterns may be due to the nature of the different statistics used. F_st_ outliers and the other statistics used by Gao et al. (2017) depend on the allele frequency of the individual locus. On the other hand, the EHH‐based statistics are calculated based on the length of haplotype homozygosity that extends in both directions from a locus, and hence the marker density around the locus matters. The loci used in our study were not evenly distributed along the length of the chromosome with a low density of markers around the centromeres (Figure S5). Due to greater marker density further away from the centromere, the EHH‐based statistics detect more sweeps toward the ends of the chromosomes. While lower recombination rates around the centromeres would be expected to contribute to longer conserved haplotypes, it did not appear to compensate for lower density of markers for detection of CSS using Rsb and xpEHH.

F_st_ and the EHH‐based statistics (Rsb and xpEHH) were poorly correlated. The EHH‐based statistics use a log of ratio of estimated average length of shared haplotypes in the two populations. This value is then standardized with the median value for Rsb and the mean value for xpEHH. By contrast, F_st_ is calculated based on allele frequencies at a given marker and is not affected by the level of homozygosity around the locus itself. The use of marker‐level and haplotype‐level information may contribute to the lack of correlation between the two types of statistics. The varying strengths and weaknesses of EHH‐based and F_st_ underline the importance of using complementary statistics to scan for selective sweeps.

We found clusters of CSS close to some known adaptation or quality genes but not others. On chromosomes 1A and 1B, clusters of CSS were found around the Glu‐A1 and Glu‐B1 loci. High molecular weight glutenin subunits—a group of seed storage proteins encoded by the Glu‐A1, Glu‐B1, and Glu‐D1 genes—are responsible for dough strength and therefore bread and sponge cake quality (Chen et al., 2019; Payne, 1987). Allelic diversity at these loci results in various combinations of high molecular weight glutenin subunits (Dai et al., 2020; Ravel et al., 2020) that give different degrees of viscoelastic dough properties suitable for multiple end uses (Shewry et al., 2002). It is notable that the 1BL.1RS wheat–rye translocation did not bear any CSS when populations were compared over time. While 60% of CIMMYT germplasm in the 1990s had 1BL.1RS, only 15% of US commercial varieties have this genotype (Crespo‐Herrera et al., 2017). This relative rarity of 1BL.1RS in the US population may have been the reason for lack of CSS around here.

Multiple CSS were on the long arm of 2A encompassing the polyphenol oxidase gene Ppo‐A1. Polyphenol oxidase enzyme is implicated in the browning of wheat products, especially undesirable in Asian‐style noodles (He et al., 2007; Morris, 2018) and has been reported to be under selection. For instance, in a study of 320 historical and modern PNW varieties, variability at the Ppo‐A1 locus among the 23 pre‐1930 winter varieties had been nearly eliminated in the 56 new varieties from 2000 to 2015 (Sthapit et al., 2020). In contrast, no signals of selection around the Ppo‐A1 locus were found in the PNW2 winter populations in the current study. For the analysis in this study, we split the population into older and newer halves without any gap in terms of years between the two populations compared for Rsb and xpEHH. The temporal continuity of the populations compared may have limited detection of selective sweeps that had already begun in the older population. The Great Plains states specialize in HRW, which is a market class important for its versatility in uses that include pan breads, hard rolls, croissants, flat breads, Asian noodles, and blending with other flours (U.S. Wheat Associates, 2020). Reduction of polyphenol oxidase activity would be important to improve the versatility of HRW, which may explain the selection around Ppo‐A1 locus in HRW2 and the Great Plains populations.

The short arms of group 2 chromosomes have the photoperiod sensitivity loci Ppd‐A1, Ppd‐B1, and Ppd‐D1 (Snape et al., 2001). We found CSS proximal of the Ppd‐A1 locus on 2A on multiple state populations, with the closest one 7.5 Mbp away. However, there may be additional copies of the photoperiod loci on group 2 chromosomes. At least four copies of the photoperiod gene have been reported on 2B of Chinese Spring, and three copies each in Sonora 64 and Timstein (Díaz et al., 2012).

Fusarium head blight or scab is a major disease of wheat worldwide, and a major gene (Fhb1) for resistance has been found on the short arm of 3B (Bai & Shaner, 2004). However, we did not find any CSS closer than 31 Mbp to the Fhb1 gene. (Figures S3–S4). After the mapping of the Fhb1 gene in 1999 (Bai et al., 1999; Waldron et al., 1999), marker‐assisted selection was used to develop modern scab‐resistant varieties (Steiner et al., 2017). About 50% of the spring wheat varieties from the University of Minnesota breeding program have the Fhb1 gene, and over 20 HRS varieties with the gene are now widely grown in Canada and the Northern United States. (Buerstmayr et al., 2020). Our variety panel only has one HRS released after 1999 and no Minnesota variety after 1996, which may explain the lack of CSS near Fhb1.

The group 4 chromosomes did not have many CSS close to the dwarfing genes (Rht‐A1, Rht‐B1, and Rht‐D1) responsible for the Green Revolution (Hedden, 2003; Peng et al., 1999). The dwarfing genes from Japanese variety Norin 10 were incorporated into breeding materials by O.A. Vogel in Washington, which were then used as the source of dwarfing genes for the Green Revolution out of CIMMYT by Norman Borlaug (Borojevic & Borojevic, 2005). These semi‐dwarf varieties rapidly replaced traditional varieties grown in developing countries in the 1960–1970s (Dalrymple, 1980). Guedira et al. (2010) genotyped 362 varieties with diagnostic markers to determine the frequency of these genes over time in the Eastern and Central United States. In hard winter wheat, they found the Rht‐B1b genotypes in 75% of the varieties in 1980–1999 and 86% in 2000–2008. In the soft winter wheat, the Rht‐D1b genotypes were in 57% of 1980–1990 varieties and 58% in 2000–2008 varieties. Our study panel includes 116 of these varieties: 53 with wildtype alleles at both loci, 42 with Rht‐B1b (on 4B), and 21 with Rht‐D1b (on 4D). As we were not able to detect many CSS near the Rht gene in the 63 population pairs of our study, we scanned for CSS in the known Rht‐B1b versus wildtype population and the Rht‐D1b versus wildtype population. In the known Rht‐B1b population, we detected two CSS at 4B:11–29 Mbp and 4B:36–44 Mbp that flank the Rht‐B1 locus. The 7 Mbp gap between these CSS had only three SNPs, which also exceeded the +2 threshold. We did not detect any CSS near the Rht‐D1 gene on 4D even with the population of known genotype due to low density of SNPs around the Rht‐D1 locus on 4D. The ability to detect CSS around Rht‐B1 in populations with known genotypes but not in other population pairs suggests that there are sufficient Rht wildtype alleles in our target population.

Vernalization (Vrn‐1) genes—responsible for vegetative to reproductive transition in wheat and therefore spring, facultative, and winter growth habits (Distelfeld et al., 20092009)—are located on the long arms of chromosomes 5A, 5B, and 5D (Yan et al., 2003). Several signals were reported on the long arms of both 5A and 5B. Copy number variation (CNV) may be a mechanism for rapid adaptation under selection, especially in domesticated plants and animals (Lye & Purugganam, 2019). In wheat, CNV has been reported for vernalization and photoperiod sensitivity genes that may explain the global adaptation and fine‐tuning of the crop through altered levels of gene expression (Würschum et al., 2015). It is possible that clusters of CSS in our study may overlap with gene duplications of important adaptation genes.

An interesting finding that needs further exploration is the high number of CSS observed on chromosome 6B, especially for selection within populations over time. Ninety out of 175 CSS in spring populations over time came from only three related populations: newer Pacific varieties (pac2), newer Pacific Northwest varieties (pnw2), and newer SWS varieties (SWS2). There has been an increased use and sharing of CIMMYT germplasm in the Pacific and the Pacific Northwest spring wheat breeding programs in the second half of the 20th century (Balfourier et al., 2019), which has been detected as a marked shift in the spring wheat population structure between the pre‐1960 and post‐1960 spring wheat varieties (Sthapit et al., 2022). One possible explanation for the concentration of CSS in the Pacific and Pacific Northwest spring varieties on 6B could be due to the incorporation of novel germplasm.

Of the 89 CSS on the D genome, 80 were reported by F_st_. Large gaps between consecutive markers make EHH calculation unreliable (Gautier et al., 2017) and therefore denser genotyping or different statistics will be required to detect more sweeps on the D genome. In addition, EHH values cannot be calculated when the allele frequency in one or both populations being compared has reached 1. Therefore, Rsb and xpEHH are appropriate for detecting ongoing selective sweeps rather than completed sweeps (Sabeti et al., 2007; Tang et al., 2007). However, F_st_ can be calculated at all allele frequencies and hence suitable for detecting completed selective sweeps or sweeps starting from a point of low or no diversity. In the current study, F_st_ detected CSS that had undergone both an increase and a decrease in genetic diversity, while Rsb and xpEHH were more likely to report CSS that had reduced in genetic diversity (Figure 3). Furthermore, there was lower correlation between F_st_ values with |Rsb| and |xpEHH| values than for Rsb and xpEHH. Therefore, using the EHH‐based and F_st_ methods can be complementary to each other. Only limited overlap of CSS between different methods has been reported (Cavanagh et al., 2013; Joukhadar et al., 2019; Gaire et al., 2020), further supporting the approach of using multiple selective sweep detection methods to generate complementary information.

One should exercise caution against overinterpreting selection patterns before conducting additional validation studies. The CSS should be considered as plausible hypotheses of selection only. Combining pedigree information with the various tests for selective sweeps may be better at detecting selection signatures that are relevant to specific breeding programs (Fradgley et al., 2019; Hao et al., 2017). Every breeding program has a few historically successful varieties and important parents that form the genetic baseline for their target production region. One approach to validate the candidate regions in narrowly defined populations could involve (a) identifying key ancestral varieties using pedigree information, (b) defining ancestral alleles based on the genotypes of ancestral varieties, and (c) then conducting genomic scans for selection to remove the false positives from the current study.

## AUTHOR CONTRIBUTIONS

**Sajal R. Sthapit**: Data curation; formal analysis; investigation; methodology; project administration; visualization; writing—original draft; writing—review and editing. **Travis M. Ruff**: Methodology. **Marcus A. Hooker**: Data curation; methodology; software. **Bosen Zhang**: Software. **Xianran Li**: Supervision. **Deven R. See**: Conceptualization; funding acquisition; methodology; project administration; resources; software; supervision; writing—review and editing.

## CONFLICT OF INTEREST STATEMENT

The authors declare no conflicts of interest.

## Supporting information



Supplemental Table S1. Wheat variety panel used for the study.
Supplemental Table S2. Number of varieties and variety release years in populations used for F_st_, Rsb, and xpEHH calculations.
Supplemental Table S3. Sequences for known informative markers and their physical positions on the ‘Chinese Spring’ wheat reference genome version 2.1.
Supplemental Table S4. Candidate selective sweeps in U.S. wheat populations.

**Supplemental Figure S1**. Comparison of standard Gaussian distribution against the observed distribution of genomewide Rsb and xpEHH values for all 63 population pairs compared.
**Supplemental Figure S2**. Normal Q‐Q plots of observed distribution of genomewide Rsb and xpEHH values for all 63 population pairs compared.

**Supplemental Figure S3**. Map of candidate selective sweeps (CSS) in U.S. wheat populations due to selection over time. Each population pair was obtained by splitting a population (e.g., HRS with 150 varieties) into two halves, one of older varieties (e.g., HRS1 with 75 oldest varieties) and the other of newer varieties (e.g., HRS2 with 75 newest varieties). F_st_, Rsb, and xpEHH were computed for these pairs. The linkage blocks from left to right show CSS in population pairs with a) both spring and winter varieties, b) just spring varieties, and c) just winter varieties. Physical positions in Mbp for the start of the sweep are on the left side of the bar. The right side includes end position of the CSS, name of the population selected in (spr, spring; win, winter; eas, Eastern; gpl, the Great Plains; nor, Northern, pac, the Pacific; pnw, the Pacific Northwest; HRS, hard red spring; HRW, hard red winter; SRW, soft red winter; SWS, soft white spring; SWW, soft white winter), growth habit (B, S, and W for both, spring, and winter), statistic and its maximum value, PIC values in target and reference population, major allele frequencies in the target and reference population, and CSS serial number. Red, green, and blue color of the label indicate CSS detected using F_st_, Rsb, and xpEHH, respectively. Size of the label corresponds with the size of the CSS. Location of known genes are indicated by (***) and F and L refer to the physical positions of the first and last SNP genotyped on the chromosome.

**Supplemental Figure S4**. Map of candidate selective sweeps (CSS) in U.S. wheat due to selection across regional, state, and market class populations. The linkage blocks from left to right show CSS in population pairs with a) both spring and winter varieties, b) just spring varieties, and c) just winter varieties. Physical positions in Mbp for the start of the CSS are on the left side of the bar. The right side includes end position of the CSS, name of the population selected in (eas, Eastern; gpl, the Great Plains; nor, Northern, pac, the Pacific; pnw, the Pacific Northwest; HRS, hard red spring; HRW, hard red winter; SRW, soft red winter; SWS, soft white spring; SWW, soft white winter), growth habit (B, S, and W for both, spring, and winter), statistic and its maximum value, PIC values in target and reference population, major allele frequencies in the target and reference population, and CSS serial number. Red, green, and blue color of the label indicate CSS detected using F_st_, Rsb, and xpEHH, respectively. Size of the label corresponds with the size of the CSS. Location of known genes are indicated by (***) and F and L refer to the physical positions of the first and last SNP genotyped on the chromosome.

**Supplemental Figure S5**. Distribution of 24,033 loci used in the analysis across the wheat chromosomes. Each bin size is 2.5Mbp and the dashed line represents the best estimated location of centromere (IWGSC et al., 2018).

## Data Availability

The relevant genotyping data, variety details, R Markdown files, knitted R Markdown html files, and output files along with documentation from the analysis are available at https://doi.org/10.5061/dryad.ghx3ffbx0.
