# Supplementary material for: Candidate selective sweeps in US wheat populations
Source: Plant Genome. 2024 Sep 25;17(4):e20513. doi: 10.1002/tpg2.20513 (PMC11628914; doi:10.1002/tpg2.20513)
Supplement: Supplementary file 1 [file TPG2-17-e20513-s001.pdf]

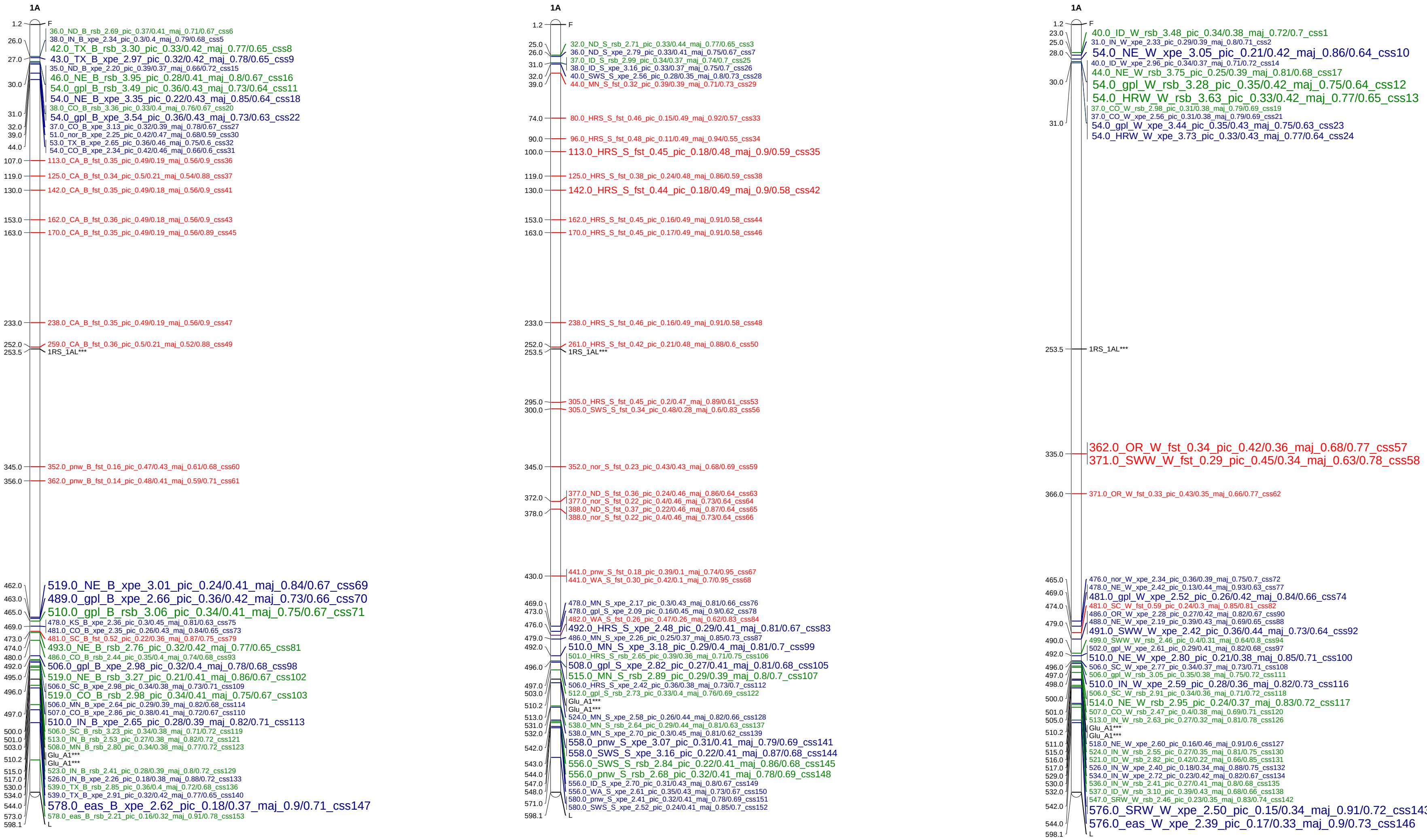

1A
F
36.0_ND_B_rsb_2.69_pic_0.37/0.41_maj_0.71/0.67_css6
38.0_IN_B_xpe_2.34_pic_0.3/0.4_maj_0.79/0.68_css5
42.0_TX_B_rsb_3.30_pic_0.33/0.42_maj_0.77/0.65_css8
43.0_TX_B_xpe_2.97_pic_0.32/0.42_maj_0.78/0.65_css9
35.0_ND_B_xpe_2.20_pic_0.39/0.37_maj_0.66/0.72_css15
46.0_NE_B_rsb_3.95_pic_0.28/0.41_maj_0.8/0.67_css16
54.0_gpl_B_rsb_3.49_pic_0.36/0.43_maj_0.73/0.64_css11
54.0_NE_B_xpe_3.35_pic_0.22/0.43_maj_0.85/0.64_css18
38.0_CO_B_rsb_3.36_pic_0.33/0.4_maj_0.76/0.67_css20
54.0_gpl_B_xpe_3.54_pic_0.36/0.43_maj_0.73/0.63_css22
37.0_CO_B_xpe_3.13_pic_0.32/0.39_maj_0.78/0.67_css27
51.0_nor_B_xpe_2.25_pic_0.42/0.47_maj_0.68/0.59_css30
53.0_TX_B_xpe_2.65_pic_0.36/0.46_maj_0.75/0.6_css32
54.0_CO_B_xpe_2.34_pic_0.42/0.46_maj_0.66/0.6_css31
113.0_CA_B_fst_0.35_pic_0.49/0.19_maj_0.56/0.9_css36
125.0_CA_B_fst_0.34_pic_0.5/0.21_maj_0.54/0.88_css37
142.0_CA_B_fst_0.35_pic_0.49/0.18_maj_0.56/0.9_css41
162.0_CA_B_fst_0.36_pic_0.49/0.18_maj_0.56/0.9_css43
170.0_CA_B_fst_0.35_pic_0.49/0.19_maj_0.56/0.89_css45
238.0_CA_B_fst_0.35_pic_0.49/0.19_maj_0.56/0.9_css47
259.0_CA_B_fst_0.36_pic_0.5/0.21_maj_0.52/0.88_css49
1RS_1AL***
352.0_pnw_B_fst_0.16_pic_0.47/0.43_maj_0.61/0.68_css60
362.0_pnw_B_fst_0.14_pic_0.48/0.41_maj_0.59/0.71_css61
519.0_NE_B_xpe_3.01_pic_0.24/0.41_maj_0.84/0.67_css69
489.0_gpl_B_xpe_2.66_pic_0.36/0.42_maj_0.73/0.66_css70
510.0_gpl_B_rsb_3.06_pic_0.34/0.41_maj_0.75/0.67_css71
478.0_KS_B_xpe_2.36_pic_0.3/0.45_maj_0.81/0.63_css75
481.0_CO_B_xpe_2.35_pic_0.26/0.43_maj_0.84/0.65_css73
481.0_SC_B_fst_0.52_pic_0.22/0.36_maj_0.87/0.75_css79
493.0_NE_B_rsb_2.76_pic_0.32/0.42_maj_0.77/0.65_css81
486.0_CO_B_rsb_2.44_pic_0.35/0.4_maj_0.74/0.68_css93
506.0_gpl_B_xpe_2.98_pic_0.32/0.4_maj_0.78/0.68_css98
519.0_NE_B_rsb_3.27_pic_0.21/0.41_maj_0.86/0.67_css102
506.0_SC_B_xpe_2.98_pic_0.34/0.38_maj_0.73/0.71_css109
519.0_CO_B_rsb_2.98_pic_0.34/0.41_maj_0.75/0.67_css103
506.0_MN_B_xpe_2.64_pic_0.29/0.39_maj_0.82/0.68_css114
507.0_CO_B_xpe_2.86_pic_0.38/0.41_maj_0.72/0.67_css110
510.0_IN_B_xpe_2.65_pic_0.28/0.39_maj_0.82/0.71_css113
506.0_SC_B_rsb_3.23_pic_0.34/0.38_maj_0.71/0.72_css119
513.0_IN_B_rsb_2.53_pic_0.27/0.38_maj_0.82/0.72_css121
508.0_MN_B_rsb_2.80_pic_0.34/0.38_maj_0.77/0.72_css123
Glu_A1***
Glu_A1***
523.0_IN_B_rsb_2.41_pic_0.28/0.39_maj_0.8/0.72_css129
526.0_IN_B_xpe_2.26_pic_0.18/0.38_maj_0.88/0.72_css133
539.0_TX_B_rsb_2.85_pic_0.36/0.4_maj_0.72/0.68_css136
539.0_TX_B_xpe_2.91_pic_0.32/0.42_maj_0.77/0.65_css140
578.0_eas_B_xpe_2.62_pic_0.18/0.37_maj_0.9/0.71_css147
578.0_eas_B_rsb_2.21_pic_0.16/0.32_maj_0.91/0.78_css153
L
1A
F
32.0_ND_S_rsb_2.71_pic_0.33/0.44_maj_0.77/0.65_css3
36.0_ND_S_xpe_2.79_pic_0.33/0.41_maj_0.75/0.67_css7
37.0_ID_S_rsb_2.99_pic_0.34/0.37_maj_0.74/0.7_css25
38.0_ID_S_xpe_3.16_pic_0.33/0.37_maj_0.75/0.7_css26
40.0_SWS_S_xpe_2.56_pic_0.28/0.35_maj_0.8/0.73_css28
44.0_MN_S_fst_0.32_pic_0.39/0.39_maj_0.71/0.73_css29
80.0_HRS_S_fst_0.46_pic_0.15/0.49_maj_0.92/0.57_css33
96.0_HRS_S_fst_0.48_pic_0.11/0.49_maj_0.94/0.55_css34
113.0_HRS_S_fst_0.45_pic_0.18/0.48_maj_0.9/0.59_css35
125.0_HRS_S_fst_0.38_pic_0.24/0.48_maj_0.86/0.59_css38
142.0_HRS_S_fst_0.44_pic_0.18/0.49_maj_0.9/0.58_css42
162.0_HRS_S_fst_0.45_pic_0.16/0.49_maj_0.91/0.58_css44
170.0_HRS_S_fst_0.45_pic_0.17/0.49_maj_0.91/0.58_css46
238.0_HRS_S_fst_0.46_pic_0.16/0.49_maj_0.91/0.58_css48
261.0_HRS_S_fst_0.42_pic_0.21/0.48_maj_0.88/0.6_css50
1RS_1AL***
305.0_HRS_S_fst_0.45_pic_0.2/0.47_maj_0.89/0.61_css53
305.0_SWS_S_fst_0.34_pic_0.48/0.28_maj_0.6/0.83_css56
352.0_nor_S_fst_0.23_pic_0.43/0.43_maj_0.68/0.69_css59
377.0_ND_S_fst_0.36_pic_0.24/0.46_maj_0.86/0.64_css63
377.0_nor_S_fst_0.22_pic_0.4/0.46_maj_0.73/0.64_css64
388.0_ND_S_fst_0.37_pic_0.22/0.46_maj_0.87/0.64_css65
388.0_nor_S_fst_0.22_pic_0.4/0.46_maj_0.73/0.64_css66
441.0_pnw_S_fst_0.18_pic_0.39/0.1_maj_0.74/0.95_css67
441.0_WA_S_fst_0.30_pic_0.42/0.1_maj_0.7/0.95_css68
478.0_MN_S_xpe_2.17_pic_0.3/0.43_maj_0.81/0.66_css76
478.0_gpl_S_xpe_2.09_pic_0.16/0.45_maj_0.9/0.62_css78
482.0_WA_S_fst_0.26_pic_0.47/0.26_maj_0.62/0.83_css84
492.0_HRS_S_xpe_2.48_pic_0.29/0.41_maj_0.81/0.67_css83
486.0_MN_S_xpe_2.26_pic_0.25/0.37_maj_0.85/0.73_css87
510.0_MN_S_xpe_3.18_pic_0.29/0.4_maj_0.81/0.7_css99
501.0_HRS_S_rsb_2.65_pic_0.39/0.36_maj_0.71/0.75_css106
508.0_gpl_S_xpe_2.82_pic_0.27/0.41_maj_0.81/0.68_css105
515.0_MN_S_rsb_2.89_pic_0.29/0.39_maj_0.8/0.7_css107
506.0_HRS_S_xpe_2.42_pic_0.36/0.38_maj_0.73/0.7_css112
512.0_gpl_S_rsb_2.73_pic_0.33/0.4_maj_0.76/0.69_css122
Glu_A1***
Glu_A1***
524.0_MN_S_xpe_2.58_pic_0.26/0.44_maj_0.82/0.66_css128
538.0_MN_S_rsb_2.64_pic_0.29/0.44_maj_0.81/0.63_css137
538.0_MN_S_xpe_2.70_pic_0.3/0.45_maj_0.81/0.62_css139
558.0_pnw_S_xpe_3.07_pic_0.31/0.41_maj_0.79/0.69_css141
558.0_SWS_S_xpe_3.16_pic_0.22/0.41_maj_0.87/0.68_css144
556.0_SWS_S_rsb_2.84_pic_0.22/0.41_maj_0.86/0.68_css145
556.0_pnw_S_rsb_2.68_pic_0.32/0.41_maj_0.78/0.69_css148
556.0_ID_S_xpe_2.70_pic_0.31/0.43_maj_0.8/0.67_css149
556.0_WA_S_xpe_2.61_pic_0.35/0.43_maj_0.73/0.67_css150
580.0_pnw_S_xpe_2.41_pic_0.32/0.41_maj_0.78/0.69_css151
580.0_SWS_S_xpe_2.52_pic_0.24/0.41_maj_0.85/0.7_css152
L
1A
F
40.0_ID_W_rsb_3.48_pic_0.34/0.38_maj_0.72/0.7_css1
31.0_IN_W_xpe_2.33_pic_0.29/0.39_maj_0.8/0.71_css2
54.0_NE_W_xpe_3.05_pic_0.21/0.42_maj_0.86/0.64_css10
40.0_ID_W_xpe_2.96_pic_0.34/0.37_maj_0.71/0.72_css14
44.0_NE_W_rsb_3.75_pic_0.25/0.39_maj_0.81/0.68_css17
54.0_gpl_W_rsb_3.28_pic_0.35/0.42_maj_0.75/0.64_css12
54.0_HRW_W_rsb_3.63_pic_0.33/0.42_maj_0.77/0.65_css13
37.0_CO_W_rsb_2.98_pic_0.31/0.38_maj_0.79/0.69_css19
37.0_CO_W_xpe_2.56_pic_0.31/0.38_maj_0.79/0.69_css21
54.0_gpl_W_xpe_3.44_pic_0.35/0.43_maj_0.75/0.63_css23
54.0_HRW_W_xpe_3.73_pic_0.33/0.43_maj_0.77/0.64_css24
1RS_1AL***
362.0_OR_W_fst_0.34_pic_0.42/0.36_maj_0.68/0.77_css57
371.0_SWW_W_fst_0.29_pic_0.45/0.34_maj_0.63/0.78_css58
371.0_OR_W_fst_0.33_pic_0.43/0.35_maj_0.66/0.77_css62
476.0_nor_W_xpe_2.34_pic_0.36/0.39_maj_0.75/0.7_css72
478.0_NE_W_xpe_2.42_pic_0.13/0.44_maj_0.93/0.63_css77
481.0_gpl_W_xpe_2.52_pic_0.26/0.42_maj_0.84/0.66_css74
481.0_SC_W_fst_0.59_pic_0.24/0.3_maj_0.85/0.81_css82
486.0_OR_W_xpe_2.28_pic_0.27/0.42_maj_0.82/0.67_css90
488.0_NE_W_xpe_2.19_pic_0.39/0.43_maj_0.69/0.65_css88
491.0_SWW_W_xpe_2.42_pic_0.36/0.44_maj_0.73/0.64_css92
499.0_SWW_W_rsb_2.46_pic_0.4/0.31_maj_0.64/0.8_css94
502.0_gpl_W_xpe_2.61_pic_0.29/0.41_maj_0.82/0.68_css97
510.0_NE_W_xpe_2.80_pic_0.21/0.38_maj_0.85/0.71_css100
506.0_SC_W_xpe_2.77_pic_0.34/0.37_maj_0.73/0.71_css108
506.0_gpl_W_rsb_3.05_pic_0.35/0.38_maj_0.75/0.72_css111
510.0_IN_W_xpe_2.59_pic_0.28/0.36_maj_0.82/0.73_css116
506.0_SC_W_rsb_2.91_pic_0.34/0.36_maj_0.71/0.72_css118
514.0_NE_W_rsb_2.95_pic_0.24/0.37_maj_0.83/0.72_css117
507.0_CO_W_rsb_2.47_pic_0.4/0.38_maj_0.69/0.71_css120
513.0_IN_W_rsb_2.63_pic_0.27/0.32_maj_0.81/0.78_css126
Glu_A1***
Glu_A1***
518.0_NE_W_xpe_2.60_pic_0.16/0.46_maj_0.91/0.6_css127
524.0_IN_W_rsb_2.55_pic_0.27/0.35_maj_0.81/0.75_css130
521.0_ID_W_rsb_2.82_pic_0.42/0.22_maj_0.66/0.85_css131
526.0_IN_W_xpe_2.40_pic_0.18/0.34_maj_0.88/0.75_css132
534.0_IN_W_xpe_2.72_pic_0.23/0.42_maj_0.82/0.67_css134
536.0_IN_W_rsb_2.41_pic_0.27/0.41_maj_0.8/0.68_css135
537.0_ID_W_rsb_3.10_pic_0.39/0.43_maj_0.68/0.66_css138
547.0_SRW_W_rsb_2.46_pic_0.23/0.35_maj_0.83/0.74_css142
576.0_SRW_W_xpe_2.50_pic_0.15/0.34_maj_0.91/0.72_css143
576.0_eas_W_xpe_2.39_pic_0.17/0.33_maj_0.9/0.73_css146
L

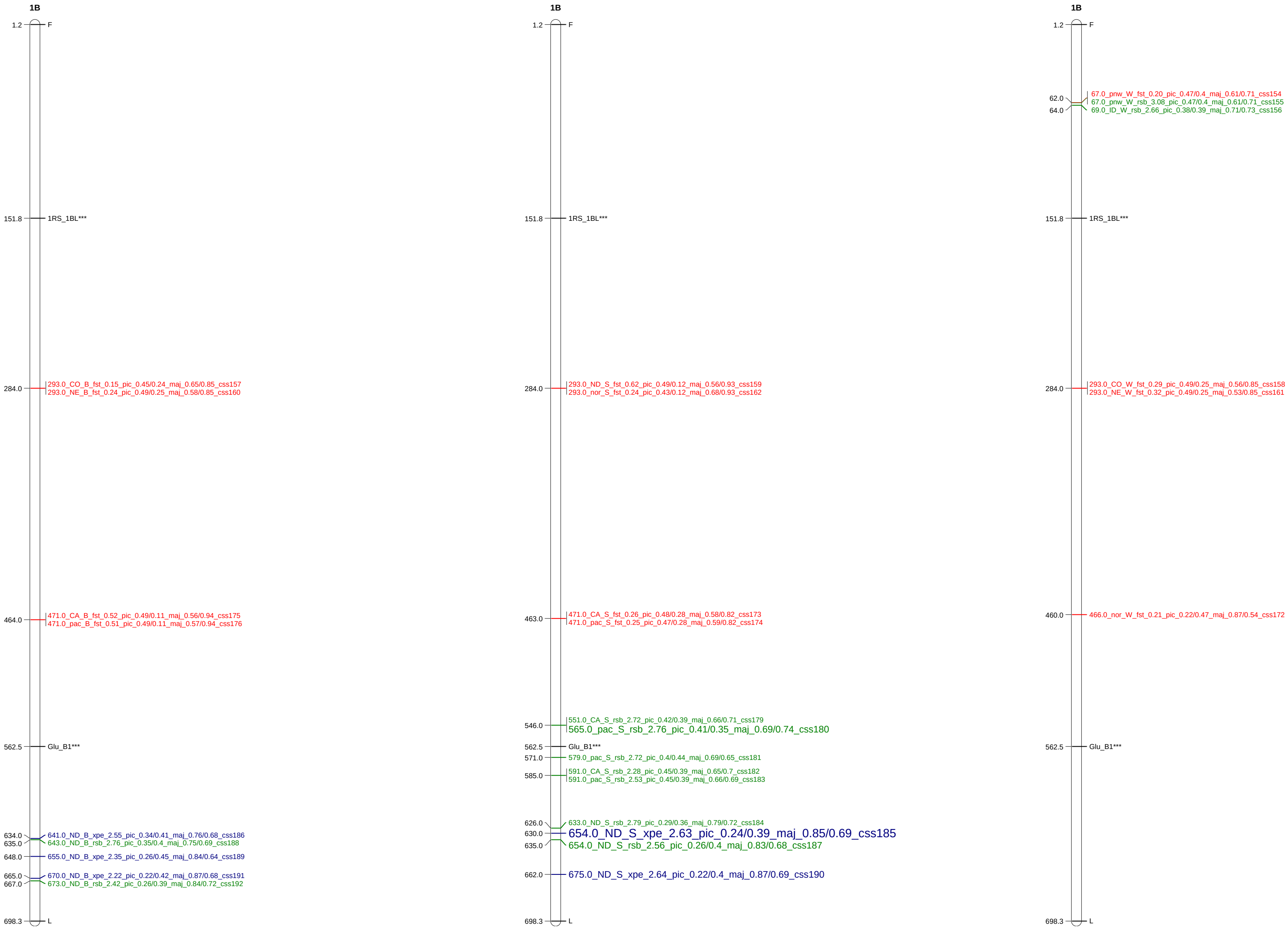
1B
1.2 F
151.8 1RS_1BL***
284.0 293.0_CO_B_fst_0.15_pic_0.45/0.24_maj_0.65/0.85_css157
293.0_NE_B_fst_0.24_pic_0.49/0.25_maj_0.58/0.85_css160
464.0 471.0_CA_B_fst_0.52_pic_0.49/0.11_maj_0.56/0.94_css175
471.0_pac_B_fst_0.51_pic_0.49/0.11_maj_0.57/0.94_css176
562.5 Glu_B1***
634.0 641.0_ND_B_xpe_2.55_pic_0.34/0.41_maj_0.76/0.68_css186
635.0 643.0_ND_B_rsb_2.76_pic_0.35/0.4_maj_0.75/0.69_css188
648.0 655.0_ND_B_xpe_2.35_pic_0.26/0.45_maj_0.84/0.64_css189
665.0 670.0_ND_B_xpe_2.22_pic_0.22/0.42_maj_0.87/0.68_css191
667.0 673.0_ND_B_rsb_2.42_pic_0.26/0.39_maj_0.84/0.72_css192
698.3 L
1B
1.2 F
151.8 1RS_1BL***
284.0 293.0_ND_S_fst_0.62_pic_0.49/0.12_maj_0.56/0.93_css159
293.0_nor_S_fst_0.24_pic_0.43/0.12_maj_0.68/0.93_css162
463.0 471.0_CA_S_fst_0.26_pic_0.48/0.28_maj_0.58/0.82_css173
471.0_pac_S_fst_0.25_pic_0.47/0.28_maj_0.59/0.82_css174
546.0 551.0_CA_S_rsb_2.72_pic_0.42/0.39_maj_0.66/0.71_css179
565.0_pac_S_rsb_2.76_pic_0.41/0.35_maj_0.69/0.74_css180
562.5 Glu_B1***
571.0 579.0_pac_S_rsb_2.72_pic_0.4/0.44_maj_0.69/0.65_css181
585.0 591.0_CA_S_rsb_2.28_pic_0.45/0.39_maj_0.65/0.7_css182
591.0_pac_S_rsb_2.53_pic_0.45/0.39_maj_0.66/0.69_css183
626.0 633.0_ND_S_rsb_2.79_pic_0.29/0.36_maj_0.79/0.72_css184
630.0 654.0_ND_S_xpe_2.63_pic_0.24/0.39_maj_0.85/0.69_css185
635.0 654.0_ND_S_rsb_2.56_pic_0.26/0.4_maj_0.83/0.68_css187
662.0 675.0_ND_S_xpe_2.64_pic_0.22/0.4_maj_0.87/0.69_css190
698.3 L
1B
1.2 F
62.0 67.0_pnw_W_fst_0.20_pic_0.47/0.4_maj_0.61/0.71_css154
67.0_pnw_W_rsb_3.08_pic_0.47/0.4_maj_0.61/0.71_css155
64.0 69.0_ID_W_rsb_2.66_pic_0.38/0.39_maj_0.71/0.73_css156
151.8 1RS_1BL***
284.0 293.0_CO_W_fst_0.29_pic_0.49/0.25_maj_0.56/0.85_css158
293.0_NE_W_fst_0.32_pic_0.49/0.25_maj_0.53/0.85_css161
460.0 466.0_nor_W_fst_0.21_pic_0.22/0.47_maj_0.87/0.54_css172
562.5 Glu_B1***
698.3 L

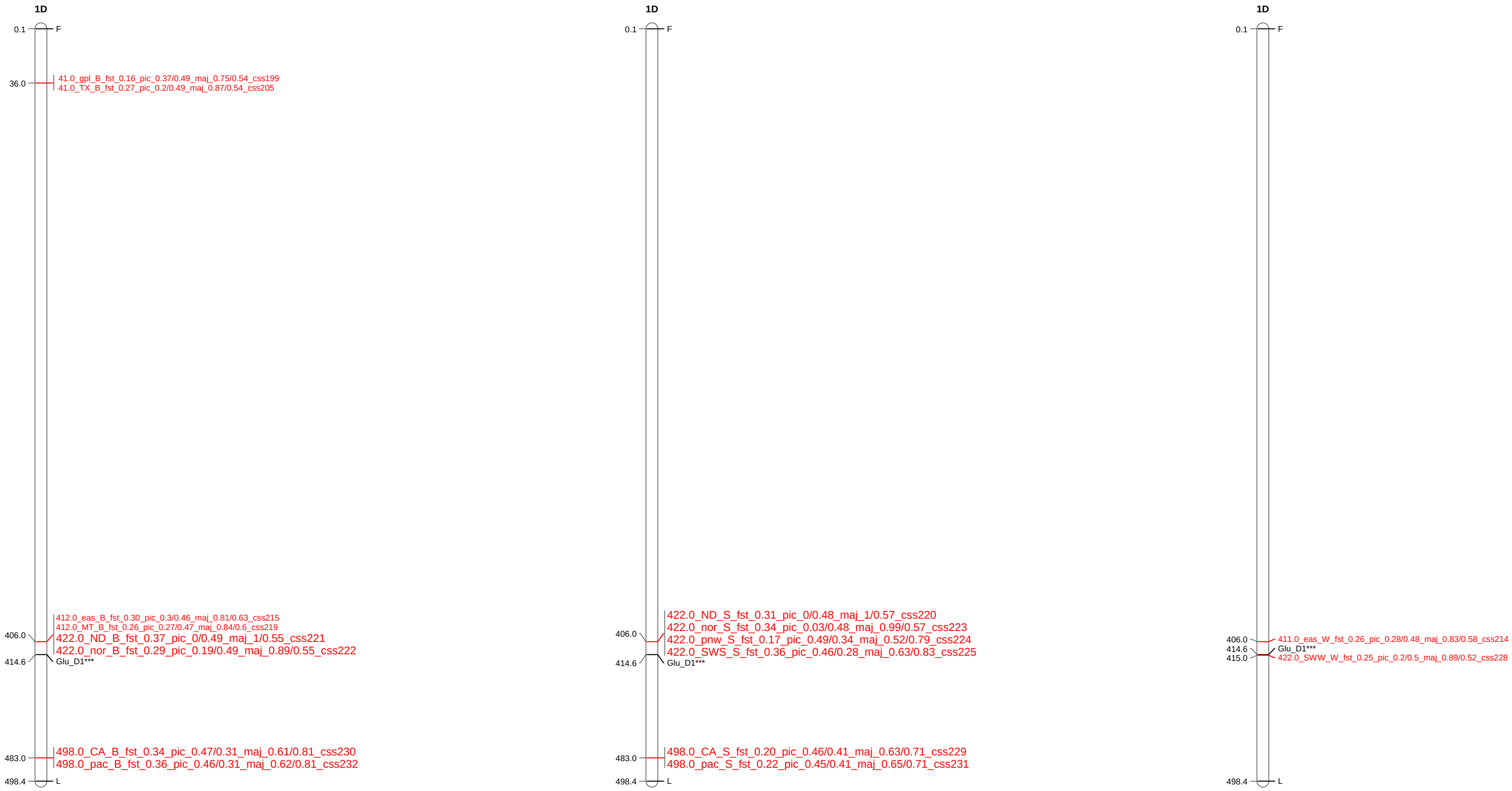
1D
0.1
F
36.0
41.0_gpl_B_fst_0.16_pic_0.37/0.49_maj_0.75/0.54_css199
41.0_TX_B_fst_0.27_pic_0.2/0.49_maj_0.87/0.54_css205
406.0
414.6
412.0_eas_B_fst_0.30_pic_0.3/0.46_maj_0.81/0.63_css215
412.0_MT_B_fst_0.26_pic_0.27/0.47_maj_0.84/0.6_css219
422.0_ND_B_fst_0.37_pic_0/0.49_maj_1/0.55_css221
422.0_nor_B_fst_0.29_pic_0.19/0.49_maj_0.89/0.55_css222
Glu_D1***
483.0
498.0_CA_B_fst_0.34_pic_0.47/0.31_maj_0.61/0.81_css230
498.0_pac_B_fst_0.36_pic_0.46/0.31_maj_0.62/0.81_css232
498.4
L
1D
0.1
F
406.0
414.6
422.0_ND_S_fst_0.31_pic_0/0.48_maj_1/0.57_css220
422.0_nor_S_fst_0.34_pic_0.03/0.48_maj_0.99/0.57_css223
422.0_pnw_S_fst_0.17_pic_0.49/0.34_maj_0.52/0.79_css224
422.0_SWS_S_fst_0.36_pic_0.46/0.28_maj_0.63/0.83_css225
Glu_D1***
483.0
498.0_CA_S_fst_0.20_pic_0.46/0.41_maj_0.63/0.71_css229
498.0_pac_S_fst_0.22_pic_0.45/0.41_maj_0.65/0.71_css231
498.4
L
1D
0.1
F
406.0
414.6
415.0
411.0_eas_W_fst_0.26_pic_0.28/0.48_maj_0.83/0.58_css214
Glu_D1***
422.0_SWW_W_fst_0.25_pic_0.2/0.5_maj_0.89/0.52_css228
498.4
L

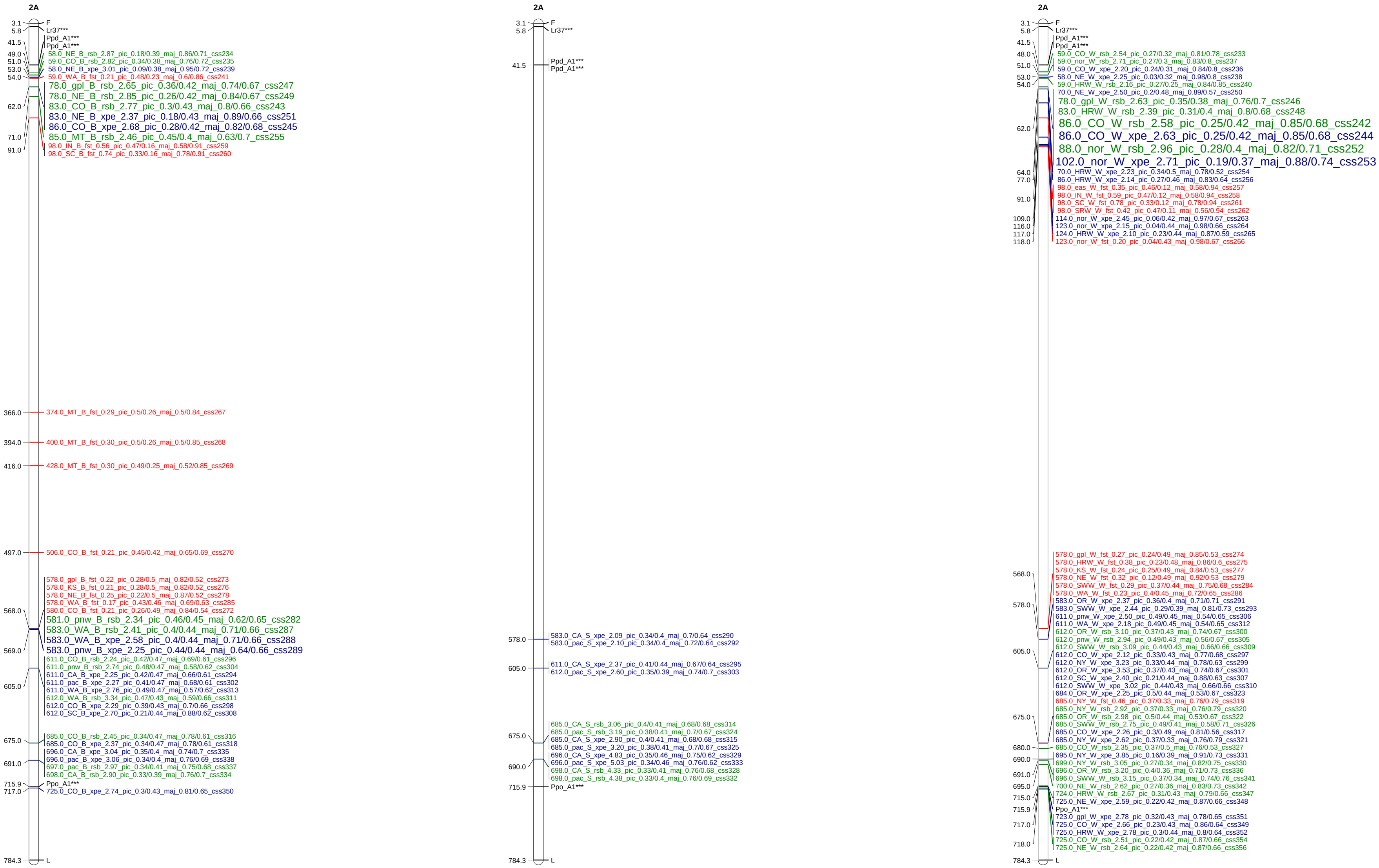

2A
3.1
5.8
41.5
49.0
51.0
53.0
54.0
62.0
71.0
91.0
366.0
394.0
416.0
497.0
568.0
569.0
605.0
675.0
691.0
715.9
717.0
784.3
F
Lr37***
Ppd_A1***
Ppd_A1***
58.0_NE_B_rsb_2.87_pic_0.18/0.39_maj_0.86/0.71_css234
59.0_CO_B_rsb_2.82_pic_0.34/0.38_maj_0.76/0.72_css235
58.0_NE_B_xpe_3.01_pic_0.09/0.38_maj_0.95/0.72_css239
59.0_WA_B_fst_0.21_pic_0.48/0.23_maj_0.6/0.86_css241
78.0_gpl_B_rsb_2.65_pic_0.36/0.42_maj_0.74/0.67_css247
78.0_NE_B_rsb_2.85_pic_0.26/0.42_maj_0.84/0.67_css249
83.0_CO_B_rsb_2.77_pic_0.3/0.43_maj_0.8/0.66_css243
83.0_NE_B_xpe_2.37_pic_0.18/0.43_maj_0.89/0.66_css251
86.0_CO_B_xpe_2.68_pic_0.28/0.42_maj_0.82/0.68_css245
85.0_MT_B_rsb_2.46_pic_0.45/0.4_maj_0.63/0.7_css255
98.0_IN_B_fst_0.56_pic_0.47/0.16_maj_0.58/0.91_css259
98.0_SC_B_fst_0.74_pic_0.33/0.16_maj_0.78/0.91_css260
374.0_MT_B_fst_0.29_pic_0.5/0.26_maj_0.5/0.84_css267
400.0_MT_B_fst_0.30_pic_0.5/0.26_maj_0.5/0.85_css268
428.0_MT_B_fst_0.30_pic_0.49/0.25_maj_0.52/0.85_css269
506.0_CO_B_fst_0.21_pic_0.45/0.42_maj_0.65/0.69_css270
578.0_gpl_B_fst_0.22_pic_0.28/0.5_maj_0.82/0.52_css273
578.0_KS_B_fst_0.21_pic_0.28/0.5_maj_0.82/0.52_css276
578.0_NE_B_fst_0.25_pic_0.22/0.5_maj_0.87/0.52_css278
578.0_WA_B_fst_0.17_pic_0.43/0.46_maj_0.69/0.63_css285
580.0_CO_B_fst_0.21_pic_0.26/0.49_maj_0.84/0.54_css272
581.0_pnw_B_rsb_2.34_pic_0.46/0.45_maj_0.62/0.65_css282
583.0_WA_B_rsb_2.41_pic_0.4/0.44_maj_0.71/0.66_css287
583.0_WA_B_xpe_2.58_pic_0.4/0.44_maj_0.71/0.66_css288
583.0_pnw_B_xpe_2.25_pic_0.44/0.44_maj_0.64/0.66_css289
611.0_CO_B_rsb_2.24_pic_0.42/0.47_maj_0.69/0.61_css296
611.0_pnw_B_rsb_2.74_pic_0.48/0.47_maj_0.58/0.62_css304
611.0_CA_B_xpe_2.25_pic_0.42/0.47_maj_0.66/0.61_css294
611.0_pac_B_xpe_2.27_pic_0.41/0.47_maj_0.68/0.61_css302
611.0_WA_B_xpe_2.76_pic_0.49/0.47_maj_0.57/0.62_css313
612.0_WA_B_rsb_3.34_pic_0.47/0.43_maj_0.59/0.66_css311
612.0_CO_B_xpe_2.29_pic_0.39/0.43_maj_0.7/0.66_css298
612.0_SC_B_xpe_2.70_pic_0.21/0.44_maj_0.88/0.62_css308
685.0_CO_B_rsb_2.45_pic_0.34/0.47_maj_0.78/0.61_css316
685.0_CO_B_xpe_2.37_pic_0.34/0.47_maj_0.78/0.61_css318
696.0_CA_B_xpe_3.04_pic_0.35/0.4_maj_0.74/0.7_css335
696.0_pac_B_xpe_3.06_pic_0.34/0.4_maj_0.76/0.69_css338
697.0_pac_B_rsb_2.97_pic_0.34/0.41_maj_0.75/0.68_css337
698.0_CA_B_rsb_2.90_pic_0.33/0.39_maj_0.76/0.7_css334
Ppo_A1***
725.0_CO_B_xpe_2.74_pic_0.3/0.43_maj_0.81/0.65_css350
L
2A
3.1
5.8
41.5
578.0
605.0
675.0
690.0
715.9
784.3
F
Lr37***
Ppd_A1***
Ppd_A1***
583.0_CA_S_xpe_2.09_pic_0.34/0.4_maj_0.7/0.64_css290
583.0_pac_S_xpe_2.10_pic_0.34/0.4_maj_0.72/0.64_css292
611.0_CA_S_xpe_2.37_pic_0.41/0.44_maj_0.67/0.64_css295
612.0_pac_S_xpe_2.60_pic_0.35/0.39_maj_0.74/0.7_css303
685.0_CA_S_rsb_3.06_pic_0.4/0.41_maj_0.68/0.68_css314
685.0_pac_S_rsb_3.19_pic_0.38/0.41_maj_0.7/0.67_css324
685.0_CA_S_xpe_2.90_pic_0.4/0.41_maj_0.68/0.68_css315
685.0_pac_S_xpe_3.20_pic_0.38/0.41_maj_0.7/0.67_css325
696.0_CA_S_xpe_4.83_pic_0.35/0.46_maj_0.75/0.62_css329
696.0_pac_S_xpe_5.03_pic_0.34/0.46_maj_0.76/0.62_css333
698.0_CA_S_rsb_4.33_pic_0.33/0.41_maj_0.76/0.68_css328
698.0_pac_S_rsb_4.38_pic_0.33/0.4_maj_0.76/0.69_css332
Ppo_A1***
L
2A
3.1
5.8
41.5
48.0
51.0
53.0
54.0
62.0
64.0
77.0
91.0
109.0
116.0
117.0
118.0
568.0
578.0
605.0
675.0
680.0
690.0
691.0
695.0
715.0
715.9
717.0
718.0
784.3
F
Lr37***
Ppd_A1***
Ppd_A1***
59.0_CO_W_rsb_2.54_pic_0.27/0.32_maj_0.81/0.78_css233
59.0_nor_W_rsb_2.71_pic_0.27/0.3_maj_0.83/0.8_css237
59.0_CO_W_xpe_2.20_pic_0.24/0.31_maj_0.84/0.8_css236
58.0_NE_W_xpe_2.25_pic_0.03/0.32_maj_0.98/0.8_css238
59.0_HRW_W_rsb_2.16_pic_0.27/0.25_maj_0.84/0.85_css240
70.0_NE_W_xpe_2.50_pic_0.2/0.48_maj_0.89/0.57_css250
78.0_gpl_W_rsb_2.63_pic_0.35/0.38_maj_0.76/0.7_css246
83.0_HRW_W_rsb_2.39_pic_0.31/0.4_maj_0.8/0.68_css248
86.0_CO_W_rsb_2.58_pic_0.25/0.42_maj_0.85/0.68_css242
86.0_CO_W_xpe_2.63_pic_0.25/0.42_maj_0.85/0.68_css244
88.0_nor_W_rsb_2.96_pic_0.28/0.4_maj_0.82/0.71_css252
102.0_nor_W_xpe_2.71_pic_0.19/0.37_maj_0.88/0.74_css253
70.0_HRW_W_xpe_2.23_pic_0.34/0.5_maj_0.78/0.52_css254
86.0_HRW_W_xpe_2.14_pic_0.27/0.46_maj_0.83/0.64_css256
98.0_eas_W_fst_0.35_pic_0.46/0.12_maj_0.58/0.94_css257
98.0_IN_W_fst_0.59_pic_0.47/0.12_maj_0.58/0.94_css258
98.0_SC_W_fst_0.78_pic_0.33/0.12_maj_0.78/0.94_css261
98.0_SRW_W_fst_0.42_pic_0.47/0.11_maj_0.56/0.94_css262
114.0_nor_W_xpe_2.45_pic_0.06/0.42_maj_0.97/0.67_css263
123.0_nor_W_xpe_2.15_pic_0.04/0.44_maj_0.98/0.66_css264
124.0_HRW_W_xpe_2.10_pic_0.23/0.44_maj_0.87/0.59_css265
123.0_nor_W_fst_0.20_pic_0.04/0.43_maj_0.98/0.67_css266
578.0_gpl_W_fst_0.27_pic_0.24/0.49_maj_0.85/0.53_css274
578.0_HRW_W_fst_0.38_pic_0.23/0.48_maj_0.86/0.6_css275
578.0_KS_W_fst_0.24_pic_0.25/0.49_maj_0.84/0.53_css277
578.0_NE_W_fst_0.32_pic_0.12/0.49_maj_0.92/0.53_css279
578.0_SWW_W_fst_0.29_pic_0.37/0.44_maj_0.75/0.68_css284
578.0_WA_W_fst_0.23_pic_0.4/0.45_maj_0.72/0.65_css286
583.0_OR_W_xpe_2.37_pic_0.36/0.4_maj_0.71/0.71_css291
583.0_SWW_W_xpe_2.44_pic_0.29/0.39_maj_0.81/0.73_css293
611.0_pnw_W_xpe_2.50_pic_0.49/0.45_maj_0.54/0.65_css306
611.0_WA_W_xpe_2.18_pic_0.49/0.45_maj_0.54/0.65_css312
612.0_OR_W_rsb_3.10_pic_0.37/0.43_maj_0.74/0.67_css300
612.0_pnw_W_rsb_2.94_pic_0.49/0.43_maj_0.56/0.67_css305
612.0_SWW_W_rsb_3.09_pic_0.44/0.43_maj_0.66/0.66_css309
612.0_CO_W_xpe_2.12_pic_0.33/0.43_maj_0.77/0.68_css297
612.0_NY_W_xpe_3.23_pic_0.33/0.44_maj_0.78/0.63_css299
612.0_OR_W_xpe_3.53_pic_0.37/0.43_maj_0.74/0.67_css301
612.0_SC_W_xpe_2.40_pic_0.21/0.44_maj_0.88/0.63_css307
612.0_SWW_W_xpe_3.02_pic_0.44/0.43_maj_0.66/0.66_css310
684.0_OR_W_xpe_2.25_pic_0.5/0.44_maj_0.53/0.67_css323
685.0_NY_W_fst_0.46_pic_0.37/0.33_maj_0.76/0.79_css319
685.0_NY_W_rsb_2.92_pic_0.37/0.33_maj_0.76/0.79_css320
685.0_OR_W_rsb_2.98_pic_0.5/0.44_maj_0.53/0.67_css322
685.0_SWW_W_rsb_2.75_pic_0.49/0.41_maj_0.58/0.71_css326
685.0_CO_W_xpe_2.26_pic_0.3/0.49_maj_0.81/0.56_css317
685.0_NY_W_xpe_2.62_pic_0.37/0.33_maj_0.76/0.79_css321
685.0_CO_W_rsb_2.35_pic_0.37/0.5_maj_0.76/0.53_css327
695.0_NY_W_xpe_3.85_pic_0.16/0.39_maj_0.91/0.73_css331
699.0_NY_W_rsb_3.05_pic_0.27/0.34_maj_0.82/0.75_css330
696.0_OR_W_rsb_3.20_pic_0.4/0.36_maj_0.71/0.73_css336
696.0_SWW_W_rsb_3.15_pic_0.37/0.34_maj_0.74/0.76_css341
700.0_NE_W_rsb_2.62_pic_0.27/0.36_maj_0.83/0.73_css342
724.0_HRW_W_rsb_2.67_pic_0.31/0.43_maj_0.79/0.66_css347
725.0_NE_W_xpe_2.59_pic_0.22/0.42_maj_0.87/0.66_css348
Ppo_A1***
723.0_gpl_W_xpe_2.78_pic_0.32/0.43_maj_0.78/0.65_css351
725.0_CO_W_xpe_2.66_pic_0.23/0.43_maj_0.86/0.64_css349
725.0_HRW_W_xpe_2.78_pic_0.3/0.44_maj_0.8/0.64_css352
725.0_CO_W_rsb_2.51_pic_0.22/0.42_maj_0.87/0.66_css354
725.0_NE_W_rsb_2.64_pic_0.22/0.42_maj_0.87/0.66_css356
L

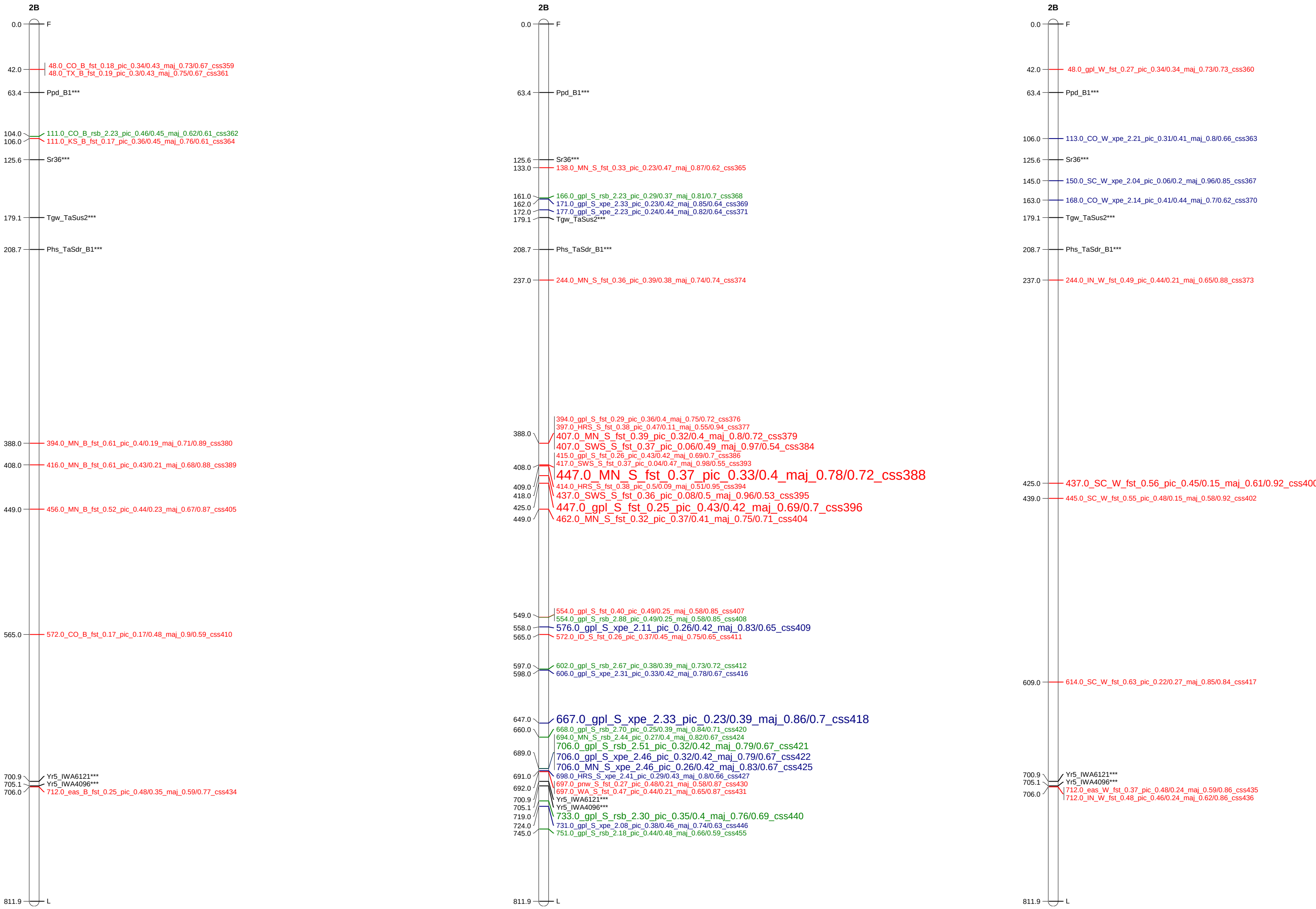
2B
0.0 F
42.0 48.0_CO_B_fst_0.18_pic_0.34/0.43_maj_0.73/0.67_css359
48.0_TX_B_fst_0.19_pic_0.3/0.43_maj_0.75/0.67_css361
63.4 Ppd_B1***
104.0 111.0_CO_B_rsb_2.23_pic_0.46/0.45_maj_0.62/0.61_css362
106.0 111.0_KS_B_fst_0.17_pic_0.36/0.45_maj_0.76/0.61_css364
125.6 Sr36***
179.1 Tgw_TaSus2***
208.7 Phs_TaSdr_B1***
388.0 394.0_MN_B_fst_0.61_pic_0.4/0.19_maj_0.71/0.89_css380
408.0 416.0_MN_B_fst_0.61_pic_0.43/0.21_maj_0.68/0.88_css389
449.0 456.0_MN_B_fst_0.52_pic_0.44/0.23_maj_0.67/0.87_css405
565.0 572.0_CO_B_fst_0.17_pic_0.17/0.48_maj_0.9/0.59_css410
700.9 Yr5_IWA6121***
705.1 Yr5_IWA4096***
706.0 712.0_eas_B_fst_0.25_pic_0.48/0.35_maj_0.59/0.77_css434
811.9 L
2B
0.0 F
63.4 Ppd_B1***
125.6 Sr36***
133.0 138.0_MN_S_fst_0.33_pic_0.23/0.47_maj_0.87/0.62_css365
161.0 166.0_gpl_S_rsb_2.23_pic_0.29/0.37_maj_0.81/0.7_css368
162.0 171.0_gpl_S_xpe_2.33_pic_0.23/0.42_maj_0.85/0.64_css369
172.0 177.0_gpl_S_xpe_2.23_pic_0.24/0.44_maj_0.82/0.64_css371
179.1 Tgw_TaSus2***
208.7 Phs_TaSdr_B1***
237.0 244.0_MN_S_fst_0.36_pic_0.39/0.38_maj_0.74/0.74_css374
394.0_gpl_S_fst_0.29_pic_0.36/0.4_maj_0.75/0.72_css376
388.0 397.0_HRS_S_fst_0.38_pic_0.47/0.11_maj_0.55/0.94_css377
407.0_MN_S_fst_0.39_pic_0.32/0.4_maj_0.8/0.72_css379
407.0_SWS_S_fst_0.37_pic_0.06/0.49_maj_0.97/0.54_css384
415.0_gpl_S_fst_0.26_pic_0.43/0.42_maj_0.69/0.7_css386
408.0 417.0_SWS_S_fst_0.37_pic_0.04/0.47_maj_0.98/0.55_css393
447.0_MN_S_fst_0.37_pic_0.33/0.4_maj_0.78/0.72_css388
409.0 414.0_HRS_S_fst_0.38_pic_0.5/0.09_maj_0.51/0.95_css394
418.0 437.0_SWS_S_fst_0.36_pic_0.08/0.5_maj_0.96/0.53_css395
425.0 447.0_gpl_S_fst_0.25_pic_0.43/0.42_maj_0.69/0.7_css396
449.0 462.0_MN_S_fst_0.32_pic_0.37/0.41_maj_0.75/0.71_css404
549.0 554.0_gpl_S_fst_0.40_pic_0.49/0.25_maj_0.58/0.85_css407
554.0_gpl_S_rsb_2.88_pic_0.49/0.25_maj_0.58/0.85_css408
558.0 576.0_gpl_S_xpe_2.11_pic_0.26/0.42_maj_0.83/0.65_css409
565.0 572.0_ID_S_fst_0.26_pic_0.37/0.45_maj_0.75/0.65_css411
597.0 602.0_gpl_S_rsb_2.67_pic_0.38/0.39_maj_0.73/0.72_css412
598.0 606.0_gpl_S_xpe_2.31_pic_0.33/0.42_maj_0.78/0.67_css416
647.0 667.0_gpl_S_xpe_2.33_pic_0.23/0.39_maj_0.86/0.7_css418
660.0 668.0_gpl_S_rsb_2.70_pic_0.25/0.39_maj_0.84/0.71_css420
694.0_MN_S_rsb_2.44_pic_0.27/0.4_maj_0.82/0.67_css424
689.0 706.0_gpl_S_rsb_2.51_pic_0.32/0.42_maj_0.79/0.67_css421
706.0_gpl_S_xpe_2.46_pic_0.32/0.42_maj_0.79/0.67_css422
706.0_MN_S_xpe_2.46_pic_0.26/0.42_maj_0.83/0.67_css425
691.0 698.0_HRS_S_xpe_2.41_pic_0.29/0.43_maj_0.8/0.66_css427
692.0 697.0_pnw_S_fst_0.27_pic_0.48/0.21_maj_0.58/0.87_css430
697.0_WA_S_fst_0.47_pic_0.44/0.21_maj_0.65/0.87_css431
700.9 Yr5_IWA6121***
705.1 Yr5_IWA4096***
719.0 733.0_gpl_S_rsb_2.30_pic_0.35/0.4_maj_0.76/0.69_css440
724.0 731.0_gpl_S_xpe_2.08_pic_0.38/0.46_maj_0.74/0.63_css446
745.0 751.0_gpl_S_rsb_2.18_pic_0.44/0.48_maj_0.66/0.59_css455
811.9 L
2B
0.0 F
42.0 48.0_gpl_W_fst_0.27_pic_0.34/0.34_maj_0.73/0.73_css360
63.4 Ppd_B1***
106.0 113.0_CO_W_xpe_2.21_pic_0.31/0.41_maj_0.8/0.66_css363
125.6 Sr36***
145.0 150.0_SC_W_xpe_2.04_pic_0.06/0.2_maj_0.96/0.85_css367
163.0 168.0_CO_W_xpe_2.14_pic_0.41/0.44_maj_0.7/0.62_css370
179.1 Tgw_TaSus2***
208.7 Phs_TaSdr_B1***
237.0 244.0_IN_W_fst_0.49_pic_0.44/0.21_maj_0.65/0.88_css373
425.0 437.0_SC_W_fst_0.56_pic_0.45/0.15_maj_0.61/0.92_css400
439.0 445.0_SC_W_fst_0.55_pic_0.48/0.15_maj_0.58/0.92_css402
609.0 614.0_SC_W_fst_0.63_pic_0.22/0.27_maj_0.85/0.84_css417
700.9 Yr5_IWA6121***
705.1 Yr5_IWA4096***
706.0 712.0_eas_W_fst_0.37_pic_0.48/0.24_maj_0.59/0.86_css435
712.0_IN_W_fst_0.48_pic_0.46/0.24_maj_0.62/0.86_css436
811.9 L

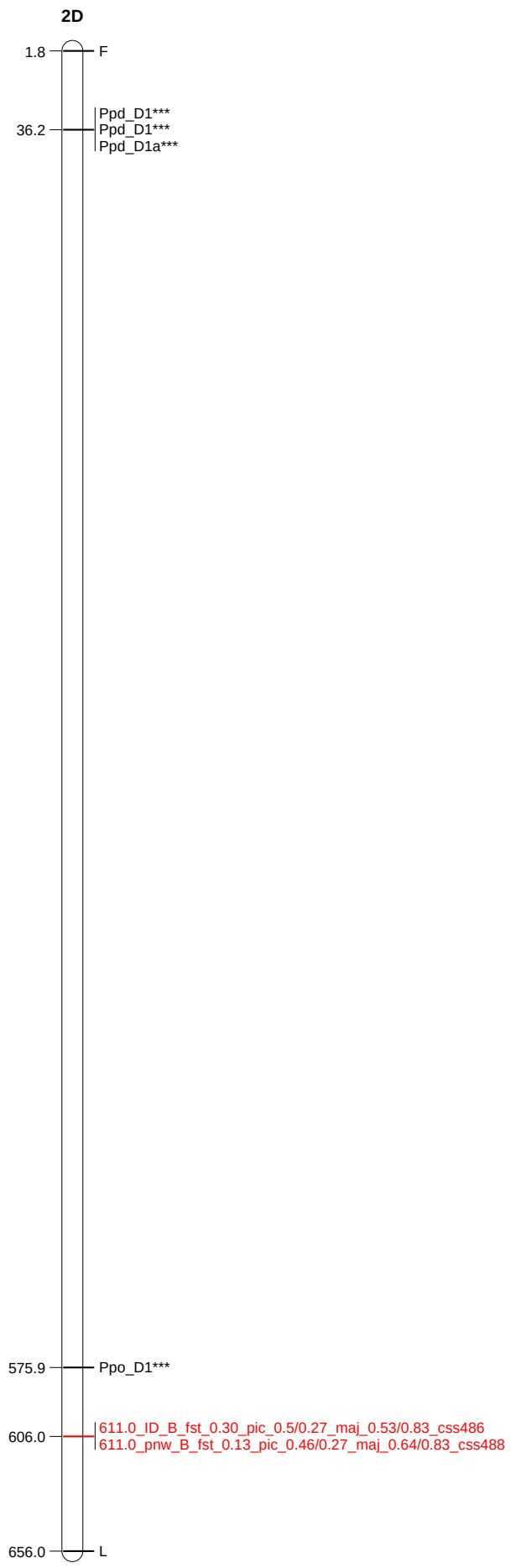
2D
1.8
F
36.2
Ppd_D1***
Ppd_D1***
Ppd_D1a***
575.9
Ppo_D1***
606.0
611.0_ID_B_fst_0.30_pic_0.5/0.27_maj_0.53/0.83_css486
611.0_pnw_B_fst_0.13_pic_0.46/0.27_maj_0.64/0.83_css488
656.0
L

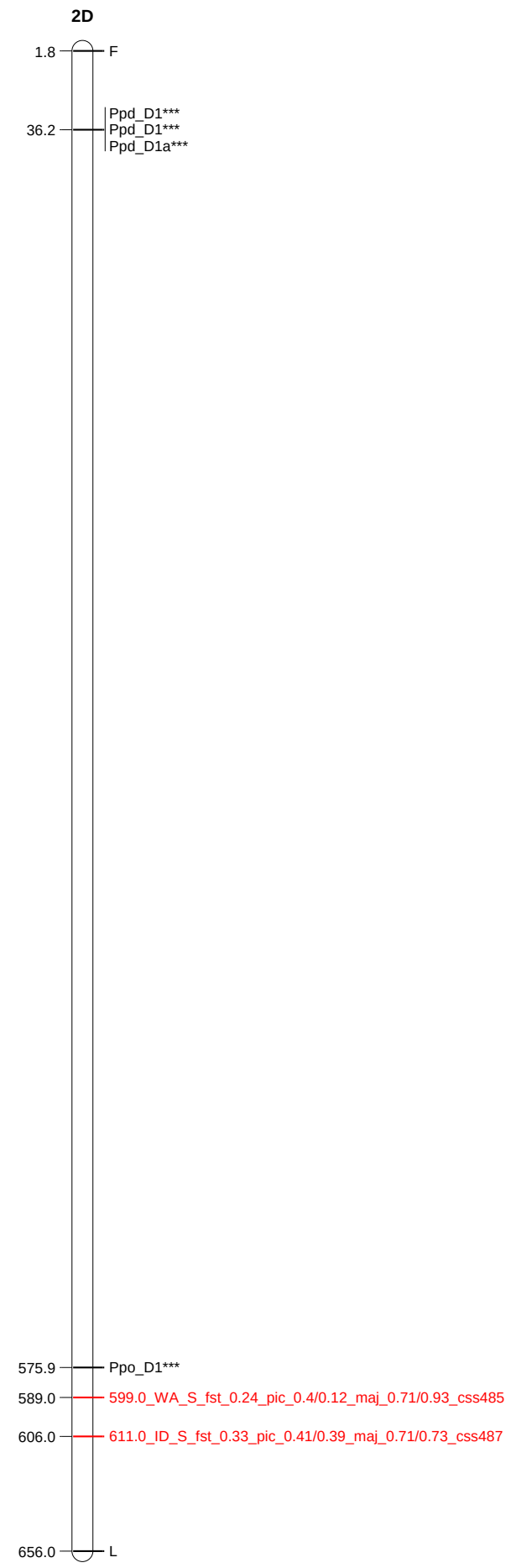
2D
1.8
F
36.2
Ppd_D1***
Ppd_D1***
Ppd_D1a***
575.9
Ppo_D1***
589.0
599.0_WA_S_fst_0.24_pic_0.4/0.12_maj_0.71/0.93_css485
606.0
611.0_ID_S_fst_0.33_pic_0.41/0.39_maj_0.71/0.73_css487
656.0
L

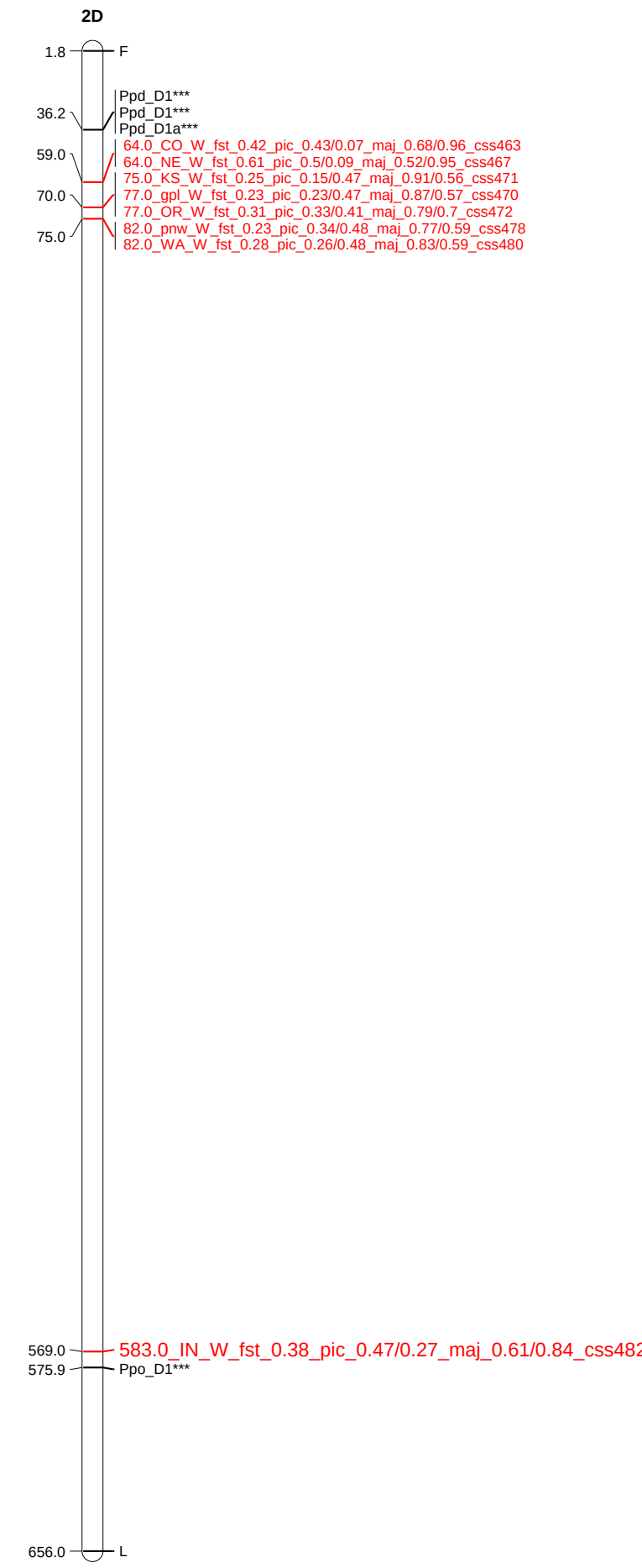
2D
1.8
F
Ppd_D1***
36.2
Ppd_D1***
Ppd_D1a***
64.0_CO_W_fst_0.42_pic_0.43/0.07_maj_0.68/0.96_css463
59.0
64.0_NE_W_fst_0.61_pic_0.5/0.09_maj_0.52/0.95_css467
75.0_KS_W_fst_0.25_pic_0.15/0.47_maj_0.91/0.56_css471
70.0
77.0_gpl_W_fst_0.23_pic_0.23/0.47_maj_0.87/0.57_css470
77.0_OR_W_fst_0.31_pic_0.33/0.41_maj_0.79/0.7_css472
75.0
82.0_pnw_W_fst_0.23_pic_0.34/0.48_maj_0.77/0.59_css478
82.0_WA_W_fst_0.28_pic_0.26/0.48_maj_0.83/0.59_css480
569.0
583.0_IN_W_fst_0.38_pic_0.47/0.27_maj_0.61/0.84_css482
575.9
Ppo_D1***
656.0
L

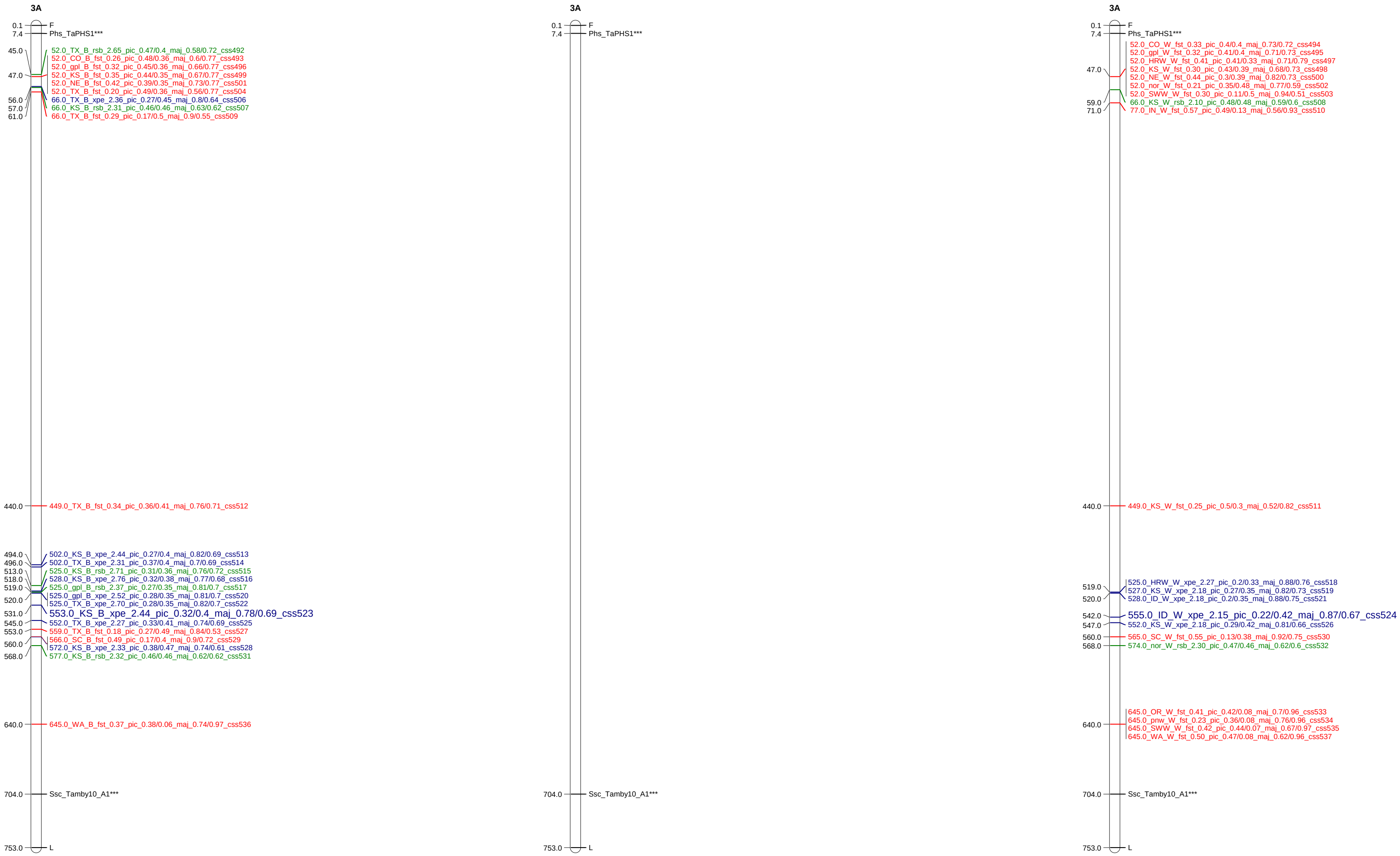
3A
0.1 F
7.4 Phs_TaPHS1***
45.0 52.0_TX_B_rsb_2.65_pic_0.47/0.4_maj_0.58/0.72_css492
52.0_CO_B_fst_0.26_pic_0.48/0.36_maj_0.6/0.77_css493
52.0_gpl_B_fst_0.32_pic_0.45/0.36_maj_0.66/0.77_css496
47.0 52.0_KS_B_fst_0.35_pic_0.44/0.35_maj_0.67/0.77_css499
52.0_NE_B_fst_0.42_pic_0.39/0.35_maj_0.73/0.77_css501
52.0_TX_B_fst_0.20_pic_0.49/0.36_maj_0.56/0.77_css504
56.0 66.0_TX_B_xpe_2.36_pic_0.27/0.45_maj_0.8/0.64_css506
57.0 66.0_KS_B_rsb_2.31_pic_0.46/0.46_maj_0.63/0.62_css507
61.0 66.0_TX_B_fst_0.29_pic_0.17/0.5_maj_0.9/0.55_css509
440.0 449.0_TX_B_fst_0.34_pic_0.36/0.41_maj_0.76/0.71_css512
494.0 502.0_KS_B_xpe_2.44_pic_0.27/0.4_maj_0.82/0.69_css513
496.0 502.0_TX_B_xpe_2.31_pic_0.37/0.4_maj_0.7/0.69_css514
513.0 525.0_KS_B_rsb_2.71_pic_0.31/0.36_maj_0.76/0.72_css515
518.0 528.0_KS_B_xpe_2.76_pic_0.32/0.38_maj_0.77/0.68_css516
519.0 525.0_gpl_B_rsb_2.37_pic_0.27/0.35_maj_0.81/0.7_css517
520.0 525.0_gpl_B_xpe_2.52_pic_0.28/0.35_maj_0.81/0.7_css520
525.0_TX_B_xpe_2.70_pic_0.28/0.35_maj_0.82/0.7_css522
531.0 553.0_KS_B_xpe_2.44_pic_0.32/0.4_maj_0.78/0.69_css523
545.0 552.0_TX_B_xpe_2.27_pic_0.33/0.41_maj_0.74/0.69_css525
553.0 559.0_TX_B_fst_0.18_pic_0.27/0.49_maj_0.84/0.53_css527
566.0_SC_B_fst_0.49_pic_0.17/0.4_maj_0.9/0.72_css529
560.0 572.0_KS_B_xpe_2.33_pic_0.38/0.47_maj_0.74/0.61_css528
568.0 577.0_KS_B_rsb_2.32_pic_0.46/0.46_maj_0.62/0.62_css531
640.0 645.0_WA_B_fst_0.37_pic_0.38/0.06_maj_0.74/0.97_css536
704.0 Ssc_Tamby10_A1***
753.0 L
3A
0.1 F
7.4 Phs_TaPHS1***
704.0 Ssc_Tamby10_A1***
753.0 L
3A
0.1 F
7.4 Phs_TaPHS1***
52.0_CO_W_fst_0.33_pic_0.4/0.4_maj_0.73/0.72_css494
52.0_gpl_W_fst_0.32_pic_0.41/0.4_maj_0.71/0.73_css495
52.0_HRW_W_fst_0.41_pic_0.41/0.33_maj_0.71/0.79_css497
47.0 52.0_KS_W_fst_0.30_pic_0.43/0.39_maj_0.68/0.73_css498
52.0_NE_W_fst_0.44_pic_0.3/0.39_maj_0.82/0.73_css500
52.0_nor_W_fst_0.21_pic_0.35/0.48_maj_0.77/0.59_css502
52.0_SWW_W_fst_0.30_pic_0.11/0.5_maj_0.94/0.51_css503
59.0 66.0_KS_W_rsb_2.10_pic_0.48/0.48_maj_0.59/0.6_css508
71.0 77.0_IN_W_fst_0.57_pic_0.49/0.13_maj_0.56/0.93_css510
440.0 449.0_KS_W_fst_0.25_pic_0.5/0.3_maj_0.52/0.82_css511
525.0_HRW_W_xpe_2.27_pic_0.2/0.33_maj_0.88/0.76_css518
519.0 527.0_KS_W_xpe_2.18_pic_0.27/0.35_maj_0.82/0.73_css519
520.0 528.0_ID_W_xpe_2.18_pic_0.2/0.35_maj_0.88/0.75_css521
542.0 555.0_ID_W_xpe_2.15_pic_0.22/0.42_maj_0.87/0.67_css524
547.0 552.0_KS_W_xpe_2.18_pic_0.29/0.42_maj_0.81/0.66_css526
560.0 565.0_SC_W_fst_0.55_pic_0.13/0.38_maj_0.92/0.75_css530
568.0 574.0_nor_W_rsb_2.30_pic_0.47/0.46_maj_0.62/0.6_css532
645.0_OR_W_fst_0.41_pic_0.42/0.08_maj_0.7/0.96_css533
645.0_pnw_W_fst_0.23_pic_0.36/0.08_maj_0.76/0.96_css534
640.0 645.0_SWW_W_fst_0.42_pic_0.44/0.07_maj_0.67/0.97_css535
645.0_WA_W_fst_0.50_pic_0.47/0.08_maj_0.62/0.96_css537
704.0 Ssc_Tamby10_A1***
753.0 L

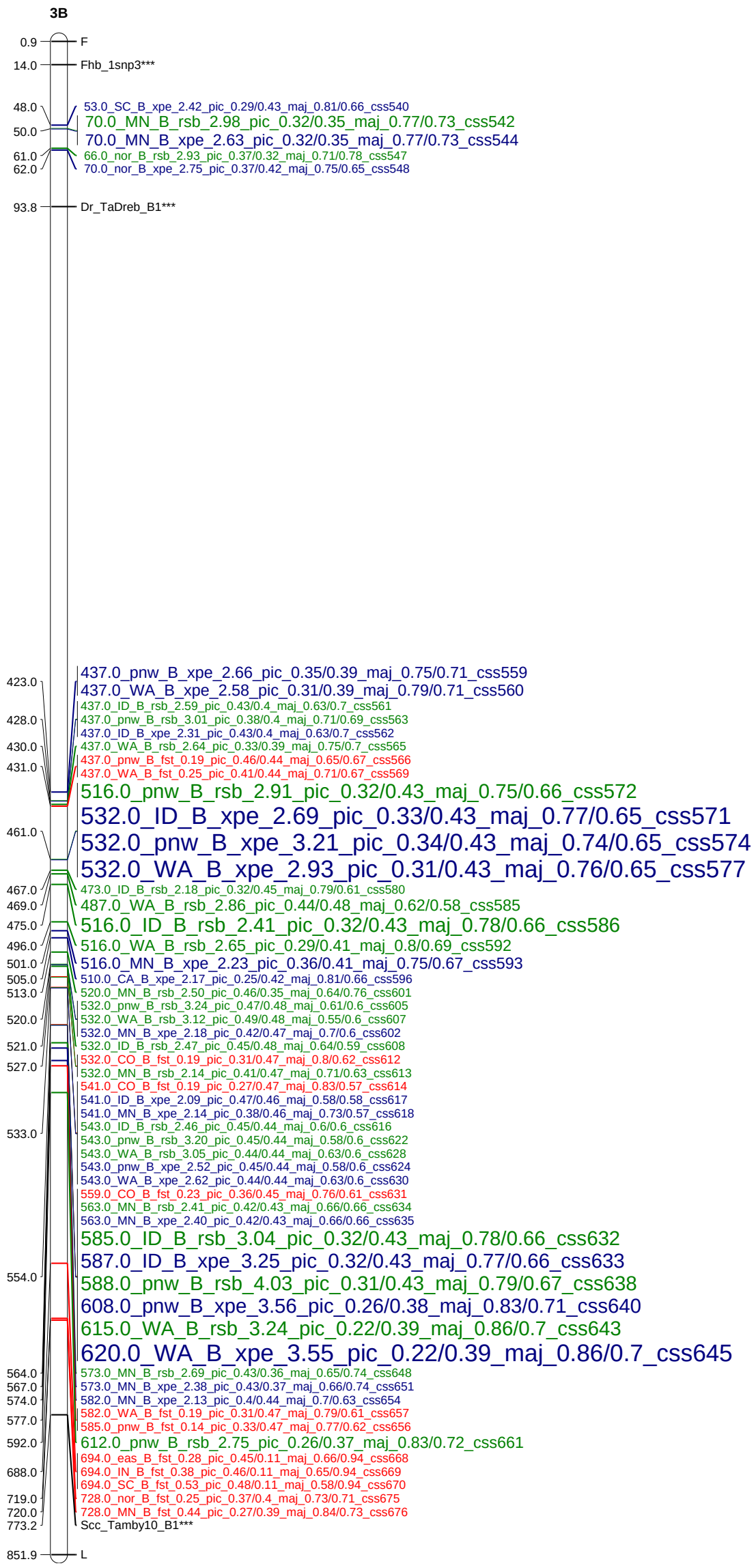
3B
0.9 F
14.0 Fhb_1snp3***
48.0 50.0 61.0 62.0
53.0_SC_B_xpe_2.42_pic_0.29/0.43_maj_0.81/0.66_css540
70.0_MN_B_rsb_2.98_pic_0.32/0.35_maj_0.77/0.73_css542
70.0_MN_B_xpe_2.63_pic_0.32/0.35_maj_0.77/0.73_css544
66.0_nor_B_rsb_2.93_pic_0.37/0.32_maj_0.71/0.78_css547
70.0_nor_B_xpe_2.75_pic_0.37/0.42_maj_0.75/0.65_css548
93.8 Dr_TaDreb_B1***
423.0 428.0 430.0 431.0 461.0 467.0 469.0 475.0 496.0 501.0 505.0 513.0 520.0 521.0 527.0 533.0 554.0 564.0 567.0 574.0 577.0 592.0 688.0 719.0 720.0 773.2
437.0_pnw_B_xpe_2.66_pic_0.35/0.39_maj_0.75/0.71_css559
437.0_WA_B_xpe_2.58_pic_0.31/0.39_maj_0.79/0.71_css560
437.0_ID_B_rsb_2.59_pic_0.43/0.4_maj_0.63/0.7_css561
437.0_pnw_B_rsb_3.01_pic_0.38/0.4_maj_0.71/0.69_css563
437.0_ID_B_xpe_2.31_pic_0.43/0.4_maj_0.63/0.7_css562
437.0_WA_B_rsb_2.64_pic_0.33/0.39_maj_0.75/0.7_css565
437.0_pnw_B_fst_0.19_pic_0.46/0.44_maj_0.65/0.67_css566
437.0_WA_B_fst_0.25_pic_0.41/0.44_maj_0.71/0.67_css569
516.0_pnw_B_rsb_2.91_pic_0.32/0.43_maj_0.75/0.66_css572
532.0_ID_B_xpe_2.69_pic_0.33/0.43_maj_0.77/0.65_css571
532.0_pnw_B_xpe_3.21_pic_0.34/0.43_maj_0.74/0.65_css574
532.0_WA_B_xpe_2.93_pic_0.31/0.43_maj_0.76/0.65_css577
473.0_ID_B_rsb_2.18_pic_0.32/0.45_maj_0.79/0.61_css580
487.0_WA_B_rsb_2.86_pic_0.44/0.48_maj_0.62/0.58_css585
516.0_ID_B_rsb_2.41_pic_0.32/0.43_maj_0.78/0.66_css586
516.0_WA_B_rsb_2.65_pic_0.29/0.41_maj_0.8/0.69_css592
516.0_MN_B_xpe_2.23_pic_0.36/0.41_maj_0.75/0.67_css593
510.0_CA_B_xpe_2.17_pic_0.25/0.42_maj_0.81/0.66_css596
520.0_MN_B_rsb_2.50_pic_0.46/0.35_maj_0.64/0.76_css601
532.0_pnw_B_rsb_3.24_pic_0.47/0.48_maj_0.61/0.6_css605
532.0_WA_B_rsb_3.12_pic_0.49/0.48_maj_0.55/0.6_css607
532.0_MN_B_xpe_2.18_pic_0.42/0.47_maj_0.7/0.6_css602
532.0_ID_B_rsb_2.47_pic_0.45/0.48_maj_0.64/0.59_css608
532.0_CO_B_fst_0.19_pic_0.31/0.47_maj_0.8/0.62_css612
532.0_MN_B_rsb_2.14_pic_0.41/0.47_maj_0.71/0.63_css613
541.0_CO_B_fst_0.19_pic_0.27/0.47_maj_0.83/0.57_css614
541.0_ID_B_xpe_2.09_pic_0.47/0.46_maj_0.58/0.58_css617
541.0_MN_B_xpe_2.14_pic_0.38/0.46_maj_0.73/0.57_css618
543.0_ID_B_rsb_2.46_pic_0.45/0.44_maj_0.6/0.6_css616
543.0_pnw_B_rsb_3.20_pic_0.45/0.44_maj_0.58/0.6_css622
543.0_WA_B_rsb_3.05_pic_0.44/0.44_maj_0.63/0.6_css628
543.0_pnw_B_xpe_2.52_pic_0.45/0.44_maj_0.58/0.6_css624
543.0_WA_B_xpe_2.62_pic_0.44/0.44_maj_0.63/0.6_css630
559.0_CO_B_fst_0.23_pic_0.36/0.45_maj_0.76/0.61_css631
563.0_MN_B_rsb_2.41_pic_0.42/0.43_maj_0.66/0.66_css634
563.0_MN_B_xpe_2.40_pic_0.42/0.43_maj_0.66/0.66_css635
585.0_ID_B_rsb_3.04_pic_0.32/0.43_maj_0.78/0.66_css632
587.0_ID_B_xpe_3.25_pic_0.32/0.43_maj_0.77/0.66_css633
588.0_pnw_B_rsb_4.03_pic_0.31/0.43_maj_0.79/0.67_css638
608.0_pnw_B_xpe_3.56_pic_0.26/0.38_maj_0.83/0.71_css640
615.0_WA_B_rsb_3.24_pic_0.22/0.39_maj_0.86/0.7_css643
620.0_WA_B_xpe_3.55_pic_0.22/0.39_maj_0.86/0.7_css645
573.0_MN_B_rsb_2.69_pic_0.43/0.36_maj_0.65/0.74_css648
573.0_MN_B_xpe_2.38_pic_0.43/0.37_maj_0.66/0.74_css651
582.0_MN_B_xpe_2.13_pic_0.4/0.44_maj_0.7/0.63_css654
582.0_WA_B_fst_0.19_pic_0.31/0.47_maj_0.79/0.61_css657
585.0_pnw_B_fst_0.14_pic_0.33/0.47_maj_0.77/0.62_css656
612.0_pnw_B_rsb_2.75_pic_0.26/0.37_maj_0.83/0.72_css661
694.0_eas_B_fst_0.28_pic_0.45/0.11_maj_0.66/0.94_css668
694.0_IN_B_fst_0.38_pic_0.46/0.11_maj_0.65/0.94_css669
694.0_SC_B_fst_0.53_pic_0.48/0.11_maj_0.58/0.94_css670
728.0_nor_B_fst_0.25_pic_0.37/0.4_maj_0.73/0.71_css675
728.0_MN_B_fst_0.44_pic_0.27/0.39_maj_0.84/0.73_css676
Scc_Tamby10_B1***
851.9 L

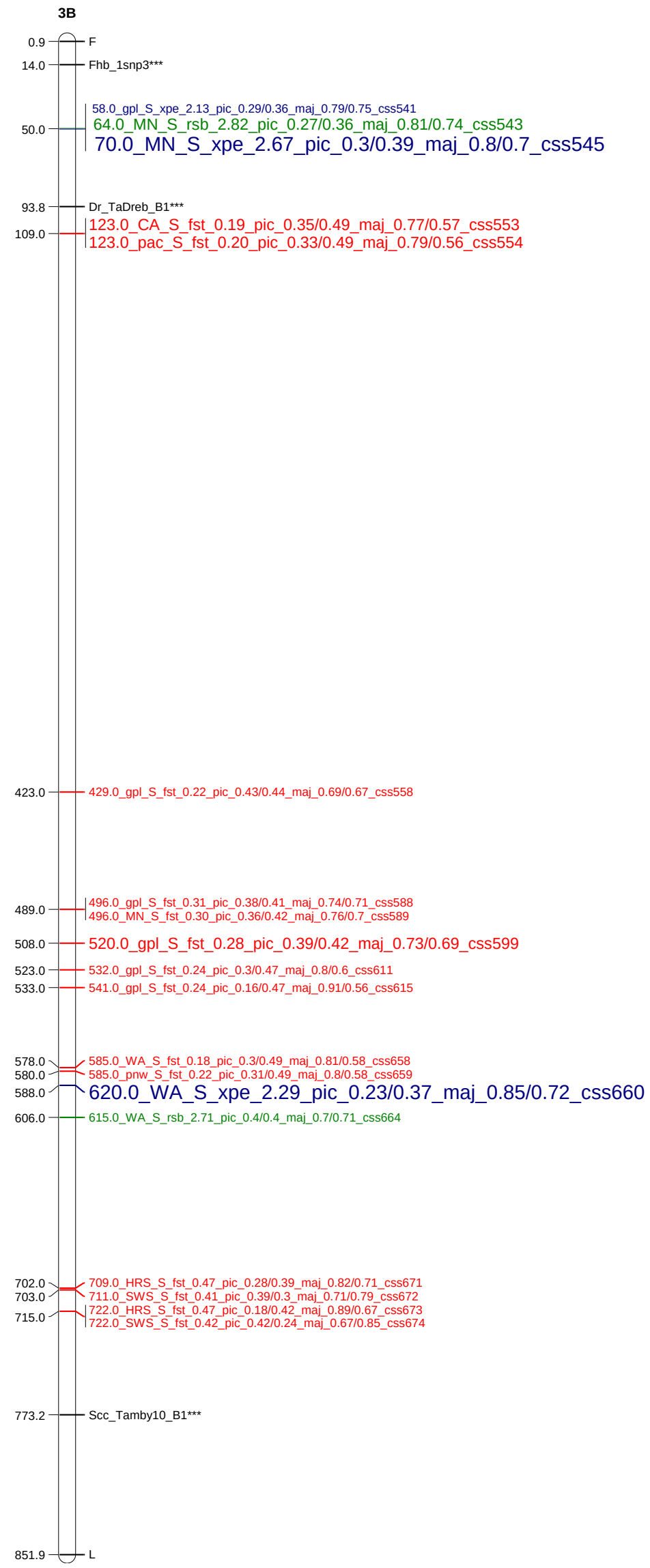
3B
0.9 F
14.0 Fhb_1snp3***
50.0
58.0_gpl_S_xpe_2.13_pic_0.29/0.36_maj_0.79/0.75_css541
64.0_MN_S_rsb_2.82_pic_0.27/0.36_maj_0.81/0.74_css543
70.0_MN_S_xpe_2.67_pic_0.3/0.39_maj_0.8/0.7_css545
93.8 Dr_TaDreb_B1***
109.0
123.0_CA_S_fst_0.19_pic_0.35/0.49_maj_0.77/0.57_css553
123.0_pac_S_fst_0.20_pic_0.33/0.49_maj_0.79/0.56_css554
423.0 429.0_gpl_S_fst_0.22_pic_0.43/0.44_maj_0.69/0.67_css558
489.0
496.0_gpl_S_fst_0.31_pic_0.38/0.41_maj_0.74/0.71_css588
496.0_MN_S_fst_0.30_pic_0.36/0.42_maj_0.76/0.7_css589
508.0 520.0_gpl_S_fst_0.28_pic_0.39/0.42_maj_0.73/0.69_css599
523.0 532.0_gpl_S_fst_0.24_pic_0.3/0.47_maj_0.8/0.6_css611
533.0 541.0_gpl_S_fst_0.24_pic_0.16/0.47_maj_0.91/0.56_css615
578.0 580.0 588.0
585.0_WA_S_fst_0.18_pic_0.3/0.49_maj_0.81/0.58_css658
585.0_pnw_S_fst_0.22_pic_0.31/0.49_maj_0.8/0.58_css659
620.0_WA_S_xpe_2.29_pic_0.23/0.37_maj_0.85/0.72_css660
606.0 615.0_WA_S_rsb_2.71_pic_0.4/0.4_maj_0.7/0.71_css664
702.0 703.0 715.0
709.0_HRS_S_fst_0.47_pic_0.28/0.39_maj_0.82/0.71_css671
711.0_SWS_S_fst_0.41_pic_0.39/0.3_maj_0.71/0.79_css672
722.0_HRS_S_fst_0.47_pic_0.18/0.42_maj_0.89/0.67_css673
722.0_SWS_S_fst_0.42_pic_0.42/0.24_maj_0.67/0.85_css674
773.2 Scc_Tamby10_B1***
851.9 L

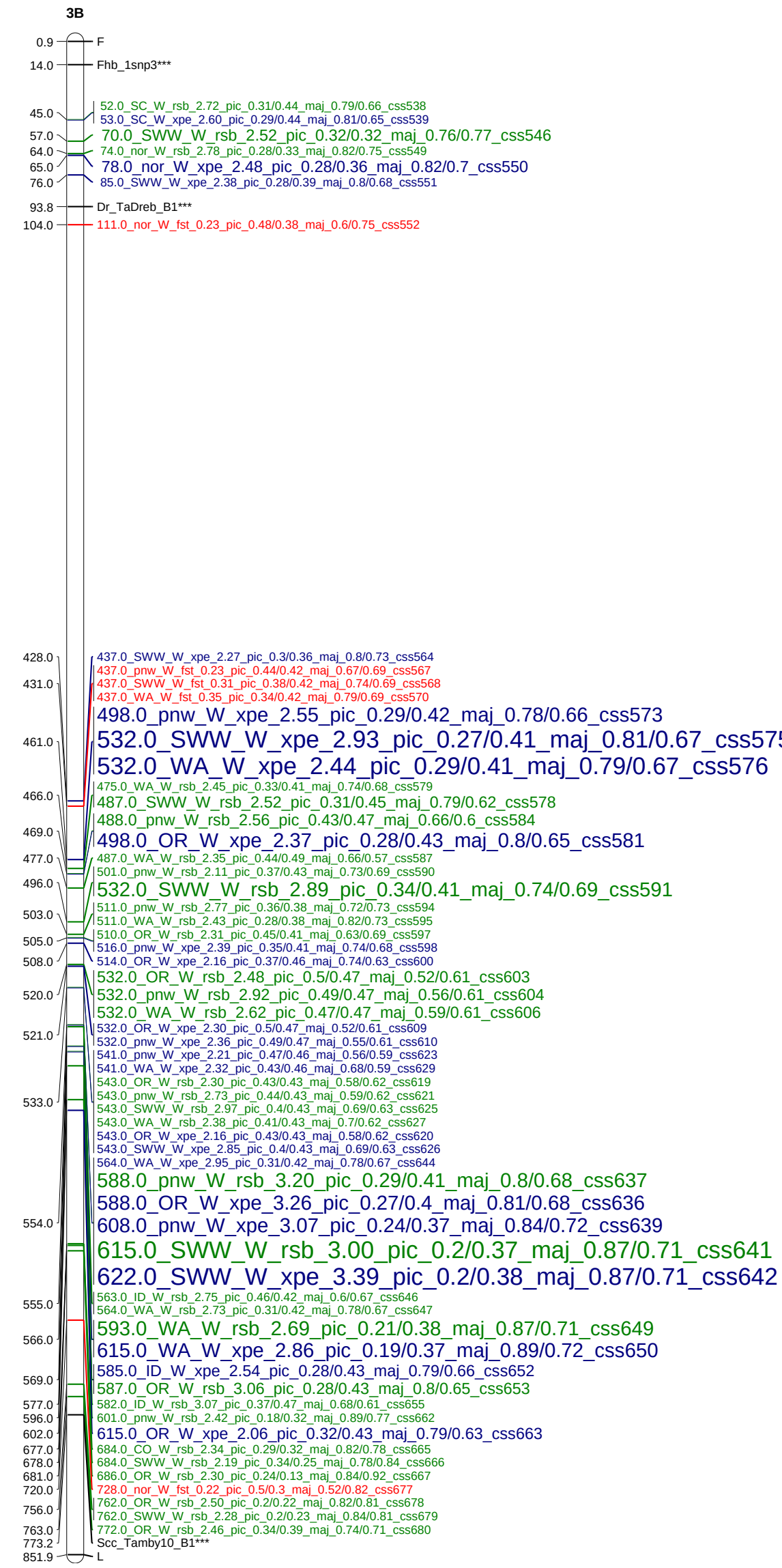
3B
0.9 F
14.0 Fhb_1snp3***
45.0 57.0 64.0 65.0 76.0
52.0_SC_W_rsb_2.72_pic_0.31/0.44_maj_0.79/0.66_css538
53.0_SC_W_xpe_2.60_pic_0.29/0.44_maj_0.81/0.65_css539
70.0_SWW_W_rsb_2.52_pic_0.32/0.32_maj_0.76/0.77_css546
74.0_nor_W_rsb_2.78_pic_0.28/0.33_maj_0.82/0.75_css549
78.0_nor_W_xpe_2.48_pic_0.28/0.36_maj_0.82/0.7_css550
85.0_SWW_W_xpe_2.38_pic_0.28/0.39_maj_0.8/0.68_css551
93.8 Dr_TaDreb_B1***
104.0 111.0_nor_W_fst_0.23_pic_0.48/0.38_maj_0.6/0.75_css552
428.0 431.0 461.0 466.0 469.0 477.0 496.0 503.0 505.0 508.0 520.0 521.0 533.0 554.0 555.0 566.0 569.0 577.0 596.0 602.0 677.0 678.0 681.0 720.0 756.0 763.0 773.2
437.0_SWW_W_xpe_2.27_pic_0.3/0.36_maj_0.8/0.73_css564
437.0_pnw_W_fst_0.23_pic_0.44/0.42_maj_0.67/0.69_css567
437.0_SWW_W_fst_0.31_pic_0.38/0.42_maj_0.74/0.69_css568
437.0_WA_W_fst_0.35_pic_0.34/0.42_maj_0.79/0.69_css570
498.0_pnw_W_xpe_2.55_pic_0.29/0.42_maj_0.78/0.66_css573
532.0_SWW_W_xpe_2.93_pic_0.27/0.41_maj_0.81/0.67_css575
532.0_WA_W_xpe_2.44_pic_0.29/0.41_maj_0.79/0.67_css576
475.0_WA_W_rsb_2.45_pic_0.33/0.41_maj_0.74/0.68_css579
487.0_SWW_W_rsb_2.52_pic_0.31/0.45_maj_0.79/0.62_css578
488.0_pnw_W_rsb_2.56_pic_0.43/0.47_maj_0.66/0.6_css584
498.0_OR_W_xpe_2.37_pic_0.28/0.43_maj_0.8/0.65_css581
487.0_WA_W_rsb_2.35_pic_0.44/0.49_maj_0.66/0.57_css587
501.0_pnw_W_rsb_2.11_pic_0.37/0.43_maj_0.73/0.69_css590
532.0_SWW_W_rsb_2.89_pic_0.34/0.41_maj_0.74/0.69_css591
511.0_pnw_W_rsb_2.77_pic_0.36/0.38_maj_0.72/0.73_css594
511.0_WA_W_rsb_2.43_pic_0.28/0.38_maj_0.82/0.73_css595
510.0_OR_W_rsb_2.31_pic_0.45/0.41_maj_0.63/0.69_css597
516.0_pnw_W_xpe_2.39_pic_0.35/0.41_maj_0.74/0.68_css598
514.0_OR_W_xpe_2.16_pic_0.37/0.46_maj_0.74/0.63_css600
532.0_OR_W_rsb_2.48_pic_0.5/0.47_maj_0.52/0.61_css603
532.0_pnw_W_rsb_2.92_pic_0.49/0.47_maj_0.56/0.61_css604
532.0_WA_W_rsb_2.62_pic_0.47/0.47_maj_0.59/0.61_css606
532.0_OR_W_xpe_2.30_pic_0.5/0.47_maj_0.52/0.61_css609
532.0_pnw_W_xpe_2.36_pic_0.49/0.47_maj_0.55/0.61_css610
541.0_pnw_W_xpe_2.21_pic_0.47/0.46_maj_0.56/0.59_css623
541.0_WA_W_xpe_2.32_pic_0.43/0.46_maj_0.68/0.59_css629
543.0_OR_W_rsb_2.30_pic_0.43/0.43_maj_0.58/0.62_css619
543.0_pnw_W_rsb_2.73_pic_0.44/0.43_maj_0.59/0.62_css621
543.0_SWW_W_rsb_2.97_pic_0.4/0.43_maj_0.69/0.63_css625
543.0_WA_W_rsb_2.38_pic_0.41/0.43_maj_0.7/0.62_css627
543.0_OR_W_xpe_2.16_pic_0.43/0.43_maj_0.58/0.62_css620
543.0_SWW_W_xpe_2.85_pic_0.4/0.43_maj_0.69/0.63_css626
564.0_WA_W_xpe_2.95_pic_0.31/0.42_maj_0.78/0.67_css644
588.0_pnw_W_rsb_3.20_pic_0.29/0.41_maj_0.8/0.68_css637
588.0_OR_W_xpe_3.26_pic_0.27/0.4_maj_0.81/0.68_css636
608.0_pnw_W_xpe_3.07_pic_0.24/0.37_maj_0.84/0.72_css639
615.0_SWW_W_rsb_3.00_pic_0.2/0.37_maj_0.87/0.71_css641
622.0_SWW_W_xpe_3.39_pic_0.2/0.38_maj_0.87/0.71_css642
563.0_ID_W_rsb_2.75_pic_0.46/0.42_maj_0.6/0.67_css646
564.0_WA_W_rsb_2.73_pic_0.31/0.42_maj_0.78/0.67_css647
593.0_WA_W_rsb_2.69_pic_0.21/0.38_maj_0.87/0.71_css649
615.0_WA_W_xpe_2.86_pic_0.19/0.37_maj_0.89/0.72_css650
585.0_ID_W_xpe_2.54_pic_0.28/0.43_maj_0.79/0.66_css652
587.0_OR_W_rsb_3.06_pic_0.28/0.43_maj_0.8/0.65_css653
582.0_ID_W_rsb_3.07_pic_0.37/0.47_maj_0.68/0.61_css655
601.0_pnw_W_rsb_2.42_pic_0.18/0.32_maj_0.89/0.77_css662
615.0_OR_W_xpe_2.06_pic_0.32/0.43_maj_0.79/0.63_css663
684.0_CO_W_rsb_2.34_pic_0.29/0.32_maj_0.82/0.78_css665
684.0_SWW_W_rsb_2.19_pic_0.34/0.25_maj_0.78/0.84_css666
686.0_OR_W_rsb_2.30_pic_0.24/0.13_maj_0.84/0.92_css667
728.0_nor_W_fst_0.22_pic_0.5/0.3_maj_0.52/0.82_css677
762.0_OR_W_rsb_2.50_pic_0.2/0.22_maj_0.82/0.81_css678
762.0_SWW_W_rsb_2.28_pic_0.2/0.23_maj_0.84/0.81_css679
772.0_OR_W_rsb_2.46_pic_0.34/0.39_maj_0.74/0.71_css680
Scc_Tamby10_B1***
851.9 L

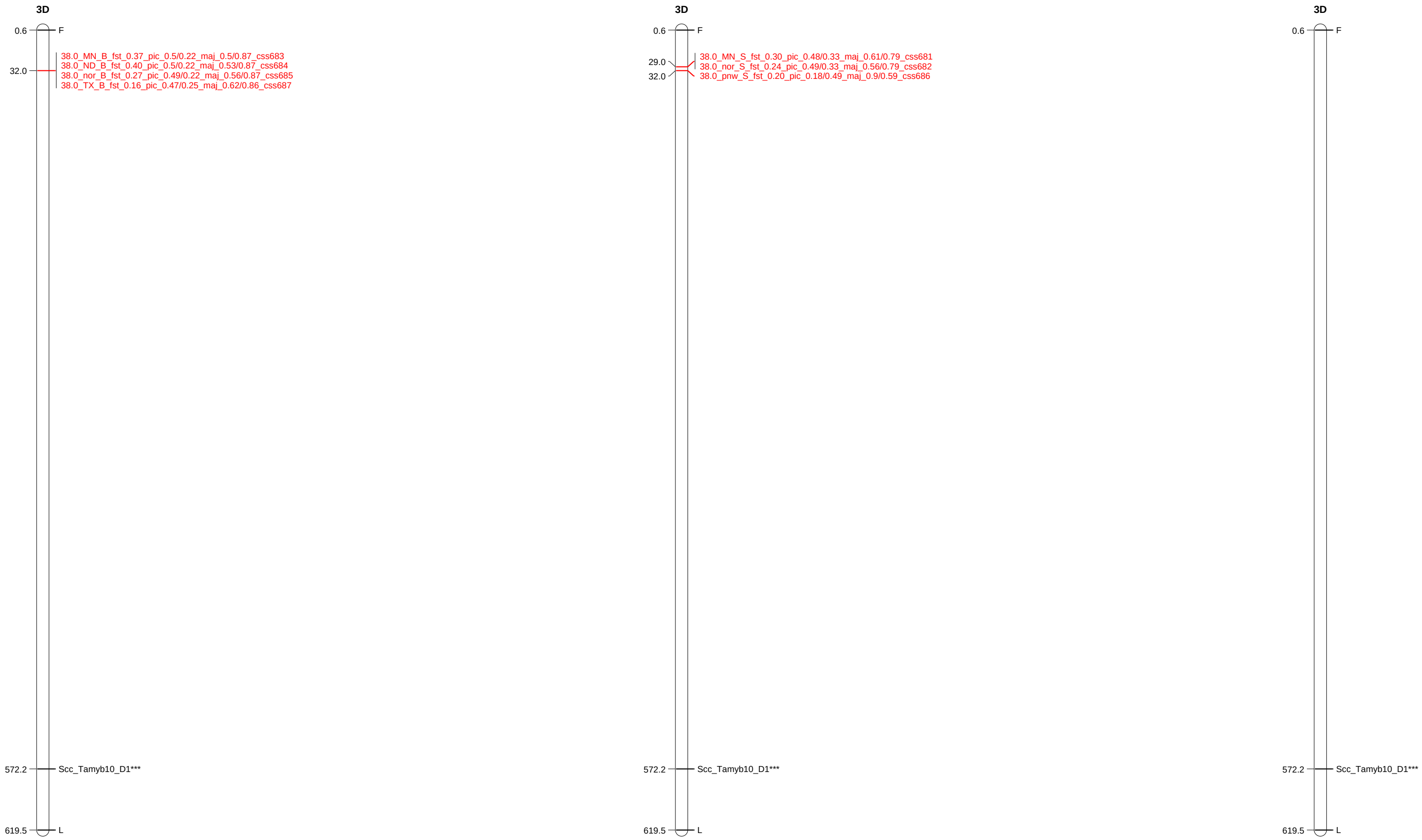
3D
0.6
F
32.0
38.0_MN_B_fst_0.37_pic_0.5/0.22_maj_0.5/0.87_css683
38.0_ND_B_fst_0.40_pic_0.5/0.22_maj_0.53/0.87_css684
38.0_nor_B_fst_0.27_pic_0.49/0.22_maj_0.56/0.87_css685
38.0_TX_B_fst_0.16_pic_0.47/0.25_maj_0.62/0.86_css687
572.2
Scc_Tamyb10_D1***
619.5
L
3D
0.6
F
29.0
32.0
38.0_MN_S_fst_0.30_pic_0.48/0.33_maj_0.61/0.79_css681
38.0_nor_S_fst_0.24_pic_0.49/0.33_maj_0.56/0.79_css682
38.0_pnw_S_fst_0.20_pic_0.18/0.49_maj_0.9/0.59_css686
572.2
Scc_Tamyb10_D1***
619.5
L
3D
0.6
F
572.2
Scc_Tamyb10_D1***
619.5
L

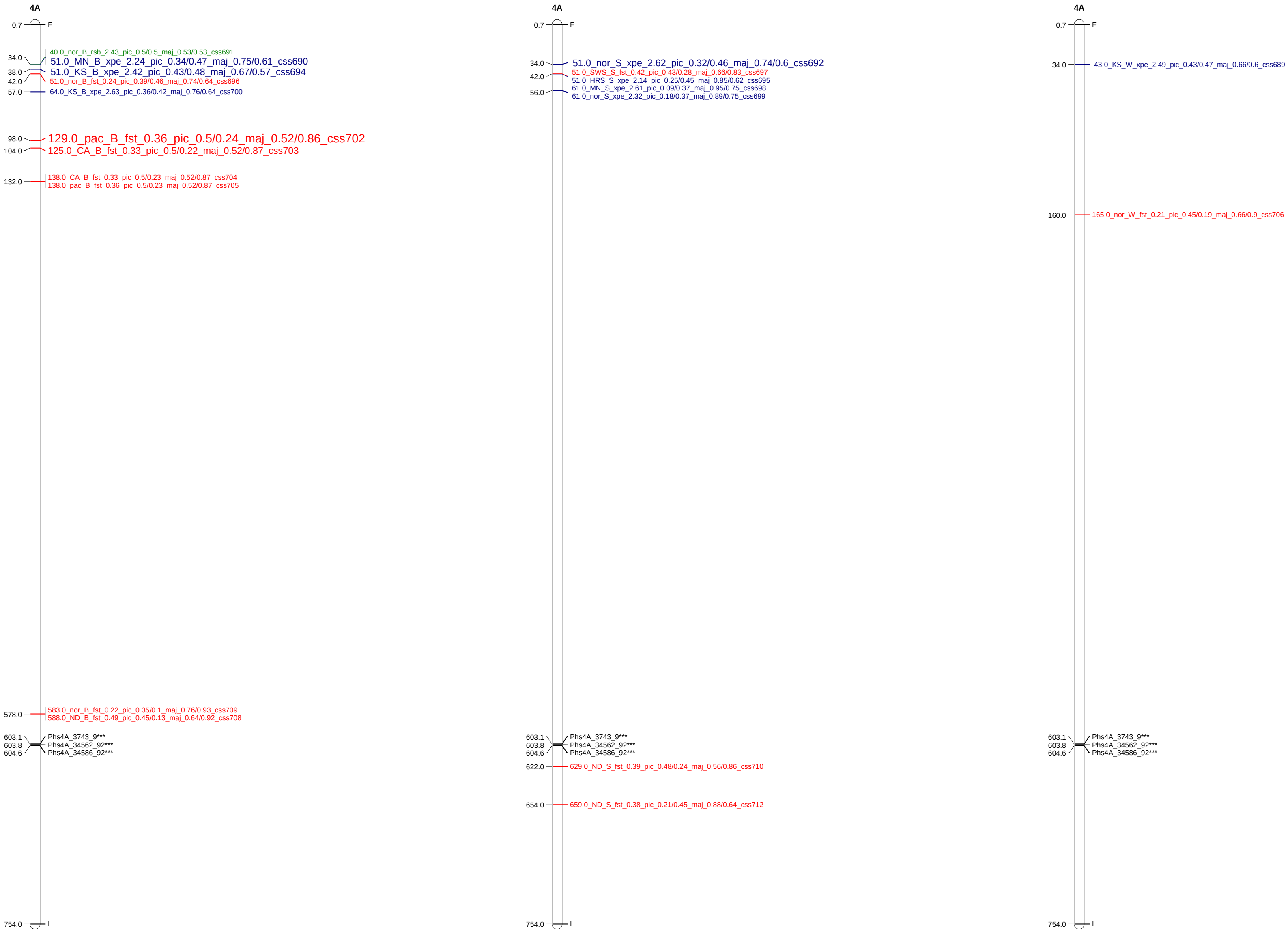
4A
0.7 F
40.0_nor_B_rsb_2.43_pic_0.5/0.5_maj_0.53/0.53_css691
34.0 51.0_MN_B_xpe_2.24_pic_0.34/0.47_maj_0.75/0.61_css690
38.0 51.0_KS_B_xpe_2.42_pic_0.43/0.48_maj_0.67/0.57_css694
42.0 51.0_nor_B_fst_0.24_pic_0.39/0.46_maj_0.74/0.64_css696
57.0 64.0_KS_B_xpe_2.63_pic_0.36/0.42_maj_0.76/0.64_css700
98.0 129.0_pac_B_fst_0.36_pic_0.5/0.24_maj_0.52/0.86_css702
104.0 125.0_CA_B_fst_0.33_pic_0.5/0.22_maj_0.52/0.87_css703
132.0 138.0_CA_B_fst_0.33_pic_0.5/0.23_maj_0.52/0.87_css704
138.0_pac_B_fst_0.36_pic_0.5/0.23_maj_0.52/0.87_css705
578.0 583.0_nor_B_fst_0.22_pic_0.35/0.1_maj_0.76/0.93_css709
588.0_ND_B_fst_0.49_pic_0.45/0.13_maj_0.64/0.92_css708
603.1 Phs4A_3743_9***
603.8 Phs4A_34562_92***
604.6 Phs4A_34586_92***
754.0 L
4A
0.7 F
34.0 51.0_nor_S_xpe_2.62_pic_0.32/0.46_maj_0.74/0.6_css692
42.0 51.0_SWS_S_fst_0.42_pic_0.43/0.28_maj_0.66/0.83_css697
51.0_HRS_S_xpe_2.14_pic_0.25/0.45_maj_0.85/0.62_css695
56.0 61.0_MN_S_xpe_2.61_pic_0.09/0.37_maj_0.95/0.75_css698
61.0_nor_S_xpe_2.32_pic_0.18/0.37_maj_0.89/0.75_css699
603.1 Phs4A_3743_9***
603.8 Phs4A_34562_92***
604.6 Phs4A_34586_92***
622.0 629.0_ND_S_fst_0.39_pic_0.48/0.24_maj_0.56/0.86_css710
654.0 659.0_ND_S_fst_0.38_pic_0.21/0.45_maj_0.88/0.64_css712
754.0 L
4A
0.7 F
34.0 43.0_KS_W_xpe_2.49_pic_0.43/0.47_maj_0.66/0.6_css689
160.0 165.0_nor_W_fst_0.21_pic_0.45/0.19_maj_0.66/0.9_css706
603.1 Phs4A_3743_9***
603.8 Phs4A_34562_92***
604.6 Phs4A_34586_92***
754.0 L

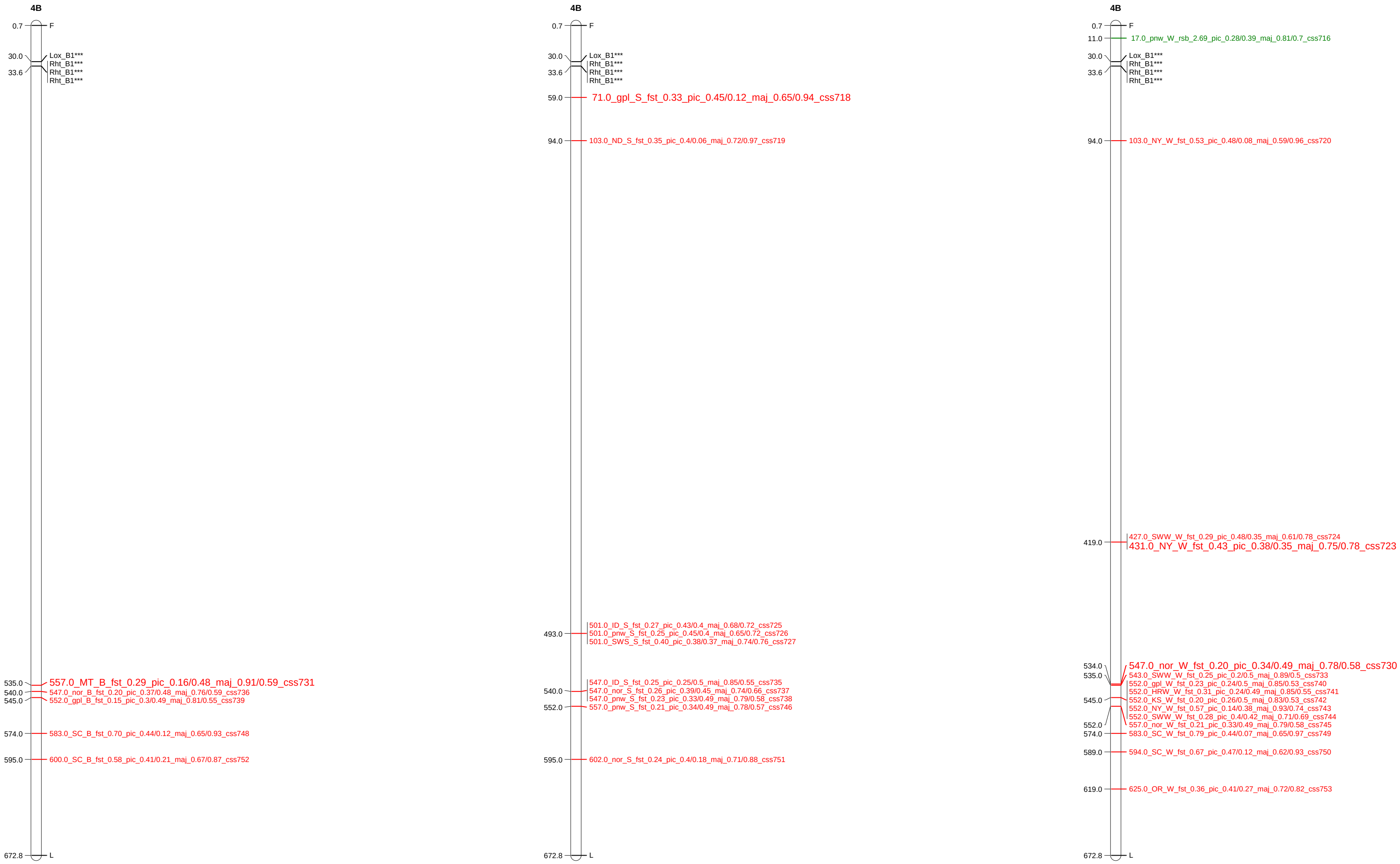
4B
0.7
F
30.0
33.6
Lox_B1***
Rht_B1***
Rht_B1***
Rht_B1***
535.0
540.0
545.0
557.0_MT_B_fst_0.29_pic_0.16/0.48_maj_0.91/0.59_css731
547.0_nor_B_fst_0.20_pic_0.37/0.48_maj_0.76/0.59_css736
552.0_gpl_B_fst_0.15_pic_0.3/0.49_maj_0.81/0.55_css739
574.0
583.0_SC_B_fst_0.70_pic_0.44/0.12_maj_0.65/0.93_css748
595.0
600.0_SC_B_fst_0.58_pic_0.41/0.21_maj_0.67/0.87_css752
672.8
L
4B
0.7
F
30.0
33.6
Lox_B1***
Rht_B1***
Rht_B1***
Rht_B1***
59.0
71.0_gpl_S_fst_0.33_pic_0.45/0.12_maj_0.65/0.94_css718
94.0
103.0_ND_S_fst_0.35_pic_0.4/0.06_maj_0.72/0.97_css719
493.0
501.0_ID_S_fst_0.27_pic_0.43/0.4_maj_0.68/0.72_css725
501.0_pnw_S_fst_0.25_pic_0.45/0.4_maj_0.65/0.72_css726
501.0_SWS_S_fst_0.40_pic_0.38/0.37_maj_0.74/0.76_css727
540.0
552.0
547.0_ID_S_fst_0.25_pic_0.25/0.5_maj_0.85/0.55_css735
547.0_nor_S_fst_0.26_pic_0.39/0.45_maj_0.74/0.66_css737
547.0_pnw_S_fst_0.23_pic_0.33/0.49_maj_0.79/0.58_css738
557.0_pnw_S_fst_0.21_pic_0.34/0.49_maj_0.78/0.57_css746
595.0
602.0_nor_S_fst_0.24_pic_0.4/0.18_maj_0.71/0.88_css751
672.8
L
4B
0.7
F
11.0
17.0_pnw_W_rsb_2.69_pic_0.28/0.39_maj_0.81/0.7_css716
30.0
33.6
Lox_B1***
Rht_B1***
Rht_B1***
Rht_B1***
94.0
103.0_NY_W_fst_0.53_pic_0.48/0.08_maj_0.59/0.96_css720
419.0
427.0_SWW_W_fst_0.29_pic_0.48/0.35_maj_0.61/0.78_css724
431.0_NY_W_fst_0.43_pic_0.38/0.35_maj_0.75/0.78_css723
534.0
535.0
545.0
552.0
547.0_nor_W_fst_0.20_pic_0.34/0.49_maj_0.78/0.58_css730
543.0_SWW_W_fst_0.25_pic_0.2/0.5_maj_0.89/0.5_css733
552.0_gpl_W_fst_0.23_pic_0.24/0.5_maj_0.85/0.53_css740
552.0_HRW_W_fst_0.31_pic_0.24/0.49_maj_0.85/0.55_css741
552.0_KS_W_fst_0.20_pic_0.26/0.5_maj_0.83/0.53_css742
552.0_NY_W_fst_0.57_pic_0.14/0.38_maj_0.93/0.74_css743
552.0_SWW_W_fst_0.28_pic_0.4/0.42_maj_0.71/0.69_css744
557.0_nor_W_fst_0.21_pic_0.33/0.49_maj_0.79/0.58_css745
574.0
583.0_SC_W_fst_0.79_pic_0.44/0.07_maj_0.65/0.97_css749
589.0
594.0_SC_W_fst_0.67_pic_0.47/0.12_maj_0.62/0.93_css750
619.0
625.0_OR_W_fst_0.36_pic_0.41/0.27_maj_0.72/0.82_css753
672.8
L

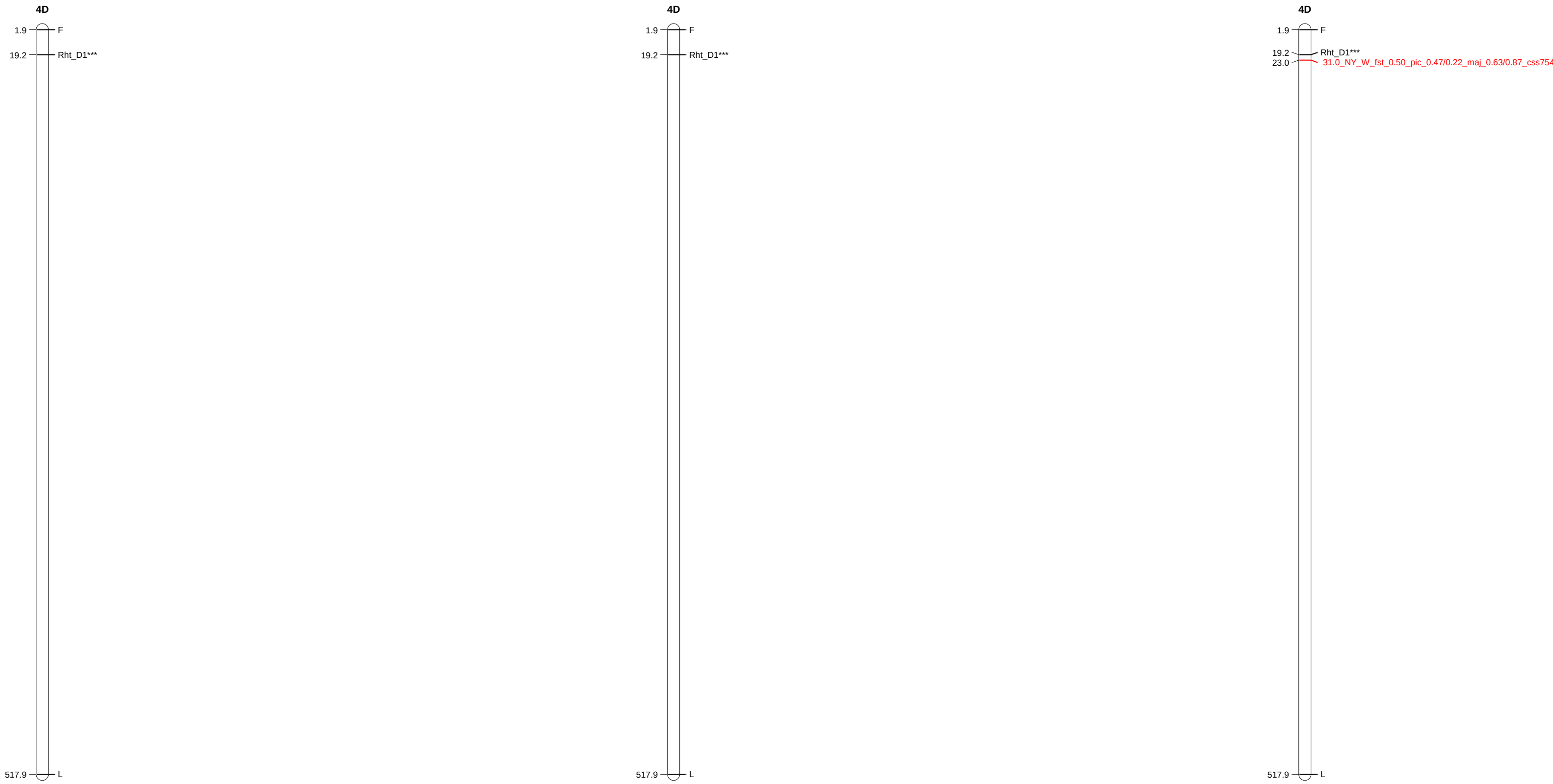
4D
1.9
F
19.2
Rht_D1***
517.9
L
4D
1.9
F
19.2
Rht_D1***
517.9
L
4D
1.9
F
19.2
Rht_D1***
23.0
31.0_NY_W_fst_0.50_pic_0.47/0.22_maj_0.63/0.87_css754
517.9
L

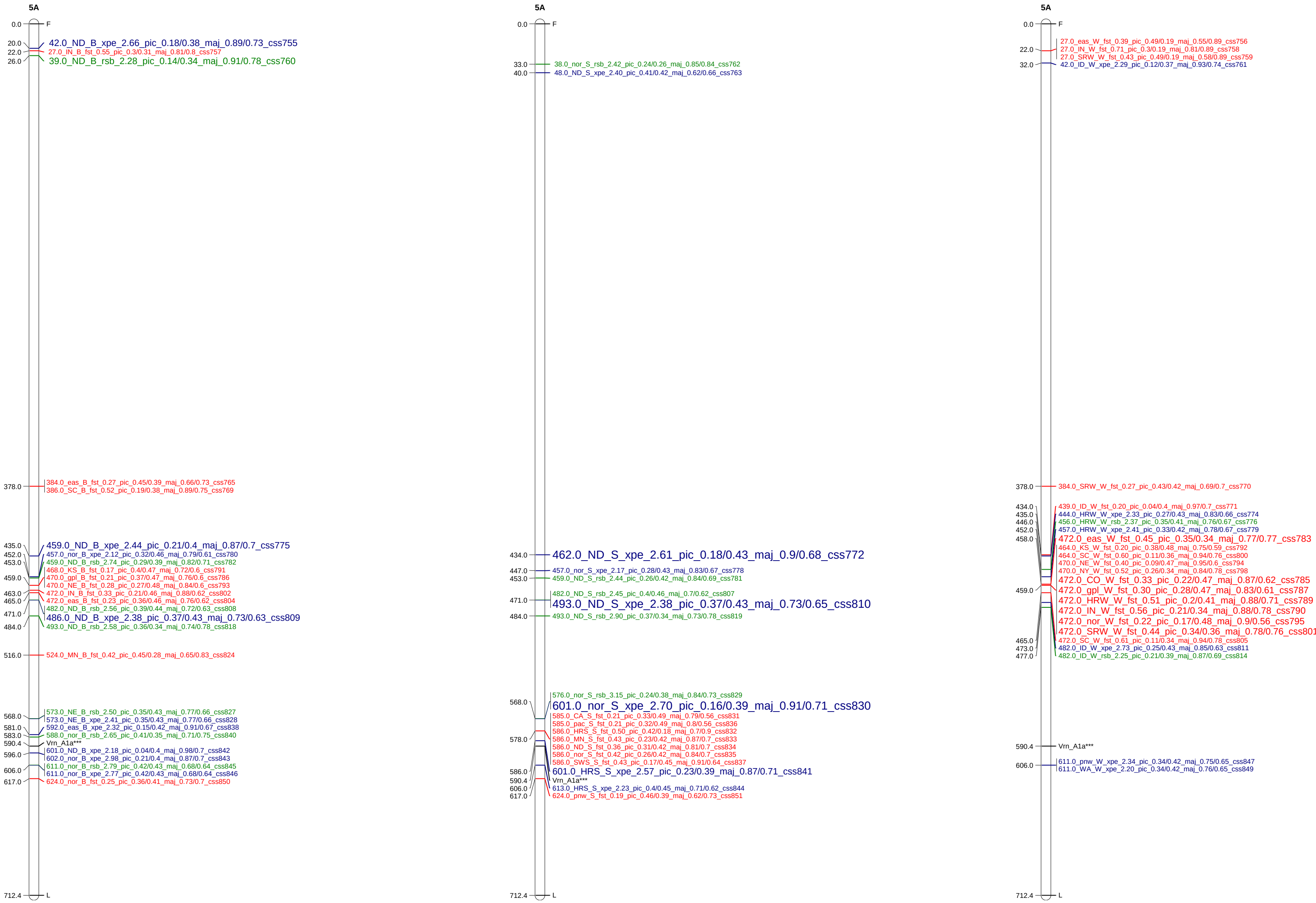

5A
0.0 F
20.0
22.0
26.0
42.0_ND_B_xpe_2.66_pic_0.18/0.38_maj_0.89/0.73_css755
27.0_IN_B_fst_0.55_pic_0.3/0.31_maj_0.81/0.8_css757
39.0_ND_B_rsb_2.28_pic_0.14/0.34_maj_0.91/0.78_css760
378.0
384.0_eas_B_fst_0.27_pic_0.45/0.39_maj_0.66/0.73_css765
386.0_SC_B_fst_0.52_pic_0.19/0.38_maj_0.89/0.75_css769
435.0
452.0
453.0
459.0
463.0
465.0
471.0
484.0
459.0_ND_B_xpe_2.44_pic_0.21/0.4_maj_0.87/0.7_css775
457.0_nor_B_xpe_2.12_pic_0.32/0.46_maj_0.79/0.61_css780
459.0_ND_B_rsb_2.74_pic_0.29/0.39_maj_0.82/0.71_css782
468.0_KS_B_fst_0.17_pic_0.4/0.47_maj_0.72/0.6_css791
470.0_gpl_B_fst_0.21_pic_0.37/0.47_maj_0.76/0.6_css786
470.0_NE_B_fst_0.28_pic_0.27/0.48_maj_0.84/0.6_css793
472.0_IN_B_fst_0.33_pic_0.21/0.46_maj_0.88/0.62_css802
472.0_eas_B_fst_0.23_pic_0.36/0.46_maj_0.76/0.62_css804
482.0_ND_B_rsb_2.56_pic_0.39/0.44_maj_0.72/0.63_css808
486.0_ND_B_xpe_2.38_pic_0.37/0.43_maj_0.73/0.63_css809
493.0_ND_B_rsb_2.58_pic_0.36/0.34_maj_0.74/0.78_css818
516.0
524.0_MN_B_fst_0.42_pic_0.45/0.28_maj_0.65/0.83_css824
568.0
581.0
583.0
590.4
596.0
606.0
617.0
573.0_NE_B_rsb_2.50_pic_0.35/0.43_maj_0.77/0.66_css827
573.0_NE_B_xpe_2.41_pic_0.35/0.43_maj_0.77/0.66_css828
592.0_eas_B_xpe_2.32_pic_0.15/0.42_maj_0.91/0.67_css838
588.0_nor_B_rsb_2.65_pic_0.41/0.35_maj_0.71/0.75_css840
Vrn_A1a***
601.0_ND_B_xpe_2.18_pic_0.04/0.4_maj_0.98/0.7_css842
602.0_nor_B_xpe_2.98_pic_0.21/0.4_maj_0.87/0.7_css843
611.0_nor_B_rsb_2.79_pic_0.42/0.43_maj_0.68/0.64_css845
611.0_nor_B_xpe_2.77_pic_0.42/0.43_maj_0.68/0.64_css846
624.0_nor_B_fst_0.25_pic_0.36/0.41_maj_0.73/0.7_css850
712.4 L
5A
0.0 F
33.0
40.0
38.0_nor_S_rsb_2.42_pic_0.24/0.26_maj_0.85/0.84_css762
48.0_ND_S_xpe_2.40_pic_0.41/0.42_maj_0.62/0.66_css763
434.0
447.0
453.0
462.0_ND_S_xpe_2.61_pic_0.18/0.43_maj_0.9/0.68_css772
457.0_nor_S_xpe_2.17_pic_0.28/0.43_maj_0.83/0.67_css778
459.0_ND_S_rsb_2.44_pic_0.26/0.42_maj_0.84/0.69_css781
471.0
484.0
482.0_ND_S_rsb_2.45_pic_0.4/0.46_maj_0.7/0.62_css807
493.0_ND_S_xpe_2.38_pic_0.37/0.43_maj_0.73/0.65_css810
493.0_ND_S_rsb_2.90_pic_0.37/0.34_maj_0.73/0.78_css819
568.0
578.0
586.0
590.4
606.0
617.0
576.0_nor_S_rsb_3.15_pic_0.24/0.38_maj_0.84/0.73_css829
601.0_nor_S_xpe_2.70_pic_0.16/0.39_maj_0.91/0.71_css830
585.0_CA_S_fst_0.21_pic_0.33/0.49_maj_0.79/0.56_css831
585.0_pac_S_fst_0.21_pic_0.32/0.49_maj_0.8/0.56_css836
586.0_HRS_S_fst_0.50_pic_0.42/0.18_maj_0.7/0.9_css832
586.0_MN_S_fst_0.43_pic_0.23/0.42_maj_0.87/0.7_css833
586.0_ND_S_fst_0.36_pic_0.31/0.42_maj_0.81/0.7_css834
586.0_nor_S_fst_0.42_pic_0.26/0.42_maj_0.84/0.7_css835
586.0_SWS_S_fst_0.43_pic_0.17/0.45_maj_0.91/0.64_css837
601.0_HRS_S_xpe_2.57_pic_0.23/0.39_maj_0.87/0.71_css841
Vrn_A1a***
613.0_HRS_S_xpe_2.23_pic_0.4/0.45_maj_0.71/0.62_css844
624.0_pnw_S_fst_0.19_pic_0.46/0.39_maj_0.62/0.73_css851
712.4 L
5A
0.0 F
22.0
32.0
27.0_eas_W_fst_0.39_pic_0.49/0.19_maj_0.55/0.89_css756
27.0_IN_W_fst_0.71_pic_0.3/0.19_maj_0.81/0.89_css758
27.0_SRW_W_fst_0.43_pic_0.49/0.19_maj_0.58/0.89_css759
42.0_ID_W_xpe_2.29_pic_0.12/0.37_maj_0.93/0.74_css761
378.0
384.0_SRW_W_fst_0.27_pic_0.43/0.42_maj_0.69/0.7_css770
434.0
435.0
446.0
452.0
458.0
459.0
465.0
473.0
477.0
439.0_ID_W_fst_0.20_pic_0.04/0.4_maj_0.97/0.7_css771
444.0_HRW_W_xpe_2.33_pic_0.27/0.43_maj_0.83/0.66_css774
456.0_HRW_W_rsb_2.37_pic_0.35/0.41_maj_0.76/0.67_css776
457.0_HRW_W_xpe_2.41_pic_0.33/0.42_maj_0.78/0.67_css779
472.0_eas_W_fst_0.45_pic_0.35/0.34_maj_0.77/0.77_css783
464.0_KS_W_fst_0.20_pic_0.38/0.48_maj_0.75/0.59_css792
464.0_SC_W_fst_0.60_pic_0.11/0.36_maj_0.94/0.76_css800
470.0_NE_W_fst_0.40_pic_0.09/0.47_maj_0.95/0.6_css794
470.0_NY_W_fst_0.52_pic_0.26/0.34_maj_0.84/0.78_css798
472.0_CO_W_fst_0.33_pic_0.22/0.47_maj_0.87/0.62_css785
472.0_gpl_W_fst_0.30_pic_0.28/0.47_maj_0.83/0.61_css787
472.0_HRW_W_fst_0.51_pic_0.2/0.41_maj_0.88/0.71_css789
472.0_IN_W_fst_0.56_pic_0.21/0.34_maj_0.88/0.78_css790
472.0_nor_W_fst_0.22_pic_0.17/0.48_maj_0.9/0.56_css795
472.0_SRW_W_fst_0.44_pic_0.34/0.36_maj_0.78/0.76_css801
472.0_SC_W_fst_0.61_pic_0.11/0.34_maj_0.94/0.78_css805
482.0_ID_W_xpe_2.73_pic_0.25/0.43_maj_0.85/0.63_css811
482.0_ID_W_rsb_2.25_pic_0.21/0.39_maj_0.87/0.69_css814
590.4
Vrn_A1a***
606.0
611.0_pnw_W_xpe_2.34_pic_0.34/0.42_maj_0.75/0.65_css847
611.0_WA_W_xpe_2.20_pic_0.34/0.42_maj_0.76/0.65_css849
712.4 L

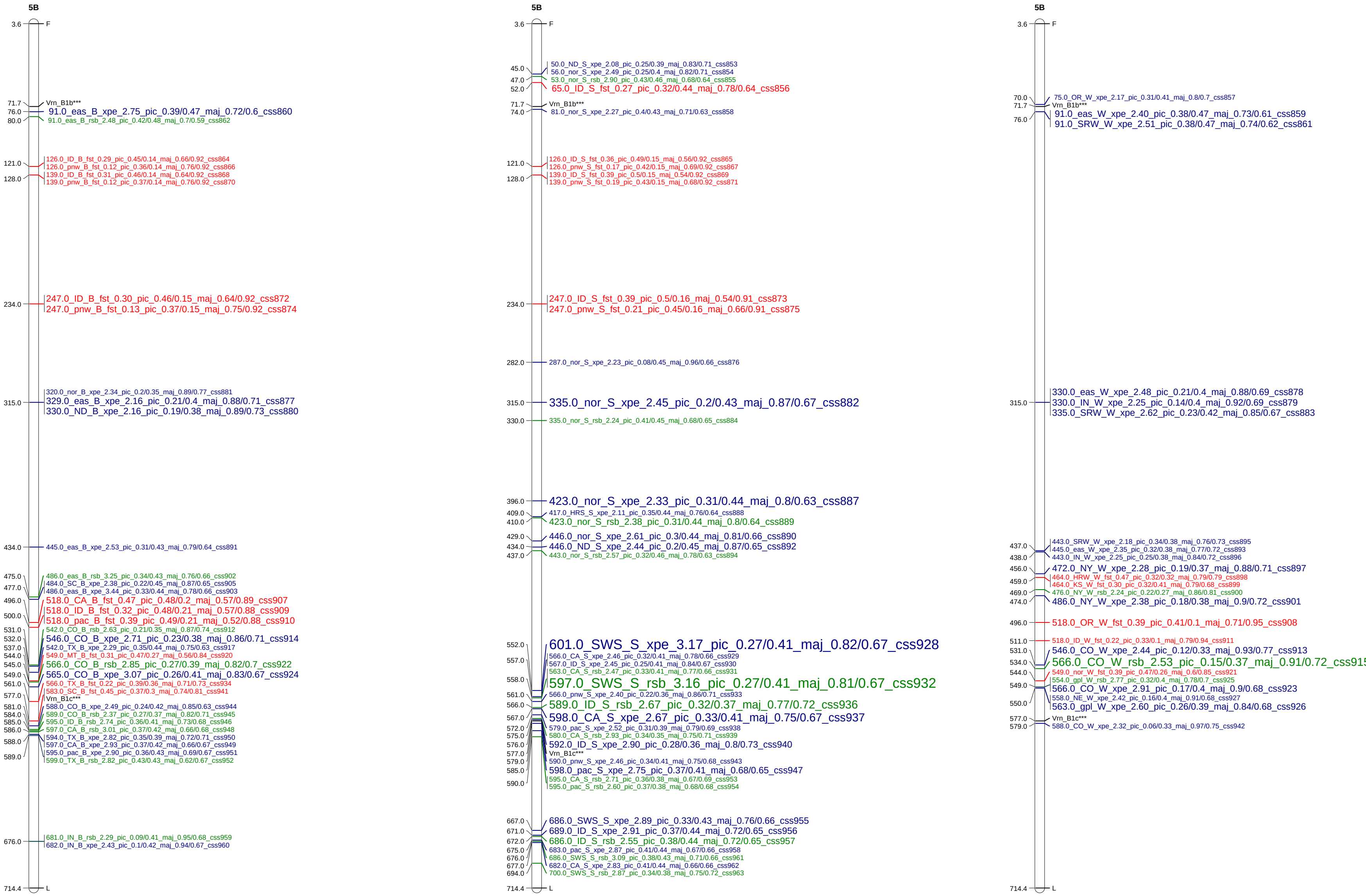

5B
3.6 F
71.7 Vrn_B1b***
76.0 91.0_eas_B_xpe_2.75_pic_0.39/0.47_maj_0.72/0.6_css860
80.0 91.0_eas_B_rsb_2.48_pic_0.42/0.48_maj_0.7/0.59_css862
121.0 126.0_ID_B_fst_0.29_pic_0.45/0.14_maj_0.66/0.92_css864
126.0_pnw_B_fst_0.12_pic_0.36/0.14_maj_0.76/0.92_css866
128.0 139.0_ID_B_fst_0.31_pic_0.46/0.14_maj_0.64/0.92_css868
139.0_pnw_B_fst_0.12_pic_0.37/0.14_maj_0.76/0.92_css870
234.0 247.0_ID_B_fst_0.30_pic_0.46/0.15_maj_0.64/0.92_css872
247.0_pnw_B_fst_0.13_pic_0.37/0.15_maj_0.75/0.92_css874
315.0 320.0_nor_B_xpe_2.34_pic_0.2/0.35_maj_0.89/0.77_css881
329.0_eas_B_xpe_2.16_pic_0.21/0.4_maj_0.88/0.71_css877
330.0_ND_B_xpe_2.16_pic_0.19/0.38_maj_0.89/0.73_css880
434.0 445.0_eas_B_xpe_2.53_pic_0.31/0.43_maj_0.79/0.64_css891
475.0 486.0_eas_B_rsb_3.25_pic_0.34/0.43_maj_0.76/0.66_css902
477.0 484.0_SC_B_xpe_2.38_pic_0.22/0.45_maj_0.87/0.65_css905
486.0_eas_B_xpe_3.44_pic_0.33/0.44_maj_0.78/0.66_css903
496.0 518.0_CA_B_fst_0.47_pic_0.48/0.2_maj_0.57/0.89_css907
500.0 518.0_ID_B_fst_0.32_pic_0.48/0.21_maj_0.57/0.88_css909
518.0_pac_B_fst_0.39_pic_0.49/0.21_maj_0.52/0.88_css910
531.0 542.0_CO_B_rsb_2.63_pic_0.21/0.35_maj_0.87/0.74_css912
532.0 546.0_CO_B_xpe_2.71_pic_0.23/0.38_maj_0.86/0.71_css914
537.0 542.0_TX_B_xpe_2.29_pic_0.35/0.44_maj_0.75/0.63_css917
544.0 549.0_MT_B_fst_0.31_pic_0.47/0.27_maj_0.56/0.84_css920
545.0 566.0_CO_B_rsb_2.85_pic_0.27/0.39_maj_0.82/0.7_css922
549.0 565.0_CO_B_xpe_3.07_pic_0.26/0.41_maj_0.83/0.67_css924
561.0 566.0_TX_B_fst_0.22_pic_0.39/0.36_maj_0.71/0.73_css934
577.0 583.0_SC_B_fst_0.45_pic_0.37/0.3_maj_0.74/0.81_css941
Vrn_B1c***
581.0 588.0_CO_B_xpe_2.49_pic_0.24/0.42_maj_0.85/0.63_css944
584.0 589.0_CO_B_rsb_2.37_pic_0.27/0.37_maj_0.82/0.71_css945
585.0 595.0_ID_B_rsb_2.74_pic_0.36/0.41_maj_0.73/0.68_css946
586.0 597.0_CA_B_rsb_3.01_pic_0.37/0.42_maj_0.66/0.68_css948
588.0 597.0_CA_B_xpe_2.93_pic_0.37/0.42_maj_0.66/0.67_css949
594.0_TX_B_xpe_2.82_pic_0.35/0.39_maj_0.72/0.71_css950
589.0 595.0_pac_B_xpe_2.90_pic_0.36/0.43_maj_0.69/0.67_css951
599.0_TX_B_rsb_2.82_pic_0.43/0.43_maj_0.62/0.67_css952
676.0 681.0_IN_B_rsb_2.29_pic_0.09/0.41_maj_0.95/0.68_css959
682.0_IN_B_xpe_2.43_pic_0.1/0.42_maj_0.94/0.67_css960
714.4 L
5B
3.6 F
45.0 50.0_ND_S_xpe_2.08_pic_0.25/0.39_maj_0.83/0.71_css853
56.0_nor_S_xpe_2.49_pic_0.25/0.4_maj_0.82/0.71_css854
47.0 53.0_nor_S_rsb_2.90_pic_0.43/0.46_maj_0.68/0.64_css855
52.0 65.0_ID_S_fst_0.27_pic_0.32/0.44_maj_0.78/0.64_css856
71.7 Vrn_B1b***
74.0 81.0_nor_S_xpe_2.27_pic_0.4/0.43_maj_0.71/0.63_css858
121.0 126.0_ID_S_fst_0.36_pic_0.49/0.15_maj_0.56/0.92_css865
126.0_pnw_S_fst_0.17_pic_0.42/0.15_maj_0.69/0.92_css867
128.0 139.0_ID_S_fst_0.39_pic_0.5/0.15_maj_0.54/0.92_css869
139.0_pnw_S_fst_0.19_pic_0.43/0.15_maj_0.68/0.92_css871
234.0 247.0_ID_S_fst_0.39_pic_0.5/0.16_maj_0.54/0.91_css873
247.0_pnw_S_fst_0.21_pic_0.45/0.16_maj_0.66/0.91_css875
282.0 287.0_nor_S_xpe_2.23_pic_0.08/0.45_maj_0.96/0.66_css876
315.0 335.0_nor_S_xpe_2.45_pic_0.2/0.43_maj_0.87/0.67_css882
330.0 335.0_nor_S_rsb_2.24_pic_0.41/0.45_maj_0.68/0.65_css884
396.0 423.0_nor_S_xpe_2.33_pic_0.31/0.44_maj_0.8/0.63_css887
409.0 417.0_HRS_S_xpe_2.11_pic_0.35/0.44_maj_0.76/0.64_css888
410.0 423.0_nor_S_rsb_2.38_pic_0.31/0.44_maj_0.8/0.64_css889
429.0 446.0_nor_S_xpe_2.61_pic_0.3/0.44_maj_0.81/0.66_css890
434.0 446.0_ND_S_xpe_2.44_pic_0.2/0.45_maj_0.87/0.65_css892
437.0 443.0_nor_S_rsb_2.57_pic_0.32/0.46_maj_0.78/0.63_css894
552.0 601.0_SWS_S_xpe_3.17_pic_0.27/0.41_maj_0.82/0.67_css928
557.0 566.0_CA_S_xpe_2.46_pic_0.32/0.41_maj_0.78/0.66_css929
567.0_ID_S_xpe_2.45_pic_0.25/0.41_maj_0.84/0.67_css930
558.0 563.0_CA_S_rsb_2.47_pic_0.33/0.41_maj_0.77/0.66_css931
597.0_SWS_S_rsb_3.16_pic_0.27/0.41_maj_0.81/0.67_css932
561.0 566.0_pnw_S_xpe_2.40_pic_0.22/0.36_maj_0.86/0.71_css933
566.0 589.0_ID_S_rsb_2.67_pic_0.32/0.37_maj_0.77/0.72_css936
567.0 598.0_CA_S_xpe_2.67_pic_0.33/0.41_maj_0.75/0.67_css937
572.0 579.0_pac_S_xpe_2.52_pic_0.31/0.39_maj_0.79/0.69_css938
575.0 580.0_CA_S_rsb_2.93_pic_0.34/0.35_maj_0.75/0.71_css939
576.0 592.0_ID_S_xpe_2.90_pic_0.28/0.36_maj_0.8/0.73_css940
577.0 Vrn_B1c***
579.0 590.0_pnw_S_xpe_2.46_pic_0.34/0.41_maj_0.75/0.68_css943
585.0 598.0_pac_S_xpe_2.75_pic_0.37/0.41_maj_0.68/0.65_css947
590.0 595.0_CA_S_rsb_2.71_pic_0.36/0.38_maj_0.67/0.69_css953
595.0_pac_S_rsb_2.60_pic_0.37/0.38_maj_0.68/0.68_css954
667.0 686.0_SWS_S_xpe_2.89_pic_0.33/0.43_maj_0.76/0.66_css955
671.0 689.0_ID_S_xpe_2.91_pic_0.37/0.44_maj_0.72/0.65_css956
672.0 686.0_ID_S_rsb_2.55_pic_0.38/0.44_maj_0.72/0.65_css957
675.0 683.0_pac_S_xpe_2.87_pic_0.41/0.44_maj_0.67/0.66_css958
676.0 686.0_SWS_S_rsb_3.09_pic_0.38/0.43_maj_0.71/0.66_css961
677.0 682.0_CA_S_xpe_2.83_pic_0.41/0.44_maj_0.66/0.66_css962
694.0 700.0_SWS_S_rsb_2.87_pic_0.34/0.38_maj_0.75/0.72_css963
714.4 L
5B
3.6 F
70.0 75.0_OR_W_xpe_2.17_pic_0.31/0.41_maj_0.8/0.7_css857
71.7 Vrn_B1b***
76.0 91.0_eas_W_xpe_2.40_pic_0.38/0.47_maj_0.73/0.61_css859
91.0_SRW_W_xpe_2.51_pic_0.38/0.47_maj_0.74/0.62_css861
315.0 330.0_eas_W_xpe_2.48_pic_0.21/0.4_maj_0.88/0.69_css878
330.0_IN_W_xpe_2.25_pic_0.14/0.4_maj_0.92/0.69_css879
335.0_SRW_W_xpe_2.62_pic_0.23/0.42_maj_0.85/0.67_css883
437.0 443.0_SRW_W_xpe_2.18_pic_0.34/0.38_maj_0.76/0.73_css895
445.0_eas_W_xpe_2.35_pic_0.32/0.38_maj_0.77/0.72_css893
438.0 443.0_IN_W_xpe_2.25_pic_0.25/0.38_maj_0.84/0.72_css896
456.0 472.0_NY_W_xpe_2.28_pic_0.19/0.37_maj_0.88/0.71_css897
459.0 464.0_HRW_W_fst_0.47_pic_0.32/0.32_maj_0.79/0.79_css898
464.0_KS_W_fst_0.30_pic_0.32/0.41_maj_0.79/0.68_css899
469.0 476.0_NY_W_rsb_2.24_pic_0.22/0.27_maj_0.86/0.81_css900
474.0 486.0_NY_W_xpe_2.38_pic_0.18/0.38_maj_0.9/0.72_css901
496.0 518.0_OR_W_fst_0.39_pic_0.41/0.1_maj_0.71/0.95_css908
511.0 518.0_ID_W_fst_0.22_pic_0.33/0.1_maj_0.79/0.94_css911
531.0 546.0_CO_W_xpe_2.44_pic_0.12/0.33_maj_0.93/0.77_css913
534.0 566.0_CO_W_rsb_2.53_pic_0.15/0.37_maj_0.91/0.72_css915
544.0 549.0_nor_W_fst_0.39_pic_0.47/0.26_maj_0.6/0.85_css921
554.0_gpl_W_rsb_2.77_pic_0.32/0.4_maj_0.78/0.7_css925
549.0 566.0_CO_W_xpe_2.91_pic_0.17/0.4_maj_0.9/0.68_css923
558.0_NE_W_xpe_2.42_pic_0.16/0.4_maj_0.91/0.68_css927
550.0 563.0_gpl_W_xpe_2.60_pic_0.26/0.39_maj_0.84/0.68_css926
577.0 Vrn_B1c***
579.0 588.0_CO_W_xpe_2.32_pic_0.06/0.33_maj_0.97/0.75_css942
714.4 L

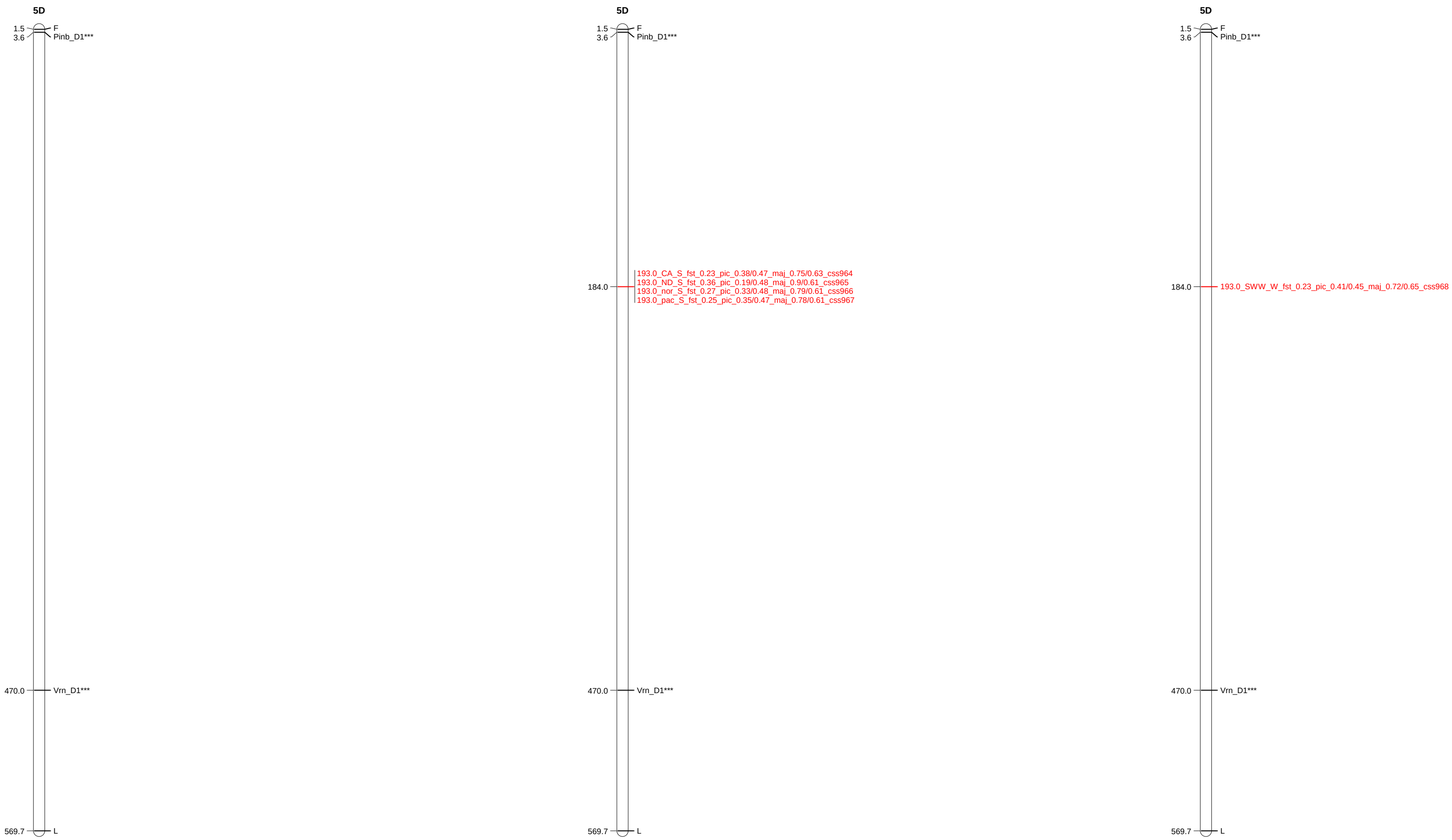
5D
1.5
3.6
F
Pinb_D1***
470.0
Vrn_D1***
569.7
L
5D
1.5
3.6
F
Pinb_D1***
184.0
193.0_CA_S_fst_0.23_pic_0.38/0.47_maj_0.75/0.63_css964
193.0_ND_S_fst_0.36_pic_0.19/0.48_maj_0.9/0.61_css965
193.0_nor_S_fst_0.27_pic_0.33/0.48_maj_0.79/0.61_css966
193.0_pac_S_fst_0.25_pic_0.35/0.47_maj_0.78/0.61_css967
470.0
Vrn_D1***
569.7
L
5D
1.5
3.6
F
Pinb_D1***
184.0
193.0_SWW_W_fst_0.23_pic_0.41/0.45_maj_0.72/0.65_css968
470.0
Vrn_D1***
569.7
L

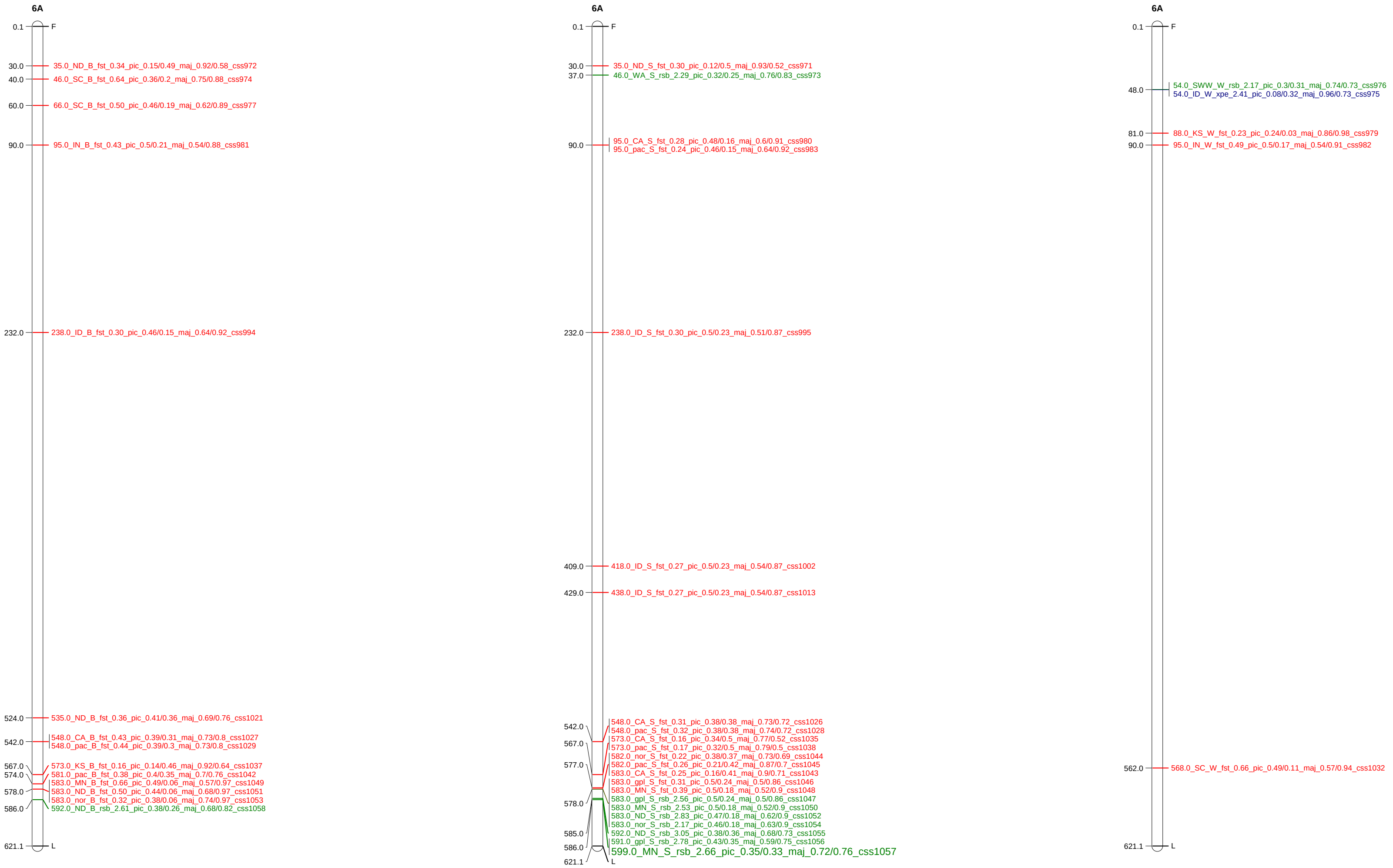
6A
0.1
F
30.0
35.0_ND_B_fst_0.34_pic_0.15/0.49_maj_0.92/0.58_css972
40.0
46.0_SC_B_fst_0.64_pic_0.36/0.2_maj_0.75/0.88_css974
60.0
66.0_SC_B_fst_0.50_pic_0.46/0.19_maj_0.62/0.89_css977
90.0
95.0_IN_B_fst_0.43_pic_0.5/0.21_maj_0.54/0.88_css981
232.0
238.0_ID_B_fst_0.30_pic_0.46/0.15_maj_0.64/0.92_css994
524.0
535.0_ND_B_fst_0.36_pic_0.41/0.36_maj_0.69/0.76_css1021
542.0
548.0_CA_B_fst_0.43_pic_0.39/0.31_maj_0.73/0.8_css1027
548.0_pac_B_fst_0.44_pic_0.39/0.3_maj_0.73/0.8_css1029
567.0
574.0
578.0
586.0
573.0_KS_B_fst_0.16_pic_0.14/0.46_maj_0.92/0.64_css1037
581.0_pac_B_fst_0.38_pic_0.4/0.35_maj_0.7/0.76_css1042
583.0_MN_B_fst_0.66_pic_0.49/0.06_maj_0.57/0.97_css1049
583.0_ND_B_fst_0.50_pic_0.44/0.06_maj_0.68/0.97_css1051
583.0_nor_B_fst_0.32_pic_0.38/0.06_maj_0.74/0.97_css1053
592.0_ND_B_rsb_2.61_pic_0.38/0.26_maj_0.68/0.82_css1058
621.1
L
6A
0.1
F
30.0
37.0
35.0_ND_S_fst_0.30_pic_0.12/0.5_maj_0.93/0.52_css971
46.0_WA_S_rsb_2.29_pic_0.32/0.25_maj_0.76/0.83_css973
90.0
95.0_CA_S_fst_0.28_pic_0.48/0.16_maj_0.6/0.91_css980
95.0_pac_S_fst_0.24_pic_0.46/0.15_maj_0.64/0.92_css983
232.0
238.0_ID_S_fst_0.30_pic_0.5/0.23_maj_0.51/0.87_css995
409.0
418.0_ID_S_fst_0.27_pic_0.5/0.23_maj_0.54/0.87_css1002
429.0
438.0_ID_S_fst_0.27_pic_0.5/0.23_maj_0.54/0.87_css1013
542.0
567.0
577.0
578.0
585.0
586.0
621.1
548.0_CA_S_fst_0.31_pic_0.38/0.38_maj_0.73/0.72_css1026
548.0_pac_S_fst_0.32_pic_0.38/0.38_maj_0.74/0.72_css1028
573.0_CA_S_fst_0.16_pic_0.34/0.5_maj_0.77/0.52_css1035
573.0_pac_S_fst_0.17_pic_0.32/0.5_maj_0.79/0.5_css1038
582.0_nor_S_fst_0.22_pic_0.38/0.37_maj_0.73/0.69_css1044
582.0_pac_S_fst_0.26_pic_0.21/0.42_maj_0.87/0.7_css1045
583.0_CA_S_fst_0.25_pic_0.16/0.41_maj_0.9/0.71_css1043
583.0_gpl_S_fst_0.31_pic_0.5/0.24_maj_0.5/0.86_css1046
583.0_MN_S_fst_0.39_pic_0.5/0.18_maj_0.52/0.9_css1048
583.0_gpl_S_rsb_2.56_pic_0.5/0.24_maj_0.5/0.86_css1047
583.0_MN_S_rsb_2.53_pic_0.5/0.18_maj_0.52/0.9_css1050
583.0_ND_S_rsb_2.83_pic_0.47/0.18_maj_0.62/0.9_css1052
583.0_nor_S_rsb_2.17_pic_0.46/0.18_maj_0.63/0.9_css1054
592.0_ND_S_rsb_3.05_pic_0.38/0.36_maj_0.68/0.73_css1055
591.0_gpl_S_rsb_2.78_pic_0.43/0.35_maj_0.59/0.75_css1056
599.0_MN_S_rsb_2.66_pic_0.35/0.33_maj_0.72/0.76_css1057
L
6A
0.1
F
48.0
54.0_SWW_W_rsb_2.17_pic_0.3/0.31_maj_0.74/0.73_css976
54.0_ID_W_xpe_2.41_pic_0.08/0.32_maj_0.96/0.73_css975
81.0
88.0_KS_W_fst_0.23_pic_0.24/0.03_maj_0.86/0.98_css979
90.0
95.0_IN_W_fst_0.49_pic_0.5/0.17_maj_0.54/0.91_css982
562.0
568.0_SC_W_fst_0.66_pic_0.49/0.11_maj_0.57/0.94_css1032
621.1
L

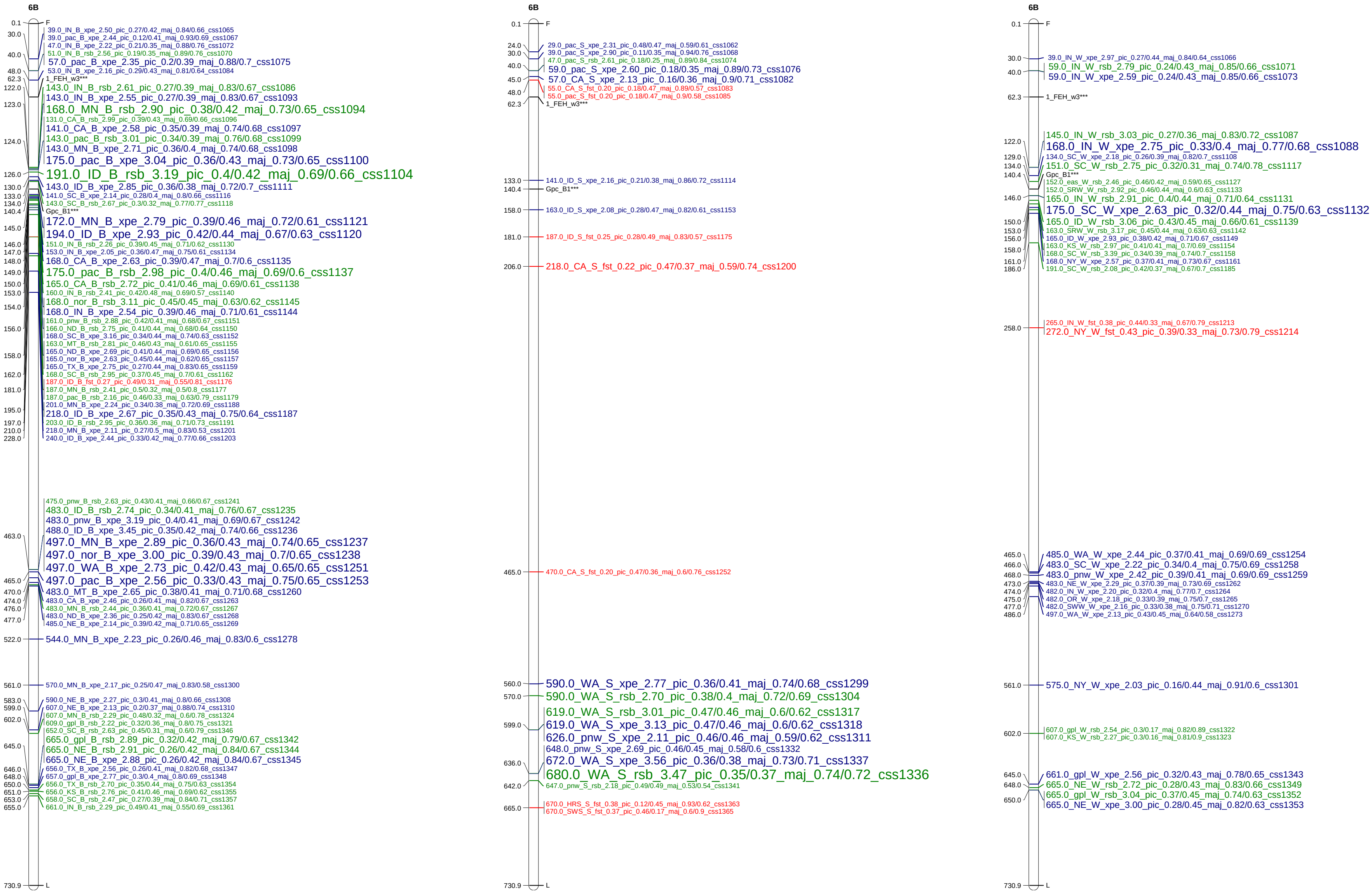
6B
F
39.0_IN_B_xpe_2.50_pic_0.27/0.42_maj_0.84/0.66_css1065
39.0_pac_B_xpe_2.44_pic_0.12/0.41_maj_0.93/0.69_css1067
47.0_IN_B_xpe_2.22_pic_0.21/0.35_maj_0.88/0.76_css1072
51.0_IN_B_rsb_2.56_pic_0.19/0.35_maj_0.89/0.76_css1070
57.0_pac_B_xpe_2.35_pic_0.2/0.39_maj_0.88/0.7_css1075
53.0_IN_B_xpe_2.16_pic_0.29/0.43_maj_0.81/0.64_css1084
1_FEH_w3***
143.0_IN_B_rsb_2.61_pic_0.27/0.39_maj_0.83/0.67_css1086
143.0_IN_B_xpe_2.55_pic_0.27/0.39_maj_0.83/0.67_css1093
168.0_MN_B_rsb_2.90_pic_0.38/0.42_maj_0.73/0.65_css1094
131.0_CA_B_rsb_2.99_pic_0.39/0.43_maj_0.69/0.66_css1096
141.0_CA_B_xpe_2.58_pic_0.35/0.39_maj_0.74/0.68_css1097
143.0_pac_B_rsb_3.01_pic_0.34/0.39_maj_0.76/0.68_css1099
143.0_MN_B_xpe_2.71_pic_0.36/0.4_maj_0.74/0.68_css1098
175.0_pac_B_xpe_3.04_pic_0.36/0.43_maj_0.73/0.65_css1100
191.0_ID_B_rsb_3.19_pic_0.4/0.42_maj_0.69/0.66_css1104
143.0_ID_B_xpe_2.85_pic_0.36/0.38_maj_0.72/0.7_css1111
141.0_SC_B_xpe_2.14_pic_0.28/0.4_maj_0.8/0.66_css1116
143.0_SC_B_rsb_2.67_pic_0.3/0.32_maj_0.77/0.77_css1118
Gpc_B1***
172.0_MN_B_xpe_2.79_pic_0.39/0.46_maj_0.72/0.61_css1121
194.0_ID_B_xpe_2.93_pic_0.42/0.44_maj_0.67/0.63_css1120
151.0_IN_B_rsb_2.26_pic_0.39/0.45_maj_0.71/0.62_css1130
153.0_IN_B_xpe_2.05_pic_0.36/0.47_maj_0.75/0.61_css1134
168.0_CA_B_xpe_2.63_pic_0.39/0.47_maj_0.7/0.6_css1135
175.0_pac_B_rsb_2.98_pic_0.4/0.46_maj_0.69/0.6_css1137
165.0_CA_B_rsb_2.72_pic_0.41/0.46_maj_0.69/0.61_css1138
160.0_IN_B_rsb_2.41_pic_0.42/0.48_maj_0.69/0.57_css1140
168.0_nor_B_rsb_3.11_pic_0.45/0.45_maj_0.63/0.62_css1145
168.0_IN_B_xpe_2.54_pic_0.39/0.46_maj_0.71/0.61_css1144
161.0_pnw_B_rsb_2.88_pic_0.42/0.41_maj_0.68/0.67_css1151
166.0_ND_B_rsb_2.75_pic_0.41/0.44_maj_0.68/0.64_css1150
168.0_SC_B_xpe_3.16_pic_0.34/0.44_maj_0.74/0.63_css1152
163.0_MT_B_rsb_2.81_pic_0.46/0.43_maj_0.61/0.65_css1155
165.0_ND_B_xpe_2.69_pic_0.41/0.44_maj_0.69/0.65_css1156
165.0_nor_B_xpe_2.63_pic_0.45/0.44_maj_0.62/0.65_css1157
165.0_TX_B_xpe_2.75_pic_0.27/0.44_maj_0.83/0.65_css1159
168.0_SC_B_rsb_2.95_pic_0.37/0.45_maj_0.7/0.61_css1162
187.0_ID_B_fst_0.27_pic_0.49/0.31_maj_0.55/0.81_css1176
187.0_MN_B_rsb_2.41_pic_0.5/0.32_maj_0.5/0.8_css1177
187.0_pac_B_rsb_2.16_pic_0.46/0.33_maj_0.63/0.79_css1179
201.0_MN_B_xpe_2.24_pic_0.34/0.38_maj_0.72/0.69_css1188
218.0_ID_B_xpe_2.67_pic_0.35/0.43_maj_0.75/0.64_css1187
203.0_ID_B_rsb_2.95_pic_0.36/0.36_maj_0.71/0.73_css1191
218.0_MN_B_xpe_2.11_pic_0.27/0.5_maj_0.83/0.53_css1201
240.0_ID_B_xpe_2.44_pic_0.33/0.42_maj_0.77/0.66_css1203
475.0_pnw_B_rsb_2.63_pic_0.43/0.41_maj_0.66/0.67_css1241
483.0_ID_B_rsb_2.74_pic_0.34/0.41_maj_0.76/0.67_css1235
483.0_pnw_B_xpe_3.19_pic_0.4/0.41_maj_0.69/0.67_css1242
488.0_ID_B_xpe_3.45_pic_0.35/0.42_maj_0.74/0.66_css1236
497.0_MN_B_xpe_2.89_pic_0.36/0.43_maj_0.74/0.65_css1237
497.0_nor_B_xpe_3.00_pic_0.39/0.43_maj_0.7/0.65_css1238
497.0_WA_B_xpe_2.73_pic_0.42/0.43_maj_0.65/0.65_css1251
497.0_pac_B_xpe_2.56_pic_0.33/0.43_maj_0.75/0.65_css1253
483.0_MT_B_xpe_2.65_pic_0.38/0.41_maj_0.71/0.68_css1260
483.0_CA_B_xpe_2.46_pic_0.26/0.41_maj_0.82/0.67_css1263
483.0_MN_B_rsb_2.44_pic_0.36/0.41_maj_0.72/0.67_css1267
483.0_ND_B_xpe_2.36_pic_0.25/0.42_maj_0.83/0.67_css1268
485.0_NE_B_xpe_2.14_pic_0.39/0.42_maj_0.71/0.65_css1269
544.0_MN_B_xpe_2.23_pic_0.26/0.46_maj_0.83/0.6_css1278
570.0_MN_B_xpe_2.17_pic_0.25/0.47_maj_0.83/0.58_css1300
590.0_NE_B_xpe_2.27_pic_0.3/0.41_maj_0.8/0.66_css1308
607.0_NE_B_xpe_2.13_pic_0.2/0.37_maj_0.88/0.74_css1310
607.0_MN_B_rsb_2.29_pic_0.48/0.32_maj_0.6/0.78_css1324
609.0_gpl_B_rsb_2.22_pic_0.32/0.36_maj_0.8/0.75_css1321
652.0_SC_B_rsb_2.63_pic_0.45/0.31_maj_0.6/0.79_css1346
665.0_gpl_B_rsb_2.89_pic_0.32/0.42_maj_0.79/0.67_css1342
665.0_NE_B_rsb_2.91_pic_0.26/0.42_maj_0.84/0.67_css1344
665.0_NE_B_xpe_2.88_pic_0.26/0.42_maj_0.84/0.67_css1345
656.0_TX_B_xpe_2.56_pic_0.26/0.41_maj_0.82/0.68_css1347
657.0_gpl_B_xpe_2.77_pic_0.3/0.4_maj_0.8/0.69_css1348
656.0_TX_B_rsb_2.70_pic_0.35/0.44_maj_0.75/0.63_css1354
656.0_KS_B_rsb_2.76_pic_0.41/0.46_maj_0.69/0.62_css1355
658.0_SC_B_rsb_2.47_pic_0.27/0.39_maj_0.84/0.71_css1357
661.0_IN_B_rsb_2.29_pic_0.49/0.41_maj_0.55/0.69_css1361
L
6B
F
29.0_pac_S_xpe_2.31_pic_0.48/0.47_maj_0.59/0.61_css1062
39.0_pac_S_xpe_2.90_pic_0.11/0.35_maj_0.94/0.76_css1068
47.0_pac_S_rsb_2.61_pic_0.18/0.25_maj_0.89/0.84_css1074
59.0_pac_S_xpe_2.60_pic_0.18/0.35_maj_0.89/0.73_css1076
57.0_CA_S_xpe_2.13_pic_0.16/0.36_maj_0.9/0.71_css1082
55.0_CA_S_fst_0.20_pic_0.18/0.47_maj_0.89/0.57_css1083
55.0_pac_S_fst_0.20_pic_0.18/0.47_maj_0.9/0.58_css1085
1_FEH_w3***
141.0_ID_S_xpe_2.16_pic_0.21/0.38_maj_0.86/0.72_css1114
Gpc_B1***
163.0_ID_S_xpe_2.08_pic_0.28/0.47_maj_0.82/0.61_css1153
187.0_ID_S_fst_0.25_pic_0.28/0.49_maj_0.83/0.57_css1175
218.0_CA_S_fst_0.22_pic_0.47/0.37_maj_0.59/0.74_css1200
470.0_CA_S_fst_0.20_pic_0.47/0.36_maj_0.6/0.76_css1252
590.0_WA_S_xpe_2.77_pic_0.36/0.41_maj_0.74/0.68_css1299
590.0_WA_S_rsb_2.70_pic_0.38/0.4_maj_0.72/0.69_css1304
619.0_WA_S_rsb_3.01_pic_0.47/0.46_maj_0.6/0.62_css1317
619.0_WA_S_xpe_3.13_pic_0.47/0.46_maj_0.6/0.62_css1318
626.0_pnw_S_xpe_2.11_pic_0.46/0.46_maj_0.59/0.62_css1311
648.0_pnw_S_xpe_2.69_pic_0.46/0.45_maj_0.58/0.6_css1332
672.0_WA_S_xpe_3.56_pic_0.36/0.38_maj_0.73/0.71_css1337
680.0_WA_S_rsb_3.47_pic_0.35/0.37_maj_0.74/0.72_css1336
647.0_pnw_S_rsb_2.18_pic_0.49/0.49_maj_0.53/0.54_css1341
670.0_HRS_S_fst_0.38_pic_0.12/0.45_maj_0.93/0.62_css1363
670.0_SWS_S_fst_0.37_pic_0.46/0.17_maj_0.6/0.9_css1365
L
6B
F
39.0_IN_W_xpe_2.97_pic_0.27/0.44_maj_0.84/0.64_css1066
59.0_IN_W_rsb_2.79_pic_0.24/0.43_maj_0.85/0.66_css1071
59.0_IN_W_xpe_2.59_pic_0.24/0.43_maj_0.85/0.66_css1073
1_FEH_w3***
145.0_IN_W_rsb_3.03_pic_0.27/0.36_maj_0.83/0.72_css1087
168.0_IN_W_xpe_2.75_pic_0.33/0.4_maj_0.77/0.68_css1088
134.0_SC_W_xpe_2.18_pic_0.26/0.39_maj_0.82/0.7_css1108
151.0_SC_W_rsb_2.75_pic_0.32/0.31_maj_0.74/0.78_css1117
Gpc_B1***
152.0_eas_W_rsb_2.46_pic_0.46/0.42_maj_0.59/0.65_css1127
152.0_SRW_W_rsb_2.92_pic_0.46/0.44_maj_0.6/0.63_css1133
165.0_IN_W_rsb_2.91_pic_0.4/0.44_maj_0.71/0.64_css1131
175.0_SC_W_xpe_2.63_pic_0.32/0.44_maj_0.75/0.63_css1132
165.0_ID_W_rsb_3.06_pic_0.43/0.45_maj_0.66/0.61_css1139
163.0_SRW_W_rsb_3.17_pic_0.45/0.44_maj_0.63/0.63_css1142
165.0_ID_W_xpe_2.93_pic_0.38/0.42_maj_0.71/0.67_css1149
163.0_KS_W_rsb_2.97_pic_0.41/0.41_maj_0.7/0.69_css1154
168.0_SC_W_rsb_3.39_pic_0.34/0.39_maj_0.74/0.7_css1158
168.0_NY_W_xpe_2.57_pic_0.37/0.41_maj_0.73/0.67_css1161
191.0_SC_W_rsb_2.08_pic_0.42/0.37_maj_0.67/0.7_css1185
265.0_IN_W_fst_0.38_pic_0.44/0.33_maj_0.67/0.79_css1213
272.0_NY_W_fst_0.43_pic_0.39/0.33_maj_0.73/0.79_css1214
485.0_WA_W_xpe_2.44_pic_0.37/0.41_maj_0.69/0.69_css1254
483.0_SC_W_xpe_2.22_pic_0.34/0.4_maj_0.75/0.69_css1258
483.0_pnw_W_xpe_2.42_pic_0.39/0.41_maj_0.69/0.69_css1259
483.0_NE_W_xpe_2.29_pic_0.37/0.39_maj_0.73/0.69_css1262
482.0_IN_W_xpe_2.20_pic_0.32/0.4_maj_0.77/0.7_css1264
482.0_OR_W_xpe_2.18_pic_0.33/0.39_maj_0.75/0.7_css1265
482.0_SWW_W_xpe_2.16_pic_0.33/0.38_maj_0.75/0.71_css1270
497.0_WA_W_xpe_2.13_pic_0.43/0.45_maj_0.64/0.58_css1273
575.0_NY_W_xpe_2.03_pic_0.16/0.44_maj_0.91/0.6_css1301
607.0_gpl_W_rsb_2.54_pic_0.3/0.17_maj_0.82/0.89_css1322
607.0_KS_W_rsb_2.27_pic_0.3/0.16_maj_0.81/0.9_css1323
661.0_gpl_W_xpe_2.56_pic_0.32/0.43_maj_0.78/0.65_css1343
665.0_NE_W_rsb_2.72_pic_0.28/0.43_maj_0.83/0.66_css1349
665.0_gpl_W_rsb_3.04_pic_0.37/0.45_maj_0.74/0.63_css1352
665.0_NE_W_xpe_3.00_pic_0.28/0.45_maj_0.82/0.63_css1353
L

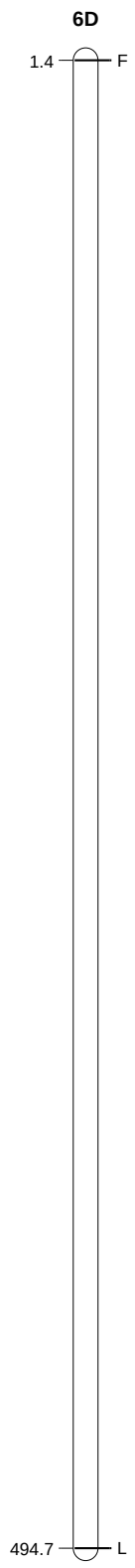
6D
1.4
F
494.7
L
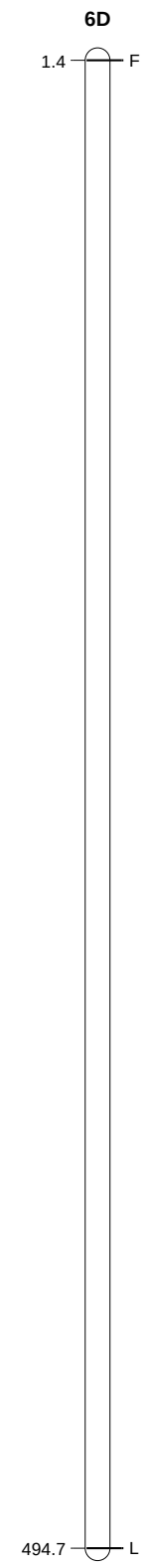
6D
1.4
F
494.7
L
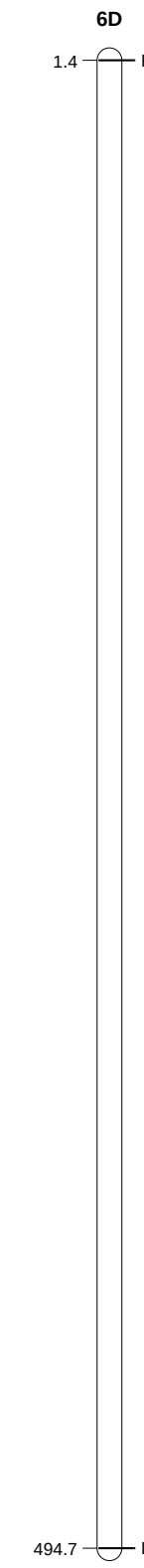
6D
1.4
F
494.7
L

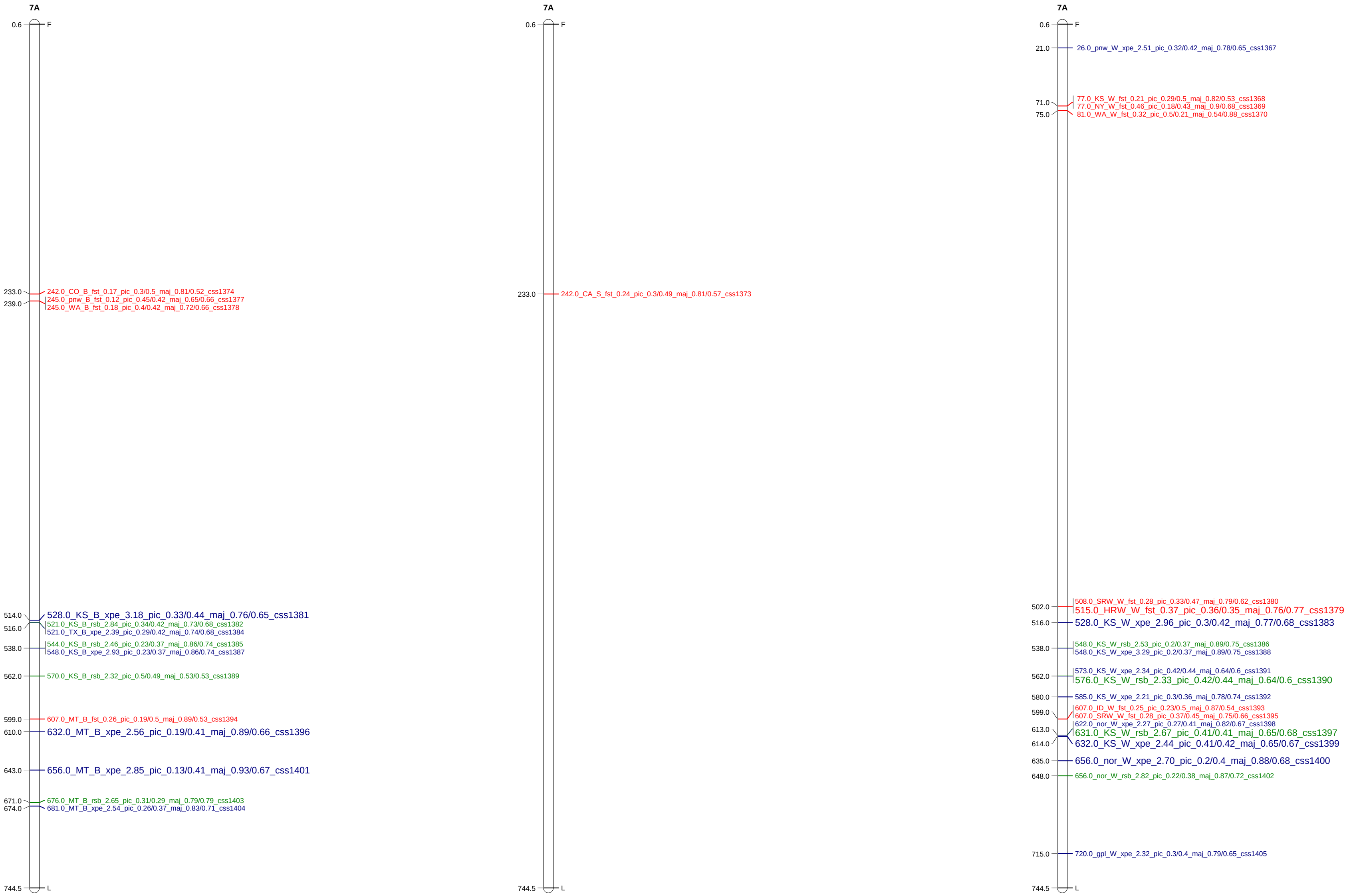

7A
0.6
F
233.0
239.0
242.0_CO_B_fst_0.17_pic_0.3/0.5_maj_0.81/0.52_css1374
245.0_pnw_B_fst_0.12_pic_0.45/0.42_maj_0.65/0.66_css1377
245.0_WA_B_fst_0.18_pic_0.4/0.42_maj_0.72/0.66_css1378
514.0
516.0
528.0_KS_B_xpe_3.18_pic_0.33/0.44_maj_0.76/0.65_css1381
521.0_KS_B_rsb_2.84_pic_0.34/0.42_maj_0.73/0.68_css1382
521.0_TX_B_xpe_2.39_pic_0.29/0.42_maj_0.74/0.68_css1384
538.0
544.0_KS_B_rsb_2.46_pic_0.23/0.37_maj_0.86/0.74_css1385
548.0_KS_B_xpe_2.93_pic_0.23/0.37_maj_0.86/0.74_css1387
562.0
570.0_KS_B_rsb_2.32_pic_0.5/0.49_maj_0.53/0.53_css1389
599.0
607.0_MT_B_fst_0.26_pic_0.19/0.5_maj_0.89/0.53_css1394
610.0
632.0_MT_B_xpe_2.56_pic_0.19/0.41_maj_0.89/0.66_css1396
643.0
656.0_MT_B_xpe_2.85_pic_0.13/0.41_maj_0.93/0.67_css1401
671.0
674.0
676.0_MT_B_rsb_2.65_pic_0.31/0.29_maj_0.79/0.79_css1403
681.0_MT_B_xpe_2.54_pic_0.26/0.37_maj_0.83/0.71_css1404
744.5
L
7A
0.6
F
233.0
242.0_CA_S_fst_0.24_pic_0.3/0.49_maj_0.81/0.57_css1373
744.5
L
7A
0.6
F
21.0
26.0_pnw_W_xpe_2.51_pic_0.32/0.42_maj_0.78/0.65_css1367
71.0
75.0
77.0_KS_W_fst_0.21_pic_0.29/0.5_maj_0.82/0.53_css1368
77.0_NY_W_fst_0.46_pic_0.18/0.43_maj_0.9/0.68_css1369
81.0_WA_W_fst_0.32_pic_0.5/0.21_maj_0.54/0.88_css1370
502.0
508.0_SRW_W_fst_0.28_pic_0.33/0.47_maj_0.79/0.62_css1380
515.0_HRW_W_fst_0.37_pic_0.36/0.35_maj_0.76/0.77_css1379
516.0
528.0_KS_W_xpe_2.96_pic_0.3/0.42_maj_0.77/0.68_css1383
538.0
548.0_KS_W_rsb_2.53_pic_0.2/0.37_maj_0.89/0.75_css1386
548.0_KS_W_xpe_3.29_pic_0.2/0.37_maj_0.89/0.75_css1388
562.0
573.0_KS_W_xpe_2.34_pic_0.42/0.44_maj_0.64/0.6_css1391
576.0_KS_W_rsb_2.33_pic_0.42/0.44_maj_0.64/0.6_css1390
580.0
585.0_KS_W_xpe_2.21_pic_0.3/0.36_maj_0.78/0.74_css1392
599.0
613.0
614.0
607.0_ID_W_fst_0.25_pic_0.23/0.5_maj_0.87/0.54_css1393
607.0_SRW_W_fst_0.28_pic_0.37/0.45_maj_0.75/0.66_css1395
622.0_nor_W_xpe_2.27_pic_0.27/0.41_maj_0.82/0.67_css1398
631.0_KS_W_rsb_2.67_pic_0.41/0.41_maj_0.65/0.68_css1397
632.0_KS_W_xpe_2.44_pic_0.41/0.42_maj_0.65/0.67_css1399
635.0
656.0_nor_W_xpe_2.70_pic_0.2/0.4_maj_0.88/0.68_css1400
648.0
656.0_nor_W_rsb_2.82_pic_0.22/0.38_maj_0.87/0.72_css1402
715.0
720.0_gpl_W_xpe_2.32_pic_0.3/0.4_maj_0.79/0.65_css1405
744.5
L

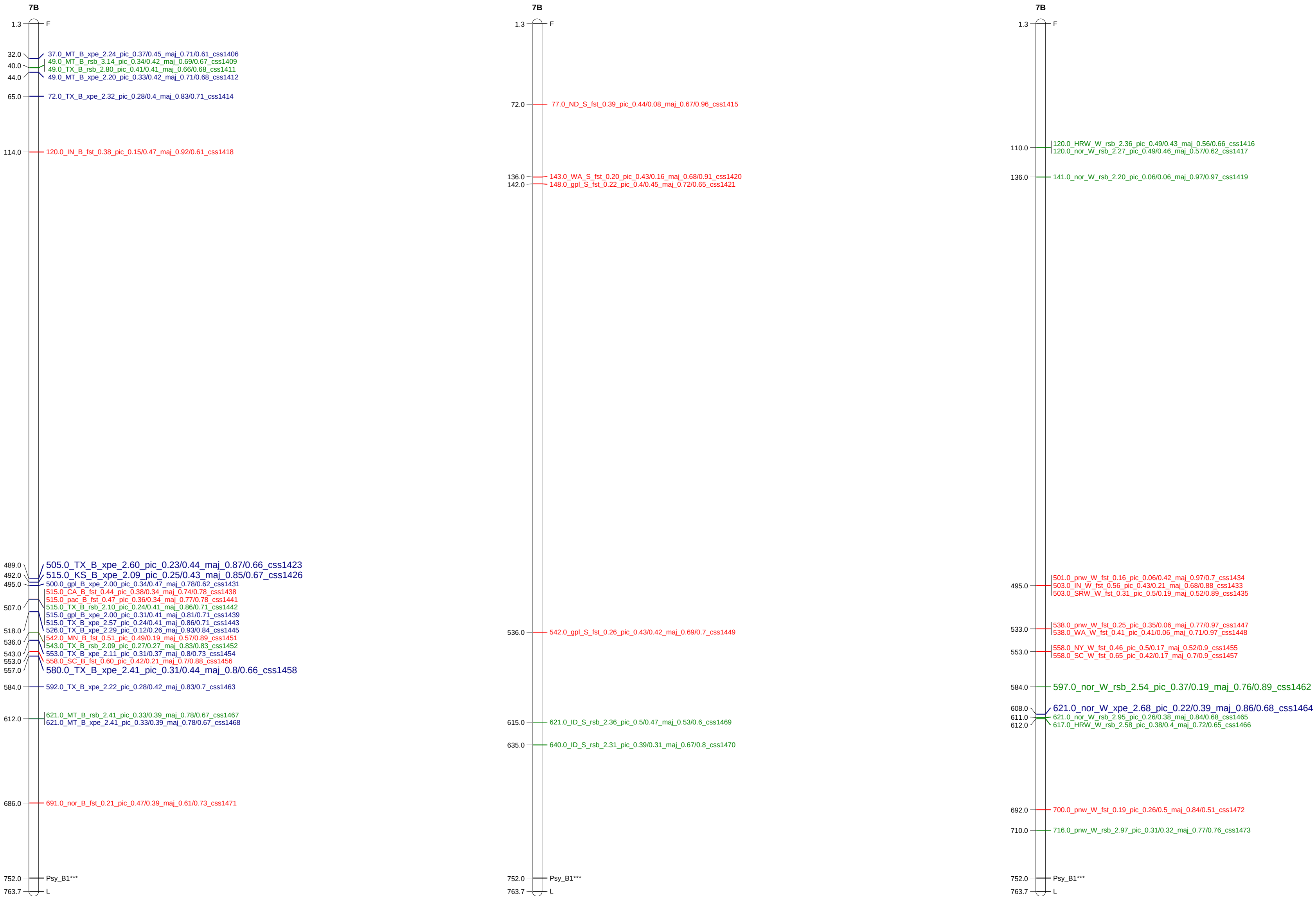

7B
1.3 F
32.0 37.0_MT_B_xpe_2.24_pic_0.37/0.45_maj_0.71/0.61_css1406
40.0 49.0_MT_B_rsb_3.14_pic_0.34/0.42_maj_0.69/0.67_css1409
44.0 49.0_TX_B_rsb_2.80_pic_0.41/0.41_maj_0.66/0.68_css1411
49.0_MT_B_xpe_2.20_pic_0.33/0.42_maj_0.71/0.68_css1412
65.0 72.0_TX_B_xpe_2.32_pic_0.28/0.4_maj_0.83/0.71_css1414
114.0 120.0_IN_B_fst_0.38_pic_0.15/0.47_maj_0.92/0.61_css1418
489.0 505.0_TX_B_xpe_2.60_pic_0.23/0.44_maj_0.87/0.66_css1423
492.0 515.0_KS_B_xpe_2.09_pic_0.25/0.43_maj_0.85/0.67_css1426
495.0 500.0_gpl_B_xpe_2.00_pic_0.34/0.47_maj_0.78/0.62_css1431
515.0_CA_B_fst_0.44_pic_0.38/0.34_maj_0.74/0.78_css1438
507.0 515.0_pac_B_fst_0.47_pic_0.36/0.34_maj_0.77/0.78_css1441
515.0_TX_B_rsb_2.10_pic_0.24/0.41_maj_0.86/0.71_css1442
515.0_gpl_B_xpe_2.00_pic_0.31/0.41_maj_0.81/0.71_css1439
515.0_TX_B_xpe_2.57_pic_0.24/0.41_maj_0.86/0.71_css1443
518.0 526.0_TX_B_xpe_2.29_pic_0.12/0.26_maj_0.93/0.84_css1445
536.0 542.0_MN_B_fst_0.51_pic_0.49/0.19_maj_0.57/0.89_css1451
543.0_TX_B_rsb_2.09_pic_0.27/0.27_maj_0.83/0.83_css1452
543.0 553.0_TX_B_xpe_2.11_pic_0.31/0.37_maj_0.8/0.73_css1454
553.0 558.0_SC_B_fst_0.60_pic_0.42/0.21_maj_0.7/0.88_css1456
557.0 580.0_TX_B_xpe_2.41_pic_0.31/0.44_maj_0.8/0.66_css1458
584.0 592.0_TX_B_xpe_2.22_pic_0.28/0.42_maj_0.83/0.7_css1463
612.0 621.0_MT_B_rsb_2.41_pic_0.33/0.39_maj_0.78/0.67_css1467
621.0_MT_B_xpe_2.41_pic_0.33/0.39_maj_0.78/0.67_css1468
686.0 691.0_nor_B_fst_0.21_pic_0.47/0.39_maj_0.61/0.73_css1471
752.0 Psy_B1***
763.7 L
7B
1.3 F
72.0 77.0_ND_S_fst_0.39_pic_0.44/0.08_maj_0.67/0.96_css1415
136.0 143.0_WA_S_fst_0.20_pic_0.43/0.16_maj_0.68/0.91_css1420
142.0 148.0_gpl_S_fst_0.22_pic_0.4/0.45_maj_0.72/0.65_css1421
536.0 542.0_gpl_S_fst_0.26_pic_0.43/0.42_maj_0.69/0.7_css1449
615.0 621.0_ID_S_rsb_2.36_pic_0.5/0.47_maj_0.53/0.6_css1469
635.0 640.0_ID_S_rsb_2.31_pic_0.39/0.31_maj_0.67/0.8_css1470
752.0 Psy_B1***
763.7 L
7B
1.3 F
110.0 120.0_HRW_W_rsb_2.36_pic_0.49/0.43_maj_0.56/0.66_css1416
120.0_nor_W_rsb_2.27_pic_0.49/0.46_maj_0.57/0.62_css1417
136.0 141.0_nor_W_rsb_2.20_pic_0.06/0.06_maj_0.97/0.97_css1419
501.0_pnw_W_fst_0.16_pic_0.06/0.42_maj_0.97/0.7_css1434
495.0 503.0_IN_W_fst_0.56_pic_0.43/0.21_maj_0.68/0.88_css1433
503.0_SRW_W_fst_0.31_pic_0.5/0.19_maj_0.52/0.89_css1435
533.0 538.0_pnw_W_fst_0.25_pic_0.35/0.06_maj_0.77/0.97_css1447
538.0_WA_W_fst_0.41_pic_0.41/0.06_maj_0.71/0.97_css1448
553.0 558.0_NY_W_fst_0.46_pic_0.5/0.17_maj_0.52/0.9_css1455
558.0_SC_W_fst_0.65_pic_0.42/0.17_maj_0.7/0.9_css1457
584.0 597.0_nor_W_rsb_2.54_pic_0.37/0.19_maj_0.76/0.89_css1462
608.0 621.0_nor_W_xpe_2.68_pic_0.22/0.39_maj_0.86/0.68_css1464
611.0 621.0_nor_W_rsb_2.95_pic_0.26/0.38_maj_0.84/0.68_css1465
612.0 617.0_HRW_W_rsb_2.58_pic_0.38/0.4_maj_0.72/0.65_css1466
692.0 700.0_pnw_W_fst_0.19_pic_0.26/0.5_maj_0.84/0.51_css1472
710.0 716.0_pnw_W_rsb_2.97_pic_0.31/0.32_maj_0.77/0.76_css1473
752.0 Psy_B1***
763.7 L

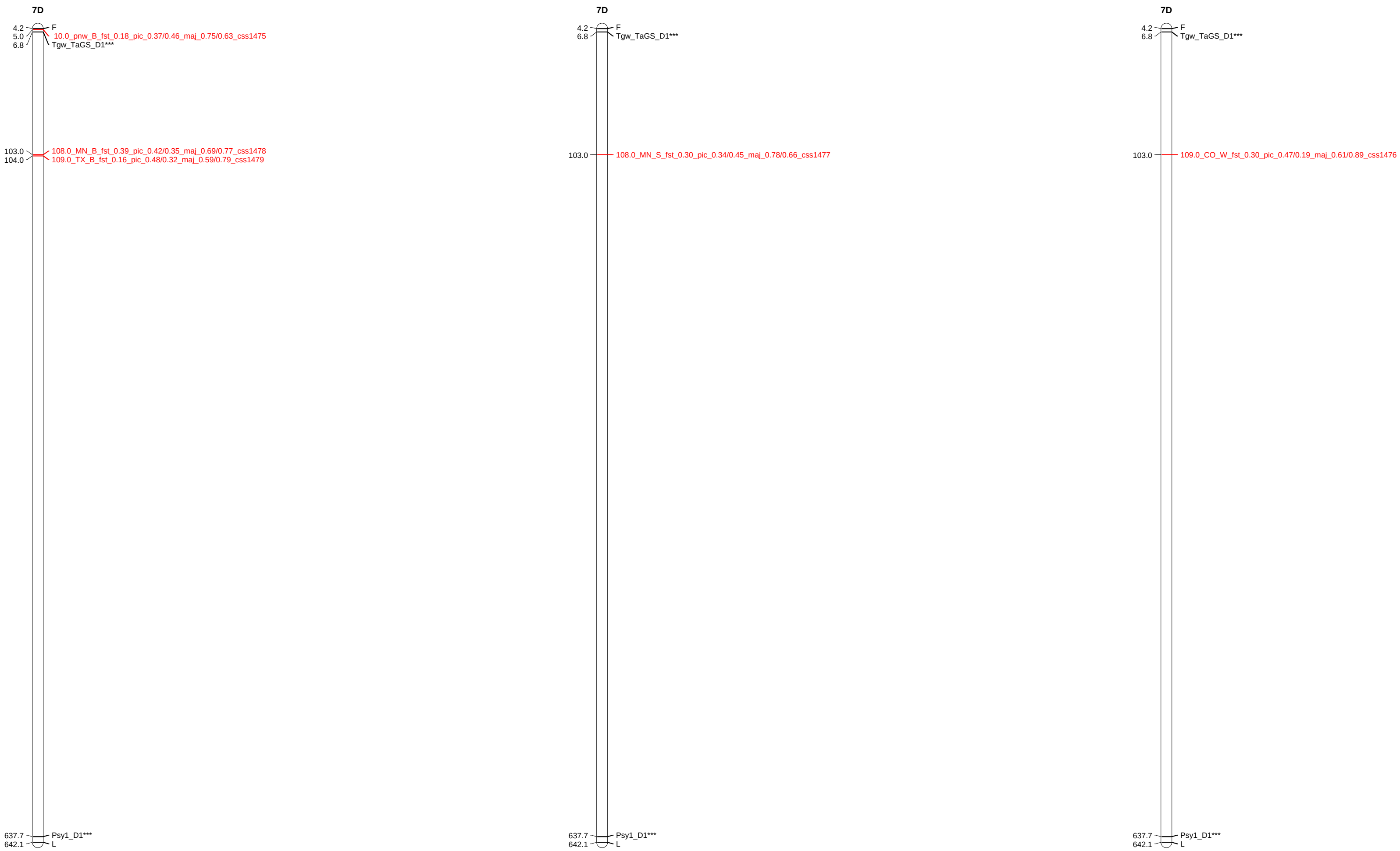

Supplemental Figure S4. Map of candidate selective sweeps (CSS) in U.S. wheat due to selection across regional, state, and market class populations. The linkage blocks from left to right show CSS in population pairs with a) both spring and winter varieties, b) just spring varieties, and c) just winter varieties. Physical positions in Mbp for the start of the CSS are on the left side of the bar. The right side includes end position of the CSS, name of the population selected in (eas, Eastern; gpl, the Great Plains; nor, Northern, pac, the Pacific; pnw, the Pacific Northwest; HRS, hard red spring; HRW, hard red winter; SRW, soft red winter; SWS, soft white spring; SWW, soft white winter), growth habit (B, S, and W for both, spring, and winter), statistic and its maximum value, PIC values in target and reference population, major allele frequencies in the target and reference population, and CSS serial number. Red, green, and blue color of the label indicate CSS detected using F_{st}, Rsb, and xpEHH respectively. Size of the label corresponds with the size of the CSS. Location of known genes are indicated by (***) and F and L refer to the physical positions of the first and last SNP genotyped on the chromosome.
