# Supplementary material for: Candidate selective sweeps in US wheat populations
Source: Plant Genome. 2024 Sep 25;17(4):e20513. doi: 10.1002/tpg2.20513 (PMC11628914; doi:10.1002/tpg2.20513)
Supplement: Supplementary file 1 — Supplemental Table S1. Wheat variety panel used for the study. Supplemental Table S2. Number of varieties and variety release years in populations used for Fst, Rsb, and xpEHH calculations. Supplemental Table S3. Sequences for known informative markers and their physical positions on the ‘Chinese Spring’ wheat reference genome version 2.1. Supplemental Table S4. Candidate selective sweeps in U.S. wheat populations. [file TPG2-17-e20513-s004.docx]

Supplemental Table S1. Wheat variety panel used for the study. Also available as /data/variety_details.txt at https://datadryad.org/stash/share/62lamvCW7tXg4-O-xY0beBGR7CODFUwSQK2aTjXTjV8.

| **Variety** | **ACNO** | **Year^a^** | **State** | **Region** | **Class** | **Headtype** | **Habit** |
| --- | --- | --- | --- | --- | --- | --- | --- |
| 711 | PI486142 | 1984 | NE | Great Plains | HRS | common | spring |
| 715 | PI486143 | 1981 | NE | Great Plains | HRS | common | spring |
| 751 | PI486144 | 1982 | NE | Great Plains | HRS | common | spring |
| 771 | PI486145 | 1980 | CA | Pacific | HRS | common | spring |
| 775 | PI601334 | 1986 | CA | Pacific | HRS | common | spring |
| 830 | PI486148 | 1982 | NE | Great Plains | HRW | common | winter |
| 835 | PI486149 | 1981 | NE | Great Plains | HRW | common | winter |
| 2154 | PI601366 | 1987 | KS | Great Plains | HRW | common | winter |
| 2157 | PI601070 | 1984 | IA | Eastern | HRW | common | winter |
| 2158 | PI601723 | 1990 | KS | Great Plains | HRW | common | winter |
| 2163 | PI601722 | 1989 | KS | Great Plains | HRW | common | winter |
| 2165 | PI601069 | 1984 | KS | Great Plains | HRW | common | winter |
| 2172 | PI601071 | 1984 | KS | Great Plains | HRW | common | winter |
| 2369 | PI600932 | 1982 | MN | Northern | HRS | common | spring |
| 2375 | PI601477 | 1989 | MN | Northern | HRS | common | spring |
| 2510 | PI550696 | 1991 | IN | Eastern | SRW | common | winter |
| 2545 | PI550697 | 1991 | IN | Eastern | SRW | common | winter |
| 2551 | PI601207 | 1985 | IN | Eastern | SRW | common | winter |
| 2552 | PI566924 | 1993 | IN | Eastern | SRW | common | winter |
| 2553 | PI601103 | 1982 | IN | Eastern | SRW | common | winter |
| 2555 | PI532914 | 1986 | IA | Eastern | SRW | common | winter |
| 2566 | PI561199 | 1993 | SC | Eastern | SRW | common | winter |
| 2571 | PI561200 | 1992 | SC | Eastern | SRW | common | winter |
| 4555 | CItr598213 | 1977 | KS | Great Plains | HRW | common | winter |
| 4578 | CItr599987 | 1978 | KS | Great Plains | HRW | common | winter |
| 5221 | CItr598214 | 1976 | KS | Great Plains | HRW | common | winter |
| 5232 | CItr598212 | 1976 | KS | Great Plains | HRW | common | winter |
| 5411 | CItr598210 | 1973 | KS | Great Plains | HRW | common | winter |
| 5422 | CItr595216 | 1977 | KS | Great Plains | HRW | common | winter |
| 5466 | CItr596624 | 1978 | KS | Great Plains | HRW | common | winter |
| 5469 | PI601249 | 1985 | KS | Great Plains | HRW | common | winter |
| 5630 | PI601248 | 1985 | KS | Great Plains | HRW | common | winter |
| 7805 | PI601606 | 1988 | KS | Great Plains | HRW | common | winter |
| 7833 | PI601607 | 1988 | KS | Great Plains | HRW | common | winter |
| 7837 | PI601608 | 1988 | KS | Great Plains | HRW | common | winter |
| 2737W | PI561197 | 1992 | IN | Eastern | SWW | common | winter |
| A99AR | PI600923 | 1982 | MN | Northern | HRS | common | spring |
| Abe | CItr15375 | 1972 | IN | Eastern | SRW | common | winter |
| Abilene | PI511307 | 1987 | KS | Great Plains | HRW | common | winter |
| Ace | CItr13384 | 1960 | AR | Eastern | SRW | common | winter |
| Adams | CItr13722 | 1968 | OR | PNW | HWS | common | spring |
| Adena | PI481852 | 1984 | OH | Eastern | SRW | common | winter |
| Admire | PI583810 | 1994 | AZ | Pacific | HRS | common | spring |
| Agassiz | PI478771 | 1983 | ND | Northern | HRW | common | winter |
| Agent | CItr13523 | 1967 | OK | Great Plains | HRW | common | winter |
| Agrus | CItr13228 | 1972 | IN | Eastern | SRW | common | winter |
| Akron | PI584504 | 1994 | CO | Great Plains | HRW | common | winter |
| Albit | CItr8275 | 1926 | WA | PNW | SWW | club | winter |
| Alex | PI17910 | 1981 | ND | Northern | HRS | common | spring |
| Alice | PI644223 | 2006 | SD | Northern | HWW | common | winter |
| Alicel | CItr11700 | 1932 | OR | PNW | SWW | club | winter |
| Allegiance | PI641952 | 2002 | KY | Eastern | SRW | common | winter |
| Alliance | PI573096 | 1993 | NE | Great Plains | HRW | common | winter |
| Alpowa | PI566596 | 1994 | WA | PNW | SWS | common | spring |
| Amidon | PI527682 | 1988 | ND | Northern | HRS | common | spring |
| Anderson | CItr12536 | 1954 | SC | Eastern | SRW | common | winter |
| Andnox | CItr13907 | 1966 | SC | Eastern | SRW | common | winter |
| Andrews | PI512282 | 1987 | WA | PNW | HRW | common | winter |
| Angus | CItr17744 | 1978 | MN | Northern | HRS | common | spring |
| Ankor | PI632275 | 2002 | CO | Great Plains | HRW | common | winter |
| Antelope | PI633910 | 2003 | NE | Great Plains | HWW | common | winter |
| Anza | CItr15284 | 1971 | CA | Pacific | HRS | common | spring |
| Apex 83 | PI601073 | 1983 | AZ | Pacific | HRS | common | spring |
| AR 910 | PI636470 | 2004 | AR | Eastern | SRW | common | winter |
| Arapahoe | PI518591 | 1988 | NE | Great Plains | HRW | common | winter |
| Arcadian | CItr565362 | 1895 | NY | Eastern | SWW | common | winter |
| Archer | CItr17940 | 1981 | CO | Great Plains | HRW | common | winter |
| Arco | CItr8246 | 1928 | OR | PNW | SWW | common | winter |
| Ark | CItr15286 | 1972 | ID | PNW | HRW | common | winter |
| Arkan | PI475771 | 1982 | KS | Great Plains | HRW | common | winter |
| Arkansas No. 105 | CItr565390 | 1918 | AR | Eastern | SRW | common | winter |
| Arrow | CItr15079 | 1971 | NY | Eastern | SWW | common | winter |
| Arrowsmith | PI633911 | 2003 | NE | Great Plains | HWW | common | winter |
| ARS-Amber | PI665047 | 2012 | WA | PNW | SWW | common | winter |
| ARS-Chrystal | PI665049 | 2012 | WA | PNW | SWW | club | winter |
| ARS-Crescent | PI665048 | 2012 | WA | PNW | SWW | club | winter |
| ARS-Selbu | PI667744 | 2013 | WA | PNW | SWW | common | winter |
| Arthur | CItr14425 | 1968 | IN | Eastern | SRW | common | winter |
| Arthur 71 | CItr15282 | 1971 | IN | Eastern | SRW | common | winter |
| Ashkof | CItr6680 | 1923 | WI | Eastern | HRW | common | winter |
| Athena | CItr11693 | 1931 | OR | PNW | SWW | common | winter |
| Atlas 50 | CItr12534 | 1948 | NC | Eastern | SRW | common | winter |
| Atlas 66 | CItr12561 | 1948 | NC | Eastern | SRW | common | winter |
| ATW 270 | PI601806 | 1989 | FL | Eastern | SRW | common | winter |
| Auburn | PI17898 | 1981 | IN | Eastern | SRW | common | winter |
| Augusta | CItr17831 | 1979 | MI | Eastern | SWW | common | winter |
| Austin | CItr12346 | 1942 | TX | Great Plains | SRW | common | winter |
| Avalanche | PI620766 | 2001 | CO | Great Plains | HWW | common | winter |
| Avoca | CItr13395 | 1954 | OK | Great Plains | HRW | common | winter |
| Awned Onas | CItr12235 | 1950 | CA | Pacific | SWS | common | spring |
| Awnless Baart | CItr12236 | 1942 | CA | Pacific | HWS | common | spring |
| Aztec | CItr13016 | 1958 | NM | Great Plains | HRW | common | winter |
| Baart 38 | CItr11907 | 1939 | CA | Pacific | HWS | common | spring |
| Baart 46 | CItr12386 | 1948 | CA | Pacific | HWS | common | spring |
| Baca | CItr15891 | 1973 | CO | Great Plains | HRW | common | winter |
| BacUp | PI596533 | 1996 | MN | Northern | HRS | common | spring |
| Bailey 4287 | PI600859 | 1982 | IN | Eastern | SRW | common | winter |
| Baker | PI573002 | 1987 | AZ | Pacific | HRS | common | spring |
| Baldrock | CItr11538 | 1931 | MI | Eastern | SRW | common | winter |
| Bannock | CItr15318 | 1972 | ID | PNW | HRS | common | spring |
| Barbee | CItr17417 | 1976 | WA | PNW | SWW | club | winter |
| Basin | PI601238 | 1984 | WA | PNW | SWW | common | winter |
| Beau | CItr17420 | 1976 | IN | Eastern | SRW | common | winter |
| Bennett | CItr17723 | 1978 | NE | Great Plains | HRW | common | winter |
| Bergen | PI538768 | 1990 | CO | Great Plains | HRS | common | spring |
| Big Club 37 | CItr11901 | 1937 | CA | Pacific | SWS | club | spring |
| Bison | CItr12518 | 1956 | KS | Great Plains | HRW | common | winter |
| Blackhawk | CItr12218 | 1944 | WI | Eastern | SRW | common | winter |
| Blackhull | CItr6251 | 1917 | KS | Great Plains | HRW | common | winter |
| Blanca | PI501533 | 1986 | ID | PNW | SWS | common | spring |
| Blizzard | PI512302 | 1989 | ID | PNW | HRW | common | winter |
| Blount's Lambrigg | CItr42207 | 1916 | CO | Great Plains | xxW | common | winter |
| Blue Jacket | CItr12502 | 1946 | KS | Great Plains | HRW | common | winter |
| Blueboy | CItr14031 | 1967 | NC | Eastern | SRW | common | winter |
| Bonanza | CItr14077 | 1969 | KS | Great Plains | HRS | common | spring |
| Brill | CItr11853 | 1936 | IL | Eastern | HRW | common | winter |
| Bronze | CItr14013 | 1974 | SD | Northern | HRW | common | winter |
| Brule | PI466739 | 1981 | NE | Great Plains | HRW | common | winter |
| Buckshot | PI481541 | 1983 | CO | Great Plains | HRS | common | spring |
| Butler | CItr12527 | 1947 | OH | Eastern | SRW | common | winter |
| Caddo | CItr13536 | 1963 | TX | Great Plains | HRW | common | winter |
| Calorwa | PI566594 | 1994 | WA | PNW | SWS | club | spring |
| Canawa | CItr11854 | 1935 | WV | Eastern | SRW | common | winter |
| Capitan | CItr17591 | 1978 | NM | Great Plains | HRW | common | winter |
| Carleeds | CItr11801 | 1936 | ND | Northern | HRS | common | spring |
| Carlson's Fife | CItr11922 | 1941 | WA | PNW | SRW | common | winter |
| Carson | PI501534 | 1986 | CO | Great Plains | HRW | common | winter |
| Cataldo | PI642361 | 2007 | ID | PNW | SWS | common | spring |
| Celtic | PI502966 | 1986 | CO | Great Plains | HRS | common | spring |
| Centennial | PI537303 | 1990 | ID | PNW | SWS | common | spring |
| Cheney | CItr17765 | 1978 | KS | Great Plains | HRW | common | winter |
| Chiefkan | CItr11754 | 1935 | KS | Great Plains | HRW | common | winter |
| Clark | PI512337 | 1987 | IN | Eastern | SRW | common | winter |
| Clemson 201 | PI585044 | 1994 | SC | Eastern | SRW | common | winter |
| Cloud | CItr17276 | 1973 | KS | Great Plains | HRW | common | winter |
| Coastal | CItr12463 | 1949 | SC | Eastern | SRW | common | winter |
| Coker 47-27 | CItr12563 | 1950 | SC | Eastern | SRW | common | winter |
| Coker 61-19 | CItr13783 | 1960 | SC | Eastern | SRW | common | winter |
| Coker 68-15 | CItr15291 | 1971 | SC | Eastern | SRW | common | winter |
| Coker 762 | PI17924 | 1980 | SC | Eastern | SRW | common | winter |
| Collin | PI511849 | 1986 | TX | Great Plains | HRW | common | winter |
| Colorado No. 50 | CItr4959 | 1909 | CO | Great Plains | SWS | common | spring |
| Colorow | CItr12865 | 1960 | CO | Great Plains | HRW | common | winter |
| Colt | PI476975 | 1983 | NE | Great Plains | HRW | common | winter |
| Comet | CItr11465 | 1940 | MT | Northern | HRS | common | spring |
| Concho | CItr12517 | 1954 | OK | Great Plains | HRW | common | winter |
| Copper | PI502644 | 1986 | ID | PNW | HRS | common | spring |
| Coteau | CItr17749 | 1978 | ND | Northern | HRS | common | spring |
| Courtney | PI476848 | 1983 | CA | Pacific | HRS | common | spring |
| Crew | PI17951 | 1982 | WA | PNW | SWW | club | winter |
| Dalen | PI552812 | 1991 | CO | Great Plains | HRS | common | spring |
| Dancer | PI17890 | 1980 | CO | Great Plains | SRW | common | winter |
| Delmar | CItr13442 | 1961 | UT | PNW | HRW | common | winter |
| Deloris | PI631447 | 2002 | UT | PNW | HRW | common | winter |
| Delta Queen | CItr17893 | 1978 | SC | Eastern | SRW | common | winter |
| Dirkwin | CItr17745 | 1978 | ID | PNW | SWS | common | spring |
| DK-49S | CItr595215 | 1978 | TX | Great Plains | HRS | common | spring |
| Dodge | PI506344 | 1986 | KS | Great Plains | HRW | common | winter |
| Downy | CItr17421 | 1976 | IN | Eastern | SRW | common | winter |
| Duke | PI17856 | 1981 | CO | Great Plains | HRW | common | winter |
| Dutro Clipper | CItr10035 | 1930 | IN | Eastern | xxW | common | winter |
| Eagle Chief | CItr8868 | 1927 | OK | Great Plains | HRW | common | winter |
| Early Blackhull | CItr8856 | 1928 | KS | Great Plains | HRW | common | winter |
| Early Defiance | CItr6480 | 1878 | CA | Pacific | SWS | common | spring |
| Early Premium | CItr11858 | 1937 | MO | Eastern | SRW | common | winter |
| Edwall | PI477919 | 1984 | WA | PNW | SWS | common | spring |
| Edwin | PI606765 | 1999 | WA | PNW | SWW | common | winter |
| Eickmeyer | CItr12035 | 1939 | WA | PNW | SWW | common | winter |
| Eider | PI478896 | 1983 | IN | Eastern | SRW | common | winter |
| Eklund | CItr614656 | 1976 | MN | Northern | HRW | common | winter |
| Elgin | CItr11755 | 1942 | OR | PNW | SWW | common | winter |
| Ellar | CItr17289 | 1974 | ND | Northern | HRS | common | spring |
| Elmar | CItr12392 | 1949 | WA | PNW | SWW | common | winter |
| Eltan | PI536994 | 1990 | WA | PNW | SWW | common | winter |
| Encore | PI600839 | 1981 | KS | Great Plains | HRW | common | winter |
| Enid | CItr11508 | 1930 | OK | Great Plains | HRW | common | winter |
| Era | CItr13986 | 1970 | MN | Northern | HRS | common | spring |
| Erhardt | PI564761 | 1996 | MT | Northern | HRW | common | winter |
| Erik | PI476849 | 1983 | CO | Great Plains | HRS | common | spring |
| Escondido | CItr8240 | 1928 | CA | Pacific | SWS | common | spring |
| Escondido 41 | CItr12239 | 1942 | CA | Pacific | SWS | common | spring |
| Eureka | CItr17738 | 1978 | SD | Northern | HRS | common | spring |
| Excel | PI555465 | 1990 | OH | Eastern | SRW | common | winter |
| Exchange | CItr12635 | 1960 | IN | Eastern | xxW | common | winter |
| Express | PI573003 | 1991 | AZ | Pacific | HRS | common | spring |
| Extra Early Blackhull | CItr13664 | 1951 | TX | Great Plains | HRW | common | winter |
| F.S. 401 | PI476850 | 1983 | IN | Eastern | SRW | common | winter |
| Fairfield | CItr12013 | 1942 | IN | Eastern | SRW | common | winter |
| Faro | CItr17590 | 1976 | OR | PNW | SWW | common | winter |
| Federation 41 | CItr12230 | 1942 | CA | Pacific | SWS | common | spring |
| Federation 41M | CItr12391 | 1942 | WA | PNW | HWS | common | spring |
| Federation 67 | CItr13732 | 1967 | ID | PNW | SWS | common | spring |
| Feland | PI600924 | 1983 | SC | Eastern | SRW | common | winter |
| FFR 555W | PI560318 | 1991 | IN | Eastern | SRW | common | winter |
| Fielder | CItr17268 | 1974 | ID | PNW | SWS | common | spring |
| Fieldwin | CItr17425 | 1977 | ID | PNW | SWS | common | spring |
| Fillmore | PI469272 | 1982 | IN | Eastern | SRW | common | winter |
| Fineway | PI653509 | 2008 | WA | PNW | HRW | common | winter |
| Finley | PI586757 | 1995 | WA | PNW | HRW | common | winter |
| Fjeld | PI531243 | 1989 | KS | Great Plains | HRS | common | spring |
| Fletcher | CItr13985 | 1970 | MN | Northern | HRS | common | spring |
| Florida 302 | PI601163 | 1984 | FL | Eastern | SRW | common | winter |
| Florida 303 | PI601807 | 1988 | FL | Eastern | SRW | common | winter |
| Florida 304 | PI562528 | 1992 | FL | Eastern | SRW | common | winter |
| Fortuna | CItr13596 | 1966 | ND | Northern | HRS | common | spring |
| Forward | CItr11392 | 1920 | NY | Eastern | SRW | common | winter |
| Forx | CItr14126 | 1964 | ND | Northern | HRS | common | spring |
| Fox | CItr13987 | 1970 | TX | Great Plains | HRW | common | winter |
| Frankenmuth | CItr17830 | 1979 | MI | Eastern | SWW | common | winter |
| Franklin | CItr15317 | 1972 | ID | PNW | HRW | common | winter |
| Freedom | PI562382 | 1991 | OH | Eastern | SRW | common | winter |
| Fremont | CItr14056 | 1970 | UT | PNW | HRS | common | spring |
| Frisco | CItr13106 | 1953 | TX | Great Plains | SRW | common | winter |
| Frontiersman | PI600832 | 1981 | KS | Great Plains | HRW | common | winter |
| Fulcaster | CItr4862 | 1886 | MD | Eastern | SRW | common | winter |
| Fulcaster 612 | CItr13715 | 1940 | TN | Eastern | SRW | common | winter |
| Fulhard | CItr8257 | 1907 | KS | Great Plains | HRW | common | winter |
| Fulhio | CItr6999 | 1920 | OH | Eastern | SRW | common | winter |
| Fulton | CItr13358 | 1964 | OH | Eastern | SRW | common | winter |
| Funk Seeds 7171 | CItr17403 | 1975 | IL | Eastern | xxW | common | winter |
| Funk Seeds 7172 | CItr17404 | 1975 | IL | Eastern | xxW | common | winter |
| Funk Seeds 7174 | CItr17405 | 1975 | IL | Eastern | xxW | common | winter |
| Funk W-332 | CItr17244 | 1972 | IL | Eastern | HRW | common | winter |
| Funk W-335 | CItr17350 | 1975 | IL | Eastern | HRW | common | winter |
| Funk W-433 | CItr17245 | 1972 | AZ | Pacific | HRS | common | spring |
| Funk W-504 | CItr17351 | 1974 | IL | Eastern | SRW | common | winter |
| Fuzz | CItr17412 | 1975 | IN | Eastern | SRW | common | winter |
| GA-Andy | PI561843 | 1990 | GA | Eastern | SRW | common | winter |
| Gage | CItr13532 | 1963 | NE | Great Plains | HRW | common | winter |
| GA-Gore | PI561842 | 1990 | GA | Eastern | SRW | common | winter |
| Gaines | CItr13448 | 1961 | WA | PNW | SWW | common | winter |
| Gasser | CItr13289 | 1955 | AK | Alaska | HRS | common | spring |
| Gasta | CItr11398 | 1931 | GA | Eastern | SRW | common | winter |
| GB 2148 | CItr595212 | 1975 | KS | Great Plains | HRW | common | winter |
| Gene | PI560129 | 1992 | OR | PNW | SWW | common | winter |
| Genro | CItr11535 | 1928 | WA | PNW | SWW | common | winter |
| Gent | CItr17293 | 1974 | SD | Northern | HRW | common | winter |
| Georgia 100 | PI538257 | 1988 | GA | Eastern | SRW | common | winter |
| Glacier | PI555586 | 1991 | WI | Eastern | SRW | common | winter |
| Glenman | PI483235 | 1985 | MT | Northern | HRS | common | spring |
| Glory | PI592750 | 1992 | OH | Eastern | SRW | common | winter |
| Glyndon | CItr25991 | 1898 | MN | Northern | HRS | common | spring |
| Golden | CItr10063 | 1930 | OR | PNW | SWW | common | winter |
| Golden 50 | CItr14190 | 1963 | KS | Great Plains | HRW | common | winter |
| Golden Cross | CItr5180 | 1888 | NY | Eastern | SRW | common | winter |
| Goldenchief | CItr14487 | 1968 | KS | Great Plains | HRW | common | winter |
| Goodstreak | PI632434 | 2002 | NE | Great Plains | HRW | common | winter |
| Goss | CItr12870 | 1949 | KS | Great Plains | HRW | common | winter |
| GR855 | PI508286 | 1985 | OH | Eastern | SRW | common | winter |
| GR876 | PI515951 | 1987 | OH | Eastern | SRW | common | winter |
| Grandin | PI531005 | 1989 | ND | Northern | HRS | common | spring |
| Grandprize | CItr4876 | 1910 | NY | Eastern | SRW | common | winter |
| Grant | PI572548 | 1993 | IN | Eastern | SRW | common | winter |
| Greer | CItr17725 | 1978 | ID | PNW | SWW | common | winter |
| Guard | PI17934 | 1983 | SD | Northern | HRS | common | spring |
| Gus | PI531004 | 1989 | ND | Northern | HRS | common | spring |
| Gypsum | CItr4762 | 1912 | CO | Great Plains | SWS | common | spring |
| Hadden | CItr13488 | 1962 | SC | Eastern | SRW | common | winter |
| Hallam | PI638790 | 2005 | NE | Great Plains | HRW | common | winter |
| Halt | PI584505 | 1994 | CO | Great Plains | HRW | common | winter |
| Hancock | PI531197 | 1988 | KS | Great Plains | SRW | common | winter |
| Hansel | CItr17296 | 1974 | UT | PNW | HRW | common | winter |
| Hard Baart | CItr8274 | 1922 | AZ | Pacific | HWS | common | spring |
| Hard Federation 31 | CItr8255 | 1928 | OR | PNW | SWS | common | spring |
| Harding | PI608049 | 1999 | CO | Great Plains | HRW | common | winter |
| Hardired | CItr12411 | 1940 | SC | Eastern | SRW | common | winter |
| Harry | PI632435 | 2002 | NE | Great Plains | HRW | common | winter |
| Hart | CItr17426 | 1976 | MO | Eastern | SRW | common | winter |
| Harvest Queen | CItr5314 | 1897 | KS | Great Plains | SRW | common | winter |
| Hatcher | PI638512 | 2004 | CO | Great Plains | HRW | common | winter |
| Hatton | CItr17772 | 1979 | WA | PNW | HRW | common | winter |
| Haven | PI547082 | 1991 | KS | Great Plains | HRW | common | winter |
| Hayden | PI608040 | 1999 | CO | Great Plains | HRW | common | winter |
| Haynes Bluestem | CItr2874 | 1895 | ND | Northern | HRS | common | spring |
| Heglar | CItr17269 | 1974 | ID | PNW | HRW | common | winter |
| Henry | CItr12265 | 1944 | WI | Eastern | HRS | common | spring |
| Hi-Line | PI549275 | 1991 | MT | Northern | HRS | common | spring |
| Hoff | PI560128 | 1992 | OR | PNW | HRW | common | winter |
| Honor | CItr6161 | 1920 | NY | Eastern | SWW | common | winter |
| Hope | CItr8178 | 1927 | SD | Northern | HRS | common | spring |
| Hosar | CItr10067 | 1930 | OR | PNW | xxW | common | winter |
| HR 53 | PI478009 | 1983 | CO | Great Plains | HRW | common | winter |
| HR 64 | PI478010 | 1983 | CO | Great Plains | HRW | common | winter |
| Hume | CItr13526 | 1965 | SD | Northern | HRW | common | winter |
| Hunter | PI468977 | 1982 | SC | Eastern | SRW | common | winter |
| Hybrid 143 | CItr4160 | 1907 | WA | PNW | SWS | common | spring |
| Hybrid 63 | CItr4510 | 1907 | WA | PNW | SWS | common | spring |
| Hymar | CItr11605 | 1935 | WA | PNW | SWW | common | winter |
| Hyper | CItr8875 | 1929 | WA | PNW | SWS | common | spring |
| Idaed | CItr11706 | 1938 | ID | PNW | SWS | common | spring |
| Idaed 59 | CItr13631 | 1962 | ID | PNW | SWS | common | spring |
| IDO644 | PI660549 | 2011 | ID | PNW | SWS | common | spring |
| IDO671 | PI660548 | 2011 | ID | PNW | SWS | common | spring |
| IDO686 | PI660551 | 2011 | ID | PNW | SWS | common | spring |
| IDO687 | PI660552 | 2011 | ID | PNW | SWS | common | spring |
| III | CItr598209 | 1974 | KS | Great Plains | HRW | common | winter |
| Illini Chief | CItr5406 | 1915 | IL | Eastern | SRW | common | winter |
| Illinois No. 2 | CItr11537 | 1931 | IL | Eastern | SRW | common | winter |
| Imbler | CItr10066 | 1930 | OR | PNW | SWW | common | winter |
| Indian | CItr4489 | 1915 | ID | PNW | SWS | common | spring |
| Ingal | CItr562647 | 1955 | AK | Alaska | HRS | common | spring |
| Inia 66R | CItr15328 | 1969 | CA | Pacific | HRS | common | spring |
| Intrada | PI631402 | 2000 | OK | Great Plains | HWW | common | winter |
| InW0731 | PI652933 | 2007 | IN | Eastern | SRW | common | winter |
| Inw8841 | PI531247 | 1989 | IN | Eastern | SRW | common | winter |
| Inw8852 | PI531248 | 1989 | IN | Eastern | SRW | common | winter |
| Inw9241 | PI583826 | 1994 | IN | Eastern | SRW | common | winter |
| Iobred | CItr6934 | 1923 | IA | Eastern | HRW | common | winter |
| Iobred 73 | CItr583669 | 1962 | IA | Eastern | HRW | common | winter |
| Iohardi | CItr12510 | 1948 | IA | Eastern | HRW | common | winter |
| Iona | PI618734 | 1999 | ID | PNW | HRS | common | spring |
| Ionia | CItr14469 | 1969 | MI | Eastern | SWW | common | winter |
| Iowin | CItr10017 | 1930 | IA | Eastern | HRW | common | winter |
| Irwin Dicklow | CItr8855 | 1912 | ID | PNW | SWS | common | spring |
| Itana | CItr12933 | 1956 | MT | Northern | HRW | common | winter |
| Jacmar | CItr608016 | 1978 | WA | PNW | SWW | common | winter |
| James | CItr17791 | 1979 | SD | Northern | HRS | common | spring |
| Jeff | CItr17270 | 1974 | ID | PNW | HRW | common | winter |
| Jefferson | PI603040 | 1998 | ID | PNW | HRS | common | spring |
| Jerry | PI632433 | 2001 | ND | Northern | HRW | common | winter |
| John | PI494095 | 1984 | WA | PNW | SWW | common | winter |
| Joleen | CItr17411 | 1975 | KS | Great Plains | HRW | common | winter |
| Jones Fife | CItr4468 | 1889 | NY | Eastern | SRW | common | winter |
| Jubilee | PI614839 | 2001 | ID | PNW | SWS | common | spring |
| Judith | PI584526 | 1989 | MT | Northern | HRW | common | winter |
| Juniper | PI639951 | 2006 | ID | PNW | HRW | common | winter |
| Justin | CItr13462 | 1962 | ND | Northern | HRS | common | spring |
| Kancom | CItr13362 | 1956 | TN | Eastern | SRW | common | winter |
| Kanhull | CItr11877 | 1935 | KS | Great Plains | HRW | common | winter |
| KanKing | CItr12719 | 1952 | KS | Great Plains | HRW | common | winter |
| Kanqueen | CItr12762 | 1949 | KS | Great Plains | SRW | common | winter |
| Kanred | CItr5146 | 1917 | KS | Great Plains | HRW | common | winter |
| Karmont | CItr6700 | 1921 | MT | Northern | HRW | common | winter |
| Kaw 61 | CItr12871 | 1960 | KS | Great Plains | HRW | common | winter |
| Kawvale | CItr8180 | 1929 | KS | Great Plains | HRW | common | winter |
| Kay | PI502970 | 1986 | IN | Eastern | SRW | common | winter |
| Kenosha | CItr14025 | 1968 | WI | Eastern | SRW | common | winter |
| Key | CItr15928 | 1976 | IN | Eastern | SRW | common | winter |
| Kiowa | CItr12133 | 1950 | KS | Great Plains | HRW | common | winter |
| Kirwin | CItr17275 | 1973 | KS | Great Plains | HRW | common | winter |
| Kitt | CItr518818 | 1975 | MN | Northern | HRS | common | spring |
| Klasic | PI486139 | 1982 | NE | Great Plains | HWS | common | spring |
| Kmor | PI536995 | 1990 | WA | PNW | SWW | common | winter |
| Knox 62 | CItr13701 | 1962 | IN | Eastern | SRW | common | winter |
| Komar | CItr8004 | 1930 | ND | Northern | HRS | common | spring |
| Krona | PI564087 | 1992 | CO | Great Plains | HRS | common | spring |
| Kruse | CItr11524 | 1957 | MT | Northern | SRW | common | winter |
| Lafron | CItr14022 | 1964 | IN | Eastern | xxW | common | winter |
| Lamar | PI559719 | 1988 | CO | Great Plains | HRW | common | winter |
| Lambert | PI583372 | 1994 | ID | PNW | SWW | common | winter |
| Lancer | CItr13547 | 1963 | NE | Great Plains | HRW | common | winter |
| Lani | CItr14021 | 1964 | IN | Eastern | xxW | common | winter |
| LaPorte | CItr12557 | 1957 | IN | Eastern | SRW | common | winter |
| Lark | CItr17338 | 1971 | CA | Pacific | HRS | common | spring |
| Leap | CItr4823 | 1907 | VA | Eastern | SRW | common | winter |
| Lee | CItr12488 | 1950 | MN | Northern | HRS | common | spring |
| Leif | PI601206 | 1985 | MN | Northern | HRS | common | spring |
| Lemhi | CItr11415 | 1939 | ID | PNW | SWS | common | spring |
| Lemhi 53 | CItr13258 | 1953 | ID | PNW | SWS | common | spring |
| Lemhi 62 | CItr13435 | 1962 | ID | PNW | SWS | common | spring |
| Lemhi 66 | CItr13969 | 1966 | ID | PNW | SWS | common | spring |
| Len | CItr17790 | 1979 | ND | Northern | HRS | common | spring |
| Lenore | CItr17726 | 1978 | ID | PNW | SWW | common | winter |
| Lew | CItr17429 | 1976 | MT | Northern | HRS | common | spring |
| Lewis | CItr13705 | 1964 | MO | Eastern | SRW | common | winter |
| Lewjain | PI17909 | 1982 | WA | PNW | SWW | common | winter |
| LHS | PI660547 | 2011 | ID | PNW | HWW | common | winter |
| Lincoln | PI506404 | 1987 | KS | Great Plains | SRW | common | winter |
| Lindon | CItr17440 | 1975 | CO | Great Plains | HRW | common | winter |
| LoLo | PI614840 | 2000 | ID | PNW | HWS | common | spring |
| Longberry No. 1 | CItr5823 | 1898 | NY | Eastern | SWW | common | winter |
| Longhorn | PI552813 | 1991 | KS | Great Plains | HRW | common | winter |
| Louise | PI634865 | 2005 | WA | PNW | SWS | common | spring |
| Lucas | CItr12990 | 1959 | OH | Eastern | SRW | common | winter |
| Luft | CItr13729 | 1962 | ID | PNW | SWW | common | winter |
| Luke | CItr14586 | 1970 | WA | PNW | SWW | common | winter |
| Lynn | PI565359 | 1991 | CA | Pacific | HWS | common | spring |
| Mace | PI651043 | 2007 | NE | Great Plains | HRW | common | winter |
| MacVicar | PI552427 | 1992 | OR | PNW | SWW | common | winter |
| Madison | PI547041 | 1990 | VA | Eastern | SRW | common | winter |
| Madsen | PI511673 | 1988 | WA | PNW | SWW | common | winter |
| Magnum | PI477285 | 1983 | CO | Great Plains | SRW | common | winter |
| Manning | CItr17846 | 1979 | UT | PNW | HRW | common | winter |
| Marberg | PI518816 | 1980 | MT | Northern | HRS | common | spring |
| Marett Chancellor | CItr12566 | 1947 | SC | Eastern | SRW | common | winter |
| Marfed | CItr11919 | 1946 | WA | PNW | SWS | common | spring |
| Maricopa | CItr14129 | 1966 | AZ | Pacific | HWS | common | spring |
| Marshall | PI17920 | 1982 | MN | Northern | HRS | common | spring |
| Martin | CItr4463 | 1880 | NY | Eastern | SWW | common | winter |
| Martin Amber | PI565360 | 1991 | NY | Eastern | SWW | common | winter |
| Marvel | CItr8876 | 1928 | SD | Northern | SRW | common | winter |
| Massey | PI17953 | 1981 | VA | Eastern | SRW | common | winter |
| Maverick | CItr17728 | 1977 | TX | Great Plains | HRW | common | winter |
| Mayview | CItr5874 | 1915 | WA | PNW | SWS | common | spring |
| McCall | CItr13842 | 1965 | WA | PNW | HRW | common | winter |
| McGuire | PI593890 | 1996 | MT | Northern | HRW | common | winter |
| McKay | PI17903 | 1981 | ID | PNW | HRS | common | spring |
| McNair 1587 | CItr17279 | 1973 | NC | Eastern | SRW | common | winter |
| McNair 1813 | CItr15289 | 1975 | NC | Eastern | SRW | common | winter |
| McNair 2203 | CItr15228 | 1970 | NC | Eastern | SRW | common | winter |
| McNeal | PI574642 | 1994 | MT | Northern | HRS | common | spring |
| Meggie | PI486337 | 1984 | CO | Great Plains | HRW | common | winter |
| Mercury | CItr11872 | 1933 | ND | Northern | HRS | common | spring |
| Meridian | PI557013 | 1992 | ID | PNW | HRW | common | winter |
| Merit | CItr11870 | 1937 | ND | Northern | HRS | common | spring |
| Merrimac | PI535454 | 1988 | WI | Eastern | SRW | common | winter |
| Mesa | PI511308 | 1987 | KS | Great Plains | HRW | common | winter |
| Michigan Amber | CItr11379 | 1875 | IN | Eastern | SRW | common | winter |
| Michikof | CItr6990 | 1920 | IN | Eastern | HRW | common | winter |
| Mida | CItr12008 | 1944 | ND | Northern | HRS | common | spring |
| Milam | CItr13369 | 1959 | TX | Great Plains | HRS | common | spring |
| Milburn | PI601252 | 1985 | TX | Great Plains | HRW | common | winter |
| Mindoro | PI601075 | 1984 | CA | Pacific | HRS | common | spring |
| MinnPro | PI532149 | 1989 | MN | Northern | HRS | common | spring |
| Minter | CItr12138 | 1948 | MN | Northern | HRW | common | winter |
| Minturki | CItr6155 | 1919 | MN | Northern | HRW | common | winter |
| Missouri Valley | CItr10046 | 1930 | ND | Northern | HRS | common | spring |
| Moking | CItr12556 | 1946 | KS | Great Plains | SRW | common | winter |
| Monon | CItr13278 | 1959 | IN | Eastern | SRW | common | winter |
| Moran | CItr13743 | 1967 | ID | PNW | HRS | common | spring |
| Mosida | CItr6688 | 1924 | ID | PNW | HRW | common | winter |
| Nabob | CItr8869 | 1928 | OH | Eastern | SRW | common | winter |
| NE01643 | PI647959 | 2007 | NE | Great Plains | HRW | common | winter |
| NE05548 | PI670462 | 2014 | NE | Great Plains | HRW | common | winter |
| NE06545 | PI667038 | 2013 | NE | Great Plains | HRW | common | winter |
| Nebraska No. 28 | CItr5147 | 1916 | NE | Great Plains | SRW | common | winter |
| Nebraska No. 6 | CItr6249 | 1919 | NE | Great Plains | HRW | common | winter |
| Nebraska No. 60 | CItr6250 | 1918 | NE | Great Plains | HRW | common | winter |
| Nebred | CItr10094 | 1938 | NE | Great Plains | HRW | common | winter |
| Neeley | PI17860 | 1980 | ID | PNW | HRW | common | winter |
| Nekota | PI584997 | 1994 | NE | Great Plains | HRW | common | winter |
| Nelson | PI17770 | 1982 | AR | Eastern | SRW | common | winter |
| New Victory | CItr12677 | 1949 | WA | PNW | SWW | common | winter |
| Newana | CItr17430 | 1976 | MT | Northern | HRS | common | spring |
| Newcaster | CItr12528 | 1946 | IL | Eastern | SRW | common | winter |
| Newest Improved Triumph | CItr13668 | 1954 | OK | Great Plains | HRW | common | winter |
| Newthatch | CItr12318 | 1944 | MN | Northern | HRS | common | spring |
| Newton | CItr17715 | 1978 | KS | Great Plains | HRW | common | winter |
| Nicoma | CItr13874 | 1971 | OK | Great Plains | HRW | common | winter |
| Niobrara | PI584996 | 1994 | NE | Great Plains | HRW | common | winter |
| Nogal | PI562646 | 1992 | AK | Alaska | HRS | common | spring |
| Norak | PI601205 | 1984 | MN | Northern | HRS | common | spring |
| Norana | CItr15927 | 1973 | MT | Northern | HRS | common | spring |
| Nordic | PI506405 | 1986 | KS | Great Plains | HRS | common | spring |
| Nordman | CItr14127 | 1964 | ND | Northern | HRS | common | spring |
| Norseman | PI494102 | 1986 | CO | Great Plains | HRS | common | spring |
| Norwin | PI491533 | 1984 | MT | Northern | HRW | common | winter |
| Nowesta | CItr17390 | 1973 | ND | Northern | HRS | common | spring |
| Nudel | CItr12672 | 1947 | DE | Eastern | SRW | common | winter |
| Nugaines | CItr13968 | 1965 | WA | PNW | SWW | common | winter |
| Nuplains | PI605741 | 1998 | NE | Great Plains | HWW | common | winter |
| Nured | CItr12455 | 1939 | NY | Eastern | SRW | common | winter |
| NY Batavia | PI595085 | 1996 | NY | Eastern | SWW | common | winter |
| Oasis | CItr15929 | 1973 | IN | Eastern | SRW | common | winter |
| Oatka Chief | CItr591889 | 1897 | NY | Eastern | SWW | common | winter |
| OK101 | PI631493 | 2001 | OK | Great Plains | HRW | common | winter |
| Olaf | CItr15930 | 1973 | ND | Northern | HRS | common | spring |
| Omaha | CItr13015 | 1960 | NE | Great Plains | HRW | common | winter |
| Omar | CItr13072 | 1955 | WA | PNW | SWW | common | winter |
| Omega 78 | CItr17721 | 1979 | GA | Eastern | SRW | common | winter |
| Onas 41 | CItr12229 | 1942 | CA | Pacific | SWS | common | spring |
| Onas 53 | CItr13069 | 1953 | CA | Pacific | SWS | common | spring |
| Oregon Zimmerman | CItr7359 | 1921 | OR | PNW | SWS | common | spring |
| Orfed | CItr11913 | 1943 | WA | PNW | SWS | common | spring |
| Orion | PI497986 | 1985 | CO | Great Plains | SRW | common | winter |
| Oro | CItr8220 | 1927 | OR | PNW | HRW | common | winter |
| Osage | CItr17292 | 1974 | OK | Great Plains | HRW | common | winter |
| Oslo | PI17901 | 1980 | CO | Great Plains | HRS | common | spring |
| Ottawa | CItr12804 | 1960 | KS | Great Plains | HRW | common | winter |
| Oveson | PI512338 | 1986 | OR | PNW | SWW | common | winter |
| Owens | PI17904 | 1981 | ID | PNW | SWS | common | spring |
| Pacer | PI614655 | 1988 | MO | Eastern | SRW | common | winter |
| Pacific Bluestem 37 | CItr11903 | 1937 | CA | Pacific | SWS | common | spring |
| Paha | CItr14485 | 1970 | WA | PNW | SWW | common | winter |
| Palala | CItr17847 | 1979 | ND | Northern | xxW | common | winter |
| Palo Duro | CItr14584 | 1969 | KS | Great Plains | HRW | common | winter |
| Parker | CItr13285 | 1966 | KS | Great Plains | HRW | common | winter |
| Payne | CItr17717 | 1977 | OK | Great Plains | HRW | common | winter |
| Peak | CItr14587 | 1971 | ID | PNW | HRS | common | spring |
| Peak 72 | CItr15319 | 1972 | ID | PNW | HRS | common | spring |
| Peck | CItr17298 | 1974 | ID | PNW | SWW | common | winter |
| Pedigreed Blackhull No. 60 | CItr8857 | 1917 | KS | Great Plains | HRW | common | winter |
| Penawawa | PI495916 | 1985 | WA | PNW | SWS | common | spring |
| Pennoll | CItr12755 | 1951 | PA | Eastern | SRW | common | winter |
| Penquite | CItr5948 | 1858 | OH | Eastern | SRW | common | winter |
| Phoenix | CItr17962 | 1981 | CA | Pacific | HWW | common | winter |
| Pilcraw | CItr5540 | 1917 | CA | Pacific | SWS | common | spring |
| Pilot | CItr11428 | 1939 | ND | Northern | HRS | common | spring |
| PL145 | PI600840 | 1982 | KS | Great Plains | HRW | common | winter |
| Plainsman | CItr14128 | 1964 | ND | Northern | HRS | common | spring |
| Polk | CItr13773 | 1968 | MN | Northern | HRS | common | spring |
| Pomerelle | PI592983 | 1996 | ID | PNW | SWS | common | spring |
| Ponca | CItr12128 | 1951 | KS | Great Plains | HRW | common | winter |
| Pondera | PI17828 | 1980 | MT | Northern | HRS | common | spring |
| Pontiac | PI573038 | 1993 | IN | Eastern | SRW | common | winter |
| Pony | PI601203 | 1985 | NE | Great Plains | HRW | common | winter |
| Portage | CItr5654 | 1923 | OH | Eastern | SRW | common | winter |
| Portola | CItr17415 | 1975 | CA | Pacific | HRS | common | spring |
| Poso | CItr8891 | 1930 | CA | Pacific | SWS | common | spring |
| Poso 41 | CItr12240 | 1941 | CA | Pacific | SWS | common | spring |
| Poso 44 | CItr12389 | 1945 | CA | Pacific | SWS | common | spring |
| Poso 48 | CItr12691 | 1948 | CA | Pacific | SWS | common | spring |
| Potomac | CItr15292 | 1975 | VA | Eastern | SRW | common | winter |
| Powerclub | CItr8276 | 1926 | ID | PNW | SWW | common | winter |
| Prairie | CItr12069 | 1943 | IL | Eastern | SRW | common | winter |
| Prairie Red | PI605390 | 1998 | CO | Great Plains | HRW | common | winter |
| Premier | CItr11940 | 1938 | ND | Northern | HRS | common | spring |
| Pride of Genesee | CItr3365 | 1893 | NY | Eastern | SRW | common | winter |
| Probred | CItr17410 | 1974 | MN | Northern | HRS | common | spring |
| Prodax | CItr17407 | 1974 | MN | Northern | HRS | common | spring |
| Profit 75 | CItr17348 | 1975 | CA | Pacific | HRS | common | spring |
| Progress | CItr6902 | 1921 | WI | Eastern | SRW | common | winter |
| Promontory | PI555458 | 1991 | UT | PNW | HRW | common | winter |
| Prospect | PI491568 | 1988 | SD | Northern | HRS | common | spring |
| Prosperity | CItr5380 | 1890 | NY | Eastern | SRW | common | winter |
| Prospur | CItr17408 | 1975 | MN | Northern | HRS | common | spring |
| Protor | CItr17409 | 1975 | MN | Northern | HRS | common | spring |
| Prowers | PI605389 | 1997 | CO | Great Plains | HRW | common | winter |
| Prowers 99 | PI612420 | 1999 | CO | Great Plains | HRW | common | winter |
| Purcam | CItr12294 | 1951 | IN | Eastern | SRW | common | winter |
| Purcell | PI17787 | 1981 | NY | Eastern | SWW | common | winter |
| Purplestraw | PI500000 | 1985 | MD | Eastern | SRW | common | winter |
| Quality | CItr6607 | 1918 | CA | Pacific | HWS | common | spring |
| R36 | CItr583674 | 1974 | IL | Eastern | HRW | common | winter |
| Racine | CItr13172 | 1956 | WI | Eastern | SRW | common | winter |
| Raeder | CItr17418 | 1976 | WA | PNW | SWW | common | winter |
| Ram | PI477287 | 1983 | CO | Great Plains | HRW | common | winter |
| Ramona | CItr8241 | 1935 | CA | Pacific | SWS | common | spring |
| Ramona 50 | CItr12390 | 1951 | CA | Pacific | HWS | common | spring |
| Rampart | PI593889 | 1996 | MT | Northern | HRW | common | winter |
| Ranger | CItr15316 | 1972 | ID | PNW | HRW | common | winter |
| Rawhide | PI543893 | 1990 | NE | Great Plains | HRW | common | winter |
| Read | CItr6401 | 1898 | VT | Eastern | SWW | common | winter |
| Red Chief | CItr12109 | 1940 | KS | Great Plains | HRW | common | winter |
| Red Clawson | CItr565369 | 1889 | NY | Eastern | SRW | common | winter |
| Red Jacket | CItr12713 | 1950 | KS | Great Plains | HRW | common | winter |
| Red River 68 | CItr14193 | 1968 | CA | Pacific | HRS | common | spring |
| Red Rock | CItr5597 | 1914 | MI | Eastern | SRW | common | winter |
| Red Wave | CItr3500 | 1905 | NY | Eastern | SRW | common | winter |
| Redhart | CItr8898 | 1921 | SC | Eastern | SRW | common | winter |
| Redhart 4 | CItr12003 | 1949 | SC | Eastern | SRW | common | winter |
| Redhart 5 | CItr12004 | 1949 | SC | Eastern | SRW | common | winter |
| Redhull | CItr11534 | 1921 | KS | Great Plains | HRW | common | winter |
| Redland | PI502907 | 1986 | NE | Great Plains | HRW | common | winter |
| Redwin | CItr17844 | 1979 | MT | Northern | HRW | common | winter |
| Regal | CItr7364 | 1926 | OR | PNW | HRS | common | spring |
| Regenerated Defiance | CItr3703 | 1907 | CO | Great Plains | HWS | common | spring |
| Rego | CItr13181 | 1957 | MT | Northern | HRW | common | winter |
| Reliance | CItr7370 | 1926 | OR | PNW | HRS | common | spring |
| Reliant | CItr12144 | 1940 | OK | Great Plains | HRW | common | winter |
| Rely | PI542401 | 1991 | WA | PNW | SWW | common | winter |
| Rew | CItr17294 | 1974 | OR | PNW | SWW | common | winter |
| Rex | CItr10065 | 1933 | OR | PNW | SWW | common | winter |
| RHS 8232 | PI601138 | 1984 | NC | Eastern | SRW | common | winter |
| Richland | PI632399 | 2001 | NY | Eastern | SWW | common | winter |
| Rick | PI614834 | 1985 | UT | PNW | HRS | common | spring |
| Ridit | CItr6703 | 1923 | WA | PNW | HRW | common | winter |
| Riley | CItr13702 | 1965 | IN | Eastern | SRW | common | winter |
| Riley 67 | CItr14110 | 1967 | IN | Eastern | SRW | common | winter |
| Rio | CItr10061 | 1930 | OR | PNW | HRW | common | winter |
| Rio Blanco | PI531244 | 1989 | KS | Great Plains | HWW | common | winter |
| Rival | CItr11708 | 1939 | ND | Northern | HRS | common | spring |
| Rocky | CItr17879 | 1978 | CO | Great Plains | HRW | common | winter |
| Rod | PI558510 | 1992 | WA | PNW | SWW | common | winter |
| Rodco | CItr13560 | 1957 | KS | Great Plains | HRW | common | winter |
| Roedel | CItr15175 | 1963 | OR | PNW | SWW | common | winter |
| Rohde | PI562529 | 1993 | OR | PNW | SWW | common | winter |
| Roland | CItr17716 | 1977 | IL | Eastern | SRW | common | winter |
| Rose | PI17795 | 1981 | SD | Northern | HRW | common | winter |
| Rosen | PI17607 | 1980 | AR | Eastern | SRW | common | winter |
| Rosetta | CItr13363 | 1959 | OK | Great Plains | HRW | common | winter |
| Roughrider | CItr17439 | 1975 | ND | Northern | HRW | common | winter |
| RSI 220 | PI584322 | 1994 | CA | Pacific | SRW | common | winter |
| Ruddy | CItr6465 | 1919 | WA | PNW | SRW | common | winter |
| Rulo | PI578137 | 1994 | WA | PNW | SWW | common | winter |
| Rural New Yorker No. 57 | CItr3516 | 1894 | NY | Eastern | SRW | common | winter |
| Rural New Yorker No. 6 | CItr5921 | 1894 | NY | Eastern | SRW | common | winter |
| Rushmore | CItr12273 | 1949 | SD | Northern | HRS | common | spring |
| Russell | CItr12484 | 1956 | WI | Eastern | HRS | common | spring |
| S 76 | CItr17608 | 1976 | KS | Great Plains | SRW | common | winter |
| S78 | CItr600823 | 1978 | IN | Eastern | SRW | common | winter |
| Saline | CItr12674 | 1950 | IL | Eastern | SRW | common | winter |
| Salmon | PI542976 | 1990 | ID | PNW | SWW | common | winter |
| Saluda | PI480474 | 1983 | VA | Eastern | SRW | common | winter |
| Sanford | CItr12026 | 1940 | GA | Eastern | SRW | common | winter |
| Satanta | CItr14582 | 1969 | KS | Great Plains | HRW | common | winter |
| Sawmont | CItr13544 | 1965 | MT | Northern | HRW | common | winter |
| Sawtana | CItr13304 | 1961 | MT | Northern | HRS | common | spring |
| Sawtell | CItr17424 | 1977 | ID | PNW | HRS | common | spring |
| Sawyer | PI559930 | 1991 | CO | Great Plains | SRW | common | winter |
| Scout | CItr13546 | 1964 | NE | Great Plains | HRW | common | winter |
| Scoutland | CItr14075 | 1970 | NE | Great Plains | HRW | common | winter |
| Seabreeze | CItr12611 | 1945 | TX | Great Plains | SRW | common | winter |
| Sentinel | CItr17265 | 1973 | NE | Great Plains | HRW | common | winter |
| Severn | PI17939 | 1981 | MD | Eastern | SRW | common | winter |
| Sharp | PI540401 | 1990 | SD | Northern | HRS | common | spring |
| Shasta | CItr17651 | 1976 | CA | Pacific | HRS | common | spring |
| Shepherd | CItr6163 | 1923 | NY | Eastern | SRW | common | winter |
| Sheridan | CItr13586 | 1966 | MT | Northern | HRS | common | spring |
| Sherman | CItr4430 | 1928 | OR | PNW | HRW | common | winter |
| Shield | PI491570 | 1986 | SD | Northern | HRS | common | spring |
| Shortana | CItr15233 | 1971 | MT | Northern | HRS | common | spring |
| Sibley 81 | CItr10084 | 1930 | OK | Great Plains | HRW | common | winter |
| SilverSheaf | CItr2496 | 1905 | NY | Eastern | SRW | common | winter |
| Sioux | CItr12142 | 1940 | NE | Great Plains | HRW | common | winter |
| Siouxland | PI483469 | 1984 | NE | Great Plains | HRW | common | winter |
| Solar | CItr486140 | 1978 | NE | Great Plains | HRS | common | spring |
| Southern Belle | PI17894 | 1980 | SC | Eastern | SRW | common | winter |
| Spillman | PI506350 | 1987 | WA | PNW | HRS | common | spring |
| Spinkcota | CItr12375 | 1944 | SD | Northern | HRS | common | spring |
| Spokane Chief | CItr13252 | 1953 | WA | PNW | SRW | common | winter |
| SpringField | CItr14589 | 1970 | ID | PNW | SWS | common | spring |
| SR-49 | PI487294 | 1984 | CO | Great Plains | SRW | common | winter |
| Stacy | PI17861 | 1980 | GA | Eastern | SRW | common | winter |
| Stadler | CItr13704 | 1964 | MO | Eastern | SRW | common | winter |
| Stafford | CItr12706 | 1949 | KS | Great Plains | HRW | common | winter |
| Steele | PI516196 | 1988 | IN | Eastern | SRW | common | winter |
| Sterling | PI17859 | 1980 | ID | PNW | SWS | common | spring |
| Stoa | PI520297 | 1984 | ND | Northern | HRS | common | spring |
| Stoddard | CItr15925 | 1973 | MO | Eastern | SRW | common | winter |
| Sturgeon | CItr11703 | 1934 | WI | Eastern | HRS | common | spring |
| Sullivan | CItr17684 | 1977 | IN | Eastern | SRW | common | winter |
| Super Triumph | CItr13669 | 1957 | OK | Great Plains | HRW | common | winter |
| Surprise | CItr25971 | 1909 | CA | Pacific | SWS | common | spring |
| Survivor | PI509503 | 1991 | ID | PNW | HRW | common | winter |
| Susquehanna | PI474581 | 1988 | MD | Eastern | SRW | common | winter |
| Sylvan | PI578867 | 1994 | CO | Great Plains | HRS | common | spring |
| Symphony | PI583754 | 1994 | WA | PNW | SRW | common | winter |
| Syringa | PI601452 | 1988 | ID | PNW | SWW | common | winter |
| TAM 106 | CItr17827 | 1979 | TX | Great Plains | HRW | common | winter |
| TAM 109 | PI554606 | 1991 | TX | Great Plains | HRW | common | winter |
| TAM 202 | PI561933 | 1992 | TX | Great Plains | HRW | common | winter |
| TAM 300 | PI576151 | 1993 | TX | Great Plains | HRW | common | winter |
| TAM W-101 | CItr15324 | 1971 | TX | Great Plains | HRW | common | winter |
| TAM W-104 | CItr17452 | 1975 | TX | Great Plains | HRW | common | winter |
| TAM-200 | PI578255 | 1986 | TX | Great Plains | HRW | common | winter |
| Tammy | PI601288 | 1986 | CA | Pacific | HRS | common | spring |
| Tayland | CItr12761 | 1954 | MD | Eastern | SRW | common | winter |
| Taylor | CItr12461 | 1953 | VA | Eastern | SRW | common | winter |
| Taylor 49 | CItr13249 | 1956 | NC | Eastern | SRW | common | winter |
| Tecumseh | CItr17287 | 1973 | MI | Eastern | SWW | common | winter |
| Telemark | PI506406 | 1987 | KS | Great Plains | HRS | common | spring |
| Tendoy | CItr13426 | 1960 | ID | PNW | HRW | common | winter |
| Tenmarq | CItr6936 | 1932 | KS | Great Plains | HRW | common | winter |
| Terral 877 | PI564083 | 1992 | LA | Eastern | SRW | common | winter |
| Teton | CItr15244 | 1971 | MT | Northern | HRW | common | winter |
| Texred | CItr17729 | 1977 | TX | Great Plains | HRW | common | winter |
| Thatcher | CItr10003 | 1934 | MN | Northern | HRS | common | spring |
| Thunderbird | PI497988 | 1985 | CO | Great Plains | HRW | common | winter |
| Tiber | PI517194 | 1988 | MT | Northern | HRW | common | winter |
| Ticonderoga | CItr17290 | 1973 | NY | Eastern | SWW | common | winter |
| Timstein | CItr12347 | 1939 | MN | Northern | xxW | common | winter |
| Timwin | CItr13787 | 1967 | WI | Eastern | SRW | common | winter |
| Tioga | CItr17286 | 1974 | ND | Northern | HRS | common | spring |
| Todd | CItr13110 | 1956 | KY | Eastern | SRW | common | winter |
| Tracey | CItr17925 | 1979 | CA | Pacific | HRS | common | spring |
| Trailblazer | PI506407 | 1987 | KS | Great Plains | HRW | common | winter |
| Trapper | CItr13999 | 1967 | NE | Great Plains | HRW | common | winter |
| Treasure | PI468962 | 1986 | ID | PNW | SWS | common | spring |
| Triplet | CItr5408 | 1918 | WA | PNW | SRW | common | winter |
| Trison | CItr17278 | 1973 | KS | Great Plains | HRW | common | winter |
| Triumph | CItr12132 | 1940 | OK | Great Plains | HRW | common | winter |
| Trumbull | CItr5657 | 1916 | OH | Eastern | SRW | common | winter |
| Twain | PI506408 | 1987 | KS | Great Plains | SRW | common | winter |
| Twin | CItr14588 | 1971 | ID | PNW | SWS | common | spring |
| Tyee | PI17773 | 1979 | WA | PNW | SWW | common | winter |
| Tyler | PI17899 | 1980 | VA | Eastern | SRW | common | winter |
| UI Lochsa | PI639952 | 2005 | ID | PNW | HWS | common | spring |
| UI Pettit | PI642363 | 2006 | ID | PNW | SWS | common | spring |
| UI Winchester | PI642362 | 2009 | ID | PNW | HRS | common | spring |
| Union | CItr11704 | 1921 | OR | PNW | SWS | common | spring |
| Urquie | CItr17413 | 1975 | WA | PNW | SWS | common | spring |
| USU-Apogee | PI592742 | 1996 | UT | PNW | HRS | common | spring |
| Utac | CItr10045 | 1928 | UT | PNW | HWS | common | spring |
| Utah-100 | PI594920 | 1996 | UT | PNW | HRW | common | winter |
| Ute | PI490017 | 1983 | UT | PNW | HRW | common | winter |
| V.P.I. 112 | CItr11397 | 1915 | VA | Eastern | SRW | common | winter |
| V.P.I. 131 | CItr10047 | 1915 | VA | Eastern | SRW | common | winter |
| Vahart | CItr12537 | 1945 | VA | Eastern | SRW | common | winter |
| Valprize | CItr11539 | 1931 | NY | Eastern | SRW | common | winter |
| Vance | PI532150 | 1989 | MN | Northern | HRS | common | spring |
| Vandal | PI546056 | 1991 | ID | PNW | HRS | common | spring |
| Vanguard | PI593891 | 1995 | MT | Northern | HRW | common | winter |
| Vermillion | CItr12748 | 1955 | IN | Eastern | SRW | common | winter |
| Vesta | CItr11712 | 1942 | ND | Northern | HRS | common | spring |
| Victory | PI497989 | 1985 | CO | Great Plains | HRW | common | winter |
| Vigal | PI545492 | 1990 | AK | Alaska | HRS | common | spring |
| Vigo | CItr12220 | 1946 | IN | Eastern | SRW | common | winter |
| Virginia | CItr565372 | 1910 | MD | Eastern | SRW | common | winter |
| Vista | PI562653 | 1992 | NE | Great Plains | HRW | common | winter |
| Voyager | PI546478 | 1991 | KS | Great Plains | HRW | common | winter |
| W2501 | PI516200 | 1988 | CO | Great Plains | HRS | common | spring |
| W2502 | PI516201 | 1988 | CO | Great Plains | HRS | common | spring |
| Waban | CItr12992 | 1978 | IN | Eastern | SRW | common | winter |
| Waco | PI531246 | 1989 | KS | Great Plains | HRW | common | winter |
| Wadual | PI506354 | 1987 | WA | PNW | SWS | common | spring |
| Wadual 94 | PI566595 | 1993 | WA | PNW | SWS | common | spring |
| Wahoo | PI619098 | 2000 | NE | Great Plains | HRW | common | winter |
| Wakanz | PI506352 | 1987 | WA | PNW | SWS | common | spring |
| Wakeland | CItr13382 | 1959 | NC | Eastern | SRW | common | winter |
| Waldron | CItr13958 | 1969 | ND | Northern | HRS | common | spring |
| Walera | PI486141 | 1982 | NE | Great Plains | HRS | common | spring |
| Wampum | PI17691 | 1980 | WA | PNW | HRS | common | spring |
| Wanken | CItr13659 | 1961 | OK | Great Plains | xxW | common | winter |
| Wanser | CItr13844 | 1965 | WA | PNW | HRW | common | winter |
| Wared | CItr15926 | 1974 | WA | PNW | HRS | common | spring |
| Warrior | CItr13190 | 1960 | NE | Great Plains | HRW | common | winter |
| Wasatch | CItr11925 | 1944 | UT | PNW | HRW | common | winter |
| Wawawai | PI574538 | 1994 | WA | PNW | SWS | common | spring |
| Wesel | CItr13090 | 1944 | OK | Great Plains | HRW | common | winter |
| Westar | CItr12110 | 1944 | TX | Great Plains | HRW | common | winter |
| Westbred 906R | PI483455 | 1981 | AZ | Pacific | HRS | common | spring |
| Westbred 911 | PI483456 | 1981 | AZ | Pacific | HRS | common | spring |
| Westbred Aim | PI599989 | 1979 | AZ | Pacific | HRS | common | spring |
| Westbred Challenger | PI601072 | 1983 | ND | Northern | HRS | common | spring |
| Westmont | CItr12930 | 1956 | MT | Northern | HRW | common | winter |
| Weston | CItr17727 | 1978 | ID | PNW | HRW | common | winter |
| Wheaton | PI469271 | 1983 | MN | Northern | HRS | common | spring |
| Wheeler | PI17900 | 1980 | VA | Eastern | SRW | common | winter |
| White Federation 38 | CItr11906 | 1939 | CA | Pacific | SWS | common | spring |
| White Federation 59 | CItr17579 | 1976 | CA | Pacific | SWS | common | spring |
| White Federation 60 | CItr17580 | 1976 | CA | Pacific | SWS | common | spring |
| White Mediterranean | CItr11489 | 1930 | TX | Great Plains | SWW | common | winter |
| White Odessa | CItr4655 | 1928 | ID | PNW | SWW | common | winter |
| Whitebird | PI592982 | 1996 | ID | PNW | SWS | common | spring |
| Wichita | CItr11952 | 1944 | KS | Great Plains | HRW | common | winter |
| Wilbur | CItr6797 | 1919 | OR | PNW | SWS | common | spring |
| Willett | CItr13099 | 1935 | MN | Northern | xxW | common | winter |
| Williams | PI508082 | 1984 | SC | Eastern | SRW | common | winter |
| Willow Creek | PI655073 | 2005 | MT | Northern | HRW | common | winter |
| Windstar | PI597379 | 1996 | NE | Great Plains | HRW | common | winter |
| Wings | CItr17880 | 1977 | CO | Great Plains | HRW | common | winter |
| Winoka | CItr14000 | 1969 | SD | Northern | HRW | common | winter |
| Winridge | PI17902 | 1981 | MT | Northern | HRW | common | winter |
| Wisconsin Pedigree No. 2 | CItr6683 | 1918 | WI | Eastern | HRW | common | winter |
| Wissler | CItr11610 | 1933 | ND | Northern | xxW | common | winter |
| World Seeds 1 | CItr17347 | 1974 | CA | Pacific | HWS | common | spring |
| World Seeds 13 | PI17824 | 1979 | CA | Pacific | SWS | common | spring |
| World Seeds 1651 | CItr15334 | 1969 | CA | Pacific | HRS | common | spring |
| World Seeds 1809 | CItr15012 | 1971 | CA | Pacific | HRS | common | spring |
| World Seeds 1812 | CItr14585 | 1969 | CA | Pacific | HRS | common | spring |
| World Seeds 1877 | CItr14591 | 1969 | CA | Pacific | HRS | common | spring |
| World Seeds 25 | CItr17825 | 1976 | CA | Pacific | HRS | common | spring |
| World Seeds 6 | CItr17345 | 1973 | CA | Pacific | HRS | common | spring |
| Wrangler | PI477288 | 1983 | CO | Great Plains | HRW | common | winter |
| Yamhill | CItr14563 | 1969 | OR | PNW | SWW | common | winter |
| Yecora Rojo | CItr17414 | 1975 | CA | Pacific | HRS | common | spring |
| Yogo | CItr8033 | 1932 | MT | Northern | HRW | common | winter |
| Yolo | PI17961 | 1981 | CA | Pacific | HRS | common | spring |
| Yorkstar | CItr14026 | 1968 | NY | Eastern | SWW | common | winter |
| Yorkwin | CItr11855 | 1935 | NY | Eastern | SWW | common | winter |
| Yukon | CItr14583 | 1969 | KS | Great Plains | HRW | common | winter |
| Yumar | PI605388 | 1998 | CO | Great Plains | HRW | common | winter |

Supplemental Table S2. Number of varieties and variety release years in populations used for F_st_, Rsb, and xpEHH calculations.

| **Habit** | **Population** | **Varieties** | **Release Years** | **First variety** | **Last variety** |
| --- | --- | --- | --- | --- | --- |
| spring | all | 236 | 1878–2011 | Early Defiance | IDO644 |
| spring | CA | 48 | 1878–1991 | Early Defiance | Lynn |
| spring | CA_Other | 171 | 1895–2011 | Haynes Bluestem | IDO644 |
| spring | gpl | 26 | 1907–1994 | Regenerated Defiance | Sylvan |
| spring | gpl_Other | 210 | 1878–2011 | Early Defiance | IDO644 |
| spring | HRS | 150 | 1895–2009 | Haynes Bluestem | UI Winchester |
| spring | HRS_Other | 86 | 1878–2011 | Early Defiance | IDO644 |
| spring | HRS1 | 75 | 1895–1978 | Haynes Bluestem | Eureka |
| spring | HRS2 | 75 | 1978–2009 | Angus | UI Winchester |
| spring | ID | 41 | 1912–2011 | Irwin Dicklow | IDO644 |
| spring | ID_Other | 152 | 1878–1996 | Early Defiance | BacUp |
| spring | MN | 23 | 1898–1996 | Glyndon | BacUp |
| spring | MN_Other | 163 | 1878–2011 | Early Defiance | IDO644 |
| spring | ND | 29 | 1895–1989 | Haynes Bluestem | Grandin |
| spring | ND_Other | 163 | 1878–2011 | Early Defiance | IDO644 |
| spring | nor | 73 | 1895–1996 | Haynes Bluestem | BacUp |
| spring | nor_Other | 163 | 1878–2011 | Early Defiance | IDO644 |
| spring | nor1 | 36 | 1895–1974 | Haynes Bluestem | Ellar |
| spring | nor2 | 37 | 1974–1996 | Probred | BacUp |
| spring | pac | 58 | 1878–1994 | Early Defiance | Admire |
| spring | pac_Other | 178 | 1895–2011 | Haynes Bluestem | IDO644 |
| spring | pac1 | 29 | 1878–1969 | Early Defiance | Inia 66R |
| spring | pac2 | 29 | 1971–1994 | Anza | Admire |
| spring | pnw | 72 | 1907–2011 | Hybrid 143 | IDO644 |
| spring | pnw_Other | 164 | 1878–1996 | Early Defiance | BacUp |
| spring | pnw1 | 36 | 1907–1977 | Hybrid 143 | Fieldwin |
| spring | pnw2 | 36 | 1978–2011 | Dirkwin | IDO644 |
| spring | spr1 | 118 | 1878–1975 | Early Defiance | Profit 75 |
| spring | spr2 | 118 | 1975–2011 | Kitt | IDO644 |
| spring | SWS | 70 | 1878–2011 | Early Defiance | IDO644 |
| spring | SWS_Other | 166 | 1895–2009 | Haynes Bluestem | UI Winchester |
| spring | SWS1 | 35 | 1878–1962 | Early Defiance | Lemhi 62 |
| spring | SWS2 | 35 | 1962–2011 | Idaed 59 | IDO644 |
| spring | WA | 20 | 1907–2005 | Hybrid 143 | Louise |
| spring | WA_Other | 164 | 1878–1996 | Early Defiance | BacUp |
| winter | all | 517 | 1858–2014 | Penquite | NE05548 |
| winter | CO | 33 | 1916–2004 | Blount's Lambrigg | Hatcher |
| winter | CO_Other | 324 | 1858–2013 | Penquite | ARS-Selbu |
| winter | eas | 202 | 1858–2007 | Penquite | InW0731 |
| winter | eas_Other | 315 | 1897–2014 | Harvest Queen | NE05548 |
| winter | eas1 | 101 | 1858–1968 | Penquite | Kenosha |
| winter | eas2 | 101 | 1968–2007 | Arthur | InW0731 |
| winter | gpl | 185 | 1897–2014 | Harvest Queen | NE05548 |
| winter | gpl_Other | 332 | 1858–2013 | Penquite | ARS-Selbu |
| winter | gpl1 | 92 | 1897–1978 | Harvest Queen | Capitan |
| winter | gpl2 | 93 | 1978–2014 | Bennett | NE05548 |
| winter | HRW | 232 | 1907–2014 | Fulhard | NE05548 |
| winter | HRW_Other | 285 | 1858–2013 | Penquite | ARS-Selbu |
| winter | HRW1 | 116 | 1907–1976 | Fulhard | Eklund |
| winter | HRW2 | 116 | 1977–2014 | Maverick | NE05548 |
| winter | ID | 23 | 1924–2011 | Mosida | LHS |
| winter | ID_Other | 406 | 1858–2014 | Penquite | NE05548 |
| winter | IN | 48 | 1875–2007 | Michigan Amber | InW0731 |
| winter | IN_Other | 315 | 1897–2014 | Harvest Queen | NE05548 |
| winter | KS | 74 | 1897–1991 | Harvest Queen | Haven |
| winter | KS_Other | 314 | 1858–2013 | Penquite | ARS-Selbu |
| winter | NE | 39 | 1916–2014 | Nebraska No. 28 | NE05548 |
| winter | NE_Other | 320 | 1858–2013 | Penquite | ARS-Selbu |
| winter | nor | 35 | 1919–2006 | Minturki | Alice |
| winter | nor_Other | 482 | 1858–2014 | Penquite | NE05548 |
| winter | NY | 27 | 1880–2001 | Martin | Richland |
| winter | NY_Other | 315 | 1897–2014 | Harvest Queen | NE05548 |
| winter | OR | 20 | 1927–1993 | Oro | Rohde |
| winter | OR_Other | 422 | 1858–2014 | Penquite | NE05548 |
| winter | pnw | 93 | 1918–2013 | Triplet | ARS-Selbu |
| winter | pnw_Other | 424 | 1858–2014 | Penquite | NE05548 |
| winter | pnw1 | 46 | 1918–1974 | Triplet | Hansel |
| winter | pnw2 | 47 | 1976–2013 | Barbee | ARS-Selbu |
| winter | SC | 21 | 1921–1994 | Redhart | Clemson 201 |
| winter | SC_Other | 315 | 1897–2014 | Harvest Queen | NE05548 |
| winter | SRW | 188 | 1858–2007 | Penquite | InW0731 |
| winter | SRW_Other | 329 | 1880–2014 | Martin | NE05548 |
| winter | SRW1 | 94 | 1858–1968 | Penquite | Kenosha |
| winter | SRW2 | 94 | 1968–2007 | Arthur | InW0731 |
| winter | SWW | 74 | 1880–2013 | Martin | ARS-Selbu |
| winter | SWW_Other | 443 | 1858–2014 | Penquite | NE05548 |
| winter | SWW1 | 37 | 1880–1974 | Martin | Rew |
| winter | SWW2 | 37 | 1974–2013 | Peck | ARS-Selbu |
| winter | WA | 42 | 1918–2013 | Triplet | ARS-Selbu |
| winter | WA_Other | 424 | 1858–2014 | Penquite | NE05548 |
| winter | win1 | 258 | 1858–1974 | Penquite | Bronze |
| winter | win2 | 259 | 1974–2014 | III | NE05548 |

Supplemental Table S3. Sequences for known informative markers and their physical positions on ‘Chinese Spring’ wheat reference genome version 2.1.

| **Chr** | **Start Position** | **Locus Name** | **Function** | **Sequence** |
| --- | --- | --- | --- | --- |
| 1A | 253484442 | 1RS_1AL | Wheat / rye translocation | ACCCTGATCCTTCAAACAAGATAACCGCTRCAGATTCTGTCATGACCTCAAGAGACATCAATGTACTGGGTAGC |
| 1A | 510221495 | Glu_A1 | Glu-A1 | GAATACCTGCACTACTAAGAAGGTATTACCTAAGTGTAACTTCTCCGCAACRGGTTTCATACTATCCAGGCCA |
| 1A | 510221438 | Glu_A1 | Glu-A1 | GAATACCTGCACTACTAAGAAGGTATTACCTAAGTGTAACTTCTCCGCAACRGGTTTCATACTATCCAGGCCA |
| 1B | 151769885 | 1RS_1BL | Wheat / rye translocation | GGACAGATCACCGTCAGGACAAGCACATCGGAGCAGGTCCAGATCGCRCTGCTGGGTCTNGTCCCAGTGATAGCCTCCATCTACCGGAGCTTCCGGCCCGGTGAACCATCGTTTGCTTATTAGCTTGTCAAG |
| 1B | 562453626 | Glu_B1 | Glu-B1 | GCAACAGTCAAGACAAAGGCAACAGGCAGGACAATGGCAACGACCGGGACAAGGGCAAYCAGGGTACTACCCAACCTCTCC |
| 1D | 414600319 | Glu_D1 | Glu-D1 | GGCCTGGATAGTATGAAACCTGCTGCGGASAAGTTACACTTGGGTAATACCTTTTTAGTAGTGCAGGTATTCCCCAAAATATACGTTGTTGGAGTTGCTGCGGT |
| 2A | 5814621 | Lr37 | Rust resistant gene | CGTTTGCTCATGCTAGAAAGCATCTGTAAGCAGGCTTTKGTGTACATACTGCTGTAGCTGCAGTCTGTGAAGCTTAGAG |
| 2A | 41525233 | Ppd_A1 | Photoperiod response | GTTCGGCCAAATCTTTACTAAAACACATCAACATCTATACAATGCTAAAGTCGCACATTATGAASCCCCTTTTAGCCGCCGGCCGCTCCGCTCCATTCGAGGACACGATTCATTCCCG |
| 2A | 41525083 | Ppd_A1 | Photoperiod response | GTTCGGCCAAATCTTTACTAAAACACATCAACATCTATACAATGCTAAAGTCGCACATTATGAASCCCCTTTTAGCCGCCGGCCGCTCCGCTCCATTCGAGGACACGATTCATTCCCG |
| 2A | 715938219 | Ppo_A1 | Polyphenol oxidase | TGTTCGTATGGTCAATGGGTTATGGATGGGACGACCTGCACCTTTCTGWGCTGAACGTCAAGGAGCCGTCACTTGTCCCTGCGTGGGTTTGCTGAACGTGCAGGTTCTACCTCTA |
| 2B | 705140521 | Yr5_IWA4096 | stripe rust resistance | TCCCCACTTTTGTACCACTGCCCAGCCTGTACACCCRAAGGAAATGACACATTTACAGCTCAGATCAAAGTTCTCATTCTTGTCACCAGGGA |
| 2B | 700891511 | Yr5_IWA6121 | stripe rust resistance | TGGCATACAGACTGAACTGGGCCAGTGCTGGTGAAAAGCGTGAYGATACCCCGGACTATACAATCTTTGTTGGGGATTTGGCAG |
| 2B | 208668139 | Phs_TaSdr_B1 | Pre-harvest sprouting | CCACGCCTTCTTAAGCCGGCACGGCACCGGGACCCAACGCCGTGCACTCCGTCCACCCCCGTCAGCAGACTTCGACTCGCRCGTGCACGCAATGGCCATGGTGCAGCCGGCGGACATGGCCGTCAAGGCCAACGAGATC |
| 2B | 125556679 | Sr36 | Stem rust resistance | CCTCGATGGGCACATTTTTGTCGATGCGGTGCTGGTAGTAGAGATCAATACAGTCGACACCCAGCCGCTTGAGGCKGCCCTCGCAAGCCGCGCGCACTACGCTGGATCCCCCTGGATGTTCCACTTGTTGTCTGCG |
| 2B | 179147685 | Tgw_TaSus2 | Thousand kernel weight | TCACTGCTGAGTACAATGCCGCGATCCCTGAGGCCGAGCGYGAGAAGCTCAAGGACACCGCCTTTGAGGATCTCCTAAG |
| 2B | 63367106 | Ppd_B1 | Photoperiod response | GCTCGTTCTCCAATCAAGGCGGGGCAGGGTTCGTCGGGAGCTGTTCGCCCAMCGGCAACAGCTCCAAGGCCGCGAAAACGGACGCCGCTCAGATGAAGCAAGGCTCAA |
| 2D | 36208368 | Ppd_D1 | Photoperiod response | GCTACCACCAGTGTTAATTAATTTGTACTGGCTCGGTAGTCATGGAGGTAYTAAATTTGGGCGTTGGTTGTGTGTCATGTTCAG |
| 2D | 36206278 | Ppd_D1 | Photoperiod response | GCTACCACCAGTGTTAATTAATTTGTACTGGCTCGGTAGTCATGGAGGTAYTAAATTTGGGCGTTGGTTGTGTGTCATGTTCAG |
| 2D | 36210757 | Ppd_D1a | Photoperiod response | ATCTAGTGATCCCCAACTCCCACCGACGCCTCCCACTACACTGGGCTTCCACCAWCCGCTGCTCATACTTCCTTGCCGCCT |
| 2D | 575922602 | Ppo_D1 | Polyphenol oxidase | CAATATCCCAAGAGACCAGCAGATCGATSAGAACCTCAAGATCATGTACCGCCAGG |
| 3A | 704036455 | Ssc_Tamby10_A1 | Seed coat color | TTGCTCTTCGCCGAACATTGCATCAATACATACATTTATCGATTATGCGYGCGATTTTCTCTTAGTTGACCGATCTCTCC |
| 3A | 7364396 | Phs_TaPHS1 | Pre-harvest sprouting | TTGTGTCTGCAGGATAGTGGTTAACATACCGGGTGGAACAGATGCAACTAAARTTAGTACTTTATTTATGACZAATGGATCATTAGTTTCATATAACACTCACAATACATTGTACTCCAATTAACCATTCACATGTAAATTGCAC |
| 3B | 13956406 | Fhb_1snp3 | Fusarium head blight resistance | TTGCCCAAGATTGTAGTCTGCCATTATAATGCACATGCATTTGCAAGGTTGTTATCSTTGTGTTGATATGAAAATTTCAAACTATTGACCTAAGGCTGCTTTGTTTCAGAGTTACAAATATGTTATCTTGTGATGCAAAC |
| 3B | 773231639 | Scc_Tamby10_B1 | Seed coat color | GCTCCAACCACCCACAGGCCAAGCCGCCGSCGGCGAGGCGCACGCCCTGCCCGAGGGCACGAGCAGCAGCCCGATACGGACCAAGGCG |
| 3B | 93810710 | Dr_TaDreb_B1 | Drought tolerance | CACCTTGTGATATGGATTGCCTTGATGAMCAGGAAGAAGAAAGTGCGCAGGAGAAGCACTGGTCCTGATTCGGTTGCTGAAA |
| 3D | 572162512 | Scc_Tamyb10_D1 | Seed coat color | AGGCCAACACCTTCTAAACGTATATAACTTCTAATCRATATGATCMAGTTATCATGATCACCTGTACACACCTACTATCC |
| 4A | 603753330 | Phs4A_34562_92 | Pre-harvest sprouting | GCTTCTGTCCCTTACTTTGAGGATTCAGAATTAAGCTCTGTTTTTGCCTCCGTCTGCCAGAACTTGGAGTCTGAAAGCATTCGRCTCTATTAAATTCAGGGTATTTTTATTGTCTGAATATTTGATTTGTGTTTTCCTATGATGCATGGA |
| 4A | 604565933 | Phs4A_34586_92 | Pre-harvest sprouting | TCGTTGATCGCGCACAATGCCGGAGTCGAAGGGGAGGTGATCGTGGAKAAAATCAAGGACAGCGAGTGGGAATTCGGCTACAACGCGATGACCGACAAGCACGAGAA |
| 4A | 603120393 | Phs4A_3743_9 | Pre-harvest sprouting | CAACAGGTATTGTACCGACAAGGTCGTTATCATTGAGGAATAGGAAGCTGAGTTGAGTCAGTTTGGCCAACCATGTYGGAACAACACCTTCAAATGAGTTCTCGCCAAGGGAAAGAGTTTGGAGGTATGGACAAGATGCAAAGCCCAATG |
| 4B | 30022343 | Lox_B1 | Lipoxygenase | ACGTCTACAACGACCTCGGCGAGGGCCGCCCCGTCCTCGGCGGCAGCGCCGASCACCCCTATCCGCGCCGCGGCCGCACCGGGCGCAAGCCCAACGCCAACGACCCGAGCTTGGAGAGCCGGCTGTCGCTGCTGGAGCAGATCTACGTG |
| 4B | 33614690 | Rht_B1 | Dwarfing gene | ATGGCGGACGTGGCGCAGAAGCTGGAGYAGCTNGAGATGGCCATGGGGATGGG |
| 4B | 33613872 | Rht_B1 | Dwarfing gene | ATGGCGGACGTGGCGCAGAAGCTGGAGYAGCTNGAGATGGCCATGGGGATGGG |
| 4B | 33614174 | Rht_B1 | Dwarfing gene | ATGGCGGACGTGGCGCAGAAGCTGGAGYAGCTNGAGATGGCCATGGGGATGGG |
| 4D | 19189816 | Rht_D1 | Dwarfing gene | ATGGCGGACGTGGCGCAGAAGCTGKAGNAGCTCGAGATGGCCATGGGGATGGG |
| 5A | 590394554 | Vrn_A1a | Vernalization requirement | CCTGTTAGTAGTGATGGTCCAATAATGCCAAACTACAGTTTTRTTTACATTGATCTATCTTTTTGGAAAACTCTGATCTTACTTTGGTGTGCTCTGG |
| 5B | 71691327 | Vrn_B1b | Vernalization requirement | CTTACAGAAATGTAGGGAAAGGCTGCGTACTATAGACCCAAAGTGGTCGGACCCTTCCCCGACCCTGCGCAAGCGGGAGCTACATSCACCACCCCCTTTGTTGTTCATGGC |
| 5B | 577021139 | Vrn_B1c | Vernalization requirement | TCCACAGGCTTTCCTATCATTCGTATTGCTAGCTCCGGCCATGCGGCCATTTTGTTGCTCCGGCCWGACTACCC |
| 5D | 3622934 | Pinb_D1 | Puroindoline/kernel hardness | GTCACCTGGCCCACAAAATGGTGGAAGRGCGGCTGTGAGCATGAGGTTCGGGAGAAGTGCTGCAAGCAGCTGAGCCAGATAGCACCACAAT |
| 5D | 470048974 | Vrn_D1 | Vernalization requirement | TTCCACAGGCTTTGCTATCATTCGAATTGCTAGCTCCGSCTAGGCACCATTCTTTCCGCCTTAGGC |
| 6B | 62263492 | 1_FEH_w3 | Auxin response element | CTATACATCCTATCGCTCTCCTCCCCCTCCCCCCTTCCTTCTGTCYCCGAGGCCCAAAGCTCGGGCCGTCTTCCTCCTCCTCCTCATCTTCTTCTACAGGAGC |
| 6B | 140409103 | Gpc_B1 | Grain protein content | TTCACATGTCTCCAAGAGGGGAGAGACATGTTACTTWATTTGAGTCTATGT |
| 7B | 751981946 | Psy_B1 | Phytoene synthase | AGACACAGTTGGTGAAGAATAAAGGCCTCAYATAACACTTT |
| 7D | 48955823 | Lr34 | Slow rusting gene | GGAATACTGTTAGAGCAAGAAAAAGGTAGAAGAAAGACACAATAGGTGTTTGGAAGTATGAAGCAATAAATCGATRCTGCGAGGTGACTGAATGATGGAGAAGACAAAGTATCAGAAACTGG |
| 7D | 637698675 | Psy1_D1 | Phytoene synthase | TTCAAGGACATGATCGACGGGATGCGGACGGACCTYAAGAAGGCNAGNTACAAGAACTTNGACGAGCTCTACATGTACTG |
| 7D | 6833106 | Tgw_TaGS_D1 | Thousand grain weight | TGAAGGCAACACAGATTTTAGTTAAAACCTCTAAATAGAAGTTTCGCGTTTTAGCCAAGAAATGTCGCTCTCAKTGGAGATTTATACAATAATACTCCCTCCGTCC |

Supplemental Table S4. Candidate selective sweeps in U.S. wheat populations. Also available additional columns as /output/ cr_pic_freq_consolidated.csv at https://datadryad.org/stash/share/62lamvCW7tXg4-O-xY0beBGR7CODFUwSQK2aTjXTjV8.

| **cssn** | **chr** | **startMBp** | **endMBp** | **sizeKB** | **details** | **stat** | **habit** | **pops** | **n_extr_mrk** | **max_mrk** | **mean_extr_mrk** |
| --- | --- | --- | --- | --- | --- | --- | --- | --- | --- | --- | --- |
| css1 | 1A | 23 | 40 | 17000 | ID (Rsb 4.85, 42/45) | Rsb | win | ID | 42 | 4.852 | 3.476 |
| css2 | 1A | 25 | 31 | 6000 | IN (xpEHH 2.61, 21/22) | xpEHH | win | IN | 21 | 2.609 | 2.325 |
| css3 | 1A | 25 | 32 | 7000 | ND (Rsb 3.42, 6/6) | Rsb | spr | ND | 6 | 3.418 | 2.706 |
| css4 | 1A | 25 | 31 | 6000 | pnw2 (Rsb 5.02, 22/22) | Rsb | win | pnw2 | 22 | 5.023 | 3.613 |
| css5 | 1A | 26 | 38 | 12000 | IN (xpEHH 2.84, 35/36) | xpEHH | both | IN | 35 | 2.839 | 2.337 |
| css6 | 1A | 26 | 36 | 10000 | ND (Rsb 3.70, 30/31) | Rsb | both | ND | 30 | 3.701 | 2.694 |
| css7 | 1A | 26 | 36 | 10000 | ND (xpEHH 3.33, 30/31) | xpEHH | spr | ND | 30 | 3.334 | 2.788 |
| css8 | 1A | 26 | 42 | 16000 | TX (Rsb 4.53, 44/45) | Rsb | both | TX | 44 | 4.530 | 3.302 |
| css9 | 1A | 27 | 43 | 16000 | TX (xpEHH 4.17, 42/42) | xpEHH | both | TX | 42 | 4.171 | 2.973 |
| css10 | 1A | 28 | 54 | 26000 | NE (xpEHH 4.88, 63/66) | xpEHH | win | NE | 63 | 4.883 | 3.048 |
| css11 | 1A | 30 | 54 | 24000 | gpl (Rsb 5.82, 57/60) | Rsb | both | gpl | 57 | 5.816 | 3.491 |
| css12 | 1A | 30 | 54 | 24000 | gpl (Rsb 6.26, 57/60) | Rsb | win | gpl | 57 | 6.258 | 3.282 |
| css13 | 1A | 30 | 54 | 24000 | HRW (Rsb 5.86, 57/60) | Rsb | win | HRW | 57 | 5.857 | 3.627 |
| css14 | 1A | 30 | 40 | 10000 | ID (xpEHH 3.84, 24/26) | xpEHH | win | ID | 24 | 3.841 | 2.958 |
| css15 | 1A | 30 | 35 | 5000 | ND (xpEHH 2.44, 9/10) | xpEHH | both | ND | 9 | 2.439 | 2.196 |
| css16 | 1A | 30 | 46 | 16000 | NE (Rsb 5.47, 34/34) | Rsb | both | NE | 34 | 5.472 | 3.947 |
| css17 | 1A | 30 | 44 | 14000 | NE (Rsb 5.57, 32/32) | Rsb | win | NE | 32 | 5.566 | 3.748 |
| css18 | 1A | 30 | 54 | 24000 | NE (xpEHH 4.98, 58/60) | xpEHH | both | NE | 58 | 4.984 | 3.347 |
| css19 | 1A | 31 | 37 | 6000 | CO (Rsb 3.60, 15/16) | Rsb | win | CO | 15 | 3.603 | 2.978 |
| css20 | 1A | 31 | 38 | 7000 | CO (Rsb 4.53, 17/17) | Rsb | both | CO | 17 | 4.534 | 3.362 |
| css21 | 1A | 31 | 37 | 6000 | CO (xpEHH 2.91, 15/16) | xpEHH | win | CO | 15 | 2.910 | 2.560 |
| css22 | 1A | 31 | 54 | 23000 | gpl (xpEHH 4.98, 53/55) | xpEHH | both | gpl | 53 | 4.977 | 3.538 |
| css23 | 1A | 31 | 54 | 23000 | gpl (xpEHH 5.03, 53/55) | xpEHH | win | gpl | 53 | 5.032 | 3.444 |
| css24 | 1A | 31 | 54 | 23000 | HRW (xpEHH 4.60, 53/55) | xpEHH | win | HRW | 53 | 4.604 | 3.731 |
| css25 | 1A | 31 | 37 | 6000 | ID (Rsb 4.73, 13/14) | Rsb | spr | ID | 13 | 4.726 | 2.991 |
| css26 | 1A | 31 | 38 | 7000 | ID (xpEHH 3.55, 14/15) | xpEHH | spr | ID | 14 | 3.548 | 3.160 |
| css27 | 1A | 32 | 37 | 5000 | CO (xpEHH 3.47, 13/14) | xpEHH | both | CO | 13 | 3.466 | 3.126 |
| css28 | 1A | 32 | 40 | 8000 | SWS (xpEHH 3.14, 17/18) | xpEHH | spr | SWS | 17 | 3.138 | 2.563 |
| css29 | 1A | 39 | 44 | 5000 | MN (Fst 0.42, 5/5) | Fst | spr | MN | 5 | 0.420 | 0.318 |
| css30 | 1A | 39 | 51 | 12000 | nor (xpEHH 2.73, 18/18) | xpEHH | both | nor | 18 | 2.733 | 2.245 |
| css31 | 1A | 44 | 54 | 10000 | CO (xpEHH 2.56, 25/27) | xpEHH | both | CO | 25 | 2.559 | 2.344 |
| css32 | 1A | 44 | 53 | 9000 | TX (xpEHH 2.92, 24/26) | xpEHH | both | TX | 24 | 2.920 | 2.647 |
| css33 | 1A | 74 | 80 | 6000 | HRS (Fst 0.49, 5/5) | Fst | spr | HRS | 5 | 0.494 | 0.457 |
| css34 | 1A | 90 | 96 | 6000 | HRS (Fst 0.49, 5/5) | Fst | spr | HRS | 5 | 0.494 | 0.483 |
| css35 | 1A | 100 | 113 | 13000 | HRS (Fst 0.48, 14/14) | Fst | spr | HRS | 14 | 0.477 | 0.452 |
| css36 | 1A | 107 | 113 | 6000 | CA (Fst 0.37, 6/6) | Fst | both | CA | 6 | 0.367 | 0.350 |
| css37 | 1A | 119 | 125 | 6000 | CA (Fst 0.35, 5/5) | Fst | both | CA | 5 | 0.353 | 0.338 |
| css38 | 1A | 119 | 125 | 6000 | HRS (Fst 0.38, 5/5) | Fst | spr | HRS | 5 | 0.379 | 0.379 |
| css39 | 1A | 119 | 125 | 6000 | pac2 (Fst 0.43, 5/5) | Fst | both | pac2 | 5 | 0.430 | 0.430 |
| css40 | 1A | 119 | 125 | 6000 | pac2 (Fst 0.45, 5/5) | Fst | spr | pac2 | 5 | 0.452 | 0.452 |
| css41 | 1A | 130 | 142 | 12000 | CA (Fst 0.37, 13/13) | Fst | both | CA | 13 | 0.367 | 0.352 |
| css42 | 1A | 130 | 142 | 12000 | HRS (Fst 0.45, 13/13) | Fst | spr | HRS | 13 | 0.450 | 0.436 |
| css43 | 1A | 153 | 162 | 9000 | CA (Fst 0.40, 9/9) | Fst | both | CA | 9 | 0.401 | 0.363 |
| css44 | 1A | 153 | 162 | 9000 | HRS (Fst 0.45, 9/9) | Fst | spr | HRS | 9 | 0.452 | 0.451 |
| css45 | 1A | 163 | 170 | 7000 | CA (Fst 0.37, 5/5) | Fst | both | CA | 5 | 0.367 | 0.346 |
| css46 | 1A | 163 | 170 | 7000 | HRS (Fst 0.45, 5/5) | Fst | spr | HRS | 5 | 0.450 | 0.448 |
| css47 | 1A | 233 | 238 | 5000 | CA (Fst 0.38, 5/5) | Fst | both | CA | 5 | 0.381 | 0.348 |
| css48 | 1A | 233 | 238 | 5000 | HRS (Fst 0.52, 5/5) | Fst | spr | HRS | 5 | 0.518 | 0.464 |
| css49 | 1A | 252 | 259 | 7000 | CA (Fst 0.37, 7/7) | Fst | both | CA | 7 | 0.366 | 0.360 |
| css50 | 1A | 252 | 261 | 9000 | HRS (Fst 0.43, 9/9) | Fst | spr | HRS | 9 | 0.427 | 0.424 |
| css51 | 1A | 252 | 261 | 9000 | pac2 (Fst 0.48, 9/9) | Fst | both | pac2 | 9 | 0.479 | 0.474 |
| css52 | 1A | 252 | 261 | 9000 | pac2 (Fst 0.50, 9/9) | Fst | spr | pac2 | 9 | 0.502 | 0.497 |
| css53 | 1A | 295 | 305 | 10000 | HRS (Fst 0.57, 17/17) | Fst | spr | HRS | 17 | 0.573 | 0.450 |
| css54 | 1A | 295 | 302 | 7000 | pac2 (Fst 0.52, 8/8) | Fst | both | pac2 | 8 | 0.520 | 0.509 |
| css55 | 1A | 295 | 314 | 19000 | pac2 (Fst 0.54, 34/34) | Fst | spr | pac2 | 34 | 0.545 | 0.470 |
| css56 | 1A | 300 | 305 | 5000 | SWS (Fst 0.35, 9/10) | Fst | spr | SWS | 9 | 0.352 | 0.344 |
| css57 | 1A | 335 | 362 | 27000 | OR (Fst 0.42, 55/57) | Fst | win | OR | 55 | 0.419 | 0.343 |
| css58 | 1A | 335 | 371 | 36000 | SWW (Fst 0.39, 76/78) | Fst | win | SWW | 76 | 0.395 | 0.293 |
| css59 | 1A | 345 | 352 | 7000 | nor (Fst 0.25, 14/14) | Fst | spr | nor | 14 | 0.251 | 0.232 |
| css60 | 1A | 345 | 352 | 7000 | pnw (Fst 0.19, 14/14) | Fst | both | pnw | 14 | 0.192 | 0.162 |
| css61 | 1A | 356 | 362 | 6000 | pnw (Fst 0.18, 16/17) | Fst | both | pnw | 16 | 0.175 | 0.140 |
| css62 | 1A | 366 | 371 | 5000 | OR (Fst 0.39, 11/11) | Fst | win | OR | 11 | 0.388 | 0.332 |
| css63 | 1A | 372 | 377 | 5000 | ND (Fst 0.36, 5/5) | Fst | spr | ND | 5 | 0.358 | 0.358 |
| css64 | 1A | 372 | 377 | 5000 | nor (Fst 0.22, 5/5) | Fst | spr | nor | 5 | 0.223 | 0.223 |
| css65 | 1A | 378 | 388 | 10000 | ND (Fst 0.39, 23/23) | Fst | spr | ND | 23 | 0.388 | 0.368 |
| css66 | 1A | 378 | 388 | 10000 | nor (Fst 0.24, 23/23) | Fst | spr | nor | 23 | 0.238 | 0.224 |
| css67 | 1A | 430 | 441 | 11000 | pnw (Fst 0.21, 12/12) | Fst | spr | pnw | 12 | 0.210 | 0.184 |
| css68 | 1A | 430 | 441 | 11000 | WA (Fst 0.38, 12/12) | Fst | spr | WA | 12 | 0.377 | 0.302 |
| css69 | 1A | 462 | 519 | 57000 | NE (xpEHH 4.15, 166/167) | xpEHH | both | NE | 166 | 4.148 | 3.010 |
| css70 | 1A | 463 | 489 | 26000 | gpl (xpEHH 3.44, 69/70) | xpEHH | both | gpl | 69 | 3.440 | 2.657 |
| css71 | 1A | 465 | 510 | 45000 | gpl (Rsb 5.02, 137/141) | Rsb | both | gpl | 137 | 5.024 | 3.060 |
| css72 | 1A | 465 | 476 | 11000 | nor (xpEHH 2.46, 17/17) | xpEHH | win | nor | 17 | 2.457 | 2.339 |
| css73 | 1A | 469 | 481 | 12000 | CO (xpEHH 2.55, 17/17) | xpEHH | both | CO | 17 | 2.552 | 2.351 |
| css74 | 1A | 469 | 481 | 12000 | gpl (xpEHH 2.77, 17/17) | xpEHH | win | gpl | 17 | 2.773 | 2.521 |
| css75 | 1A | 469 | 478 | 9000 | KS (xpEHH 2.53, 13/14) | xpEHH | both | KS | 13 | 2.534 | 2.356 |
| css76 | 1A | 469 | 478 | 9000 | MN (xpEHH 2.22, 13/14) | xpEHH | spr | MN | 13 | 2.224 | 2.165 |
| css77 | 1A | 469 | 478 | 9000 | NE (xpEHH 2.54, 13/14) | xpEHH | win | NE | 13 | 2.542 | 2.416 |
| css78 | 1A | 473 | 478 | 5000 | gpl (xpEHH 2.13, 11/12) | xpEHH | spr | gpl | 11 | 2.128 | 2.092 |
| css79 | 1A | 473 | 481 | 8000 | SC (Fst 0.68, 14/15) | Fst | both | SC | 14 | 0.680 | 0.523 |
| css80 | 1A | 474 | 489 | 15000 | all2 (xpEHH 2.75, 54/54) | xpEHH | both | all2 | 54 | 2.749 | 2.348 |
| css81 | 1A | 474 | 493 | 19000 | NE (Rsb 3.93, 57/61) | Rsb | both | NE | 57 | 3.930 | 2.755 |
| css82 | 1A | 474 | 481 | 7000 | SC (Fst 0.73, 11/11) | Fst | win | SC | 11 | 0.734 | 0.592 |
| css83 | 1A | 476 | 492 | 16000 | HRS (xpEHH 2.85, 54/55) | xpEHH | spr | HRS | 54 | 2.847 | 2.481 |
| css84 | 1A | 476 | 482 | 6000 | WA (Fst 0.33, 8/8) | Fst | spr | WA | 8 | 0.326 | 0.260 |
| css85 | 1A | 479 | 491 | 12000 | eas2 (xpEHH 2.62, 48/49) | xpEHH | win | eas2 | 48 | 2.625 | 2.331 |
| css86 | 1A | 479 | 491 | 12000 | eas2 (xpEHH 2.76, 48/49) | xpEHH | both | eas2 | 48 | 2.759 | 2.438 |
| css87 | 1A | 479 | 486 | 7000 | MN (xpEHH 2.54, 30/30) | xpEHH | spr | MN | 30 | 2.539 | 2.260 |
| css88 | 1A | 479 | 488 | 9000 | NE (xpEHH 2.58, 39/41) | xpEHH | win | NE | 39 | 2.578 | 2.194 |
| css89 | 1A | 479 | 492 | 13000 | nor2 (xpEHH 3.25, 47/48) | xpEHH | both | nor2 | 47 | 3.249 | 2.693 |
| css90 | 1A | 479 | 486 | 7000 | OR (xpEHH 2.47, 28/30) | xpEHH | win | OR | 28 | 2.467 | 2.280 |
| css91 | 1A | 479 | 491 | 12000 | SRW2 (xpEHH 2.50, 48/49) | xpEHH | win | SRW2 | 48 | 2.503 | 2.256 |
| css92 | 1A | 479 | 491 | 12000 | SWW (xpEHH 2.85, 47/49) | xpEHH | win | SWW | 47 | 2.848 | 2.424 |
| css93 | 1A | 480 | 486 | 6000 | CO (Rsb 2.94, 27/29) | Rsb | both | CO | 27 | 2.940 | 2.443 |
| css94 | 1A | 490 | 499 | 9000 | SWW (Rsb 3.11, 10/10) | Rsb | win | SWW | 10 | 3.105 | 2.462 |
| css95 | 1A | 492 | 517 | 25000 | eas2 (xpEHH 4.34, 90/91) | xpEHH | win | eas2 | 90 | 4.338 | 3.424 |
| css96 | 1A | 492 | 517 | 25000 | eas2 (xpEHH 4.41, 90/91) | xpEHH | both | eas2 | 90 | 4.407 | 3.397 |
| css97 | 1A | 492 | 502 | 10000 | gpl (xpEHH 2.97, 20/20) | xpEHH | win | gpl | 20 | 2.972 | 2.605 |
| css98 | 1A | 492 | 506 | 14000 | gpl (xpEHH 4.13, 47/48) | xpEHH | both | gpl | 47 | 4.133 | 2.982 |
| css99 | 1A | 492 | 510 | 18000 | MN (xpEHH 4.17, 65/65) | xpEHH | spr | MN | 65 | 4.168 | 3.180 |
| css100 | 1A | 492 | 510 | 18000 | NE (xpEHH 3.42, 67/67) | xpEHH | win | NE | 67 | 3.416 | 2.802 |
| css101 | 1A | 492 | 519 | 27000 | SRW2 (xpEHH 4.26, 94/94) | xpEHH | win | SRW2 | 94 | 4.264 | 3.435 |
| css102 | 1A | 495 | 519 | 24000 | NE (Rsb 5.11, 88/88) | Rsb | both | NE | 88 | 5.114 | 3.275 |
| css103 | 1A | 496 | 519 | 23000 | CO (Rsb 4.69, 79/83) | Rsb | both | CO | 79 | 4.690 | 2.977 |
| css104 | 1A | 496 | 502 | 6000 | eas2 (Rsb 4.19, 12/12) | Rsb | both | eas2 | 12 | 4.185 | 2.842 |
| css105 | 1A | 496 | 508 | 12000 | gpl (xpEHH 3.50, 43/45) | xpEHH | spr | gpl | 43 | 3.501 | 2.825 |
| css106 | 1A | 496 | 501 | 5000 | HRS (Rsb 3.58, 9/10) | Rsb | spr | HRS | 9 | 3.579 | 2.649 |
| css107 | 1A | 496 | 515 | 19000 | MN (Rsb 4.22, 67/69) | Rsb | spr | MN | 67 | 4.217 | 2.895 |
| css108 | 1A | 496 | 506 | 10000 | SC (xpEHH 3.35, 35/37) | xpEHH | win | SC | 35 | 3.353 | 2.769 |
| css109 | 1A | 496 | 506 | 10000 | SC (xpEHH 3.42, 35/37) | xpEHH | both | SC | 35 | 3.419 | 2.981 |
| css110 | 1A | 497 | 507 | 10000 | CO (xpEHH 3.51, 41/43) | xpEHH | both | CO | 41 | 3.512 | 2.860 |
| css111 | 1A | 497 | 506 | 9000 | gpl (Rsb 4.26, 35/39) | Rsb | win | gpl | 35 | 4.263 | 3.047 |
| css112 | 1A | 497 | 506 | 9000 | HRS (xpEHH 2.73, 36/38) | xpEHH | spr | HRS | 36 | 2.726 | 2.424 |
| css113 | 1A | 497 | 510 | 13000 | IN (xpEHH 3.15, 57/58) | xpEHH | both | IN | 57 | 3.148 | 2.654 |
| css114 | 1A | 497 | 506 | 9000 | MN (xpEHH 3.14, 37/39) | xpEHH | both | MN | 37 | 3.140 | 2.635 |
| css115 | 1A | 497 | 519 | 22000 | SRW2 (Rsb 5.37, 84/85) | Rsb | win | SRW2 | 84 | 5.368 | 3.129 |
| css116 | 1A | 498 | 510 | 12000 | IN (xpEHH 3.07, 56/58) | xpEHH | win | IN | 56 | 3.072 | 2.591 |
| css117 | 1A | 500 | 514 | 14000 | NE (Rsb 4.65, 59/60) | Rsb | win | NE | 59 | 4.653 | 2.951 |
| css118 | 1A | 500 | 506 | 6000 | SC (Rsb 4.25, 27/28) | Rsb | win | SC | 27 | 4.250 | 2.912 |
| css119 | 1A | 500 | 506 | 6000 | SC (Rsb 4.40, 28/28) | Rsb | both | SC | 28 | 4.396 | 3.231 |
| css120 | 1A | 501 | 507 | 6000 | CO (Rsb 3.38, 31/34) | Rsb | win | CO | 31 | 3.383 | 2.472 |
| css121 | 1A | 501 | 513 | 12000 | IN (Rsb 3.02, 13/13) | Rsb | both | IN | 13 | 3.016 | 2.530 |
| css122 | 1A | 503 | 512 | 9000 | gpl (Rsb 3.49, 39/41) | Rsb | spr | gpl | 39 | 3.489 | 2.726 |
| css123 | 1A | 503 | 508 | 5000 | MN (Rsb 3.43, 26/28) | Rsb | both | MN | 26 | 3.430 | 2.797 |
| css124 | 1A | 504 | 519 | 15000 | eas2 (Rsb 5.43, 61/64) | Rsb | win | eas2 | 61 | 5.433 | 3.188 |
| css125 | 1A | 504 | 519 | 15000 | eas2 (Rsb 5.47, 61/64) | Rsb | both | eas2 | 61 | 5.474 | 3.173 |
| css126 | 1A | 505 | 513 | 8000 | IN (Rsb 3.41, 10/10) | Rsb | win | IN | 10 | 3.414 | 2.635 |
| css127 | 1A | 511 | 518 | 7000 | NE (xpEHH 2.82, 14/14) | xpEHH | win | NE | 14 | 2.823 | 2.599 |
| css128 | 1A | 513 | 524 | 11000 | MN (xpEHH 3.18, 23/25) | xpEHH | spr | MN | 23 | 3.179 | 2.583 |
| css129 | 1A | 515 | 523 | 8000 | IN (Rsb 2.52, 7/7) | Rsb | both | IN | 7 | 2.523 | 2.405 |
| css130 | 1A | 515 | 524 | 9000 | IN (Rsb 2.71, 8/8) | Rsb | win | IN | 8 | 2.711 | 2.550 |
| css131 | 1A | 516 | 521 | 5000 | ID (Rsb 3.21, 10/11) | Rsb | win | ID | 10 | 3.207 | 2.819 |
| css132 | 1A | 517 | 526 | 9000 | IN (xpEHH 2.67, 18/20) | xpEHH | win | IN | 18 | 2.671 | 2.403 |
| css133 | 1A | 517 | 526 | 9000 | IN (xpEHH 2.70, 18/20) | xpEHH | both | IN | 18 | 2.700 | 2.264 |
| css134 | 1A | 529 | 534 | 5000 | IN (xpEHH 3.54, 18/20) | xpEHH | win | IN | 18 | 3.544 | 2.720 |
| css135 | 1A | 530 | 536 | 6000 | IN (Rsb 4.17, 10/11) | Rsb | win | IN | 10 | 4.168 | 2.408 |
| css136 | 1A | 530 | 539 | 9000 | TX (Rsb 4.68, 36/39) | Rsb | both | TX | 36 | 4.684 | 2.846 |
| css137 | 1A | 531 | 538 | 7000 | MN (Rsb 4.16, 30/32) | Rsb | spr | MN | 30 | 4.160 | 2.637 |
| css138 | 1A | 532 | 537 | 5000 | ID (Rsb 5.35, 18/20) | Rsb | win | ID | 18 | 5.350 | 3.096 |
| css139 | 1A | 532 | 538 | 6000 | MN (xpEHH 3.94, 26/26) | xpEHH | spr | MN | 26 | 3.942 | 2.705 |
| css140 | 1A | 534 | 539 | 5000 | TX (xpEHH 4.44, 23/23) | xpEHH | both | TX | 23 | 4.445 | 2.905 |
| css141 | 1A | 542 | 558 | 16000 | pnw (xpEHH 4.56, 72/72) | xpEHH | spr | pnw | 72 | 4.562 | 3.069 |
| css142 | 1A | 542 | 547 | 5000 | SRW (Rsb 3.06, 23/24) | Rsb | win | SRW | 23 | 3.065 | 2.459 |
| css143 | 1A | 542 | 576 | 34000 | SRW (xpEHH 3.29, 114/117) | xpEHH | win | SRW | 114 | 3.289 | 2.497 |
| css144 | 1A | 542 | 558 | 16000 | SWS (xpEHH 4.42, 72/72) | xpEHH | spr | SWS | 72 | 4.416 | 3.157 |
| css145 | 1A | 543 | 556 | 13000 | SWS (Rsb 3.79, 65/69) | Rsb | spr | SWS | 65 | 3.787 | 2.838 |
| css146 | 1A | 544 | 576 | 32000 | eas (xpEHH 3.08, 102/107) | xpEHH | win | eas | 102 | 3.077 | 2.389 |
| css147 | 1A | 544 | 578 | 34000 | eas (xpEHH 3.26, 111/111) | xpEHH | both | eas | 111 | 3.261 | 2.623 |
| css148 | 1A | 544 | 556 | 12000 | pnw (Rsb 3.59, 59/60) | Rsb | spr | pnw | 59 | 3.589 | 2.684 |
| css149 | 1A | 547 | 556 | 9000 | ID (xpEHH 3.46, 44/46) | xpEHH | spr | ID | 44 | 3.457 | 2.703 |
| css150 | 1A | 548 | 556 | 8000 | WA (xpEHH 3.65, 34/36) | xpEHH | spr | WA | 34 | 3.654 | 2.614 |
| css151 | 1A | 571 | 580 | 9000 | pnw (xpEHH 2.59, 30/32) | xpEHH | spr | pnw | 30 | 2.591 | 2.411 |
| css152 | 1A | 571 | 580 | 9000 | SWS (xpEHH 2.85, 30/32) | xpEHH | spr | SWS | 30 | 2.845 | 2.523 |
| css153 | 1A | 573 | 578 | 5000 | eas (Rsb 2.64, 13/14) | Rsb | both | eas | 13 | 2.642 | 2.210 |
| css154 | 1B | 62 | 67 | 5000 | pnw (Fst 0.25, 14/15) | Fst | win | pnw | 14 | 0.245 | 0.197 |
| css155 | 1B | 62 | 67 | 5000 | pnw (Rsb 3.34, 14/15) | Rsb | win | pnw | 14 | 3.345 | 3.082 |
| css156 | 1B | 64 | 69 | 5000 | ID (Rsb 2.93, 9/10) | Rsb | win | ID | 9 | 2.926 | 2.659 |
| css157 | 1B | 284 | 293 | 9000 | CO (Fst 0.15, 11/12) | Fst | both | CO | 11 | 0.146 | 0.146 |
| css158 | 1B | 284 | 293 | 9000 | CO (Fst 0.29, 11/12) | Fst | win | CO | 11 | 0.287 | 0.287 |
| css159 | 1B | 284 | 293 | 9000 | ND (Fst 0.62, 11/12) | Fst | spr | ND | 11 | 0.616 | 0.616 |
| css160 | 1B | 284 | 293 | 9000 | NE (Fst 0.24, 11/12) | Fst | both | NE | 11 | 0.245 | 0.245 |
| css161 | 1B | 284 | 293 | 9000 | NE (Fst 0.32, 11/12) | Fst | win | NE | 11 | 0.322 | 0.322 |
| css162 | 1B | 284 | 293 | 9000 | nor (Fst 0.24, 11/12) | Fst | spr | nor | 11 | 0.239 | 0.239 |
| css163 | 1B | 370 | 379 | 9000 | pnw2 (Fst 0.23, 10/10) | Fst | both | pnw2 | 10 | 0.226 | 0.203 |
| css164 | 1B | 370 | 379 | 9000 | pnw2 (Fst 0.24, 10/10) | Fst | win | pnw2 | 10 | 0.237 | 0.212 |
| css165 | 1B | 370 | 379 | 9000 | SWW2 (Fst 0.19, 10/10) | Fst | win | SWW2 | 10 | 0.187 | 0.187 |
| css166 | 1B | 371 | 379 | 8000 | all2 (Fst 0.10, 7/7) | Fst | both | all2 | 7 | 0.103 | 0.097 |
| css167 | 1B | 371 | 376 | 5000 | HRS2 (Fst 0.18, 5/5) | Fst | spr | HRS2 | 5 | 0.182 | 0.182 |
| css168 | 1B | 371 | 376 | 5000 | spr2 (Fst 0.20, 5/5) | Fst | spr | spr2 | 5 | 0.204 | 0.197 |
| css169 | 1B | 386 | 395 | 9000 | pnw2 (Fst 0.24, 8/8) | Fst | win | pnw2 | 8 | 0.237 | 0.218 |
| css170 | 1B | 386 | 395 | 9000 | SWW2 (Fst 0.19, 8/8) | Fst | win | SWW2 | 8 | 0.187 | 0.187 |
| css171 | 1B | 390 | 395 | 5000 | pnw2 (Fst 0.16, 5/5) | Fst | both | pnw2 | 5 | 0.164 | 0.156 |
| css172 | 1B | 460 | 466 | 6000 | nor (Fst 0.23, 14/15) | Fst | win | nor | 14 | 0.229 | 0.215 |
| css173 | 1B | 463 | 471 | 8000 | CA (Fst 0.28, 12/12) | Fst | spr | CA | 12 | 0.280 | 0.258 |
| css174 | 1B | 463 | 471 | 8000 | pac (Fst 0.26, 12/12) | Fst | spr | pac | 12 | 0.262 | 0.246 |
| css175 | 1B | 464 | 471 | 7000 | CA (Fst 0.52, 8/8) | Fst | both | CA | 8 | 0.518 | 0.518 |
| css176 | 1B | 464 | 471 | 7000 | pac (Fst 0.51, 8/8) | Fst | both | pac | 8 | 0.507 | 0.507 |
| css177 | 1B | 487 | 494 | 7000 | SWW2 (Fst 0.36, 23/25) | Fst | win | SWW2 | 23 | 0.365 | 0.267 |
| css178 | 1B | 489 | 494 | 5000 | win2 (Rsb 2.61, 14/15) | Rsb | win | win2 | 14 | 2.613 | 2.319 |
| css179 | 1B | 546 | 551 | 5000 | CA (Rsb 3.30, 21/23) | Rsb | spr | CA | 21 | 3.298 | 2.718 |
| css180 | 1B | 546 | 565 | 19000 | pac (Rsb 3.63, 66/73) | Rsb | spr | pac | 66 | 3.633 | 2.757 |
| css181 | 1B | 571 | 579 | 8000 | pac (Rsb 3.86, 40/44) | Rsb | spr | pac | 40 | 3.859 | 2.723 |
| css182 | 1B | 585 | 591 | 6000 | CA (Rsb 2.60, 6/6) | Rsb | spr | CA | 6 | 2.603 | 2.278 |
| css183 | 1B | 585 | 591 | 6000 | pac (Rsb 3.20, 6/6) | Rsb | spr | pac | 6 | 3.204 | 2.528 |
| css184 | 1B | 626 | 633 | 7000 | ND (Rsb 3.67, 16/17) | Rsb | spr | ND | 16 | 3.668 | 2.789 |
| css185 | 1B | 630 | 654 | 24000 | ND (xpEHH 3.23, 131/133) | xpEHH | spr | ND | 131 | 3.231 | 2.629 |
| css186 | 1B | 634 | 641 | 7000 | ND (xpEHH 3.37, 39/39) | xpEHH | both | ND | 39 | 3.369 | 2.552 |
| css187 | 1B | 635 | 654 | 19000 | ND (Rsb 3.72, 75/79) | Rsb | spr | ND | 75 | 3.718 | 2.560 |
| css188 | 1B | 635 | 643 | 8000 | ND (Rsb 4.43, 40/41) | Rsb | both | ND | 40 | 4.432 | 2.760 |
| css189 | 1B | 648 | 655 | 7000 | ND (xpEHH 2.82, 36/38) | xpEHH | both | ND | 36 | 2.824 | 2.350 |
| css190 | 1B | 662 | 675 | 13000 | ND (xpEHH 3.49, 20/20) | xpEHH | spr | ND | 20 | 3.486 | 2.641 |
| css191 | 1B | 665 | 670 | 5000 | ND (xpEHH 2.42, 12/12) | xpEHH | both | ND | 12 | 2.419 | 2.221 |
| css192 | 1B | 667 | 673 | 6000 | ND (Rsb 2.94, 9/9) | Rsb | both | ND | 9 | 2.942 | 2.423 |
| css193 | 1D | 26 | 33 | 7000 | gpl2 (xpEHH 2.53, 7/7) | xpEHH | both | gpl2 | 7 | 2.534 | 2.298 |
| css194 | 1D | 26 | 33 | 7000 | gpl2 (xpEHH 2.71, 7/7) | xpEHH | win | gpl2 | 7 | 2.706 | 2.447 |
| css195 | 1D | 26 | 35 | 9000 | HRW2 (xpEHH 3.00, 9/9) | xpEHH | win | HRW2 | 9 | 3.001 | 2.568 |
| css196 | 1D | 31 | 41 | 10000 | HRW2 (Rsb 2.75, 17/17) | Rsb | win | HRW2 | 17 | 2.752 | 2.657 |
| css197 | 1D | 31 | 58 | 27000 | SWS2 (Fst 0.60, 56/57) | Fst | spr | SWS2 | 56 | 0.597 | 0.497 |
| css198 | 1D | 34 | 41 | 7000 | gpl2 (Rsb 2.34, 14/15) | Rsb | win | gpl2 | 14 | 2.342 | 2.163 |
| css199 | 1D | 36 | 41 | 5000 | gpl (Fst 0.17, 9/10) | Fst | both | gpl | 9 | 0.171 | 0.161 |
| css200 | 1D | 36 | 41 | 5000 | gpl2 (Rsb 2.29, 9/10) | Rsb | both | gpl2 | 9 | 2.290 | 2.194 |
| css201 | 1D | 36 | 58 | 22000 | nor2 (Fst 0.30, 49/50) | Fst | both | nor2 | 49 | 0.296 | 0.218 |
| css202 | 1D | 36 | 41 | 5000 | pac2 (Fst 0.45, 9/10) | Fst | both | pac2 | 9 | 0.451 | 0.451 |
| css203 | 1D | 36 | 58 | 22000 | pac2 (Fst 0.55, 49/50) | Fst | spr | pac2 | 49 | 0.545 | 0.505 |
| css204 | 1D | 36 | 58 | 22000 | spr2 (Fst 0.41, 49/50) | Fst | spr | spr2 | 49 | 0.410 | 0.288 |
| css205 | 1D | 36 | 41 | 5000 | TX (Fst 0.29, 9/10) | Fst | both | TX | 9 | 0.288 | 0.265 |
| css206 | 1D | 38 | 58 | 20000 | pnw2 (Fst 0.68, 42/43) | Fst | spr | pnw2 | 42 | 0.678 | 0.573 |
| css207 | 1D | 42 | 58 | 16000 | all2 (Fst 0.16, 33/33) | Fst | both | all2 | 33 | 0.163 | 0.119 |
| css208 | 1D | 42 | 58 | 16000 | eas2 (Fst 0.23, 32/33) | Fst | both | eas2 | 32 | 0.233 | 0.206 |
| css209 | 1D | 42 | 58 | 16000 | pac2 (Fst 0.47, 33/33) | Fst | both | pac2 | 33 | 0.467 | 0.435 |
| css210 | 1D | 42 | 54 | 12000 | pnw2 (Fst 0.31, 29/29) | Fst | both | pnw2 | 29 | 0.305 | 0.269 |
| css211 | 1D | 45 | 58 | 13000 | nor2 (Fst 0.27, 27/27) | Fst | spr | nor2 | 27 | 0.267 | 0.263 |
| css212 | 1D | 46 | 58 | 12000 | eas2 (Fst 0.21, 26/26) | Fst | win | eas2 | 26 | 0.214 | 0.201 |
| css213 | 1D | 46 | 58 | 12000 | SRW2 (Fst 0.25, 26/26) | Fst | win | SRW2 | 26 | 0.250 | 0.243 |
| css214 | 1D | 406 | 411 | 5000 | eas (Fst 0.31, 24/26) | Fst | win | eas | 24 | 0.315 | 0.262 |
| css215 | 1D | 406 | 412 | 6000 | eas (Fst 0.33, 25/27) | Fst | both | eas | 25 | 0.334 | 0.295 |
| css216 | 1D | 406 | 422 | 16000 | gpl2 (Fst 0.26, 60/62) | Fst | both | gpl2 | 60 | 0.262 | 0.236 |
| css217 | 1D | 406 | 422 | 16000 | gpl2 (Fst 0.28, 60/62) | Fst | win | gpl2 | 60 | 0.277 | 0.234 |
| css218 | 1D | 406 | 422 | 16000 | HRW2 (Fst 0.16, 60/62) | Fst | win | HRW2 | 60 | 0.160 | 0.126 |
| css219 | 1D | 406 | 412 | 6000 | MT (Fst 0.26, 25/27) | Fst | both | MT | 25 | 0.265 | 0.258 |
| css220 | 1D | 406 | 422 | 16000 | ND (Fst 0.31, 60/62) | Fst | spr | ND | 60 | 0.311 | 0.305 |
| css221 | 1D | 406 | 422 | 16000 | ND (Fst 0.41, 60/62) | Fst | both | ND | 60 | 0.410 | 0.374 |
| css222 | 1D | 406 | 422 | 16000 | nor (Fst 0.33, 60/62) | Fst | both | nor | 60 | 0.332 | 0.290 |
| css223 | 1D | 406 | 422 | 16000 | nor (Fst 0.34, 60/62) | Fst | spr | nor | 60 | 0.344 | 0.337 |
| css224 | 1D | 406 | 422 | 16000 | pnw (Fst 0.19, 60/62) | Fst | spr | pnw | 60 | 0.188 | 0.172 |
| css225 | 1D | 406 | 422 | 16000 | SWS (Fst 0.39, 60/62) | Fst | spr | SWS | 60 | 0.390 | 0.361 |
| css226 | 1D | 406 | 422 | 16000 | win2 (Fst 0.12, 60/62) | Fst | win | win2 | 60 | 0.117 | 0.085 |
| css227 | 1D | 411 | 422 | 11000 | all2 (Fst 0.09, 35/36) | Fst | both | all2 | 35 | 0.092 | 0.079 |
| css228 | 1D | 415 | 422 | 7000 | SWW (Fst 0.26, 17/18) | Fst | win | SWW | 17 | 0.259 | 0.250 |
| css229 | 1D | 483 | 498 | 15000 | CA (Fst 0.22, 98/104) | Fst | spr | CA | 98 | 0.218 | 0.197 |
| css230 | 1D | 483 | 498 | 15000 | CA (Fst 0.35, 97/104) | Fst | both | CA | 97 | 0.355 | 0.336 |
| css231 | 1D | 483 | 498 | 15000 | pac (Fst 0.24, 98/104) | Fst | spr | pac | 98 | 0.237 | 0.219 |
| css232 | 1D | 483 | 498 | 15000 | pac (Fst 0.38, 97/104) | Fst | both | pac | 97 | 0.375 | 0.360 |
| css233 | 2A | 48 | 59 | 11000 | CO (Rsb 2.89, 17/17) | Rsb | win | CO | 17 | 2.885 | 2.535 |
| css234 | 2A | 49 | 58 | 9000 | NE (Rsb 3.46, 9/10) | Rsb | both | NE | 9 | 3.459 | 2.875 |
| css235 | 2A | 51 | 59 | 8000 | CO (Rsb 3.47, 12/13) | Rsb | both | CO | 12 | 3.474 | 2.820 |
| css236 | 2A | 51 | 59 | 8000 | CO (xpEHH 2.52, 12/13) | xpEHH | win | CO | 12 | 2.517 | 2.197 |
| css237 | 2A | 51 | 59 | 8000 | nor (Rsb 3.08, 12/13) | Rsb | win | nor | 12 | 3.082 | 2.709 |
| css238 | 2A | 53 | 58 | 5000 | NE (xpEHH 2.30, 6/6) | xpEHH | win | NE | 6 | 2.303 | 2.246 |
| css239 | 2A | 53 | 58 | 5000 | NE (xpEHH 3.17, 6/6) | xpEHH | both | NE | 6 | 3.170 | 3.014 |
| css240 | 2A | 54 | 59 | 5000 | HRW (Rsb 2.23, 6/6) | Rsb | win | HRW | 6 | 2.228 | 2.157 |
| css241 | 2A | 54 | 59 | 5000 | WA (Fst 0.23, 6/6) | Fst | both | WA | 6 | 0.233 | 0.208 |
| css242 | 2A | 62 | 86 | 24000 | CO (Rsb 3.51, 57/57) | Rsb | win | CO | 57 | 3.512 | 2.584 |
| css243 | 2A | 62 | 83 | 21000 | CO (Rsb 3.91, 42/42) | Rsb | both | CO | 42 | 3.908 | 2.769 |
| css244 | 2A | 62 | 86 | 24000 | CO (xpEHH 3.12, 57/57) | xpEHH | win | CO | 57 | 3.117 | 2.631 |
| css245 | 2A | 62 | 86 | 24000 | CO (xpEHH 3.47, 57/57) | xpEHH | both | CO | 57 | 3.474 | 2.684 |
| css246 | 2A | 62 | 78 | 16000 | gpl (Rsb 3.33, 20/20) | Rsb | win | gpl | 20 | 3.326 | 2.628 |
| css247 | 2A | 62 | 78 | 16000 | gpl (Rsb 3.38, 20/20) | Rsb | both | gpl | 20 | 3.376 | 2.655 |
| css248 | 2A | 62 | 83 | 21000 | HRW (Rsb 3.45, 41/42) | Rsb | win | HRW | 41 | 3.455 | 2.388 |
| css249 | 2A | 62 | 78 | 16000 | NE (Rsb 3.52, 19/19) | Rsb | both | NE | 19 | 3.518 | 2.848 |
| css250 | 2A | 62 | 70 | 8000 | NE (xpEHH 2.67, 8/8) | xpEHH | win | NE | 8 | 2.667 | 2.498 |
| css251 | 2A | 62 | 83 | 21000 | NE (xpEHH 3.43, 39/41) | xpEHH | both | NE | 39 | 3.428 | 2.374 |
| css252 | 2A | 62 | 88 | 26000 | nor (Rsb 4.10, 57/57) | Rsb | win | nor | 57 | 4.097 | 2.963 |
| css253 | 2A | 62 | 102 | 40000 | nor (xpEHH 3.48, 79/79) | xpEHH | win | nor | 79 | 3.476 | 2.706 |
| css254 | 2A | 64 | 70 | 6000 | HRW (xpEHH 2.41, 6/6) | xpEHH | win | HRW | 6 | 2.407 | 2.226 |
| css255 | 2A | 71 | 85 | 14000 | MT (Rsb 3.20, 45/47) | Rsb | both | MT | 45 | 3.203 | 2.459 |
| css256 | 2A | 77 | 86 | 9000 | HRW (xpEHH 2.42, 37/37) | xpEHH | win | HRW | 37 | 2.422 | 2.139 |
| css257 | 2A | 91 | 98 | 7000 | eas (Fst 0.38, 12/13) | Fst | win | eas | 12 | 0.376 | 0.354 |
| css258 | 2A | 91 | 98 | 7000 | IN (Fst 0.63, 12/13) | Fst | win | IN | 12 | 0.625 | 0.588 |
| css259 | 2A | 91 | 98 | 7000 | IN (Fst 0.65, 12/13) | Fst | both | IN | 12 | 0.649 | 0.558 |
| css260 | 2A | 91 | 98 | 7000 | SC (Fst 0.82, 12/13) | Fst | both | SC | 12 | 0.824 | 0.738 |
| css261 | 2A | 91 | 98 | 7000 | SC (Fst 0.82, 12/13) | Fst | win | SC | 12 | 0.824 | 0.783 |
| css262 | 2A | 91 | 98 | 7000 | SRW (Fst 0.44, 12/13) | Fst | win | SRW | 12 | 0.441 | 0.417 |
| css263 | 2A | 109 | 114 | 5000 | nor (xpEHH 2.70, 6/6) | xpEHH | win | nor | 6 | 2.702 | 2.455 |
| css264 | 2A | 116 | 123 | 7000 | nor (xpEHH 2.31, 8/8) | xpEHH | win | nor | 8 | 2.307 | 2.147 |
| css265 | 2A | 117 | 124 | 7000 | HRW (xpEHH 2.14, 10/11) | xpEHH | win | HRW | 10 | 2.135 | 2.097 |
| css266 | 2A | 118 | 123 | 5000 | nor (Fst 0.21, 9/10) | Fst | win | nor | 9 | 0.206 | 0.198 |
| css267 | 2A | 366 | 374 | 8000 | MT (Fst 0.29, 7/7) | Fst | both | MT | 7 | 0.294 | 0.293 |
| css268 | 2A | 394 | 400 | 6000 | MT (Fst 0.31, 5/5) | Fst | both | MT | 5 | 0.306 | 0.297 |
| css269 | 2A | 416 | 428 | 12000 | MT (Fst 0.31, 11/11) | Fst | both | MT | 11 | 0.310 | 0.300 |
| css270 | 2A | 497 | 506 | 9000 | CO (Fst 0.23, 11/11) | Fst | both | CO | 11 | 0.226 | 0.206 |
| css271 | 2A | 568 | 578 | 10000 | all2 (Fst 0.10, 15/16) | Fst | both | all2 | 15 | 0.098 | 0.093 |
| css272 | 2A | 568 | 580 | 12000 | CO (Fst 0.23, 20/21) | Fst | both | CO | 20 | 0.231 | 0.210 |
| css273 | 2A | 568 | 578 | 10000 | gpl (Fst 0.23, 15/16) | Fst | both | gpl | 15 | 0.232 | 0.223 |
| css274 | 2A | 568 | 578 | 10000 | gpl (Fst 0.28, 15/16) | Fst | win | gpl | 15 | 0.280 | 0.272 |
| css275 | 2A | 568 | 578 | 10000 | HRW (Fst 0.39, 15/16) | Fst | win | HRW | 15 | 0.386 | 0.375 |
| css276 | 2A | 568 | 578 | 10000 | KS (Fst 0.21, 15/16) | Fst | both | KS | 15 | 0.213 | 0.207 |
| css277 | 2A | 568 | 578 | 10000 | KS (Fst 0.24, 15/16) | Fst | win | KS | 15 | 0.245 | 0.238 |
| css278 | 2A | 568 | 578 | 10000 | NE (Fst 0.25, 15/16) | Fst | both | NE | 15 | 0.250 | 0.247 |
| css279 | 2A | 568 | 578 | 10000 | NE (Fst 0.32, 15/16) | Fst | win | NE | 15 | 0.322 | 0.319 |
| css280 | 2A | 568 | 578 | 10000 | nor2 (Fst 0.23, 15/16) | Fst | both | nor2 | 15 | 0.227 | 0.221 |
| css281 | 2A | 568 | 578 | 10000 | nor2 (Fst 0.36, 15/16) | Fst | spr | nor2 | 15 | 0.357 | 0.346 |
| css282 | 2A | 568 | 581 | 13000 | pnw (Rsb 2.76, 22/23) | Rsb | both | pnw | 22 | 2.760 | 2.345 |
| css283 | 2A | 568 | 578 | 10000 | spr2 (Fst 0.20, 15/16) | Fst | spr | spr2 | 15 | 0.200 | 0.192 |
| css284 | 2A | 568 | 578 | 10000 | SWW (Fst 0.30, 15/16) | Fst | win | SWW | 15 | 0.299 | 0.292 |
| css285 | 2A | 568 | 578 | 10000 | WA (Fst 0.17, 15/16) | Fst | both | WA | 15 | 0.173 | 0.170 |
| css286 | 2A | 568 | 578 | 10000 | WA (Fst 0.23, 15/16) | Fst | win | WA | 15 | 0.234 | 0.229 |
| css287 | 2A | 568 | 583 | 15000 | WA (Rsb 2.76, 23/24) | Rsb | both | WA | 23 | 2.762 | 2.407 |
| css288 | 2A | 568 | 583 | 15000 | WA (xpEHH 2.94, 24/24) | xpEHH | both | WA | 24 | 2.942 | 2.577 |
| css289 | 2A | 569 | 583 | 14000 | pnw (xpEHH 2.71, 23/24) | xpEHH | both | pnw | 23 | 2.713 | 2.250 |
| css290 | 2A | 578 | 583 | 5000 | CA (xpEHH 2.12, 8/8) | xpEHH | spr | CA | 8 | 2.121 | 2.092 |
| css291 | 2A | 578 | 583 | 5000 | OR (xpEHH 2.41, 7/7) | xpEHH | win | OR | 7 | 2.407 | 2.373 |
| css292 | 2A | 578 | 583 | 5000 | pac (xpEHH 2.13, 8/8) | xpEHH | spr | pac | 8 | 2.130 | 2.098 |
| css293 | 2A | 578 | 583 | 5000 | SWW (xpEHH 2.48, 7/7) | xpEHH | win | SWW | 7 | 2.484 | 2.437 |
| css294 | 2A | 605 | 611 | 6000 | CA (xpEHH 2.30, 5/5) | xpEHH | both | CA | 5 | 2.302 | 2.255 |
| css295 | 2A | 605 | 611 | 6000 | CA (xpEHH 2.43, 5/5) | xpEHH | spr | CA | 5 | 2.429 | 2.369 |
| css296 | 2A | 605 | 611 | 6000 | CO (Rsb 2.88, 5/5) | Rsb | both | CO | 5 | 2.884 | 2.238 |
| css297 | 2A | 605 | 612 | 7000 | CO (xpEHH 2.30, 7/7) | xpEHH | win | CO | 7 | 2.295 | 2.119 |
| css298 | 2A | 605 | 612 | 7000 | CO (xpEHH 2.51, 7/7) | xpEHH | both | CO | 7 | 2.514 | 2.285 |
| css299 | 2A | 605 | 612 | 7000 | NY (xpEHH 3.36, 7/7) | xpEHH | win | NY | 7 | 3.364 | 3.227 |
| css300 | 2A | 605 | 612 | 7000 | OR (Rsb 3.57, 7/7) | Rsb | win | OR | 7 | 3.574 | 3.102 |
| css301 | 2A | 605 | 612 | 7000 | OR (xpEHH 3.79, 7/7) | xpEHH | win | OR | 7 | 3.794 | 3.535 |
| css302 | 2A | 605 | 611 | 6000 | pac (xpEHH 2.33, 5/5) | xpEHH | both | pac | 5 | 2.326 | 2.268 |
| css303 | 2A | 605 | 612 | 7000 | pac (xpEHH 2.75, 7/7) | xpEHH | spr | pac | 7 | 2.754 | 2.596 |
| css304 | 2A | 605 | 611 | 6000 | pnw (Rsb 2.96, 5/5) | Rsb | both | pnw | 5 | 2.965 | 2.738 |
| css305 | 2A | 605 | 612 | 7000 | pnw (Rsb 3.33, 7/7) | Rsb | win | pnw | 7 | 3.329 | 2.940 |
| css306 | 2A | 605 | 611 | 6000 | pnw (xpEHH 2.63, 5/5) | xpEHH | win | pnw | 5 | 2.631 | 2.496 |
| css307 | 2A | 605 | 612 | 7000 | SC (xpEHH 2.48, 6/6) | xpEHH | win | SC | 6 | 2.477 | 2.402 |
| css308 | 2A | 605 | 612 | 7000 | SC (xpEHH 2.76, 6/6) | xpEHH | both | SC | 6 | 2.762 | 2.699 |
| css309 | 2A | 605 | 612 | 7000 | SWW (Rsb 3.77, 7/7) | Rsb | win | SWW | 7 | 3.775 | 3.089 |
| css310 | 2A | 605 | 612 | 7000 | SWW (xpEHH 3.23, 7/7) | xpEHH | win | SWW | 7 | 3.228 | 3.020 |
| css311 | 2A | 605 | 612 | 7000 | WA (Rsb 3.99, 7/7) | Rsb | both | WA | 7 | 3.987 | 3.344 |
| css312 | 2A | 605 | 611 | 6000 | WA (xpEHH 2.28, 5/5) | xpEHH | win | WA | 5 | 2.285 | 2.182 |
| css313 | 2A | 605 | 611 | 6000 | WA (xpEHH 2.87, 5/5) | xpEHH | both | WA | 5 | 2.874 | 2.759 |
| css314 | 2A | 675 | 685 | 10000 | CA (Rsb 3.26, 18/18) | Rsb | spr | CA | 18 | 3.264 | 3.055 |
| css315 | 2A | 675 | 685 | 10000 | CA (xpEHH 2.93, 18/18) | xpEHH | spr | CA | 18 | 2.931 | 2.901 |
| css316 | 2A | 675 | 685 | 10000 | CO (Rsb 2.64, 18/18) | Rsb | both | CO | 18 | 2.635 | 2.447 |
| css317 | 2A | 675 | 685 | 10000 | CO (xpEHH 2.29, 18/18) | xpEHH | win | CO | 18 | 2.290 | 2.263 |
| css318 | 2A | 675 | 685 | 10000 | CO (xpEHH 2.42, 18/18) | xpEHH | both | CO | 18 | 2.419 | 2.367 |
| css319 | 2A | 675 | 685 | 10000 | NY (Fst 0.54, 18/18) | Fst | win | NY | 18 | 0.539 | 0.462 |
| css320 | 2A | 675 | 685 | 10000 | NY (Rsb 3.24, 18/18) | Rsb | win | NY | 18 | 3.240 | 2.919 |
| css321 | 2A | 675 | 685 | 10000 | NY (xpEHH 2.67, 18/18) | xpEHH | win | NY | 18 | 2.670 | 2.615 |
| css322 | 2A | 675 | 685 | 10000 | OR (Rsb 3.14, 18/18) | Rsb | win | OR | 18 | 3.136 | 2.981 |
| css323 | 2A | 675 | 684 | 9000 | OR (xpEHH 2.28, 17/18) | xpEHH | win | OR | 17 | 2.283 | 2.251 |
| css324 | 2A | 675 | 685 | 10000 | pac (Rsb 3.36, 18/18) | Rsb | spr | pac | 18 | 3.363 | 3.193 |
| css325 | 2A | 675 | 685 | 10000 | pac (xpEHH 3.23, 18/18) | xpEHH | spr | pac | 18 | 3.228 | 3.198 |
| css326 | 2A | 675 | 685 | 10000 | SWW (Rsb 2.93, 18/18) | Rsb | win | SWW | 18 | 2.927 | 2.754 |
| css327 | 2A | 680 | 685 | 5000 | CO (Rsb 2.39, 8/8) | Rsb | win | CO | 8 | 2.385 | 2.353 |
| css328 | 2A | 690 | 698 | 8000 | CA (Rsb 5.86, 22/24) | Rsb | spr | CA | 22 | 5.855 | 4.326 |
| css329 | 2A | 690 | 696 | 6000 | CA (xpEHH 5.42, 14/14) | xpEHH | spr | CA | 14 | 5.422 | 4.830 |
| css330 | 2A | 690 | 699 | 9000 | NY (Rsb 4.02, 23/25) | Rsb | win | NY | 23 | 4.023 | 3.054 |
| css331 | 2A | 690 | 695 | 5000 | NY (xpEHH 4.15, 7/7) | xpEHH | win | NY | 7 | 4.152 | 3.851 |
| css332 | 2A | 690 | 698 | 8000 | pac (Rsb 5.71, 22/24) | Rsb | spr | pac | 22 | 5.712 | 4.382 |
| css333 | 2A | 690 | 696 | 6000 | pac (xpEHH 5.56, 14/14) | xpEHH | spr | pac | 14 | 5.562 | 5.029 |
| css334 | 2A | 691 | 698 | 7000 | CA (Rsb 3.63, 21/23) | Rsb | both | CA | 21 | 3.631 | 2.900 |
| css335 | 2A | 691 | 696 | 5000 | CA (xpEHH 3.58, 13/13) | xpEHH | both | CA | 13 | 3.578 | 3.043 |
| css336 | 2A | 691 | 696 | 5000 | OR (Rsb 3.89, 13/13) | Rsb | win | OR | 13 | 3.889 | 3.196 |
| css337 | 2A | 691 | 697 | 6000 | pac (Rsb 3.58, 20/22) | Rsb | both | pac | 20 | 3.577 | 2.974 |
| css338 | 2A | 691 | 696 | 5000 | pac (xpEHH 3.51, 13/13) | xpEHH | both | pac | 13 | 3.508 | 3.061 |
| css339 | 2A | 691 | 700 | 9000 | pac2 (Rsb 2.55, 8/8) | Rsb | both | pac2 | 8 | 2.552 | 2.544 |
| css340 | 2A | 691 | 696 | 5000 | pac2 (xpEHH 2.25, 8/8) | xpEHH | spr | pac2 | 8 | 2.254 | 2.204 |
| css341 | 2A | 691 | 696 | 5000 | SWW (Rsb 4.01, 13/13) | Rsb | win | SWW | 13 | 4.014 | 3.153 |
| css342 | 2A | 695 | 700 | 5000 | NE (Rsb 3.27, 20/21) | Rsb | win | NE | 20 | 3.265 | 2.623 |
| css343 | 2A | 697 | 712 | 15000 | HRW2 (xpEHH 3.48, 22/23) | xpEHH | win | HRW2 | 22 | 3.478 | 2.704 |
| css344 | 2A | 714 | 727 | 13000 | gpl2 (xpEHH 3.36, 34/35) | xpEHH | win | gpl2 | 34 | 3.362 | 2.713 |
| css345 | 2A | 714 | 722 | 8000 | HRW2 (Rsb 3.47, 27/29) | Rsb | win | HRW2 | 27 | 3.469 | 2.741 |
| css346 | 2A | 714 | 727 | 13000 | HRW2 (xpEHH 3.40, 33/34) | xpEHH | win | HRW2 | 33 | 3.404 | 2.627 |
| css347 | 2A | 715 | 724 | 9000 | HRW (Rsb 4.84, 29/33) | Rsb | win | HRW | 29 | 4.842 | 2.673 |
| css348 | 2A | 715 | 725 | 10000 | NE (xpEHH 3.53, 30/32) | xpEHH | win | NE | 30 | 3.526 | 2.591 |
| css349 | 2A | 717 | 725 | 8000 | CO (xpEHH 3.43, 23/24) | xpEHH | win | CO | 23 | 3.434 | 2.660 |
| css350 | 2A | 717 | 725 | 8000 | CO (xpEHH 3.80, 23/24) | xpEHH | both | CO | 23 | 3.801 | 2.742 |
| css351 | 2A | 717 | 723 | 6000 | gpl (xpEHH 3.68, 21/23) | xpEHH | win | gpl | 21 | 3.675 | 2.781 |
| css352 | 2A | 717 | 725 | 8000 | HRW (xpEHH 3.86, 23/24) | xpEHH | win | HRW | 23 | 3.857 | 2.777 |
| css353 | 2A | 717 | 722 | 5000 | win2 (Rsb 4.25, 18/19) | Rsb | win | win2 | 18 | 4.253 | 3.141 |
| css354 | 2A | 718 | 725 | 7000 | CO (Rsb 3.59, 18/21) | Rsb | win | CO | 18 | 3.593 | 2.510 |
| css355 | 2A | 718 | 727 | 9000 | gpl2 (xpEHH 2.71, 19/21) | xpEHH | both | gpl2 | 19 | 2.707 | 2.400 |
| css356 | 2A | 718 | 725 | 7000 | NE (Rsb 3.62, 19/20) | Rsb | win | NE | 19 | 3.620 | 2.640 |
| css357 | 2A | 718 | 727 | 9000 | win2 (xpEHH 3.74, 19/21) | xpEHH | win | win2 | 19 | 3.745 | 3.030 |
| css358 | 2A | 764 | 770 | 6000 | SWW2 (Fst 0.36, 29/33) | Fst | win | SWW2 | 29 | 0.365 | 0.281 |
| css359 | 2B | 42 | 48 | 6000 | CO (Fst 0.24, 7/7) | Fst | both | CO | 7 | 0.241 | 0.185 |
| css360 | 2B | 42 | 48 | 6000 | gpl (Fst 0.38, 7/7) | Fst | win | gpl | 7 | 0.380 | 0.272 |
| css361 | 2B | 42 | 48 | 6000 | TX (Fst 0.25, 7/7) | Fst | both | TX | 7 | 0.253 | 0.189 |
| css362 | 2B | 104 | 111 | 7000 | CO (Rsb 2.48, 19/21) | Rsb | both | CO | 19 | 2.477 | 2.227 |
| css363 | 2B | 106 | 113 | 7000 | CO (xpEHH 2.48, 22/23) | xpEHH | win | CO | 22 | 2.478 | 2.215 |
| css364 | 2B | 106 | 111 | 5000 | KS (Fst 0.24, 11/12) | Fst | both | KS | 11 | 0.236 | 0.170 |
| css365 | 2B | 133 | 138 | 5000 | MN (Fst 0.35, 5/5) | Fst | spr | MN | 5 | 0.353 | 0.335 |
| css366 | 2B | 142 | 149 | 7000 | gpl2 (xpEHH 2.96, 34/37) | xpEHH | both | gpl2 | 34 | 2.955 | 2.520 |
| css367 | 2B | 145 | 150 | 5000 | SC (xpEHH 2.08, 5/5) | xpEHH | win | SC | 5 | 2.081 | 2.036 |
| css368 | 2B | 161 | 166 | 5000 | gpl (Rsb 2.81, 10/11) | Rsb | spr | gpl | 10 | 2.808 | 2.234 |
| css369 | 2B | 162 | 171 | 9000 | gpl (xpEHH 2.68, 17/18) | xpEHH | spr | gpl | 17 | 2.683 | 2.335 |
| css370 | 2B | 163 | 168 | 5000 | CO (xpEHH 2.28, 12/13) | xpEHH | win | CO | 12 | 2.280 | 2.135 |
| css371 | 2B | 172 | 177 | 5000 | gpl (xpEHH 2.28, 10/11) | xpEHH | spr | gpl | 10 | 2.275 | 2.232 |
| css372 | 2B | 237 | 244 | 7000 | HRW2 (Rsb 2.23, 11/11) | Rsb | win | HRW2 | 11 | 2.226 | 2.134 |
| css373 | 2B | 237 | 244 | 7000 | IN (Fst 0.61, 11/11) | Fst | win | IN | 11 | 0.609 | 0.488 |
| css374 | 2B | 237 | 244 | 7000 | MN (Fst 0.39, 11/11) | Fst | spr | MN | 11 | 0.394 | 0.364 |
| css375 | 2B | 388 | 394 | 6000 | all2 (Fst 0.12, 9/10) | Fst | both | all2 | 9 | 0.119 | 0.081 |
| css376 | 2B | 388 | 394 | 6000 | gpl (Fst 0.32, 9/10) | Fst | spr | gpl | 9 | 0.316 | 0.295 |
| css377 | 2B | 388 | 397 | 9000 | HRS (Fst 0.43, 17/18) | Fst | spr | HRS | 17 | 0.431 | 0.385 |
| css378 | 2B | 388 | 393 | 5000 | HRS2 (Fst 0.20, 5/5) | Fst | spr | HRS2 | 5 | 0.204 | 0.187 |
| css379 | 2B | 388 | 407 | 19000 | MN (Fst 0.39, 36/37) | Fst | spr | MN | 36 | 0.394 | 0.387 |
| css380 | 2B | 388 | 394 | 6000 | MN (Fst 0.65, 9/10) | Fst | both | MN | 9 | 0.649 | 0.611 |
| css381 | 2B | 388 | 393 | 5000 | nor2 (Fst 0.28, 5/5) | Fst | both | nor2 | 5 | 0.284 | 0.211 |
| css382 | 2B | 388 | 393 | 5000 | nor2 (Fst 0.39, 5/5) | Fst | spr | nor2 | 5 | 0.392 | 0.350 |
| css383 | 2B | 388 | 393 | 5000 | spr2 (Fst 0.19, 5/5) | Fst | spr | spr2 | 5 | 0.190 | 0.181 |
| css384 | 2B | 388 | 407 | 19000 | SWS (Fst 0.41, 36/37) | Fst | spr | SWS | 36 | 0.407 | 0.371 |
| css385 | 2B | 408 | 416 | 8000 | all2 (Fst 0.11, 16/17) | Fst | both | all2 | 16 | 0.110 | 0.074 |
| css386 | 2B | 408 | 415 | 7000 | gpl (Fst 0.26, 12/13) | Fst | spr | gpl | 12 | 0.261 | 0.259 |
| css387 | 2B | 408 | 415 | 7000 | HRS2 (Fst 0.19, 12/13) | Fst | spr | HRS2 | 12 | 0.189 | 0.177 |
| css388 | 2B | 408 | 447 | 39000 | MN (Fst 0.40, 79/81) | Fst | spr | MN | 79 | 0.402 | 0.371 |
| css389 | 2B | 408 | 416 | 8000 | MN (Fst 0.64, 16/17) | Fst | both | MN | 16 | 0.640 | 0.612 |
| css390 | 2B | 408 | 415 | 7000 | nor2 (Fst 0.28, 12/13) | Fst | both | nor2 | 12 | 0.284 | 0.216 |
| css391 | 2B | 408 | 415 | 7000 | nor2 (Fst 0.39, 12/13) | Fst | spr | nor2 | 12 | 0.392 | 0.362 |
| css392 | 2B | 408 | 415 | 7000 | spr2 (Fst 0.18, 12/13) | Fst | spr | spr2 | 12 | 0.181 | 0.181 |
| css393 | 2B | 408 | 417 | 9000 | SWS (Fst 0.39, 19/20) | Fst | spr | SWS | 19 | 0.395 | 0.367 |
| css394 | 2B | 409 | 414 | 5000 | HRS (Fst 0.42, 9/10) | Fst | spr | HRS | 9 | 0.417 | 0.376 |
| css395 | 2B | 418 | 437 | 19000 | SWS (Fst 0.39, 43/44) | Fst | spr | SWS | 43 | 0.388 | 0.357 |
| css396 | 2B | 425 | 447 | 22000 | gpl (Fst 0.39, 37/38) | Fst | spr | gpl | 37 | 0.390 | 0.248 |
| css397 | 2B | 425 | 445 | 20000 | HRS2 (Fst 0.20, 32/33) | Fst | spr | HRS2 | 32 | 0.204 | 0.190 |
| css398 | 2B | 425 | 445 | 20000 | nor2 (Fst 0.31, 32/33) | Fst | both | nor2 | 32 | 0.310 | 0.239 |
| css399 | 2B | 425 | 445 | 20000 | nor2 (Fst 0.43, 32/33) | Fst | spr | nor2 | 32 | 0.429 | 0.378 |
| css400 | 2B | 425 | 437 | 12000 | SC (Fst 0.63, 21/22) | Fst | win | SC | 21 | 0.628 | 0.558 |
| css401 | 2B | 425 | 445 | 20000 | spr2 (Fst 0.19, 32/33) | Fst | spr | spr2 | 32 | 0.190 | 0.178 |
| css402 | 2B | 439 | 445 | 6000 | SC (Fst 0.61, 5/5) | Fst | win | SC | 5 | 0.611 | 0.551 |
| css403 | 2B | 447 | 458 | 11000 | nor2 (Fst 0.43, 23/24) | Fst | spr | nor2 | 23 | 0.429 | 0.370 |
| css404 | 2B | 449 | 462 | 13000 | MN (Fst 0.35, 29/31) | Fst | spr | MN | 29 | 0.349 | 0.320 |
| css405 | 2B | 449 | 456 | 7000 | MN (Fst 0.61, 16/17) | Fst | both | MN | 16 | 0.609 | 0.518 |
| css406 | 2B | 451 | 458 | 7000 | nor2 (Fst 0.31, 15/16) | Fst | both | nor2 | 15 | 0.310 | 0.236 |
| css407 | 2B | 549 | 554 | 5000 | gpl (Fst 0.52, 8/8) | Fst | spr | gpl | 8 | 0.521 | 0.396 |
| css408 | 2B | 549 | 554 | 5000 | gpl (Rsb 3.22, 8/8) | Rsb | spr | gpl | 8 | 3.217 | 2.883 |
| css409 | 2B | 558 | 576 | 18000 | gpl (xpEHH 2.28, 17/17) | xpEHH | spr | gpl | 17 | 2.281 | 2.112 |
| css410 | 2B | 565 | 572 | 7000 | CO (Fst 0.18, 8/8) | Fst | both | CO | 8 | 0.185 | 0.167 |
| css411 | 2B | 565 | 572 | 7000 | ID (Fst 0.31, 8/8) | Fst | spr | ID | 8 | 0.310 | 0.258 |
| css412 | 2B | 597 | 602 | 5000 | gpl (Rsb 3.27, 6/6) | Rsb | spr | gpl | 6 | 3.269 | 2.674 |
| css413 | 2B | 597 | 606 | 9000 | SRW2 (xpEHH 2.53, 8/8) | xpEHH | win | SRW2 | 8 | 2.526 | 2.419 |
| css414 | 2B | 598 | 606 | 8000 | eas2 (xpEHH 2.45, 7/7) | xpEHH | win | eas2 | 7 | 2.445 | 2.371 |
| css415 | 2B | 598 | 606 | 8000 | eas2 (xpEHH 2.50, 7/7) | xpEHH | both | eas2 | 7 | 2.502 | 2.428 |
| css416 | 2B | 598 | 606 | 8000 | gpl (xpEHH 2.45, 7/7) | xpEHH | spr | gpl | 7 | 2.453 | 2.311 |
| css417 | 2B | 609 | 614 | 5000 | SC (Fst 0.68, 5/5) | Fst | win | SC | 5 | 0.678 | 0.628 |
| css418 | 2B | 647 | 667 | 20000 | gpl (xpEHH 2.94, 57/58) | xpEHH | spr | gpl | 57 | 2.938 | 2.327 |
| css419 | 2B | 655 | 665 | 10000 | HRS2 (xpEHH 2.76, 22/22) | xpEHH | spr | HRS2 | 22 | 2.764 | 2.397 |
| css420 | 2B | 660 | 668 | 8000 | gpl (Rsb 3.63, 20/21) | Rsb | spr | gpl | 20 | 3.632 | 2.699 |
| css421 | 2B | 689 | 706 | 17000 | gpl (Rsb 3.62, 60/60) | Rsb | spr | gpl | 60 | 3.618 | 2.515 |
| css422 | 2B | 689 | 706 | 17000 | gpl (xpEHH 2.86, 60/60) | xpEHH | spr | gpl | 60 | 2.859 | 2.462 |
| css423 | 2B | 689 | 711 | 22000 | HRS2 (xpEHH 3.26, 71/72) | xpEHH | spr | HRS2 | 71 | 3.257 | 2.516 |
| css424 | 2B | 689 | 694 | 5000 | MN (Rsb 2.95, 18/19) | Rsb | spr | MN | 18 | 2.954 | 2.441 |
| css425 | 2B | 689 | 706 | 17000 | MN (xpEHH 2.92, 58/59) | xpEHH | spr | MN | 58 | 2.919 | 2.459 |
| css426 | 2B | 689 | 694 | 5000 | nor2 (xpEHH 2.36, 16/16) | xpEHH | spr | nor2 | 16 | 2.364 | 2.232 |
| css427 | 2B | 691 | 698 | 7000 | HRS (xpEHH 2.73, 25/25) | xpEHH | spr | HRS | 25 | 2.734 | 2.408 |
| css428 | 2B | 692 | 697 | 5000 | HRS2 (Rsb 2.41, 7/7) | Rsb | spr | HRS2 | 7 | 2.407 | 2.151 |
| css429 | 2B | 692 | 697 | 5000 | nor2 (Rsb 2.20, 6/6) | Rsb | spr | nor2 | 6 | 2.200 | 2.079 |
| css430 | 2B | 692 | 697 | 5000 | pnw (Fst 0.30, 7/7) | Fst | spr | pnw | 7 | 0.301 | 0.265 |
| css431 | 2B | 692 | 697 | 5000 | WA (Fst 0.51, 7/7) | Fst | spr | WA | 7 | 0.505 | 0.473 |
| css432 | 2B | 696 | 712 | 16000 | nor2 (xpEHH 3.27, 46/46) | xpEHH | spr | nor2 | 46 | 3.270 | 2.718 |
| css433 | 2B | 698 | 712 | 14000 | nor2 (xpEHH 3.10, 45/45) | xpEHH | both | nor2 | 45 | 3.103 | 2.707 |
| css434 | 2B | 706 | 712 | 6000 | eas (Fst 0.29, 11/13) | Fst | both | eas | 11 | 0.288 | 0.251 |
| css435 | 2B | 706 | 712 | 6000 | eas (Fst 0.39, 12/13) | Fst | win | eas | 12 | 0.392 | 0.367 |
| css436 | 2B | 706 | 712 | 6000 | IN (Fst 0.56, 11/13) | Fst | win | IN | 11 | 0.562 | 0.476 |
| css437 | 2B | 718 | 733 | 15000 | HRS2 (xpEHH 2.80, 32/32) | xpEHH | spr | HRS2 | 32 | 2.801 | 2.569 |
| css438 | 2B | 718 | 734 | 16000 | nor2 (xpEHH 2.81, 33/33) | xpEHH | spr | nor2 | 33 | 2.809 | 2.604 |
| css439 | 2B | 718 | 733 | 15000 | nor2 (xpEHH 2.84, 32/32) | xpEHH | both | nor2 | 32 | 2.840 | 2.498 |
| css440 | 2B | 719 | 733 | 14000 | gpl (Rsb 3.01, 31/32) | Rsb | spr | gpl | 31 | 3.012 | 2.302 |
| css441 | 2B | 719 | 728 | 9000 | HRS2 (Rsb 2.88, 21/22) | Rsb | spr | HRS2 | 21 | 2.880 | 2.457 |
| css442 | 2B | 719 | 728 | 9000 | nor2 (Rsb 3.46, 20/22) | Rsb | spr | nor2 | 20 | 3.459 | 2.554 |
| css443 | 2B | 719 | 733 | 14000 | nor2 (Rsb 3.75, 30/32) | Rsb | both | nor2 | 30 | 3.748 | 2.749 |
| css444 | 2B | 719 | 733 | 14000 | spr2 (Rsb 3.40, 30/32) | Rsb | spr | spr2 | 30 | 3.402 | 2.520 |
| css445 | 2B | 721 | 731 | 10000 | spr2 (xpEHH 2.73, 28/30) | xpEHH | spr | spr2 | 28 | 2.733 | 2.406 |
| css446 | 2B | 724 | 731 | 7000 | gpl (xpEHH 2.17, 14/15) | xpEHH | spr | gpl | 14 | 2.174 | 2.082 |
| css447 | 2B | 724 | 733 | 9000 | gpl2 (Rsb 3.84, 17/17) | Rsb | both | gpl2 | 17 | 3.837 | 2.863 |
| css448 | 2B | 724 | 739 | 15000 | gpl2 (xpEHH 3.55, 28/28) | xpEHH | both | gpl2 | 28 | 3.551 | 2.649 |
| css449 | 2B | 735 | 743 | 8000 | nor2 (xpEHH 2.36, 13/14) | xpEHH | spr | nor2 | 13 | 2.358 | 2.216 |
| css450 | 2B | 735 | 757 | 22000 | nor2 (xpEHH 3.14, 34/34) | xpEHH | both | nor2 | 34 | 3.143 | 2.619 |
| css451 | 2B | 736 | 743 | 7000 | gpl2 (Rsb 3.05, 12/14) | Rsb | both | gpl2 | 12 | 3.054 | 2.391 |
| css452 | 2B | 738 | 745 | 7000 | nor2 (Rsb 3.88, 13/14) | Rsb | both | nor2 | 13 | 3.884 | 2.556 |
| css453 | 2B | 742 | 756 | 14000 | gpl2 (xpEHH 2.78, 19/20) | xpEHH | both | gpl2 | 19 | 2.783 | 2.528 |
| css454 | 2B | 742 | 747 | 5000 | HRS2 (xpEHH 2.19, 6/6) | xpEHH | spr | HRS2 | 6 | 2.191 | 2.100 |
| css455 | 2B | 745 | 751 | 6000 | gpl (Rsb 2.35, 5/5) | Rsb | spr | gpl | 5 | 2.350 | 2.180 |
| css456 | 2B | 745 | 751 | 6000 | HRW2 (Fst 0.18, 5/5) | Fst | win | HRW2 | 5 | 0.177 | 0.148 |
| css457 | 2B | 748 | 757 | 9000 | nor2 (xpEHH 2.49, 16/17) | xpEHH | spr | nor2 | 16 | 2.490 | 2.279 |
| css458 | 2B | 749 | 757 | 8000 | gpl2 (Rsb 3.10, 15/16) | Rsb | both | gpl2 | 15 | 3.101 | 2.369 |
| css459 | 2B | 749 | 757 | 8000 | nor2 (Rsb 3.38, 15/16) | Rsb | both | nor2 | 15 | 3.377 | 2.809 |
| css460 | 2B | 755 | 762 | 7000 | eas2 (xpEHH 2.73, 21/24) | xpEHH | win | eas2 | 21 | 2.728 | 2.354 |
| css461 | 2B | 755 | 762 | 7000 | eas2 (xpEHH 2.82, 21/24) | xpEHH | both | eas2 | 21 | 2.822 | 2.410 |
| css462 | 2D | 59 | 64 | 5000 | all2 (Fst 0.09, 5/5) | Fst | both | all2 | 5 | 0.088 | 0.081 |
| css463 | 2D | 59 | 64 | 5000 | CO (Fst 0.52, 5/5) | Fst | win | CO | 5 | 0.518 | 0.422 |
| css464 | 2D | 59 | 64 | 5000 | gpl2 (Fst 0.17, 5/5) | Fst | both | gpl2 | 5 | 0.171 | 0.161 |
| css465 | 2D | 59 | 64 | 5000 | gpl2 (Fst 0.21, 5/5) | Fst | win | gpl2 | 5 | 0.210 | 0.196 |
| css466 | 2D | 59 | 64 | 5000 | HRW2 (Fst 0.19, 5/5) | Fst | win | HRW2 | 5 | 0.193 | 0.187 |
| css467 | 2D | 59 | 64 | 5000 | NE (Fst 0.70, 5/5) | Fst | win | NE | 5 | 0.702 | 0.609 |
| css468 | 2D | 59 | 64 | 5000 | SWS2 (Fst 0.44, 5/5) | Fst | spr | SWS2 | 5 | 0.438 | 0.428 |
| css469 | 2D | 59 | 64 | 5000 | win2 (Fst 0.09, 5/5) | Fst | win | win2 | 5 | 0.092 | 0.086 |
| css470 | 2D | 70 | 77 | 7000 | gpl (Fst 0.25, 14/15) | Fst | win | gpl | 14 | 0.251 | 0.229 |
| css471 | 2D | 70 | 75 | 5000 | KS (Fst 0.27, 9/10) | Fst | win | KS | 9 | 0.268 | 0.245 |
| css472 | 2D | 70 | 77 | 7000 | OR (Fst 0.34, 14/15) | Fst | win | OR | 14 | 0.336 | 0.313 |
| css473 | 2D | 73 | 82 | 9000 | win2 (Fst 0.16, 43/44) | Fst | win | win2 | 43 | 0.157 | 0.092 |
| css474 | 2D | 75 | 82 | 7000 | all2 (xpEHH 2.26, 39/42) | xpEHH | both | all2 | 39 | 2.264 | 2.171 |
| css475 | 2D | 75 | 82 | 7000 | eas2 (Fst 0.26, 39/42) | Fst | both | eas2 | 39 | 0.265 | 0.223 |
| css476 | 2D | 75 | 82 | 7000 | eas2 (Fst 0.29, 39/42) | Fst | win | eas2 | 39 | 0.290 | 0.252 |
| css477 | 2D | 75 | 82 | 7000 | gpl2 (xpEHH 2.55, 40/42) | xpEHH | both | gpl2 | 40 | 2.548 | 2.365 |
| css478 | 2D | 75 | 82 | 7000 | pnw (Fst 0.29, 38/42) | Fst | win | pnw | 38 | 0.290 | 0.230 |
| css479 | 2D | 75 | 82 | 7000 | SRW2 (Fst 0.34, 39/42) | Fst | win | SRW2 | 39 | 0.343 | 0.302 |
| css480 | 2D | 75 | 82 | 7000 | WA (Fst 0.33, 38/42) | Fst | win | WA | 38 | 0.328 | 0.284 |
| css481 | 2D | 80 | 85 | 5000 | SWW2 (Rsb 2.41, 9/9) | Rsb | win | SWW2 | 9 | 2.413 | 2.098 |
| css482 | 2D | 569 | 583 | 14000 | IN (Fst 0.44, 71/72) | Fst | win | IN | 71 | 0.437 | 0.384 |
| css483 | 2D | 569 | 583 | 14000 | pac2 (Fst 0.44, 71/72) | Fst | both | pac2 | 71 | 0.438 | 0.438 |
| css484 | 2D | 569 | 583 | 14000 | pac2 (Fst 0.46, 71/72) | Fst | spr | pac2 | 71 | 0.459 | 0.459 |
| css485 | 2D | 589 | 599 | 10000 | WA (Fst 0.24, 21/22) | Fst | spr | WA | 21 | 0.239 | 0.238 |
| css486 | 2D | 606 | 611 | 5000 | ID (Fst 0.31, 9/10) | Fst | both | ID | 9 | 0.307 | 0.299 |
| css487 | 2D | 606 | 611 | 5000 | ID (Fst 0.34, 9/10) | Fst | spr | ID | 9 | 0.344 | 0.329 |
| css488 | 2D | 606 | 611 | 5000 | pnw (Fst 0.14, 9/10) | Fst | both | pnw | 9 | 0.136 | 0.133 |
| css489 | 2D | 606 | 611 | 5000 | SWS2 (Fst 0.52, 9/10) | Fst | spr | SWS2 | 9 | 0.517 | 0.482 |
| css490 | 2D | 612 | 619 | 7000 | pac2 (Fst 0.47, 24/26) | Fst | both | pac2 | 24 | 0.467 | 0.417 |
| css491 | 2D | 612 | 618 | 6000 | SWS2 (Fst 0.48, 21/24) | Fst | spr | SWS2 | 21 | 0.478 | 0.452 |
| css492 | 3A | 45 | 52 | 7000 | TX (Rsb 3.30, 9/10) | Rsb | both | TX | 9 | 3.305 | 2.651 |
| css493 | 3A | 47 | 52 | 5000 | CO (Fst 0.31, 5/5) | Fst | both | CO | 5 | 0.312 | 0.257 |
| css494 | 3A | 47 | 52 | 5000 | CO (Fst 0.36, 5/5) | Fst | win | CO | 5 | 0.364 | 0.326 |
| css495 | 3A | 47 | 52 | 5000 | gpl (Fst 0.35, 5/5) | Fst | win | gpl | 5 | 0.354 | 0.321 |
| css496 | 3A | 47 | 52 | 5000 | gpl (Fst 0.38, 5/5) | Fst | both | gpl | 5 | 0.383 | 0.318 |
| css497 | 3A | 47 | 52 | 5000 | HRW (Fst 0.46, 5/5) | Fst | win | HRW | 5 | 0.463 | 0.408 |
| css498 | 3A | 47 | 52 | 5000 | KS (Fst 0.34, 5/5) | Fst | win | KS | 5 | 0.341 | 0.297 |
| css499 | 3A | 47 | 52 | 5000 | KS (Fst 0.44, 5/5) | Fst | both | KS | 5 | 0.436 | 0.346 |
| css500 | 3A | 47 | 52 | 5000 | NE (Fst 0.50, 5/5) | Fst | win | NE | 5 | 0.504 | 0.436 |
| css501 | 3A | 47 | 52 | 5000 | NE (Fst 0.52, 5/5) | Fst | both | NE | 5 | 0.522 | 0.415 |
| css502 | 3A | 47 | 52 | 5000 | nor (Fst 0.23, 5/5) | Fst | win | nor | 5 | 0.228 | 0.209 |
| css503 | 3A | 47 | 52 | 5000 | SWW (Fst 0.32, 5/5) | Fst | win | SWW | 5 | 0.315 | 0.297 |
| css504 | 3A | 47 | 52 | 5000 | TX (Fst 0.23, 5/5) | Fst | both | TX | 5 | 0.231 | 0.203 |
| css505 | 3A | 49 | 58 | 9000 | HRW2 (Fst 0.15, 16/16) | Fst | win | HRW2 | 16 | 0.152 | 0.137 |
| css506 | 3A | 56 | 66 | 10000 | TX (xpEHH 2.94, 24/24) | xpEHH | both | TX | 24 | 2.935 | 2.362 |
| css507 | 3A | 57 | 66 | 9000 | KS (Rsb 2.55, 18/20) | Rsb | both | KS | 18 | 2.550 | 2.306 |
| css508 | 3A | 59 | 66 | 7000 | KS (Rsb 2.22, 11/11) | Rsb | win | KS | 11 | 2.219 | 2.103 |
| css509 | 3A | 61 | 66 | 5000 | TX (Fst 0.29, 6/6) | Fst | both | TX | 6 | 0.290 | 0.286 |
| css510 | 3A | 71 | 77 | 6000 | IN (Fst 0.71, 6/6) | Fst | win | IN | 6 | 0.708 | 0.573 |
| css511 | 3A | 440 | 449 | 9000 | KS (Fst 0.28, 9/9) | Fst | win | KS | 9 | 0.282 | 0.253 |
| css512 | 3A | 440 | 449 | 9000 | TX (Fst 0.35, 9/9) | Fst | both | TX | 9 | 0.352 | 0.340 |
| css513 | 3A | 494 | 502 | 8000 | KS (xpEHH 2.75, 11/11) | xpEHH | both | KS | 11 | 2.752 | 2.439 |
| css514 | 3A | 496 | 502 | 6000 | TX (xpEHH 2.56, 9/10) | xpEHH | both | TX | 9 | 2.564 | 2.309 |
| css515 | 3A | 513 | 525 | 12000 | KS (Rsb 3.33, 25/25) | Rsb | both | KS | 25 | 3.325 | 2.711 |
| css516 | 3A | 518 | 528 | 10000 | KS (xpEHH 3.50, 30/32) | xpEHH | both | KS | 30 | 3.501 | 2.763 |
| css517 | 3A | 519 | 525 | 6000 | gpl (Rsb 2.80, 15/16) | Rsb | both | gpl | 15 | 2.798 | 2.370 |
| css518 | 3A | 519 | 525 | 6000 | HRW (xpEHH 2.72, 15/16) | xpEHH | win | HRW | 15 | 2.716 | 2.272 |
| css519 | 3A | 519 | 527 | 8000 | KS (xpEHH 2.61, 21/24) | xpEHH | win | KS | 21 | 2.610 | 2.182 |
| css520 | 3A | 520 | 525 | 5000 | gpl (xpEHH 2.72, 14/15) | xpEHH | both | gpl | 14 | 2.724 | 2.520 |
| css521 | 3A | 520 | 528 | 8000 | ID (xpEHH 2.47, 26/28) | xpEHH | win | ID | 26 | 2.470 | 2.185 |
| css522 | 3A | 520 | 525 | 5000 | TX (xpEHH 2.85, 12/13) | xpEHH | both | TX | 12 | 2.850 | 2.699 |
| css523 | 3A | 531 | 553 | 22000 | KS (xpEHH 2.92, 73/76) | xpEHH | both | KS | 73 | 2.925 | 2.442 |
| css524 | 3A | 542 | 555 | 13000 | ID (xpEHH 2.31, 40/43) | xpEHH | win | ID | 40 | 2.309 | 2.151 |
| css525 | 3A | 545 | 552 | 7000 | TX (xpEHH 2.46, 23/23) | xpEHH | both | TX | 23 | 2.461 | 2.273 |
| css526 | 3A | 547 | 552 | 5000 | KS (xpEHH 2.32, 21/23) | xpEHH | win | KS | 21 | 2.324 | 2.184 |
| css527 | 3A | 553 | 559 | 6000 | TX (Fst 0.21, 13/13) | Fst | both | TX | 13 | 0.212 | 0.179 |
| css528 | 3A | 560 | 572 | 12000 | KS (xpEHH 2.77, 42/42) | xpEHH | both | KS | 42 | 2.773 | 2.328 |
| css529 | 3A | 560 | 566 | 6000 | SC (Fst 0.53, 22/23) | Fst | both | SC | 22 | 0.534 | 0.490 |
| css530 | 3A | 560 | 565 | 5000 | SC (Fst 0.59, 10/11) | Fst | win | SC | 10 | 0.593 | 0.550 |
| css531 | 3A | 568 | 577 | 9000 | KS (Rsb 2.67, 14/14) | Rsb | both | KS | 14 | 2.675 | 2.323 |
| css532 | 3A | 568 | 574 | 6000 | nor (Rsb 3.28, 12/12) | Rsb | win | nor | 12 | 3.284 | 2.305 |
| css533 | 3A | 640 | 645 | 5000 | OR (Fst 0.43, 5/5) | Fst | win | OR | 5 | 0.427 | 0.411 |
| css534 | 3A | 640 | 645 | 5000 | pnw (Fst 0.24, 5/5) | Fst | win | pnw | 5 | 0.236 | 0.230 |
| css535 | 3A | 640 | 645 | 5000 | SWW (Fst 0.42, 5/5) | Fst | win | SWW | 5 | 0.423 | 0.416 |
| css536 | 3A | 640 | 645 | 5000 | WA (Fst 0.38, 5/5) | Fst | both | WA | 5 | 0.383 | 0.369 |
| css537 | 3A | 640 | 645 | 5000 | WA (Fst 0.52, 5/5) | Fst | win | WA | 5 | 0.516 | 0.504 |
| css538 | 3B | 45 | 52 | 7000 | SC (Rsb 3.27, 8/8) | Rsb | win | SC | 8 | 3.273 | 2.717 |
| css539 | 3B | 45 | 53 | 8000 | SC (xpEHH 3.04, 10/10) | xpEHH | win | SC | 10 | 3.040 | 2.597 |
| css540 | 3B | 48 | 53 | 5000 | SC (xpEHH 2.98, 9/10) | xpEHH | both | SC | 9 | 2.984 | 2.423 |
| css541 | 3B | 50 | 58 | 8000 | gpl (xpEHH 2.25, 6/6) | xpEHH | spr | gpl | 6 | 2.252 | 2.133 |
| css542 | 3B | 50 | 70 | 20000 | MN (Rsb 3.88, 23/23) | Rsb | both | MN | 23 | 3.875 | 2.981 |
| css543 | 3B | 50 | 64 | 14000 | MN (Rsb 4.04, 14/14) | Rsb | spr | MN | 14 | 4.036 | 2.825 |
| css544 | 3B | 50 | 70 | 20000 | MN (xpEHH 3.32, 23/23) | xpEHH | both | MN | 23 | 3.322 | 2.629 |
| css545 | 3B | 50 | 70 | 20000 | MN (xpEHH 3.58, 23/23) | xpEHH | spr | MN | 23 | 3.584 | 2.671 |
| css546 | 3B | 57 | 70 | 13000 | SWW (Rsb 3.47, 16/16) | Rsb | win | SWW | 16 | 3.467 | 2.525 |
| css547 | 3B | 61 | 66 | 5000 | nor (Rsb 3.59, 9/10) | Rsb | both | nor | 9 | 3.585 | 2.927 |
| css548 | 3B | 62 | 70 | 8000 | nor (xpEHH 3.00, 11/11) | xpEHH | both | nor | 11 | 3.002 | 2.754 |
| css549 | 3B | 64 | 74 | 10000 | nor (Rsb 3.46, 20/20) | Rsb | win | nor | 20 | 3.461 | 2.780 |
| css550 | 3B | 65 | 78 | 13000 | nor (xpEHH 3.74, 40/40) | xpEHH | win | nor | 40 | 3.738 | 2.482 |
| css551 | 3B | 76 | 85 | 9000 | SWW (xpEHH 2.61, 23/23) | xpEHH | win | SWW | 23 | 2.609 | 2.382 |
| css552 | 3B | 104 | 111 | 7000 | nor (Fst 0.23, 5/5) | Fst | win | nor | 5 | 0.228 | 0.228 |
| css553 | 3B | 109 | 123 | 14000 | CA (Fst 0.19, 14/14) | Fst | spr | CA | 14 | 0.195 | 0.189 |
| css554 | 3B | 109 | 123 | 14000 | pac (Fst 0.21, 14/14) | Fst | spr | pac | 14 | 0.209 | 0.204 |
| css555 | 3B | 131 | 140 | 9000 | pnw2 (Fst 0.24, 9/9) | Fst | both | pnw2 | 9 | 0.239 | 0.196 |
| css556 | 3B | 131 | 140 | 9000 | SWS2 (Fst 0.47, 9/9) | Fst | spr | SWS2 | 9 | 0.471 | 0.426 |
| css557 | 3B | 145 | 157 | 12000 | SWW2 (Fst 0.23, 21/23) | Fst | win | SWW2 | 21 | 0.231 | 0.210 |
| css558 | 3B | 423 | 429 | 6000 | gpl (Fst 0.22, 5/5) | Fst | spr | gpl | 5 | 0.217 | 0.217 |
| css559 | 3B | 423 | 437 | 14000 | pnw (xpEHH 3.06, 17/17) | xpEHH | both | pnw | 17 | 3.064 | 2.660 |
| css560 | 3B | 423 | 437 | 14000 | WA (xpEHH 2.90, 16/16) | xpEHH | both | WA | 16 | 2.903 | 2.585 |
| css561 | 3B | 428 | 437 | 9000 | ID (Rsb 3.68, 12/12) | Rsb | both | ID | 12 | 3.677 | 2.591 |
| css562 | 3B | 428 | 437 | 9000 | ID (xpEHH 2.58, 12/12) | xpEHH | both | ID | 12 | 2.578 | 2.309 |
| css563 | 3B | 428 | 437 | 9000 | pnw (Rsb 4.76, 11/12) | Rsb | both | pnw | 11 | 4.760 | 3.012 |
| css564 | 3B | 428 | 437 | 9000 | SWW (xpEHH 2.41, 11/11) | xpEHH | win | SWW | 11 | 2.409 | 2.273 |
| css565 | 3B | 430 | 437 | 7000 | WA (Rsb 2.80, 7/7) | Rsb | both | WA | 7 | 2.800 | 2.640 |
| css566 | 3B | 431 | 437 | 6000 | pnw (Fst 0.23, 5/5) | Fst | both | pnw | 5 | 0.228 | 0.186 |
| css567 | 3B | 431 | 437 | 6000 | pnw (Fst 0.29, 5/5) | Fst | win | pnw | 5 | 0.290 | 0.233 |
| css568 | 3B | 431 | 437 | 6000 | SWW (Fst 0.33, 5/5) | Fst | win | SWW | 5 | 0.328 | 0.305 |
| css569 | 3B | 431 | 437 | 6000 | WA (Fst 0.31, 5/5) | Fst | both | WA | 5 | 0.308 | 0.251 |
| css570 | 3B | 431 | 437 | 6000 | WA (Fst 0.43, 5/5) | Fst | win | WA | 5 | 0.427 | 0.354 |
| css571 | 3B | 461 | 532 | 71000 | ID (xpEHH 3.10, 115/115) | xpEHH | both | ID | 115 | 3.101 | 2.688 |
| css572 | 3B | 461 | 516 | 55000 | pnw (Rsb 3.79, 95/96) | Rsb | both | pnw | 95 | 3.786 | 2.907 |
| css573 | 3B | 461 | 498 | 37000 | pnw (xpEHH 2.89, 61/61) | xpEHH | win | pnw | 61 | 2.894 | 2.552 |
| css574 | 3B | 461 | 532 | 71000 | pnw (xpEHH 3.77, 115/115) | xpEHH | both | pnw | 115 | 3.771 | 3.207 |
| css575 | 3B | 461 | 532 | 71000 | SWW (xpEHH 3.35, 98/98) | xpEHH | win | SWW | 98 | 3.352 | 2.934 |
| css576 | 3B | 461 | 532 | 71000 | WA (xpEHH 2.90, 97/97) | xpEHH | win | WA | 97 | 2.895 | 2.439 |
| css577 | 3B | 461 | 532 | 71000 | WA (xpEHH 3.55, 115/115) | xpEHH | both | WA | 115 | 3.553 | 2.927 |
| css578 | 3B | 466 | 487 | 21000 | SWW (Rsb 2.78, 24/25) | Rsb | win | SWW | 24 | 2.784 | 2.524 |
| css579 | 3B | 466 | 475 | 9000 | WA (Rsb 2.56, 7/7) | Rsb | win | WA | 7 | 2.561 | 2.453 |
| css580 | 3B | 467 | 473 | 6000 | ID (Rsb 2.29, 9/10) | Rsb | both | ID | 9 | 2.289 | 2.180 |
| css581 | 3B | 469 | 498 | 29000 | OR (xpEHH 2.71, 51/53) | xpEHH | win | OR | 51 | 2.709 | 2.372 |
| css582 | 3B | 469 | 475 | 6000 | pac2 (Fst 0.42, 7/7) | Fst | both | pac2 | 7 | 0.416 | 0.416 |
| css583 | 3B | 469 | 475 | 6000 | pac2 (Fst 0.43, 7/7) | Fst | spr | pac2 | 7 | 0.432 | 0.432 |
| css584 | 3B | 469 | 488 | 19000 | pnw (Rsb 3.10, 28/29) | Rsb | win | pnw | 28 | 3.100 | 2.564 |
| css585 | 3B | 469 | 487 | 18000 | WA (Rsb 3.24, 26/26) | Rsb | both | WA | 26 | 3.242 | 2.860 |
| css586 | 3B | 475 | 516 | 41000 | ID (Rsb 3.16, 80/81) | Rsb | both | ID | 80 | 3.159 | 2.406 |
| css587 | 3B | 477 | 487 | 10000 | WA (Rsb 2.41, 15/15) | Rsb | win | WA | 15 | 2.414 | 2.347 |
| css588 | 3B | 489 | 496 | 7000 | gpl (Fst 0.35, 20/22) | Fst | spr | gpl | 20 | 0.351 | 0.307 |
| css589 | 3B | 489 | 496 | 7000 | MN (Fst 0.31, 21/22) | Fst | spr | MN | 21 | 0.310 | 0.302 |
| css590 | 3B | 496 | 501 | 5000 | pnw (Rsb 2.46, 10/10) | Rsb | win | pnw | 10 | 2.456 | 2.115 |
| css591 | 3B | 496 | 532 | 36000 | SWW (Rsb 3.79, 54/55) | Rsb | win | SWW | 54 | 3.786 | 2.892 |
| css592 | 3B | 496 | 516 | 20000 | WA (Rsb 3.15, 36/37) | Rsb | both | WA | 36 | 3.148 | 2.646 |
| css593 | 3B | 501 | 516 | 15000 | MN (xpEHH 2.42, 26/27) | xpEHH | both | MN | 26 | 2.423 | 2.227 |
| css594 | 3B | 503 | 511 | 8000 | pnw (Rsb 3.58, 9/9) | Rsb | win | pnw | 9 | 3.581 | 2.767 |
| css595 | 3B | 503 | 511 | 8000 | WA (Rsb 2.95, 8/8) | Rsb | win | WA | 8 | 2.948 | 2.429 |
| css596 | 3B | 505 | 510 | 5000 | CA (xpEHH 2.26, 5/5) | xpEHH | both | CA | 5 | 2.258 | 2.168 |
| css597 | 3B | 505 | 510 | 5000 | OR (Rsb 2.39, 5/5) | Rsb | win | OR | 5 | 2.390 | 2.306 |
| css598 | 3B | 505 | 516 | 11000 | pnw (xpEHH 2.87, 18/19) | xpEHH | win | pnw | 18 | 2.874 | 2.394 |
| css599 | 3B | 508 | 520 | 12000 | gpl (Fst 0.35, 17/17) | Fst | spr | gpl | 17 | 0.355 | 0.284 |
| css600 | 3B | 508 | 514 | 6000 | OR (xpEHH 2.41, 8/8) | xpEHH | win | OR | 8 | 2.414 | 2.160 |
| css601 | 3B | 513 | 520 | 7000 | MN (Rsb 3.07, 9/9) | Rsb | both | MN | 9 | 3.074 | 2.497 |
| css602 | 3B | 520 | 532 | 12000 | MN (xpEHH 2.40, 16/16) | xpEHH | both | MN | 16 | 2.399 | 2.179 |
| css603 | 3B | 520 | 532 | 12000 | OR (Rsb 2.77, 16/16) | Rsb | win | OR | 16 | 2.768 | 2.478 |
| css604 | 3B | 520 | 532 | 12000 | pnw (Rsb 3.37, 16/16) | Rsb | win | pnw | 16 | 3.370 | 2.921 |
| css605 | 3B | 520 | 532 | 12000 | pnw (Rsb 3.68, 16/16) | Rsb | both | pnw | 16 | 3.685 | 3.240 |
| css606 | 3B | 520 | 532 | 12000 | WA (Rsb 3.08, 16/16) | Rsb | win | WA | 16 | 3.079 | 2.618 |
| css607 | 3B | 520 | 532 | 12000 | WA (Rsb 3.57, 16/16) | Rsb | both | WA | 16 | 3.573 | 3.125 |
| css608 | 3B | 521 | 532 | 11000 | ID (Rsb 2.80, 13/13) | Rsb | both | ID | 13 | 2.804 | 2.470 |
| css609 | 3B | 521 | 532 | 11000 | OR (xpEHH 2.43, 13/13) | xpEHH | win | OR | 13 | 2.425 | 2.300 |
| css610 | 3B | 521 | 532 | 11000 | pnw (xpEHH 2.52, 13/13) | xpEHH | win | pnw | 13 | 2.523 | 2.362 |
| css611 | 3B | 523 | 532 | 9000 | gpl (Fst 0.26, 12/13) | Fst | spr | gpl | 12 | 0.263 | 0.236 |
| css612 | 3B | 527 | 532 | 5000 | CO (Fst 0.24, 6/6) | Fst | both | CO | 6 | 0.241 | 0.191 |
| css613 | 3B | 527 | 532 | 5000 | MN (Rsb 2.25, 6/6) | Rsb | both | MN | 6 | 2.250 | 2.145 |
| css614 | 3B | 533 | 541 | 8000 | CO (Fst 0.24, 12/13) | Fst | both | CO | 12 | 0.236 | 0.185 |
| css615 | 3B | 533 | 541 | 8000 | gpl (Fst 0.26, 12/13) | Fst | spr | gpl | 12 | 0.265 | 0.238 |
| css616 | 3B | 533 | 543 | 10000 | ID (Rsb 2.79, 14/14) | Rsb | both | ID | 14 | 2.792 | 2.464 |
| css617 | 3B | 533 | 541 | 8000 | ID (xpEHH 2.22, 12/13) | xpEHH | both | ID | 12 | 2.217 | 2.092 |
| css618 | 3B | 533 | 541 | 8000 | MN (xpEHH 2.31, 12/13) | xpEHH | both | MN | 12 | 2.312 | 2.145 |
| css619 | 3B | 533 | 543 | 10000 | OR (Rsb 2.62, 12/12) | Rsb | win | OR | 12 | 2.616 | 2.296 |
| css620 | 3B | 533 | 543 | 10000 | OR (xpEHH 2.42, 12/12) | xpEHH | win | OR | 12 | 2.418 | 2.164 |
| css621 | 3B | 533 | 543 | 10000 | pnw (Rsb 3.11, 14/14) | Rsb | win | pnw | 14 | 3.106 | 2.726 |
| css622 | 3B | 533 | 543 | 10000 | pnw (Rsb 3.67, 14/14) | Rsb | both | pnw | 14 | 3.674 | 3.200 |
| css623 | 3B | 533 | 541 | 8000 | pnw (xpEHH 2.51, 12/13) | xpEHH | win | pnw | 12 | 2.513 | 2.206 |
| css624 | 3B | 533 | 543 | 10000 | pnw (xpEHH 2.75, 14/14) | xpEHH | both | pnw | 14 | 2.747 | 2.515 |
| css625 | 3B | 533 | 543 | 10000 | SWW (Rsb 3.32, 14/14) | Rsb | win | SWW | 14 | 3.316 | 2.968 |
| css626 | 3B | 533 | 543 | 10000 | SWW (xpEHH 3.18, 14/14) | xpEHH | win | SWW | 14 | 3.184 | 2.852 |
| css627 | 3B | 533 | 543 | 10000 | WA (Rsb 2.84, 14/14) | Rsb | win | WA | 14 | 2.842 | 2.381 |
| css628 | 3B | 533 | 543 | 10000 | WA (Rsb 3.42, 14/14) | Rsb | both | WA | 14 | 3.419 | 3.051 |
| css629 | 3B | 533 | 541 | 8000 | WA (xpEHH 2.56, 12/13) | xpEHH | win | WA | 12 | 2.558 | 2.320 |
| css630 | 3B | 533 | 543 | 10000 | WA (xpEHH 2.88, 14/14) | xpEHH | both | WA | 14 | 2.879 | 2.615 |
| css631 | 3B | 554 | 559 | 5000 | CO (Fst 0.40, 5/5) | Fst | both | CO | 5 | 0.396 | 0.234 |
| css632 | 3B | 554 | 585 | 31000 | ID (Rsb 4.29, 48/48) | Rsb | both | ID | 48 | 4.289 | 3.037 |
| css633 | 3B | 554 | 587 | 33000 | ID (xpEHH 3.93, 51/51) | xpEHH | both | ID | 51 | 3.926 | 3.248 |
| css634 | 3B | 554 | 563 | 9000 | MN (Rsb 3.10, 11/12) | Rsb | both | MN | 11 | 3.103 | 2.414 |
| css635 | 3B | 554 | 563 | 9000 | MN (xpEHH 2.62, 11/12) | xpEHH | both | MN | 11 | 2.621 | 2.400 |
| css636 | 3B | 554 | 588 | 34000 | OR (xpEHH 4.01, 45/45) | xpEHH | win | OR | 45 | 4.006 | 3.257 |
| css637 | 3B | 554 | 588 | 34000 | pnw (Rsb 4.71, 53/53) | Rsb | win | pnw | 53 | 4.706 | 3.198 |
| css638 | 3B | 554 | 588 | 34000 | pnw (Rsb 5.63, 54/54) | Rsb | both | pnw | 54 | 5.632 | 4.035 |
| css639 | 3B | 554 | 608 | 54000 | pnw (xpEHH 4.64, 100/100) | xpEHH | win | pnw | 100 | 4.637 | 3.066 |
| css640 | 3B | 554 | 608 | 54000 | pnw (xpEHH 5.52, 107/107) | xpEHH | both | pnw | 107 | 5.519 | 3.557 |
| css641 | 3B | 554 | 615 | 61000 | SWW (Rsb 4.40, 107/107) | Rsb | win | SWW | 107 | 4.404 | 3.004 |
| css642 | 3B | 554 | 622 | 68000 | SWW (xpEHH 4.86, 114/114) | xpEHH | win | SWW | 114 | 4.856 | 3.392 |
| css643 | 3B | 554 | 615 | 61000 | WA (Rsb 4.92, 106/106) | Rsb | both | WA | 106 | 4.916 | 3.239 |
| css644 | 3B | 554 | 564 | 10000 | WA (xpEHH 3.36, 12/12) | xpEHH | win | WA | 12 | 3.358 | 2.946 |
| css645 | 3B | 554 | 620 | 66000 | WA (xpEHH 5.06, 112/112) | xpEHH | both | WA | 112 | 5.059 | 3.547 |
| css646 | 3B | 555 | 563 | 8000 | ID (Rsb 3.46, 10/12) | Rsb | win | ID | 10 | 3.461 | 2.752 |
| css647 | 3B | 555 | 564 | 9000 | WA (Rsb 3.52, 11/12) | Rsb | win | WA | 11 | 3.518 | 2.727 |
| css648 | 3B | 564 | 573 | 9000 | MN (Rsb 3.72, 15/16) | Rsb | both | MN | 15 | 3.718 | 2.693 |
| css649 | 3B | 566 | 593 | 27000 | WA (Rsb 3.56, 43/43) | Rsb | win | WA | 43 | 3.564 | 2.685 |
| css650 | 3B | 566 | 615 | 49000 | WA (xpEHH 4.01, 81/82) | xpEHH | win | WA | 81 | 4.014 | 2.860 |
| css651 | 3B | 567 | 573 | 6000 | MN (xpEHH 2.80, 13/15) | xpEHH | both | MN | 13 | 2.798 | 2.378 |
| css652 | 3B | 569 | 585 | 16000 | ID (xpEHH 2.85, 26/27) | xpEHH | win | ID | 26 | 2.852 | 2.542 |
| css653 | 3B | 569 | 587 | 18000 | OR (Rsb 4.05, 25/27) | Rsb | win | OR | 25 | 4.051 | 3.065 |
| css654 | 3B | 574 | 582 | 8000 | MN (xpEHH 2.45, 15/16) | xpEHH | both | MN | 15 | 2.455 | 2.134 |
| css655 | 3B | 577 | 582 | 5000 | ID (Rsb 3.38, 6/6) | Rsb | win | ID | 6 | 3.380 | 3.068 |
| css656 | 3B | 577 | 585 | 8000 | pnw (Fst 0.18, 10/10) | Fst | both | pnw | 10 | 0.178 | 0.142 |
| css657 | 3B | 577 | 582 | 5000 | WA (Fst 0.23, 8/8) | Fst | both | WA | 8 | 0.233 | 0.193 |
| css658 | 3B | 578 | 585 | 7000 | WA (Fst 0.20, 8/8) | Fst | spr | WA | 8 | 0.197 | 0.183 |
| css659 | 3B | 580 | 585 | 5000 | pnw (Fst 0.24, 7/7) | Fst | spr | pnw | 7 | 0.238 | 0.219 |
| css660 | 3B | 588 | 620 | 32000 | WA (xpEHH 2.78, 52/52) | xpEHH | spr | WA | 52 | 2.778 | 2.286 |
| css661 | 3B | 592 | 612 | 20000 | pnw (Rsb 3.48, 51/52) | Rsb | both | pnw | 51 | 3.475 | 2.752 |
| css662 | 3B | 596 | 601 | 5000 | pnw (Rsb 2.97, 17/18) | Rsb | win | pnw | 17 | 2.968 | 2.419 |
| css663 | 3B | 602 | 615 | 13000 | OR (xpEHH 2.12, 14/14) | xpEHH | win | OR | 14 | 2.124 | 2.064 |
| css664 | 3B | 606 | 615 | 9000 | WA (Rsb 2.99, 10/11) | Rsb | spr | WA | 10 | 2.990 | 2.713 |
| css665 | 3B | 677 | 684 | 7000 | CO (Rsb 2.52, 11/12) | Rsb | win | CO | 11 | 2.520 | 2.342 |
| css666 | 3B | 678 | 684 | 6000 | SWW (Rsb 2.33, 10/11) | Rsb | win | SWW | 10 | 2.327 | 2.194 |
| css667 | 3B | 681 | 686 | 5000 | OR (Rsb 2.58, 12/12) | Rsb | win | OR | 12 | 2.578 | 2.304 |
| css668 | 3B | 688 | 694 | 6000 | eas (Fst 0.31, 5/5) | Fst | both | eas | 5 | 0.306 | 0.278 |
| css669 | 3B | 688 | 694 | 6000 | IN (Fst 0.40, 5/5) | Fst | both | IN | 5 | 0.403 | 0.379 |
| css670 | 3B | 688 | 694 | 6000 | SC (Fst 0.63, 5/5) | Fst | both | SC | 5 | 0.631 | 0.528 |
| css671 | 3B | 702 | 709 | 7000 | HRS (Fst 0.55, 11/11) | Fst | spr | HRS | 11 | 0.548 | 0.472 |
| css672 | 3B | 703 | 711 | 8000 | SWS (Fst 0.49, 10/10) | Fst | spr | SWS | 10 | 0.494 | 0.408 |
| css673 | 3B | 715 | 722 | 7000 | HRS (Fst 0.53, 9/9) | Fst | spr | HRS | 9 | 0.530 | 0.467 |
| css674 | 3B | 715 | 722 | 7000 | SWS (Fst 0.50, 9/9) | Fst | spr | SWS | 9 | 0.502 | 0.416 |
| css675 | 3B | 719 | 728 | 9000 | nor (Fst 0.32, 15/16) | Fst | both | nor | 15 | 0.315 | 0.255 |
| css676 | 3B | 720 | 728 | 8000 | MN (Fst 0.47, 12/12) | Fst | both | MN | 12 | 0.475 | 0.443 |
| css677 | 3B | 720 | 728 | 8000 | nor (Fst 0.25, 12/12) | Fst | win | nor | 12 | 0.251 | 0.225 |
| css678 | 3B | 756 | 762 | 6000 | OR (Rsb 3.01, 5/5) | Rsb | win | OR | 5 | 3.012 | 2.496 |
| css679 | 3B | 756 | 762 | 6000 | SWW (Rsb 2.86, 5/5) | Rsb | win | SWW | 5 | 2.863 | 2.279 |
| css680 | 3B | 763 | 772 | 9000 | OR (Rsb 2.77, 9/9) | Rsb | win | OR | 9 | 2.766 | 2.459 |
| css681 | 3D | 29 | 38 | 9000 | MN (Fst 0.30, 10/10) | Fst | spr | MN | 10 | 0.301 | 0.297 |
| css682 | 3D | 29 | 38 | 9000 | nor (Fst 0.24, 10/10) | Fst | spr | nor | 10 | 0.240 | 0.236 |
| css683 | 3D | 32 | 38 | 6000 | MN (Fst 0.37, 9/9) | Fst | both | MN | 9 | 0.369 | 0.367 |
| css684 | 3D | 32 | 38 | 6000 | ND (Fst 0.40, 9/9) | Fst | both | ND | 9 | 0.405 | 0.403 |
| css685 | 3D | 32 | 38 | 6000 | nor (Fst 0.27, 9/9) | Fst | both | nor | 9 | 0.267 | 0.265 |
| css686 | 3D | 32 | 38 | 6000 | pnw (Fst 0.20, 9/9) | Fst | spr | pnw | 9 | 0.197 | 0.197 |
| css687 | 3D | 32 | 38 | 6000 | TX (Fst 0.16, 9/9) | Fst | both | TX | 9 | 0.163 | 0.162 |
| css688 | 3D | 619 | 624 | 5000 | eas2 (Fst 0.18, 9/10) | Fst | both | eas2 | 9 | 0.179 | 0.159 |
| css689 | 4A | 34 | 43 | 9000 | KS (xpEHH 2.82, 14/15) | xpEHH | win | KS | 14 | 2.819 | 2.494 |
| css690 | 4A | 34 | 51 | 17000 | MN (xpEHH 2.45, 21/21) | xpEHH | both | MN | 21 | 2.455 | 2.245 |
| css691 | 4A | 34 | 40 | 6000 | nor (Rsb 2.51, 5/5) | Rsb | both | nor | 5 | 2.509 | 2.431 |
| css692 | 4A | 34 | 51 | 17000 | nor (xpEHH 2.93, 22/22) | xpEHH | spr | nor | 22 | 2.932 | 2.617 |
| css693 | 4A | 34 | 40 | 6000 | pnw2 (xpEHH 2.71, 9/9) | xpEHH | win | pnw2 | 9 | 2.711 | 2.428 |
| css694 | 4A | 38 | 51 | 13000 | KS (xpEHH 2.76, 18/18) | xpEHH | both | KS | 18 | 2.755 | 2.416 |
| css695 | 4A | 42 | 51 | 9000 | HRS (xpEHH 2.25, 7/7) | xpEHH | spr | HRS | 7 | 2.251 | 2.144 |
| css696 | 4A | 42 | 51 | 9000 | nor (Fst 0.26, 7/7) | Fst | both | nor | 7 | 0.259 | 0.241 |
| css697 | 4A | 42 | 51 | 9000 | SWS (Fst 0.50, 7/7) | Fst | spr | SWS | 7 | 0.499 | 0.417 |
| css698 | 4A | 56 | 61 | 5000 | MN (xpEHH 2.78, 5/5) | xpEHH | spr | MN | 5 | 2.783 | 2.614 |
| css699 | 4A | 56 | 61 | 5000 | nor (xpEHH 2.46, 5/5) | xpEHH | spr | nor | 5 | 2.458 | 2.316 |
| css700 | 4A | 57 | 64 | 7000 | KS (xpEHH 2.67, 5/5) | xpEHH | both | KS | 5 | 2.673 | 2.626 |
| css701 | 4A | 83 | 93 | 10000 | pnw2 (Fst 0.24, 13/13) | Fst | win | pnw2 | 13 | 0.245 | 0.192 |
| css702 | 4A | 98 | 129 | 31000 | pac (Fst 0.42, 56/57) | Fst | both | pac | 56 | 0.422 | 0.359 |
| css703 | 4A | 104 | 125 | 21000 | CA (Fst 0.39, 37/38) | Fst | both | CA | 37 | 0.392 | 0.332 |
| css704 | 4A | 132 | 138 | 6000 | CA (Fst 0.33, 9/9) | Fst | both | CA | 9 | 0.334 | 0.327 |
| css705 | 4A | 132 | 138 | 6000 | pac (Fst 0.37, 9/9) | Fst | both | pac | 9 | 0.369 | 0.363 |
| css706 | 4A | 160 | 165 | 5000 | nor (Fst 0.21, 6/6) | Fst | win | nor | 6 | 0.209 | 0.209 |
| css707 | 4A | 558 | 565 | 7000 | gpl2 (Fst 0.13, 5/5) | Fst | win | gpl2 | 5 | 0.135 | 0.121 |
| css708 | 4A | 578 | 588 | 10000 | ND (Fst 0.60, 25/27) | Fst | both | ND | 25 | 0.604 | 0.490 |
| css709 | 4A | 578 | 583 | 5000 | nor (Fst 0.23, 14/15) | Fst | both | nor | 14 | 0.225 | 0.218 |
| css710 | 4A | 622 | 629 | 7000 | ND (Fst 0.72, 10/11) | Fst | spr | ND | 10 | 0.718 | 0.385 |
| css711 | 4A | 647 | 652 | 5000 | SWW2 (xpEHH 2.17, 5/5) | xpEHH | win | SWW2 | 5 | 2.170 | 2.146 |
| css712 | 4A | 654 | 659 | 5000 | ND (Fst 0.61, 5/5) | Fst | spr | ND | 5 | 0.609 | 0.383 |
| css713 | 4A | 718 | 724 | 6000 | SWW2 (Rsb 3.37, 17/18) | Rsb | win | SWW2 | 17 | 3.373 | 2.807 |
| css714 | 4A | 718 | 724 | 6000 | SWW2 (xpEHH 3.29, 17/18) | xpEHH | win | SWW2 | 17 | 3.294 | 2.642 |
| css715 | 4A | 718 | 724 | 6000 | win2 (xpEHH 3.83, 20/21) | xpEHH | win | win2 | 20 | 3.827 | 3.297 |
| css716 | 4B | 11 | 17 | 6000 | pnw (Rsb 3.32, 14/15) | Rsb | win | pnw | 14 | 3.318 | 2.693 |
| css717 | 4B | 15 | 24 | 9000 | gpl2 (xpEHH 3.52, 27/29) | xpEHH | win | gpl2 | 27 | 3.524 | 2.659 |
| css718 | 4B | 59 | 71 | 12000 | gpl (Fst 0.33, 16/16) | Fst | spr | gpl | 16 | 0.334 | 0.334 |
| css719 | 4B | 94 | 103 | 9000 | ND (Fst 0.37, 7/7) | Fst | spr | ND | 7 | 0.374 | 0.347 |
| css720 | 4B | 94 | 103 | 9000 | NY (Fst 0.54, 7/7) | Fst | win | NY | 7 | 0.541 | 0.534 |
| css721 | 4B | 419 | 424 | 5000 | HRS2 (Fst 0.18, 5/5) | Fst | spr | HRS2 | 5 | 0.182 | 0.170 |
| css722 | 4B | 419 | 424 | 5000 | nor2 (Fst 0.36, 5/5) | Fst | spr | nor2 | 5 | 0.361 | 0.296 |
| css723 | 4B | 419 | 431 | 12000 | NY (Fst 0.44, 14/14) | Fst | win | NY | 14 | 0.444 | 0.431 |
| css724 | 4B | 419 | 427 | 8000 | SWW (Fst 0.30, 12/12) | Fst | win | SWW | 12 | 0.302 | 0.286 |
| css725 | 4B | 493 | 501 | 8000 | ID (Fst 0.30, 7/7) | Fst | spr | ID | 7 | 0.297 | 0.271 |
| css726 | 4B | 493 | 501 | 8000 | pnw (Fst 0.27, 7/7) | Fst | spr | pnw | 7 | 0.274 | 0.247 |
| css727 | 4B | 493 | 501 | 8000 | SWS (Fst 0.43, 7/7) | Fst | spr | SWS | 7 | 0.426 | 0.396 |
| css728 | 4B | 523 | 529 | 6000 | gpl2 (xpEHH 2.23, 8/8) | xpEHH | both | gpl2 | 8 | 2.226 | 2.204 |
| css729 | 4B | 523 | 529 | 6000 | gpl2 (xpEHH 2.35, 8/8) | xpEHH | win | gpl2 | 8 | 2.353 | 2.335 |
| css730 | 4B | 534 | 547 | 13000 | nor (Fst 0.22, 30/30) | Fst | win | nor | 30 | 0.215 | 0.201 |
| css731 | 4B | 535 | 557 | 22000 | MT (Fst 0.34, 44/44) | Fst | both | MT | 44 | 0.336 | 0.294 |
| css732 | 4B | 535 | 547 | 12000 | SWS2 (Fst 0.41, 22/22) | Fst | spr | SWS2 | 22 | 0.412 | 0.397 |
| css733 | 4B | 535 | 543 | 8000 | SWW (Fst 0.25, 15/15) | Fst | win | SWW | 15 | 0.250 | 0.247 |
| css734 | 4B | 540 | 547 | 7000 | HRW2 (Fst 0.10, 9/10) | Fst | win | HRW2 | 9 | 0.096 | 0.096 |
| css735 | 4B | 540 | 547 | 7000 | ID (Fst 0.25, 10/10) | Fst | spr | ID | 10 | 0.254 | 0.254 |
| css736 | 4B | 540 | 547 | 7000 | nor (Fst 0.21, 10/10) | Fst | both | nor | 10 | 0.207 | 0.203 |
| css737 | 4B | 540 | 547 | 7000 | nor (Fst 0.26, 10/10) | Fst | spr | nor | 10 | 0.260 | 0.260 |
| css738 | 4B | 540 | 547 | 7000 | pnw (Fst 0.23, 10/10) | Fst | spr | pnw | 10 | 0.231 | 0.231 |
| css739 | 4B | 545 | 552 | 7000 | gpl (Fst 0.15, 10/11) | Fst | both | gpl | 10 | 0.151 | 0.151 |
| css740 | 4B | 545 | 552 | 7000 | gpl (Fst 0.24, 10/11) | Fst | win | gpl | 10 | 0.236 | 0.234 |
| css741 | 4B | 545 | 552 | 7000 | HRW (Fst 0.31, 10/11) | Fst | win | HRW | 10 | 0.312 | 0.307 |
| css742 | 4B | 545 | 552 | 7000 | KS (Fst 0.21, 10/11) | Fst | win | KS | 10 | 0.207 | 0.200 |
| css743 | 4B | 545 | 552 | 7000 | NY (Fst 0.58, 10/11) | Fst | win | NY | 10 | 0.578 | 0.572 |
| css744 | 4B | 545 | 552 | 7000 | SWW (Fst 0.31, 10/11) | Fst | win | SWW | 10 | 0.308 | 0.277 |
| css745 | 4B | 552 | 557 | 5000 | nor (Fst 0.22, 11/11) | Fst | win | nor | 11 | 0.215 | 0.209 |
| css746 | 4B | 552 | 557 | 5000 | pnw (Fst 0.23, 11/11) | Fst | spr | pnw | 11 | 0.227 | 0.207 |
| css747 | 4B | 552 | 557 | 5000 | SWS2 (Fst 0.41, 11/11) | Fst | spr | SWS2 | 11 | 0.412 | 0.412 |
| css748 | 4B | 574 | 583 | 9000 | SC (Fst 0.87, 13/14) | Fst | both | SC | 13 | 0.866 | 0.703 |
| css749 | 4B | 574 | 583 | 9000 | SC (Fst 0.87, 13/14) | Fst | win | SC | 13 | 0.873 | 0.793 |
| css750 | 4B | 589 | 594 | 5000 | SC (Fst 0.68, 7/7) | Fst | win | SC | 7 | 0.683 | 0.673 |
| css751 | 4B | 595 | 602 | 7000 | nor (Fst 0.29, 9/9) | Fst | spr | nor | 9 | 0.289 | 0.239 |
| css752 | 4B | 595 | 600 | 5000 | SC (Fst 0.61, 6/6) | Fst | both | SC | 6 | 0.612 | 0.584 |
| css753 | 4B | 619 | 625 | 6000 | OR (Fst 0.47, 6/6) | Fst | win | OR | 6 | 0.471 | 0.357 |
| css754 | 4D | 23 | 31 | 8000 | NY (Fst 0.51, 5/5) | Fst | win | NY | 5 | 0.505 | 0.502 |
| css755 | 5A | 20 | 42 | 22000 | ND (xpEHH 3.49, 33/34) | xpEHH | both | ND | 33 | 3.492 | 2.664 |
| css756 | 5A | 22 | 27 | 5000 | eas (Fst 0.41, 7/7) | Fst | win | eas | 7 | 0.406 | 0.392 |
| css757 | 5A | 22 | 27 | 5000 | IN (Fst 0.56, 7/7) | Fst | both | IN | 7 | 0.564 | 0.546 |
| css758 | 5A | 22 | 27 | 5000 | IN (Fst 0.72, 7/7) | Fst | win | IN | 7 | 0.722 | 0.709 |
| css759 | 5A | 22 | 27 | 5000 | SRW (Fst 0.44, 7/7) | Fst | win | SRW | 7 | 0.444 | 0.428 |
| css760 | 5A | 26 | 39 | 13000 | ND (Rsb 2.78, 15/15) | Rsb | both | ND | 15 | 2.781 | 2.276 |
| css761 | 5A | 32 | 42 | 10000 | ID (xpEHH 2.63, 17/17) | xpEHH | win | ID | 17 | 2.628 | 2.292 |
| css762 | 5A | 33 | 38 | 5000 | nor (Rsb 3.14, 11/12) | Rsb | spr | nor | 11 | 3.139 | 2.421 |
| css763 | 5A | 40 | 48 | 8000 | ND (xpEHH 2.43, 7/7) | xpEHH | spr | ND | 7 | 2.430 | 2.401 |
| css764 | 5A | 74 | 80 | 6000 | gpl2 (Fst 0.13, 8/8) | Fst | both | gpl2 | 8 | 0.128 | 0.128 |
| css765 | 5A | 378 | 384 | 6000 | eas (Fst 0.27, 5/5) | Fst | both | eas | 5 | 0.274 | 0.269 |
| css766 | 5A | 378 | 384 | 6000 | eas2 (Fst 0.17, 5/5) | Fst | win | eas2 | 5 | 0.172 | 0.160 |
| css767 | 5A | 378 | 384 | 6000 | eas2 (Fst 0.18, 5/5) | Fst | both | eas2 | 5 | 0.179 | 0.167 |
| css768 | 5A | 378 | 386 | 8000 | HRW2 (Fst 0.14, 7/7) | Fst | win | HRW2 | 7 | 0.139 | 0.133 |
| css769 | 5A | 378 | 386 | 8000 | SC (Fst 0.54, 7/7) | Fst | both | SC | 7 | 0.543 | 0.517 |
| css770 | 5A | 378 | 384 | 6000 | SRW (Fst 0.27, 5/5) | Fst | win | SRW | 5 | 0.271 | 0.266 |
| css771 | 5A | 434 | 439 | 5000 | ID (Fst 0.23, 8/8) | Fst | win | ID | 8 | 0.232 | 0.204 |
| css772 | 5A | 434 | 462 | 28000 | ND (xpEHH 3.03, 49/50) | xpEHH | spr | ND | 49 | 3.034 | 2.615 |
| css773 | 5A | 434 | 462 | 28000 | pnw2 (Rsb 3.89, 47/49) | Rsb | win | pnw2 | 47 | 3.887 | 2.790 |
| css774 | 5A | 435 | 444 | 9000 | HRW (xpEHH 2.66, 14/15) | xpEHH | win | HRW | 14 | 2.658 | 2.329 |
| css775 | 5A | 435 | 459 | 24000 | ND (xpEHH 3.05, 46/48) | xpEHH | both | ND | 46 | 3.048 | 2.444 |
| css776 | 5A | 446 | 456 | 10000 | HRW (Rsb 2.75, 14/14) | Rsb | win | HRW | 14 | 2.751 | 2.369 |
| css777 | 5A | 446 | 493 | 47000 | pnw2 (xpEHH 3.54, 199/199) | xpEHH | win | pnw2 | 199 | 3.536 | 3.038 |
| css778 | 5A | 447 | 457 | 10000 | nor (xpEHH 2.29, 15/16) | xpEHH | spr | nor | 15 | 2.290 | 2.175 |
| css779 | 5A | 452 | 457 | 5000 | HRW (xpEHH 2.57, 5/5) | xpEHH | win | HRW | 5 | 2.569 | 2.406 |
| css780 | 5A | 452 | 457 | 5000 | nor (xpEHH 2.23, 5/5) | xpEHH | both | nor | 5 | 2.233 | 2.124 |
| css781 | 5A | 453 | 459 | 6000 | ND (Rsb 2.92, 13/14) | Rsb | spr | ND | 13 | 2.917 | 2.435 |
| css782 | 5A | 453 | 459 | 6000 | ND (Rsb 3.41, 13/14) | Rsb | both | ND | 13 | 3.411 | 2.740 |
| css783 | 5A | 458 | 472 | 14000 | eas (Fst 0.50, 102/108) | Fst | win | eas | 102 | 0.496 | 0.449 |
| css784 | 5A | 458 | 470 | 12000 | SWW2 (Fst 0.24, 81/89) | Fst | win | SWW2 | 81 | 0.244 | 0.214 |
| css785 | 5A | 459 | 472 | 13000 | CO (Fst 0.34, 94/105) | Fst | win | CO | 94 | 0.335 | 0.326 |
| css786 | 5A | 459 | 470 | 11000 | gpl (Fst 0.24, 81/86) | Fst | both | gpl | 81 | 0.237 | 0.209 |
| css787 | 5A | 459 | 472 | 13000 | gpl (Fst 0.31, 95/105) | Fst | win | gpl | 95 | 0.315 | 0.299 |
| css788 | 5A | 459 | 464 | 5000 | HRS2 (Fst 0.19, 30/32) | Fst | spr | HRS2 | 30 | 0.189 | 0.180 |
| css789 | 5A | 459 | 472 | 13000 | HRW (Fst 0.56, 99/105) | Fst | win | HRW | 99 | 0.558 | 0.510 |
| css790 | 5A | 459 | 472 | 13000 | IN (Fst 0.72, 100/105) | Fst | win | IN | 100 | 0.722 | 0.555 |
| css791 | 5A | 459 | 468 | 9000 | KS (Fst 0.18, 63/70) | Fst | both | KS | 63 | 0.179 | 0.169 |
| css792 | 5A | 459 | 464 | 5000 | KS (Fst 0.21, 30/32) | Fst | win | KS | 30 | 0.206 | 0.201 |
| css793 | 5A | 459 | 470 | 11000 | NE (Fst 0.32, 79/86) | Fst | both | NE | 79 | 0.320 | 0.282 |
| css794 | 5A | 459 | 470 | 11000 | NE (Fst 0.42, 79/86) | Fst | win | NE | 79 | 0.420 | 0.401 |
| css795 | 5A | 459 | 472 | 13000 | nor (Fst 0.27, 94/105) | Fst | win | nor | 94 | 0.272 | 0.217 |
| css796 | 5A | 459 | 472 | 13000 | nor2 (Fst 0.37, 100/105) | Fst | both | nor2 | 100 | 0.369 | 0.213 |
| css797 | 5A | 459 | 472 | 13000 | nor2 (Fst 0.43, 100/105) | Fst | spr | nor2 | 100 | 0.428 | 0.405 |
| css798 | 5A | 459 | 470 | 11000 | NY (Fst 0.56, 79/86) | Fst | win | NY | 79 | 0.559 | 0.525 |
| css799 | 5A | 459 | 472 | 13000 | pnw2 (Fst 0.21, 98/105) | Fst | win | pnw2 | 98 | 0.215 | 0.212 |
| css800 | 5A | 459 | 464 | 5000 | SC (Fst 0.61, 30/32) | Fst | win | SC | 30 | 0.608 | 0.600 |
| css801 | 5A | 459 | 472 | 13000 | SRW (Fst 0.50, 98/105) | Fst | win | SRW | 98 | 0.498 | 0.435 |
| css802 | 5A | 463 | 472 | 9000 | IN (Fst 0.60, 76/85) | Fst | both | IN | 76 | 0.600 | 0.334 |
| css803 | 5A | 464 | 470 | 6000 | pnw2 (Rsb 2.37, 49/52) | Rsb | win | pnw2 | 49 | 2.371 | 2.270 |
| css804 | 5A | 465 | 472 | 7000 | eas (Fst 0.32, 54/60) | Fst | both | eas | 54 | 0.324 | 0.229 |
| css805 | 5A | 465 | 472 | 7000 | SC (Fst 0.66, 53/60) | Fst | win | SC | 53 | 0.660 | 0.610 |
| css806 | 5A | 467 | 472 | 5000 | HRS2 (Fst 0.22, 48/53) | Fst | spr | HRS2 | 48 | 0.217 | 0.206 |
| css807 | 5A | 471 | 482 | 11000 | ND (Rsb 2.89, 50/53) | Rsb | spr | ND | 50 | 2.893 | 2.454 |
| css808 | 5A | 471 | 482 | 11000 | ND (Rsb 3.57, 52/53) | Rsb | both | ND | 52 | 3.572 | 2.558 |
| css809 | 5A | 471 | 486 | 15000 | ND (xpEHH 2.60, 62/64) | xpEHH | both | ND | 62 | 2.598 | 2.375 |
| css810 | 5A | 471 | 493 | 22000 | ND (xpEHH 2.75, 78/78) | xpEHH | spr | ND | 78 | 2.747 | 2.382 |
| css811 | 5A | 473 | 482 | 9000 | ID (xpEHH 2.99, 45/45) | xpEHH | win | ID | 45 | 2.993 | 2.729 |
| css812 | 5A | 473 | 479 | 6000 | nor2 (Fst 0.43, 32/36) | Fst | spr | nor2 | 32 | 0.429 | 0.384 |
| css813 | 5A | 475 | 493 | 18000 | pnw2 (Rsb 4.19, 52/55) | Rsb | win | pnw2 | 52 | 4.193 | 2.491 |
| css814 | 5A | 477 | 482 | 5000 | ID (Rsb 2.86, 15/16) | Rsb | win | ID | 15 | 2.859 | 2.252 |
| css815 | 5A | 481 | 486 | 5000 | HRW2 (xpEHH 2.19, 10/11) | xpEHH | win | HRW2 | 10 | 2.191 | 2.146 |
| css816 | 5A | 481 | 486 | 5000 | win2 (Rsb 3.56, 10/11) | Rsb | win | win2 | 10 | 3.555 | 3.102 |
| css817 | 5A | 481 | 493 | 12000 | win2 (xpEHH 3.36, 25/25) | xpEHH | win | win2 | 25 | 3.364 | 2.893 |
| css818 | 5A | 484 | 493 | 9000 | ND (Rsb 3.17, 14/15) | Rsb | both | ND | 14 | 3.173 | 2.585 |
| css819 | 5A | 484 | 493 | 9000 | ND (Rsb 3.62, 15/15) | Rsb | spr | ND | 15 | 3.625 | 2.898 |
| css820 | 5A | 488 | 493 | 5000 | HRW2 (xpEHH 2.38, 11/11) | xpEHH | win | HRW2 | 11 | 2.379 | 2.241 |
| css821 | 5A | 499 | 508 | 9000 | pnw2 (xpEHH 3.27, 20/22) | xpEHH | win | pnw2 | 20 | 3.268 | 2.289 |
| css822 | 5A | 499 | 504 | 5000 | win2 (Rsb 4.25, 11/12) | Rsb | win | win2 | 11 | 4.249 | 3.353 |
| css823 | 5A | 499 | 510 | 11000 | win2 (xpEHH 3.32, 28/28) | xpEHH | win | win2 | 28 | 3.324 | 2.878 |
| css824 | 5A | 516 | 524 | 8000 | MN (Fst 0.49, 5/5) | Fst | both | MN | 5 | 0.487 | 0.423 |
| css825 | 5A | 557 | 563 | 6000 | gpl2 (xpEHH 2.33, 9/10) | xpEHH | win | gpl2 | 9 | 2.333 | 2.177 |
| css826 | 5A | 557 | 569 | 12000 | HRW2 (xpEHH 2.47, 19/21) | xpEHH | win | HRW2 | 19 | 2.474 | 2.201 |
| css827 | 5A | 568 | 573 | 5000 | NE (Rsb 3.45, 20/21) | Rsb | both | NE | 20 | 3.453 | 2.497 |
| css828 | 5A | 568 | 573 | 5000 | NE (xpEHH 2.69, 20/21) | xpEHH | both | NE | 20 | 2.686 | 2.415 |
| css829 | 5A | 568 | 576 | 8000 | nor (Rsb 4.41, 33/33) | Rsb | spr | nor | 33 | 4.406 | 3.149 |
| css830 | 5A | 568 | 601 | 33000 | nor (xpEHH 4.05, 117/119) | xpEHH | spr | nor | 117 | 4.050 | 2.704 |
| css831 | 5A | 578 | 585 | 7000 | CA (Fst 0.22, 15/16) | Fst | spr | CA | 15 | 0.223 | 0.210 |
| css832 | 5A | 578 | 586 | 8000 | HRS (Fst 0.54, 16/17) | Fst | spr | HRS | 16 | 0.539 | 0.505 |
| css833 | 5A | 578 | 586 | 8000 | MN (Fst 0.45, 16/17) | Fst | spr | MN | 16 | 0.447 | 0.426 |
| css834 | 5A | 578 | 586 | 8000 | ND (Fst 0.44, 16/17) | Fst | spr | ND | 16 | 0.443 | 0.355 |
| css835 | 5A | 578 | 586 | 8000 | nor (Fst 0.47, 16/17) | Fst | spr | nor | 16 | 0.469 | 0.418 |
| css836 | 5A | 578 | 585 | 7000 | pac (Fst 0.23, 15/16) | Fst | spr | pac | 15 | 0.227 | 0.213 |
| css837 | 5A | 578 | 586 | 8000 | SWS (Fst 0.45, 16/17) | Fst | spr | SWS | 16 | 0.450 | 0.430 |
| css838 | 5A | 581 | 592 | 11000 | eas (xpEHH 2.94, 38/41) | xpEHH | both | eas | 38 | 2.943 | 2.319 |
| css839 | 5A | 581 | 586 | 5000 | gpl2 (xpEHH 2.31, 12/13) | xpEHH | win | gpl2 | 12 | 2.309 | 2.230 |
| css840 | 5A | 583 | 588 | 5000 | nor (Rsb 3.69, 18/20) | Rsb | both | nor | 18 | 3.686 | 2.649 |
| css841 | 5A | 586 | 601 | 15000 | HRS (xpEHH 3.23, 59/64) | xpEHH | spr | HRS | 59 | 3.230 | 2.571 |
| css842 | 5A | 596 | 601 | 5000 | ND (xpEHH 2.59, 5/5) | xpEHH | both | ND | 5 | 2.587 | 2.178 |
| css843 | 5A | 596 | 602 | 6000 | nor (xpEHH 3.68, 12/12) | xpEHH | both | nor | 12 | 3.682 | 2.976 |
| css844 | 5A | 606 | 613 | 7000 | HRS (xpEHH 2.37, 10/10) | xpEHH | spr | HRS | 10 | 2.368 | 2.233 |
| css845 | 5A | 606 | 611 | 5000 | nor (Rsb 3.59, 6/6) | Rsb | both | nor | 6 | 3.590 | 2.790 |
| css846 | 5A | 606 | 611 | 5000 | nor (xpEHH 3.03, 6/6) | xpEHH | both | nor | 6 | 3.025 | 2.774 |
| css847 | 5A | 606 | 611 | 5000 | pnw (xpEHH 2.83, 6/6) | xpEHH | win | pnw | 6 | 2.828 | 2.338 |
| css848 | 5A | 606 | 616 | 10000 | SWW2 (xpEHH 3.00, 11/11) | xpEHH | win | SWW2 | 11 | 2.998 | 2.422 |
| css849 | 5A | 606 | 611 | 5000 | WA (xpEHH 2.58, 6/6) | xpEHH | win | WA | 6 | 2.583 | 2.203 |
| css850 | 5A | 617 | 624 | 7000 | nor (Fst 0.30, 9/10) | Fst | both | nor | 9 | 0.298 | 0.255 |
| css851 | 5A | 617 | 624 | 7000 | pnw (Fst 0.21, 9/10) | Fst | spr | pnw | 9 | 0.211 | 0.192 |
| css852 | 5A | 654 | 659 | 5000 | SWW2 (xpEHH 2.60, 10/10) | xpEHH | win | SWW2 | 10 | 2.605 | 2.508 |
| css853 | 5B | 45 | 50 | 5000 | ND (xpEHH 2.17, 6/6) | xpEHH | spr | ND | 6 | 2.175 | 2.078 |
| css854 | 5B | 45 | 56 | 11000 | nor (xpEHH 2.83, 14/14) | xpEHH | spr | nor | 14 | 2.834 | 2.487 |
| css855 | 5B | 47 | 53 | 6000 | nor (Rsb 3.10, 6/6) | Rsb | spr | nor | 6 | 3.104 | 2.898 |
| css856 | 5B | 52 | 65 | 13000 | ID (Fst 0.32, 34/35) | Fst | spr | ID | 34 | 0.317 | 0.273 |
| css857 | 5B | 70 | 75 | 5000 | OR (xpEHH 2.34, 10/10) | xpEHH | win | OR | 10 | 2.345 | 2.173 |
| css858 | 5B | 74 | 81 | 7000 | nor (xpEHH 2.73, 14/15) | xpEHH | spr | nor | 14 | 2.730 | 2.273 |
| css859 | 5B | 76 | 91 | 15000 | eas (xpEHH 2.92, 27/27) | xpEHH | win | eas | 27 | 2.920 | 2.396 |
| css860 | 5B | 76 | 91 | 15000 | eas (xpEHH 3.47, 27/27) | xpEHH | both | eas | 27 | 3.465 | 2.752 |
| css861 | 5B | 76 | 91 | 15000 | SRW (xpEHH 2.89, 27/27) | xpEHH | win | SRW | 27 | 2.889 | 2.511 |
| css862 | 5B | 80 | 91 | 11000 | eas (Rsb 3.51, 18/18) | Rsb | both | eas | 18 | 3.507 | 2.483 |
| css863 | 5B | 81 | 89 | 8000 | HRS2 (Fst 0.13, 14/16) | Fst | spr | HRS2 | 14 | 0.134 | 0.134 |
| css864 | 5B | 121 | 126 | 5000 | ID (Fst 0.30, 5/5) | Fst | both | ID | 5 | 0.297 | 0.290 |
| css865 | 5B | 121 | 126 | 5000 | ID (Fst 0.36, 5/5) | Fst | spr | ID | 5 | 0.363 | 0.363 |
| css866 | 5B | 121 | 126 | 5000 | pnw (Fst 0.12, 5/5) | Fst | both | pnw | 5 | 0.123 | 0.118 |
| css867 | 5B | 121 | 126 | 5000 | pnw (Fst 0.17, 5/5) | Fst | spr | pnw | 5 | 0.173 | 0.173 |
| css868 | 5B | 128 | 139 | 11000 | ID (Fst 0.31, 14/14) | Fst | both | ID | 14 | 0.308 | 0.308 |
| css869 | 5B | 128 | 139 | 11000 | ID (Fst 0.39, 14/14) | Fst | spr | ID | 14 | 0.394 | 0.394 |
| css870 | 5B | 128 | 139 | 11000 | pnw (Fst 0.12, 14/14) | Fst | both | pnw | 14 | 0.123 | 0.122 |
| css871 | 5B | 128 | 139 | 11000 | pnw (Fst 0.19, 14/14) | Fst | spr | pnw | 14 | 0.190 | 0.190 |
| css872 | 5B | 234 | 247 | 13000 | ID (Fst 0.32, 24/24) | Fst | both | ID | 24 | 0.319 | 0.303 |
| css873 | 5B | 234 | 247 | 13000 | ID (Fst 0.39, 23/24) | Fst | spr | ID | 23 | 0.394 | 0.394 |
| css874 | 5B | 234 | 247 | 13000 | pnw (Fst 0.13, 23/24) | Fst | both | pnw | 23 | 0.126 | 0.126 |
| css875 | 5B | 234 | 247 | 13000 | pnw (Fst 0.21, 23/24) | Fst | spr | pnw | 23 | 0.206 | 0.206 |
| css876 | 5B | 282 | 287 | 5000 | nor (xpEHH 2.24, 5/5) | xpEHH | spr | nor | 5 | 2.240 | 2.225 |
| css877 | 5B | 315 | 329 | 14000 | eas (xpEHH 2.81, 35/36) | xpEHH | both | eas | 35 | 2.808 | 2.163 |
| css878 | 5B | 315 | 330 | 15000 | eas (xpEHH 2.81, 38/38) | xpEHH | win | eas | 38 | 2.813 | 2.481 |
| css879 | 5B | 315 | 330 | 15000 | IN (xpEHH 2.73, 37/37) | xpEHH | win | IN | 37 | 2.730 | 2.247 |
| css880 | 5B | 315 | 330 | 15000 | ND (xpEHH 2.76, 36/36) | xpEHH | both | ND | 36 | 2.755 | 2.157 |
| css881 | 5B | 315 | 320 | 5000 | nor (xpEHH 2.70, 8/8) | xpEHH | both | nor | 8 | 2.698 | 2.337 |
| css882 | 5B | 315 | 335 | 20000 | nor (xpEHH 3.11, 45/45) | xpEHH | spr | nor | 45 | 3.108 | 2.454 |
| css883 | 5B | 315 | 335 | 20000 | SRW (xpEHH 3.36, 48/48) | xpEHH | win | SRW | 48 | 3.356 | 2.619 |
| css884 | 5B | 330 | 335 | 5000 | nor (Rsb 2.34, 9/10) | Rsb | spr | nor | 9 | 2.341 | 2.240 |
| css885 | 5B | 330 | 335 | 5000 | pac2 (Fst 0.48, 9/10) | Fst | both | pac2 | 9 | 0.479 | 0.441 |
| css886 | 5B | 330 | 335 | 5000 | pac2 (Fst 0.55, 9/10) | Fst | spr | pac2 | 9 | 0.553 | 0.513 |
| css887 | 5B | 396 | 423 | 27000 | nor (xpEHH 2.67, 42/42) | xpEHH | spr | nor | 42 | 2.669 | 2.333 |
| css888 | 5B | 409 | 417 | 8000 | HRS (xpEHH 2.16, 11/12) | xpEHH | spr | HRS | 11 | 2.159 | 2.109 |
| css889 | 5B | 410 | 423 | 13000 | nor (Rsb 2.80, 18/19) | Rsb | spr | nor | 18 | 2.798 | 2.385 |
| css890 | 5B | 429 | 446 | 17000 | nor (xpEHH 3.11, 26/26) | xpEHH | spr | nor | 26 | 3.114 | 2.609 |
| css891 | 5B | 434 | 445 | 11000 | eas (xpEHH 2.92, 16/16) | xpEHH | both | eas | 16 | 2.921 | 2.527 |
| css892 | 5B | 434 | 446 | 12000 | ND (xpEHH 2.71, 13/13) | xpEHH | spr | ND | 13 | 2.712 | 2.444 |
| css893 | 5B | 437 | 445 | 8000 | eas (xpEHH 2.47, 10/10) | xpEHH | win | eas | 10 | 2.468 | 2.349 |
| css894 | 5B | 437 | 443 | 6000 | nor (Rsb 3.38, 9/10) | Rsb | spr | nor | 9 | 3.377 | 2.574 |
| css895 | 5B | 437 | 443 | 6000 | SRW (xpEHH 2.31, 9/10) | xpEHH | win | SRW | 9 | 2.306 | 2.181 |
| css896 | 5B | 438 | 443 | 5000 | IN (xpEHH 2.50, 9/10) | xpEHH | win | IN | 9 | 2.495 | 2.249 |
| css897 | 5B | 456 | 472 | 16000 | NY (xpEHH 3.23, 49/52) | xpEHH | win | NY | 49 | 3.227 | 2.279 |
| css898 | 5B | 459 | 464 | 5000 | HRW (Fst 0.52, 19/21) | Fst | win | HRW | 19 | 0.520 | 0.474 |
| css899 | 5B | 459 | 464 | 5000 | KS (Fst 0.34, 19/21) | Fst | win | KS | 19 | 0.340 | 0.303 |
| css900 | 5B | 469 | 476 | 7000 | NY (Rsb 2.71, 13/14) | Rsb | win | NY | 13 | 2.706 | 2.245 |
| css901 | 5B | 474 | 486 | 12000 | NY (xpEHH 2.84, 45/47) | xpEHH | win | NY | 45 | 2.840 | 2.385 |
| css902 | 5B | 475 | 486 | 11000 | eas (Rsb 4.71, 49/52) | Rsb | both | eas | 49 | 4.705 | 3.247 |
| css903 | 5B | 477 | 486 | 9000 | eas (xpEHH 4.51, 41/42) | xpEHH | both | eas | 41 | 4.512 | 3.441 |
| css904 | 5B | 477 | 493 | 16000 | HRW2 (xpEHH 3.16, 61/63) | xpEHH | win | HRW2 | 61 | 3.163 | 2.462 |
| css905 | 5B | 477 | 484 | 7000 | SC (xpEHH 3.00, 33/38) | xpEHH | both | SC | 33 | 2.997 | 2.380 |
| css906 | 5B | 486 | 493 | 7000 | gpl2 (xpEHH 2.67, 21/21) | xpEHH | win | gpl2 | 21 | 2.668 | 2.270 |
| css907 | 5B | 496 | 518 | 22000 | CA (Fst 0.52, 111/116) | Fst | both | CA | 111 | 0.521 | 0.467 |
| css908 | 5B | 496 | 518 | 22000 | OR (Fst 0.41, 105/116) | Fst | win | OR | 105 | 0.414 | 0.390 |
| css909 | 5B | 500 | 518 | 18000 | ID (Fst 0.34, 106/112) | Fst | both | ID | 106 | 0.339 | 0.318 |
| css910 | 5B | 500 | 518 | 18000 | pac (Fst 0.50, 107/112) | Fst | both | pac | 107 | 0.500 | 0.395 |
| css911 | 5B | 511 | 518 | 7000 | ID (Fst 0.23, 24/26) | Fst | win | ID | 24 | 0.232 | 0.220 |
| css912 | 5B | 531 | 542 | 11000 | CO (Rsb 3.57, 44/45) | Rsb | both | CO | 44 | 3.565 | 2.628 |
| css913 | 5B | 531 | 546 | 15000 | CO (xpEHH 2.92, 47/50) | xpEHH | win | CO | 47 | 2.918 | 2.441 |
| css914 | 5B | 532 | 546 | 14000 | CO (xpEHH 3.38, 48/50) | xpEHH | both | CO | 48 | 3.382 | 2.713 |
| css915 | 5B | 534 | 566 | 32000 | CO (Rsb 3.73, 109/111) | Rsb | win | CO | 109 | 3.735 | 2.528 |
| css916 | 5B | 537 | 547 | 10000 | HRW2 (xpEHH 2.81, 26/26) | xpEHH | win | HRW2 | 26 | 2.813 | 2.389 |
| css917 | 5B | 537 | 542 | 5000 | TX (xpEHH 2.48, 15/16) | xpEHH | both | TX | 15 | 2.479 | 2.285 |
| css918 | 5B | 538 | 551 | 13000 | gpl2 (xpEHH 2.68, 41/41) | xpEHH | win | gpl2 | 41 | 2.676 | 2.203 |
| css919 | 5B | 540 | 546 | 6000 | win2 (xpEHH 2.96, 9/10) | xpEHH | win | win2 | 9 | 2.957 | 2.511 |
| css920 | 5B | 544 | 549 | 5000 | MT (Fst 0.38, 11/12) | Fst | both | MT | 11 | 0.377 | 0.313 |
| css921 | 5B | 544 | 549 | 5000 | nor (Fst 0.40, 11/12) | Fst | win | nor | 11 | 0.405 | 0.395 |
| css922 | 5B | 545 | 566 | 21000 | CO (Rsb 4.11, 72/73) | Rsb | both | CO | 72 | 4.110 | 2.846 |
| css923 | 5B | 549 | 566 | 17000 | CO (xpEHH 3.75, 58/58) | xpEHH | win | CO | 58 | 3.747 | 2.910 |
| css924 | 5B | 549 | 565 | 16000 | CO (xpEHH 4.15, 60/61) | xpEHH | both | CO | 60 | 4.152 | 3.068 |
| css925 | 5B | 549 | 554 | 5000 | gpl (Rsb 3.18, 18/18) | Rsb | win | gpl | 18 | 3.179 | 2.770 |
| css926 | 5B | 550 | 563 | 13000 | gpl (xpEHH 3.73, 52/53) | xpEHH | win | gpl | 52 | 3.731 | 2.599 |
| css927 | 5B | 550 | 558 | 8000 | NE (xpEHH 2.98, 27/29) | xpEHH | win | NE | 27 | 2.981 | 2.417 |
| css928 | 5B | 552 | 601 | 49000 | SWS (xpEHH 4.24, 164/165) | xpEHH | spr | SWS | 164 | 4.244 | 3.170 |
| css929 | 5B | 557 | 566 | 9000 | CA (xpEHH 2.68, 22/25) | xpEHH | spr | CA | 22 | 2.678 | 2.464 |
| css930 | 5B | 557 | 567 | 10000 | ID (xpEHH 3.24, 28/30) | xpEHH | spr | ID | 28 | 3.239 | 2.447 |
| css931 | 5B | 558 | 563 | 5000 | CA (Rsb 3.43, 19/20) | Rsb | spr | CA | 19 | 3.431 | 2.469 |
| css932 | 5B | 558 | 597 | 39000 | SWS (Rsb 4.59, 124/127) | Rsb | spr | SWS | 124 | 4.591 | 3.163 |
| css933 | 5B | 561 | 566 | 5000 | pnw (xpEHH 2.67, 10/11) | xpEHH | spr | pnw | 10 | 2.667 | 2.396 |
| css934 | 5B | 561 | 566 | 5000 | TX (Fst 0.28, 10/11) | Fst | both | TX | 10 | 0.277 | 0.224 |
| css935 | 5B | 566 | 576 | 10000 | HRW2 (xpEHH 2.52, 41/43) | xpEHH | win | HRW2 | 41 | 2.523 | 2.214 |
| css936 | 5B | 566 | 589 | 23000 | ID (Rsb 4.35, 65/68) | Rsb | spr | ID | 65 | 4.349 | 2.667 |
| css937 | 5B | 567 | 598 | 31000 | CA (xpEHH 3.54, 95/99) | xpEHH | spr | CA | 95 | 3.537 | 2.674 |
| css938 | 5B | 572 | 579 | 7000 | pac (xpEHH 3.03, 23/25) | xpEHH | spr | pac | 23 | 3.032 | 2.525 |
| css939 | 5B | 575 | 580 | 5000 | CA (Rsb 3.47, 9/9) | Rsb | spr | CA | 9 | 3.474 | 2.928 |
| css940 | 5B | 576 | 592 | 16000 | ID (xpEHH 3.73, 34/36) | xpEHH | spr | ID | 34 | 3.729 | 2.905 |
| css941 | 5B | 577 | 583 | 6000 | SC (Fst 0.45, 9/10) | Fst | both | SC | 9 | 0.454 | 0.450 |
| css942 | 5B | 579 | 588 | 9000 | CO (xpEHH 2.54, 9/9) | xpEHH | win | CO | 9 | 2.544 | 2.317 |
| css943 | 5B | 579 | 590 | 11000 | pnw (xpEHH 3.01, 26/27) | xpEHH | spr | pnw | 26 | 3.013 | 2.463 |
| css944 | 5B | 581 | 588 | 7000 | CO (xpEHH 2.86, 9/9) | xpEHH | both | CO | 9 | 2.863 | 2.486 |
| css945 | 5B | 584 | 589 | 5000 | CO (Rsb 3.11, 11/12) | Rsb | both | CO | 11 | 3.111 | 2.370 |
| css946 | 5B | 585 | 595 | 10000 | ID (Rsb 3.98, 29/32) | Rsb | both | ID | 29 | 3.978 | 2.739 |
| css947 | 5B | 585 | 598 | 13000 | pac (xpEHH 3.61, 41/42) | xpEHH | spr | pac | 41 | 3.612 | 2.752 |
| css948 | 5B | 586 | 597 | 11000 | CA (Rsb 4.40, 35/38) | Rsb | both | CA | 35 | 4.403 | 3.013 |
| css949 | 5B | 588 | 597 | 9000 | CA (xpEHH 4.45, 32/35) | xpEHH | both | CA | 32 | 4.451 | 2.930 |
| css950 | 5B | 588 | 594 | 6000 | TX (xpEHH 3.98, 21/22) | xpEHH | both | TX | 21 | 3.984 | 2.820 |
| css951 | 5B | 589 | 595 | 6000 | pac (xpEHH 3.98, 24/24) | xpEHH | both | pac | 24 | 3.975 | 2.902 |
| css952 | 5B | 589 | 599 | 10000 | TX (Rsb 3.98, 37/40) | Rsb | both | TX | 37 | 3.978 | 2.815 |
| css953 | 5B | 590 | 595 | 5000 | CA (Rsb 4.03, 18/19) | Rsb | spr | CA | 18 | 4.031 | 2.711 |
| css954 | 5B | 590 | 595 | 5000 | pac (Rsb 3.76, 20/21) | Rsb | spr | pac | 20 | 3.764 | 2.596 |
| css955 | 5B | 667 | 686 | 19000 | SWS (xpEHH 3.58, 84/87) | xpEHH | spr | SWS | 84 | 3.577 | 2.892 |
| css956 | 5B | 671 | 689 | 18000 | ID (xpEHH 3.53, 74/77) | xpEHH | spr | ID | 74 | 3.533 | 2.908 |
| css957 | 5B | 672 | 686 | 14000 | ID (Rsb 3.68, 30/30) | Rsb | spr | ID | 30 | 3.683 | 2.552 |
| css958 | 5B | 675 | 683 | 8000 | pac (xpEHH 3.32, 34/35) | xpEHH | spr | pac | 34 | 3.321 | 2.870 |
| css959 | 5B | 676 | 681 | 5000 | IN (Rsb 2.88, 25/27) | Rsb | both | IN | 25 | 2.880 | 2.287 |
| css960 | 5B | 676 | 682 | 6000 | IN (xpEHH 2.56, 29/32) | xpEHH | both | IN | 29 | 2.562 | 2.432 |
| css961 | 5B | 676 | 686 | 10000 | SWS (Rsb 4.39, 47/50) | Rsb | spr | SWS | 47 | 4.388 | 3.093 |
| css962 | 5B | 677 | 682 | 5000 | CA (xpEHH 3.10, 31/34) | xpEHH | spr | CA | 31 | 3.098 | 2.833 |
| css963 | 5B | 694 | 700 | 6000 | SWS (Rsb 4.21, 19/19) | Rsb | spr | SWS | 19 | 4.207 | 2.871 |
| css964 | 5D | 184 | 193 | 9000 | CA (Fst 0.23, 6/6) | Fst | spr | CA | 6 | 0.230 | 0.230 |
| css965 | 5D | 184 | 193 | 9000 | ND (Fst 0.36, 6/6) | Fst | spr | ND | 6 | 0.359 | 0.359 |
| css966 | 5D | 184 | 193 | 9000 | nor (Fst 0.27, 6/6) | Fst | spr | nor | 6 | 0.266 | 0.266 |
| css967 | 5D | 184 | 193 | 9000 | pac (Fst 0.25, 6/6) | Fst | spr | pac | 6 | 0.246 | 0.246 |
| css968 | 5D | 184 | 193 | 9000 | SWW (Fst 0.23, 6/6) | Fst | win | SWW | 6 | 0.230 | 0.230 |
| css969 | 6A | 29 | 35 | 6000 | pac2 (Fst 0.52, 24/27) | Fst | both | pac2 | 24 | 0.520 | 0.520 |
| css970 | 6A | 29 | 35 | 6000 | pac2 (Fst 0.59, 24/27) | Fst | spr | pac2 | 24 | 0.595 | 0.595 |
| css971 | 6A | 30 | 35 | 5000 | ND (Fst 0.30, 23/25) | Fst | spr | ND | 23 | 0.298 | 0.298 |
| css972 | 6A | 30 | 35 | 5000 | ND (Fst 0.35, 24/25) | Fst | both | ND | 24 | 0.347 | 0.336 |
| css973 | 6A | 37 | 46 | 9000 | WA (Rsb 2.60, 22/24) | Rsb | spr | WA | 22 | 2.601 | 2.291 |
| css974 | 6A | 40 | 46 | 6000 | SC (Fst 0.68, 11/12) | Fst | both | SC | 11 | 0.677 | 0.643 |
| css975 | 6A | 48 | 54 | 6000 | ID (xpEHH 2.56, 5/5) | xpEHH | win | ID | 5 | 2.559 | 2.405 |
| css976 | 6A | 48 | 54 | 6000 | SWW (Rsb 2.49, 5/5) | Rsb | win | SWW | 5 | 2.493 | 2.173 |
| css977 | 6A | 60 | 66 | 6000 | SC (Fst 0.57, 21/23) | Fst | both | SC | 21 | 0.570 | 0.497 |
| css978 | 6A | 74 | 88 | 14000 | pnw2 (Rsb 2.32, 23/24) | Rsb | spr | pnw2 | 23 | 2.318 | 2.141 |
| css979 | 6A | 81 | 88 | 7000 | KS (Fst 0.23, 10/11) | Fst | win | KS | 10 | 0.227 | 0.227 |
| css980 | 6A | 90 | 95 | 5000 | CA (Fst 0.28, 5/5) | Fst | spr | CA | 5 | 0.284 | 0.284 |
| css981 | 6A | 90 | 95 | 5000 | IN (Fst 0.44, 5/5) | Fst | both | IN | 5 | 0.436 | 0.433 |
| css982 | 6A | 90 | 95 | 5000 | IN (Fst 0.49, 5/5) | Fst | win | IN | 5 | 0.494 | 0.489 |
| css983 | 6A | 90 | 95 | 5000 | pac (Fst 0.24, 5/5) | Fst | spr | pac | 5 | 0.243 | 0.243 |
| css984 | 6A | 103 | 112 | 9000 | all2 (Fst 0.12, 9/9) | Fst | both | all2 | 9 | 0.121 | 0.100 |
| css985 | 6A | 103 | 112 | 9000 | pnw2 (Fst 0.23, 9/9) | Fst | both | pnw2 | 9 | 0.232 | 0.187 |
| css986 | 6A | 103 | 112 | 9000 | win2 (Fst 0.13, 9/9) | Fst | win | win2 | 9 | 0.128 | 0.120 |
| css987 | 6A | 106 | 112 | 6000 | eas2 (Fst 0.20, 6/6) | Fst | both | eas2 | 6 | 0.198 | 0.198 |
| css988 | 6A | 106 | 112 | 6000 | eas2 (Fst 0.20, 6/6) | Fst | win | eas2 | 6 | 0.203 | 0.203 |
| css989 | 6A | 106 | 112 | 6000 | pnw2 (Fst 0.21, 6/6) | Fst | win | pnw2 | 6 | 0.209 | 0.209 |
| css990 | 6A | 106 | 112 | 6000 | pnw2 (Rsb 2.07, 6/6) | Rsb | win | pnw2 | 6 | 2.067 | 2.067 |
| css991 | 6A | 106 | 112 | 6000 | SRW2 (Fst 0.24, 6/6) | Fst | win | SRW2 | 6 | 0.242 | 0.236 |
| css992 | 6A | 106 | 112 | 6000 | SWW2 (Fst 0.19, 6/6) | Fst | win | SWW2 | 6 | 0.187 | 0.187 |
| css993 | 6A | 106 | 112 | 6000 | SWW2 (Rsb 2.17, 6/6) | Rsb | win | SWW2 | 6 | 2.172 | 2.172 |
| css994 | 6A | 232 | 238 | 6000 | ID (Fst 0.30, 5/5) | Fst | both | ID | 5 | 0.296 | 0.296 |
| css995 | 6A | 232 | 238 | 6000 | ID (Fst 0.30, 5/5) | Fst | spr | ID | 5 | 0.297 | 0.297 |
| css996 | 6A | 232 | 238 | 6000 | pnw2 (Fst 0.22, 5/5) | Fst | both | pnw2 | 5 | 0.224 | 0.224 |
| css997 | 6A | 232 | 238 | 6000 | pnw2 (Fst 0.28, 5/5) | Fst | spr | pnw2 | 5 | 0.283 | 0.283 |
| css998 | 6A | 409 | 418 | 9000 | all2 (Fst 0.12, 8/8) | Fst | both | all2 | 8 | 0.125 | 0.124 |
| css999 | 6A | 409 | 418 | 9000 | eas2 (Fst 0.19, 8/8) | Fst | both | eas2 | 8 | 0.188 | 0.188 |
| css1000 | 6A | 409 | 418 | 9000 | eas2 (Fst 0.19, 8/8) | Fst | win | eas2 | 8 | 0.194 | 0.194 |
| css1001 | 6A | 409 | 418 | 9000 | HRW2 (Fst 0.10, 8/8) | Fst | win | HRW2 | 8 | 0.100 | 0.100 |
| css1002 | 6A | 409 | 418 | 9000 | ID (Fst 0.27, 8/8) | Fst | spr | ID | 8 | 0.268 | 0.268 |
| css1003 | 6A | 409 | 418 | 9000 | pnw2 (Fst 0.21, 8/8) | Fst | win | pnw2 | 8 | 0.209 | 0.198 |
| css1004 | 6A | 409 | 418 | 9000 | pnw2 (Fst 0.30, 8/8) | Fst | both | pnw2 | 8 | 0.302 | 0.296 |
| css1005 | 6A | 409 | 418 | 9000 | pnw2 (Fst 0.33, 8/8) | Fst | spr | pnw2 | 8 | 0.329 | 0.329 |
| css1006 | 6A | 409 | 418 | 9000 | SRW2 (Fst 0.21, 8/8) | Fst | win | SRW2 | 8 | 0.209 | 0.209 |
| css1007 | 6A | 409 | 418 | 9000 | SWS2 (Fst 0.38, 8/8) | Fst | spr | SWS2 | 8 | 0.382 | 0.382 |
| css1008 | 6A | 409 | 418 | 9000 | win2 (Fst 0.15, 8/8) | Fst | win | win2 | 8 | 0.148 | 0.147 |
| css1009 | 6A | 429 | 438 | 9000 | all2 (Fst 0.12, 7/7) | Fst | both | all2 | 7 | 0.125 | 0.125 |
| css1010 | 6A | 429 | 438 | 9000 | eas2 (Fst 0.19, 7/7) | Fst | both | eas2 | 7 | 0.188 | 0.188 |
| css1011 | 6A | 429 | 438 | 9000 | eas2 (Fst 0.19, 7/7) | Fst | win | eas2 | 7 | 0.194 | 0.194 |
| css1012 | 6A | 429 | 438 | 9000 | HRW2 (Fst 0.10, 7/7) | Fst | win | HRW2 | 7 | 0.100 | 0.100 |
| css1013 | 6A | 429 | 438 | 9000 | ID (Fst 0.27, 7/7) | Fst | spr | ID | 7 | 0.268 | 0.268 |
| css1014 | 6A | 429 | 444 | 15000 | pnw2 (Fst 0.21, 14/14) | Fst | win | pnw2 | 14 | 0.209 | 0.209 |
| css1015 | 6A | 429 | 438 | 9000 | pnw2 (Fst 0.30, 7/7) | Fst | both | pnw2 | 7 | 0.302 | 0.302 |
| css1016 | 6A | 429 | 438 | 9000 | pnw2 (Fst 0.33, 7/7) | Fst | spr | pnw2 | 7 | 0.329 | 0.329 |
| css1017 | 6A | 429 | 438 | 9000 | SRW2 (Fst 0.21, 7/7) | Fst | win | SRW2 | 7 | 0.209 | 0.209 |
| css1018 | 6A | 429 | 446 | 17000 | SWS2 (Fst 0.38, 15/15) | Fst | spr | SWS2 | 15 | 0.382 | 0.382 |
| css1019 | 6A | 429 | 438 | 9000 | win2 (Fst 0.15, 7/7) | Fst | win | win2 | 7 | 0.148 | 0.148 |
| css1020 | 6A | 520 | 538 | 18000 | SWS2 (Fst 0.58, 31/32) | Fst | spr | SWS2 | 31 | 0.583 | 0.513 |
| css1021 | 6A | 524 | 535 | 11000 | ND (Fst 0.38, 18/18) | Fst | both | ND | 18 | 0.382 | 0.359 |
| css1022 | 6A | 524 | 538 | 14000 | pnw2 (Fst 0.24, 25/25) | Fst | both | pnw2 | 25 | 0.243 | 0.186 |
| css1023 | 6A | 529 | 540 | 11000 | pac2 (Fst 0.54, 21/22) | Fst | spr | pac2 | 21 | 0.541 | 0.488 |
| css1024 | 6A | 529 | 540 | 11000 | pac2 (Fst 0.56, 21/22) | Fst | both | pac2 | 21 | 0.558 | 0.507 |
| css1025 | 6A | 529 | 538 | 9000 | pnw2 (Fst 0.54, 18/18) | Fst | spr | pnw2 | 18 | 0.536 | 0.418 |
| css1026 | 6A | 542 | 548 | 6000 | CA (Fst 0.39, 15/17) | Fst | spr | CA | 15 | 0.389 | 0.310 |
| css1027 | 6A | 542 | 548 | 6000 | CA (Fst 0.50, 15/17) | Fst | both | CA | 15 | 0.503 | 0.433 |
| css1028 | 6A | 542 | 548 | 6000 | pac (Fst 0.39, 15/17) | Fst | spr | pac | 15 | 0.395 | 0.317 |
| css1029 | 6A | 542 | 548 | 6000 | pac (Fst 0.52, 15/17) | Fst | both | pac | 15 | 0.515 | 0.444 |
| css1030 | 6A | 562 | 568 | 6000 | pnw2 (Fst 0.25, 8/8) | Fst | both | pnw2 | 8 | 0.249 | 0.222 |
| css1031 | 6A | 562 | 568 | 6000 | pnw2 (Fst 0.36, 8/8) | Fst | spr | pnw2 | 8 | 0.360 | 0.353 |
| css1032 | 6A | 562 | 568 | 6000 | SC (Fst 0.70, 8/8) | Fst | win | SC | 8 | 0.700 | 0.660 |
| css1033 | 6A | 562 | 568 | 6000 | spr2 (Fst 0.25, 8/8) | Fst | spr | spr2 | 8 | 0.250 | 0.232 |
| css1034 | 6A | 562 | 568 | 6000 | SWS2 (Fst 0.44, 8/8) | Fst | spr | SWS2 | 8 | 0.441 | 0.441 |
| css1035 | 6A | 567 | 573 | 6000 | CA (Fst 0.16, 21/23) | Fst | spr | CA | 21 | 0.158 | 0.158 |
| css1036 | 6A | 567 | 573 | 6000 | eas2 (Fst 0.17, 21/23) | Fst | win | eas2 | 21 | 0.167 | 0.161 |
| css1037 | 6A | 567 | 573 | 6000 | KS (Fst 0.16, 21/23) | Fst | both | KS | 21 | 0.158 | 0.157 |
| css1038 | 6A | 567 | 573 | 6000 | pac (Fst 0.17, 21/23) | Fst | spr | pac | 21 | 0.166 | 0.166 |
| css1039 | 6A | 567 | 573 | 6000 | pnw2 (Rsb 2.03, 21/23) | Rsb | spr | pnw2 | 21 | 2.031 | 2.031 |
| css1040 | 6A | 567 | 573 | 6000 | SRW2 (Fst 0.20, 21/23) | Fst | win | SRW2 | 21 | 0.199 | 0.199 |
| css1041 | 6A | 567 | 573 | 6000 | SWS2 (Rsb 2.05, 21/23) | Rsb | spr | SWS2 | 21 | 2.047 | 2.047 |
| css1042 | 6A | 574 | 581 | 7000 | pac (Fst 0.54, 7/7) | Fst | both | pac | 7 | 0.540 | 0.380 |
| css1043 | 6A | 577 | 583 | 6000 | CA (Fst 0.33, 10/10) | Fst | spr | CA | 10 | 0.326 | 0.246 |
| css1044 | 6A | 577 | 582 | 5000 | nor (Fst 0.22, 8/8) | Fst | spr | nor | 8 | 0.219 | 0.215 |
| css1045 | 6A | 577 | 582 | 5000 | pac (Fst 0.32, 8/8) | Fst | spr | pac | 8 | 0.318 | 0.261 |
| css1046 | 6A | 578 | 583 | 5000 | gpl (Fst 0.32, 5/5) | Fst | spr | gpl | 5 | 0.317 | 0.309 |
| css1047 | 6A | 578 | 583 | 5000 | gpl (Rsb 2.58, 5/5) | Rsb | spr | gpl | 5 | 2.576 | 2.555 |
| css1048 | 6A | 578 | 583 | 5000 | MN (Fst 0.39, 5/5) | Fst | spr | MN | 5 | 0.388 | 0.388 |
| css1049 | 6A | 578 | 583 | 5000 | MN (Fst 0.67, 5/5) | Fst | both | MN | 5 | 0.667 | 0.657 |
| css1050 | 6A | 578 | 583 | 5000 | MN (Rsb 2.53, 5/5) | Rsb | spr | MN | 5 | 2.525 | 2.525 |
| css1051 | 6A | 578 | 583 | 5000 | ND (Fst 0.52, 5/5) | Fst | both | ND | 5 | 0.516 | 0.504 |
| css1052 | 6A | 578 | 583 | 5000 | ND (Rsb 2.83, 5/5) | Rsb | spr | ND | 5 | 2.834 | 2.834 |
| css1053 | 6A | 578 | 583 | 5000 | nor (Fst 0.33, 5/5) | Fst | both | nor | 5 | 0.332 | 0.317 |
| css1054 | 6A | 578 | 583 | 5000 | nor (Rsb 2.24, 5/5) | Rsb | spr | nor | 5 | 2.238 | 2.167 |
| css1055 | 6A | 585 | 592 | 7000 | ND (Rsb 3.64, 12/13) | Rsb | spr | ND | 12 | 3.645 | 3.050 |
| css1056 | 6A | 586 | 591 | 5000 | gpl (Rsb 3.29, 10/11) | Rsb | spr | gpl | 10 | 3.285 | 2.781 |
| css1057 | 6A | 586 | 599 | 13000 | MN (Rsb 3.52, 22/24) | Rsb | spr | MN | 22 | 3.516 | 2.657 |
| css1058 | 6A | 586 | 592 | 6000 | ND (Rsb 3.43, 9/9) | Rsb | both | ND | 9 | 3.435 | 2.605 |
| css1059 | 6A | 609 | 617 | 8000 | pnw2 (Rsb 2.94, 6/6) | Rsb | win | pnw2 | 6 | 2.938 | 2.606 |
| css1060 | 6A | 612 | 619 | 7000 | pnw2 (xpEHH 3.05, 26/26) | xpEHH | win | pnw2 | 26 | 3.045 | 2.507 |
| css1061 | 6B | 18 | 23 | 5000 | gpl2 (Fst 0.29, 9/10) | Fst | win | gpl2 | 9 | 0.289 | 0.203 |
| css1062 | 6B | 24 | 29 | 5000 | pac (xpEHH 2.46, 9/9) | xpEHH | spr | pac | 9 | 2.464 | 2.309 |
| css1063 | 6B | 30 | 36 | 6000 | eas2 (xpEHH 2.47, 8/8) | xpEHH | both | eas2 | 8 | 2.474 | 2.378 |
| css1064 | 6B | 30 | 36 | 6000 | eas2 (xpEHH 2.52, 8/8) | xpEHH | win | eas2 | 8 | 2.521 | 2.422 |
| css1065 | 6B | 30 | 39 | 9000 | IN (xpEHH 2.70, 10/10) | xpEHH | both | IN | 10 | 2.697 | 2.498 |
| css1066 | 6B | 30 | 39 | 9000 | IN (xpEHH 3.12, 10/10) | xpEHH | win | IN | 10 | 3.117 | 2.970 |
| css1067 | 6B | 30 | 39 | 9000 | pac (xpEHH 2.69, 10/10) | xpEHH | both | pac | 10 | 2.691 | 2.444 |
| css1068 | 6B | 30 | 39 | 9000 | pac (xpEHH 3.18, 10/10) | xpEHH | spr | pac | 10 | 3.184 | 2.904 |
| css1069 | 6B | 30 | 36 | 6000 | SRW2 (xpEHH 2.49, 8/8) | xpEHH | win | SRW2 | 8 | 2.485 | 2.342 |
| css1070 | 6B | 40 | 51 | 11000 | IN (Rsb 3.40, 21/21) | Rsb | both | IN | 21 | 3.399 | 2.562 |
| css1071 | 6B | 40 | 59 | 19000 | IN (Rsb 3.88, 37/38) | Rsb | win | IN | 37 | 3.881 | 2.792 |
| css1072 | 6B | 40 | 47 | 7000 | IN (xpEHH 2.60, 17/17) | xpEHH | both | IN | 17 | 2.596 | 2.224 |
| css1073 | 6B | 40 | 59 | 19000 | IN (xpEHH 3.14, 38/38) | xpEHH | win | IN | 38 | 3.142 | 2.589 |
| css1074 | 6B | 40 | 47 | 7000 | pac (Rsb 2.95, 16/17) | Rsb | spr | pac | 16 | 2.954 | 2.612 |
| css1075 | 6B | 40 | 57 | 17000 | pac (xpEHH 2.76, 38/39) | xpEHH | both | pac | 38 | 2.762 | 2.350 |
| css1076 | 6B | 40 | 59 | 19000 | pac (xpEHH 2.88, 39/39) | xpEHH | spr | pac | 39 | 2.876 | 2.599 |
| css1077 | 6B | 40 | 45 | 5000 | pac2 (xpEHH 2.64, 6/6) | xpEHH | both | pac2 | 6 | 2.643 | 2.416 |
| css1078 | 6B | 40 | 47 | 7000 | pac2 (xpEHH 2.66, 18/18) | xpEHH | spr | pac2 | 18 | 2.658 | 2.153 |
| css1079 | 6B | 42 | 47 | 5000 | eas2 (xpEHH 2.83, 16/17) | xpEHH | win | eas2 | 16 | 2.832 | 2.454 |
| css1080 | 6B | 42 | 47 | 5000 | eas2 (xpEHH 2.97, 17/17) | xpEHH | both | eas2 | 17 | 2.966 | 2.520 |
| css1081 | 6B | 42 | 55 | 13000 | pac2 (Rsb 2.66, 27/27) | Rsb | spr | pac2 | 27 | 2.656 | 2.248 |
| css1082 | 6B | 45 | 57 | 12000 | CA (xpEHH 2.31, 31/33) | xpEHH | spr | CA | 31 | 2.314 | 2.127 |
| css1083 | 6B | 48 | 55 | 7000 | CA (Fst 0.24, 12/13) | Fst | spr | CA | 12 | 0.236 | 0.198 |
| css1084 | 6B | 48 | 53 | 5000 | IN (xpEHH 2.25, 9/10) | xpEHH | both | IN | 9 | 2.252 | 2.156 |
| css1085 | 6B | 48 | 55 | 7000 | pac (Fst 0.24, 12/13) | Fst | spr | pac | 12 | 0.243 | 0.205 |
| css1086 | 6B | 122 | 143 | 21000 | IN (Rsb 3.77, 83/83) | Rsb | both | IN | 83 | 3.770 | 2.609 |
| css1087 | 6B | 122 | 145 | 23000 | IN (Rsb 4.28, 85/85) | Rsb | win | IN | 85 | 4.285 | 3.028 |
| css1088 | 6B | 122 | 168 | 46000 | IN (xpEHH 3.70, 178/178) | xpEHH | win | IN | 178 | 3.701 | 2.754 |
| css1089 | 6B | 122 | 127 | 5000 | pac2 (Rsb 2.51, 7/7) | Rsb | spr | pac2 | 7 | 2.512 | 2.264 |
| css1090 | 6B | 122 | 168 | 46000 | pnw2 (Rsb 4.56, 159/159) | Rsb | spr | pnw2 | 159 | 4.559 | 3.049 |
| css1091 | 6B | 122 | 175 | 53000 | pnw2 (xpEHH 4.13, 166/166) | xpEHH | spr | pnw2 | 166 | 4.130 | 3.602 |
| css1092 | 6B | 122 | 191 | 69000 | spr2 (Rsb 5.06, 208/208) | Rsb | spr | spr2 | 208 | 5.063 | 2.976 |
| css1093 | 6B | 123 | 143 | 20000 | IN (xpEHH 2.91, 89/91) | xpEHH | both | IN | 89 | 2.907 | 2.553 |
| css1094 | 6B | 123 | 168 | 45000 | MN (Rsb 4.42, 178/179) | Rsb | both | MN | 178 | 4.416 | 2.902 |
| css1095 | 6B | 123 | 128 | 5000 | SWS2 (Rsb 3.52, 9/10) | Rsb | spr | SWS2 | 9 | 3.517 | 2.645 |
| css1096 | 6B | 124 | 131 | 7000 | CA (Rsb 4.00, 21/22) | Rsb | both | CA | 21 | 4.004 | 2.986 |
| css1097 | 6B | 124 | 141 | 17000 | CA (xpEHH 3.31, 84/86) | xpEHH | both | CA | 84 | 3.314 | 2.581 |
| css1098 | 6B | 124 | 143 | 19000 | MN (xpEHH 3.37, 88/90) | xpEHH | both | MN | 88 | 3.373 | 2.710 |
| css1099 | 6B | 124 | 143 | 19000 | pac (Rsb 4.23, 91/91) | Rsb | both | pac | 91 | 4.234 | 3.014 |
| css1100 | 6B | 124 | 175 | 51000 | pac (xpEHH 4.36, 185/186) | xpEHH | both | pac | 185 | 4.363 | 3.035 |
| css1101 | 6B | 124 | 143 | 19000 | pac2 (xpEHH 2.69, 83/84) | xpEHH | both | pac2 | 83 | 2.692 | 2.529 |
| css1102 | 6B | 124 | 143 | 19000 | pac2 (xpEHH 2.86, 83/84) | xpEHH | spr | pac2 | 83 | 2.858 | 2.710 |
| css1103 | 6B | 124 | 194 | 70000 | spr2 (xpEHH 4.55, 208/209) | xpEHH | spr | spr2 | 208 | 4.552 | 3.262 |
| css1104 | 6B | 126 | 191 | 65000 | ID (Rsb 5.59, 201/202) | Rsb | both | ID | 201 | 5.595 | 3.186 |
| css1105 | 6B | 126 | 194 | 68000 | pnw2 (xpEHH 5.58, 204/205) | xpEHH | both | pnw2 | 204 | 5.584 | 3.163 |
| css1106 | 6B | 126 | 143 | 17000 | SRW2 (Rsb 3.38, 83/86) | Rsb | win | SRW2 | 83 | 3.379 | 2.460 |
| css1107 | 6B | 128 | 163 | 35000 | pnw2 (Rsb 7.17, 156/160) | Rsb | both | pnw2 | 156 | 7.174 | 3.091 |
| css1108 | 6B | 129 | 134 | 5000 | SC (xpEHH 2.57, 47/51) | xpEHH | win | SC | 47 | 2.568 | 2.185 |
| css1109 | 6B | 129 | 141 | 12000 | SRW2 (xpEHH 3.00, 69/73) | xpEHH | win | SRW2 | 69 | 3.004 | 2.421 |
| css1110 | 6B | 129 | 143 | 14000 | SWS2 (xpEHH 3.30, 46/48) | xpEHH | spr | SWS2 | 46 | 3.303 | 3.004 |
| css1111 | 6B | 130 | 143 | 13000 | ID (xpEHH 3.06, 65/66) | xpEHH | both | ID | 65 | 3.062 | 2.849 |
| css1112 | 6B | 130 | 143 | 13000 | SWS2 (Rsb 4.18, 39/39) | Rsb | spr | SWS2 | 39 | 4.181 | 2.574 |
| css1113 | 6B | 133 | 138 | 5000 | all2 (Rsb 3.62, 32/34) | Rsb | both | all2 | 32 | 3.624 | 2.423 |
| css1114 | 6B | 133 | 141 | 8000 | ID (xpEHH 2.23, 43/44) | xpEHH | spr | ID | 43 | 2.229 | 2.155 |
| css1115 | 6B | 133 | 138 | 5000 | nor2 (xpEHH 2.89, 31/33) | xpEHH | spr | nor2 | 31 | 2.888 | 2.634 |
| css1116 | 6B | 133 | 141 | 8000 | SC (xpEHH 2.22, 38/42) | xpEHH | both | SC | 38 | 2.224 | 2.143 |
| css1117 | 6B | 134 | 151 | 17000 | SC (Rsb 3.53, 29/31) | Rsb | win | SC | 29 | 3.532 | 2.752 |
| css1118 | 6B | 134 | 143 | 9000 | SC (Rsb 3.67, 18/20) | Rsb | both | SC | 18 | 3.675 | 2.670 |
| css1119 | 6B | 142 | 160 | 18000 | nor2 (xpEHH 2.77, 56/57) | xpEHH | both | nor2 | 56 | 2.773 | 2.566 |
| css1120 | 6B | 145 | 194 | 49000 | ID (xpEHH 4.64, 116/116) | xpEHH | both | ID | 116 | 4.638 | 2.933 |
| css1121 | 6B | 145 | 172 | 27000 | MN (xpEHH 4.11, 90/90) | xpEHH | both | MN | 90 | 4.111 | 2.793 |
| css1122 | 6B | 145 | 165 | 20000 | pac2 (xpEHH 3.10, 78/78) | xpEHH | both | pac2 | 78 | 3.097 | 2.767 |
| css1123 | 6B | 145 | 168 | 23000 | pac2 (xpEHH 3.28, 81/81) | xpEHH | spr | pac2 | 81 | 3.283 | 2.923 |
| css1124 | 6B | 145 | 168 | 23000 | SRW2 (Rsb 5.28, 85/85) | Rsb | win | SRW2 | 85 | 5.282 | 3.112 |
| css1125 | 6B | 145 | 165 | 20000 | SWS2 (xpEHH 4.37, 80/80) | xpEHH | spr | SWS2 | 80 | 4.374 | 3.589 |
| css1126 | 6B | 146 | 168 | 22000 | all2 (Rsb 7.53, 81/85) | Rsb | both | all2 | 81 | 7.530 | 3.904 |
| css1127 | 6B | 146 | 152 | 6000 | eas (Rsb 3.08, 10/11) | Rsb | win | eas | 10 | 3.081 | 2.460 |
| css1128 | 6B | 146 | 168 | 22000 | eas2 (Rsb 6.31, 82/85) | Rsb | win | eas2 | 82 | 6.307 | 3.351 |
| css1129 | 6B | 146 | 168 | 22000 | eas2 (Rsb 6.42, 82/85) | Rsb | both | eas2 | 82 | 6.425 | 3.359 |
| css1130 | 6B | 146 | 151 | 5000 | IN (Rsb 2.35, 5/5) | Rsb | both | IN | 5 | 2.345 | 2.260 |
| css1131 | 6B | 146 | 165 | 19000 | IN (Rsb 3.62, 36/37) | Rsb | win | IN | 36 | 3.619 | 2.913 |
| css1132 | 6B | 146 | 175 | 29000 | SC (xpEHH 4.19, 91/92) | xpEHH | win | SC | 91 | 4.191 | 2.629 |
| css1133 | 6B | 146 | 152 | 6000 | SRW (Rsb 3.26, 10/11) | Rsb | win | SRW | 10 | 3.261 | 2.924 |
| css1134 | 6B | 147 | 153 | 6000 | IN (xpEHH 2.10, 11/11) | xpEHH | both | IN | 11 | 2.105 | 2.052 |
| css1135 | 6B | 148 | 168 | 20000 | CA (xpEHH 3.84, 78/78) | xpEHH | both | CA | 78 | 3.844 | 2.634 |
| css1136 | 6B | 148 | 168 | 20000 | SRW2 (xpEHH 5.17, 79/80) | xpEHH | win | SRW2 | 79 | 5.166 | 3.137 |
| css1137 | 6B | 149 | 175 | 26000 | pac (Rsb 4.56, 85/87) | Rsb | both | pac | 85 | 4.565 | 2.979 |
| css1138 | 6B | 150 | 165 | 15000 | CA (Rsb 4.31, 69/73) | Rsb | both | CA | 69 | 4.312 | 2.722 |
| css1139 | 6B | 150 | 165 | 15000 | ID (Rsb 4.34, 70/73) | Rsb | win | ID | 70 | 4.338 | 3.065 |
| css1140 | 6B | 153 | 160 | 7000 | IN (Rsb 3.25, 13/13) | Rsb | both | IN | 13 | 3.252 | 2.411 |
| css1141 | 6B | 153 | 163 | 10000 | pac2 (Rsb 3.40, 49/51) | Rsb | spr | pac2 | 49 | 3.402 | 2.375 |
| css1142 | 6B | 153 | 163 | 10000 | SRW (Rsb 4.82, 58/61) | Rsb | win | SRW | 58 | 4.825 | 3.173 |
| css1143 | 6B | 153 | 163 | 10000 | SWS2 (Rsb 4.16, 59/61) | Rsb | spr | SWS2 | 59 | 4.156 | 2.598 |
| css1144 | 6B | 154 | 168 | 14000 | IN (xpEHH 3.21, 49/49) | xpEHH | both | IN | 49 | 3.209 | 2.535 |
| css1145 | 6B | 154 | 168 | 14000 | nor (Rsb 4.36, 48/49) | Rsb | both | nor | 48 | 4.365 | 3.111 |
| css1146 | 6B | 155 | 163 | 8000 | pac2 (Rsb 3.34, 24/26) | Rsb | both | pac2 | 24 | 3.336 | 2.484 |
| css1147 | 6B | 156 | 168 | 12000 | eas2 (xpEHH 5.44, 36/36) | xpEHH | both | eas2 | 36 | 5.438 | 4.087 |
| css1148 | 6B | 156 | 168 | 12000 | eas2 (xpEHH 5.48, 36/36) | xpEHH | win | eas2 | 36 | 5.475 | 4.160 |
| css1149 | 6B | 156 | 165 | 9000 | ID (xpEHH 3.52, 33/33) | xpEHH | win | ID | 33 | 3.520 | 2.929 |
| css1150 | 6B | 156 | 166 | 10000 | ND (Rsb 3.36, 33/36) | Rsb | both | ND | 33 | 3.355 | 2.748 |
| css1151 | 6B | 156 | 161 | 5000 | pnw (Rsb 3.55, 12/13) | Rsb | both | pnw | 12 | 3.551 | 2.885 |
| css1152 | 6B | 156 | 168 | 12000 | SC (xpEHH 4.06, 35/35) | xpEHH | both | SC | 35 | 4.060 | 3.159 |
| css1153 | 6B | 158 | 163 | 5000 | ID (xpEHH 2.13, 22/22) | xpEHH | spr | ID | 22 | 2.135 | 2.076 |
| css1154 | 6B | 158 | 163 | 5000 | KS (Rsb 3.77, 19/21) | Rsb | win | KS | 19 | 3.768 | 2.966 |
| css1155 | 6B | 158 | 163 | 5000 | MT (Rsb 3.42, 21/22) | Rsb | both | MT | 21 | 3.425 | 2.809 |
| css1156 | 6B | 158 | 165 | 7000 | ND (xpEHH 3.13, 25/27) | xpEHH | both | ND | 25 | 3.135 | 2.688 |
| css1157 | 6B | 158 | 165 | 7000 | nor (xpEHH 3.25, 25/27) | xpEHH | both | nor | 25 | 3.250 | 2.629 |
| css1158 | 6B | 158 | 168 | 10000 | SC (Rsb 4.30, 28/29) | Rsb | win | SC | 28 | 4.300 | 3.393 |
| css1159 | 6B | 158 | 165 | 7000 | TX (xpEHH 3.59, 23/25) | xpEHH | both | TX | 23 | 3.589 | 2.748 |
| css1160 | 6B | 161 | 172 | 11000 | nor2 (xpEHH 2.62, 28/28) | xpEHH | both | nor2 | 28 | 2.617 | 2.206 |
| css1161 | 6B | 161 | 168 | 7000 | NY (xpEHH 3.18, 23/23) | xpEHH | win | NY | 23 | 3.184 | 2.569 |
| css1162 | 6B | 162 | 168 | 6000 | SC (Rsb 3.92, 16/17) | Rsb | both | SC | 16 | 3.922 | 2.949 |
| css1163 | 6B | 167 | 175 | 8000 | pnw2 (Rsb 3.19, 8/8) | Rsb | both | pnw2 | 8 | 3.188 | 2.476 |
| css1164 | 6B | 176 | 189 | 13000 | all2 (Rsb 3.68, 16/16) | Rsb | both | all2 | 16 | 3.679 | 3.172 |
| css1165 | 6B | 176 | 189 | 13000 | pac2 (Fst 0.59, 16/16) | Fst | spr | pac2 | 16 | 0.595 | 0.486 |
| css1166 | 6B | 176 | 187 | 11000 | pnw2 (Rsb 2.28, 14/14) | Rsb | spr | pnw2 | 14 | 2.283 | 2.171 |
| css1167 | 6B | 176 | 189 | 13000 | pnw2 (Rsb 4.07, 16/16) | Rsb | both | pnw2 | 16 | 4.070 | 3.363 |
| css1168 | 6B | 176 | 190 | 14000 | pnw2 (xpEHH 2.90, 17/17) | xpEHH | spr | pnw2 | 17 | 2.903 | 2.782 |
| css1169 | 6B | 181 | 187 | 6000 | all2 (Fst 0.11, 8/8) | Fst | both | all2 | 8 | 0.109 | 0.109 |
| css1170 | 6B | 181 | 187 | 6000 | gpl2 (Fst 0.13, 8/8) | Fst | win | gpl2 | 8 | 0.134 | 0.134 |
| css1171 | 6B | 181 | 187 | 6000 | gpl2 (Fst 0.17, 8/8) | Fst | both | gpl2 | 8 | 0.168 | 0.161 |
| css1172 | 6B | 181 | 187 | 6000 | gpl2 (Rsb 2.30, 8/8) | Rsb | win | gpl2 | 8 | 2.303 | 2.303 |
| css1173 | 6B | 181 | 187 | 6000 | gpl2 (Rsb 2.92, 8/8) | Rsb | both | gpl2 | 8 | 2.917 | 2.877 |
| css1174 | 6B | 181 | 187 | 6000 | HRW2 (Rsb 2.24, 8/8) | Rsb | win | HRW2 | 8 | 2.244 | 2.182 |
| css1175 | 6B | 181 | 187 | 6000 | ID (Fst 0.26, 8/8) | Fst | spr | ID | 8 | 0.255 | 0.252 |
| css1176 | 6B | 181 | 187 | 6000 | ID (Fst 0.28, 8/8) | Fst | both | ID | 8 | 0.283 | 0.271 |
| css1177 | 6B | 181 | 187 | 6000 | MN (Rsb 2.43, 8/8) | Rsb | both | MN | 8 | 2.428 | 2.411 |
| css1178 | 6B | 181 | 187 | 6000 | nor2 (Rsb 2.07, 8/8) | Rsb | both | nor2 | 8 | 2.073 | 2.073 |
| css1179 | 6B | 181 | 187 | 6000 | pac (Rsb 2.18, 8/8) | Rsb | both | pac | 8 | 2.180 | 2.163 |
| css1180 | 6B | 181 | 187 | 6000 | pnw2 (Fst 0.21, 8/8) | Fst | both | pnw2 | 8 | 0.209 | 0.198 |
| css1181 | 6B | 181 | 189 | 8000 | pnw2 (Fst 0.56, 10/10) | Fst | spr | pnw2 | 10 | 0.564 | 0.515 |
| css1182 | 6B | 181 | 189 | 8000 | spr2 (Fst 0.28, 10/10) | Fst | spr | spr2 | 10 | 0.278 | 0.259 |
| css1183 | 6B | 181 | 189 | 8000 | SWS2 (Fst 0.63, 10/10) | Fst | spr | SWS2 | 10 | 0.629 | 0.592 |
| css1184 | 6B | 181 | 187 | 6000 | SWS2 (Rsb 2.21, 8/8) | Rsb | spr | SWS2 | 8 | 2.208 | 2.208 |
| css1185 | 6B | 186 | 191 | 5000 | SC (Rsb 2.13, 5/5) | Rsb | win | SC | 5 | 2.125 | 2.085 |
| css1186 | 6B | 188 | 194 | 6000 | all2 (xpEHH 2.62, 6/6) | xpEHH | both | all2 | 6 | 2.623 | 2.350 |
| css1187 | 6B | 195 | 218 | 23000 | ID (xpEHH 3.09, 19/19) | xpEHH | both | ID | 19 | 3.091 | 2.665 |
| css1188 | 6B | 195 | 201 | 6000 | MN (xpEHH 2.37, 5/5) | xpEHH | both | MN | 5 | 2.365 | 2.238 |
| css1189 | 6B | 195 | 218 | 23000 | pnw2 (xpEHH 3.64, 19/19) | xpEHH | both | pnw2 | 19 | 3.641 | 3.149 |
| css1190 | 6B | 195 | 218 | 23000 | spr2 (xpEHH 3.13, 19/19) | xpEHH | spr | spr2 | 19 | 3.130 | 2.823 |
| css1191 | 6B | 197 | 203 | 6000 | ID (Rsb 4.36, 5/5) | Rsb | both | ID | 5 | 4.364 | 2.949 |
| css1192 | 6B | 197 | 212 | 15000 | spr2 (Rsb 3.97, 12/12) | Rsb | spr | spr2 | 12 | 3.970 | 2.745 |
| css1193 | 6B | 203 | 218 | 15000 | all2 (Rsb 3.75, 13/13) | Rsb | both | all2 | 13 | 3.753 | 2.867 |
| css1194 | 6B | 203 | 218 | 15000 | all2 (xpEHH 2.63, 13/13) | xpEHH | both | all2 | 13 | 2.631 | 2.406 |
| css1195 | 6B | 203 | 218 | 15000 | pnw2 (Rsb 2.67, 13/13) | Rsb | spr | pnw2 | 13 | 2.673 | 2.342 |
| css1196 | 6B | 203 | 212 | 9000 | pnw2 (Rsb 4.01, 7/7) | Rsb | both | pnw2 | 7 | 4.011 | 3.307 |
| css1197 | 6B | 203 | 218 | 15000 | pnw2 (xpEHH 3.01, 13/13) | xpEHH | spr | pnw2 | 13 | 3.015 | 2.926 |
| css1198 | 6B | 203 | 218 | 15000 | SWS2 (Fst 0.63, 13/13) | Fst | spr | SWS2 | 13 | 0.629 | 0.513 |
| css1199 | 6B | 203 | 218 | 15000 | SWS2 (xpEHH 2.48, 13/13) | xpEHH | spr | SWS2 | 13 | 2.479 | 2.391 |
| css1200 | 6B | 206 | 218 | 12000 | CA (Fst 0.29, 11/11) | Fst | spr | CA | 11 | 0.291 | 0.217 |
| css1201 | 6B | 210 | 218 | 8000 | MN (xpEHH 2.19, 6/6) | xpEHH | both | MN | 6 | 2.193 | 2.112 |
| css1202 | 6B | 228 | 240 | 12000 | all2 (xpEHH 2.49, 28/29) | xpEHH | both | all2 | 28 | 2.486 | 2.340 |
| css1203 | 6B | 228 | 240 | 12000 | ID (xpEHH 2.71, 28/28) | xpEHH | both | ID | 28 | 2.707 | 2.443 |
| css1204 | 6B | 228 | 240 | 12000 | pnw2 (Rsb 2.97, 26/27) | Rsb | both | pnw2 | 26 | 2.971 | 2.357 |
| css1205 | 6B | 228 | 243 | 15000 | pnw2 (xpEHH 3.28, 33/33) | xpEHH | both | pnw2 | 33 | 3.283 | 2.989 |
| css1206 | 6B | 228 | 243 | 15000 | spr2 (xpEHH 2.83, 33/33) | xpEHH | spr | spr2 | 33 | 2.826 | 2.698 |
| css1207 | 6B | 231 | 240 | 9000 | pnw2 (Rsb 2.78, 15/15) | Rsb | spr | pnw2 | 15 | 2.778 | 2.341 |
| css1208 | 6B | 231 | 240 | 9000 | pnw2 (xpEHH 3.02, 15/15) | xpEHH | spr | pnw2 | 15 | 3.020 | 2.922 |
| css1209 | 6B | 231 | 242 | 11000 | SWS2 (xpEHH 2.48, 16/16) | xpEHH | spr | SWS2 | 16 | 2.476 | 2.429 |
| css1210 | 6B | 232 | 242 | 10000 | spr2 (Rsb 3.54, 26/28) | Rsb | spr | spr2 | 26 | 3.543 | 2.704 |
| css1211 | 6B | 233 | 240 | 7000 | all2 (Rsb 3.00, 20/21) | Rsb | both | all2 | 20 | 2.996 | 2.645 |
| css1212 | 6B | 258 | 265 | 7000 | gpl2 (Fst 0.13, 12/13) | Fst | both | gpl2 | 12 | 0.133 | 0.133 |
| css1213 | 6B | 258 | 265 | 7000 | IN (Fst 0.39, 12/13) | Fst | win | IN | 12 | 0.387 | 0.382 |
| css1214 | 6B | 258 | 272 | 14000 | NY (Fst 0.45, 29/29) | Fst | win | NY | 29 | 0.449 | 0.432 |
| css1215 | 6B | 258 | 272 | 14000 | pnw2 (Fst 0.52, 29/29) | Fst | spr | pnw2 | 29 | 0.520 | 0.520 |
| css1216 | 6B | 276 | 289 | 13000 | pnw2 (Fst 0.55, 13/13) | Fst | spr | pnw2 | 13 | 0.550 | 0.522 |
| css1217 | 6B | 315 | 320 | 5000 | pnw2 (Fst 0.14, 5/5) | Fst | both | pnw2 | 5 | 0.142 | 0.142 |
| css1218 | 6B | 315 | 320 | 5000 | pnw2 (Fst 0.52, 5/5) | Fst | spr | pnw2 | 5 | 0.520 | 0.520 |
| css1219 | 6B | 421 | 435 | 14000 | pnw2 (Fst 0.66, 15/15) | Fst | spr | pnw2 | 15 | 0.658 | 0.514 |
| css1220 | 6B | 425 | 435 | 10000 | pnw2 (xpEHH 2.51, 12/12) | xpEHH | spr | pnw2 | 12 | 2.512 | 2.277 |
| css1221 | 6B | 430 | 435 | 5000 | spr2 (xpEHH 2.52, 7/7) | xpEHH | spr | spr2 | 7 | 2.519 | 2.389 |
| css1222 | 6B | 438 | 443 | 5000 | pac2 (xpEHH 2.09, 5/5) | xpEHH | both | pac2 | 5 | 2.093 | 2.091 |
| css1223 | 6B | 438 | 443 | 5000 | pac2 (xpEHH 2.11, 5/5) | xpEHH | spr | pac2 | 5 | 2.108 | 2.108 |
| css1224 | 6B | 438 | 443 | 5000 | pnw2 (Fst 0.52, 5/5) | Fst | spr | pnw2 | 5 | 0.520 | 0.484 |
| css1225 | 6B | 438 | 443 | 5000 | pnw2 (xpEHH 2.34, 5/5) | xpEHH | spr | pnw2 | 5 | 2.339 | 2.335 |
| css1226 | 6B | 438 | 443 | 5000 | spr2 (xpEHH 2.29, 5/5) | xpEHH | spr | spr2 | 5 | 2.292 | 2.281 |
| css1227 | 6B | 445 | 451 | 6000 | pnw2 (xpEHH 3.15, 5/5) | xpEHH | both | pnw2 | 5 | 3.154 | 2.846 |
| css1228 | 6B | 445 | 451 | 6000 | spr2 (Rsb 3.18, 5/5) | Rsb | spr | spr2 | 5 | 3.175 | 2.628 |
| css1229 | 6B | 445 | 451 | 6000 | spr2 (xpEHH 3.06, 5/5) | xpEHH | spr | spr2 | 5 | 3.060 | 2.835 |
| css1230 | 6B | 463 | 497 | 34000 | all2 (Rsb 7.90, 68/68) | Rsb | both | all2 | 68 | 7.897 | 4.905 |
| css1231 | 6B | 463 | 497 | 34000 | all2 (xpEHH 7.47, 68/68) | xpEHH | both | all2 | 68 | 7.470 | 5.523 |
| css1232 | 6B | 463 | 485 | 22000 | eas2 (Rsb 4.17, 56/57) | Rsb | both | eas2 | 56 | 4.169 | 2.973 |
| css1233 | 6B | 463 | 485 | 22000 | eas2 (xpEHH 4.93, 56/57) | xpEHH | win | eas2 | 56 | 4.926 | 3.685 |
| css1234 | 6B | 463 | 488 | 25000 | eas2 (xpEHH 5.10, 58/58) | xpEHH | both | eas2 | 58 | 5.104 | 3.681 |
| css1235 | 6B | 463 | 483 | 20000 | ID (Rsb 3.91, 47/50) | Rsb | both | ID | 47 | 3.906 | 2.743 |
| css1236 | 6B | 463 | 488 | 25000 | ID (xpEHH 4.19, 58/58) | xpEHH | both | ID | 58 | 4.189 | 3.446 |
| css1237 | 6B | 463 | 497 | 34000 | MN (xpEHH 3.40, 68/68) | xpEHH | both | MN | 68 | 3.396 | 2.891 |
| css1238 | 6B | 463 | 497 | 34000 | nor (xpEHH 3.72, 68/68) | xpEHH | both | nor | 68 | 3.719 | 3.001 |
| css1239 | 6B | 463 | 497 | 34000 | pac2 (xpEHH 2.79, 60/60) | xpEHH | both | pac2 | 60 | 2.792 | 2.456 |
| css1240 | 6B | 463 | 497 | 34000 | pac2 (xpEHH 2.79, 60/60) | xpEHH | spr | pac2 | 60 | 2.789 | 2.456 |
| css1241 | 6B | 463 | 475 | 12000 | pnw (Rsb 4.30, 19/20) | Rsb | both | pnw | 19 | 4.305 | 2.632 |
| css1242 | 6B | 463 | 483 | 20000 | pnw (xpEHH 3.92, 50/50) | xpEHH | both | pnw | 50 | 3.921 | 3.192 |
| css1243 | 6B | 463 | 497 | 34000 | pnw2 (Rsb 4.10, 64/64) | Rsb | spr | pnw2 | 64 | 4.103 | 2.720 |
| css1244 | 6B | 463 | 497 | 34000 | pnw2 (xpEHH 3.46, 64/64) | xpEHH | spr | pnw2 | 64 | 3.458 | 3.120 |
| css1245 | 6B | 463 | 497 | 34000 | pnw2 (xpEHH 4.12, 68/68) | xpEHH | both | pnw2 | 68 | 4.122 | 3.527 |
| css1246 | 6B | 463 | 497 | 34000 | spr2 (Rsb 4.11, 68/68) | Rsb | spr | spr2 | 68 | 4.111 | 3.078 |
| css1247 | 6B | 463 | 497 | 34000 | spr2 (xpEHH 3.61, 68/68) | xpEHH | spr | spr2 | 68 | 3.615 | 3.248 |
| css1248 | 6B | 463 | 483 | 20000 | SRW2 (xpEHH 3.98, 50/50) | xpEHH | win | SRW2 | 50 | 3.978 | 3.154 |
| css1249 | 6B | 463 | 470 | 7000 | SWS2 (Rsb 3.28, 8/8) | Rsb | spr | SWS2 | 8 | 3.281 | 2.537 |
| css1250 | 6B | 463 | 488 | 25000 | SWS2 (xpEHH 3.20, 54/54) | xpEHH | spr | SWS2 | 54 | 3.200 | 2.681 |
| css1251 | 6B | 463 | 497 | 34000 | WA (xpEHH 3.45, 68/68) | xpEHH | both | WA | 68 | 3.447 | 2.734 |
| css1252 | 6B | 465 | 470 | 5000 | CA (Fst 0.24, 6/6) | Fst | spr | CA | 6 | 0.241 | 0.200 |
| css1253 | 6B | 465 | 497 | 32000 | pac (xpEHH 3.07, 66/66) | xpEHH | both | pac | 66 | 3.074 | 2.556 |
| css1254 | 6B | 465 | 485 | 20000 | WA (xpEHH 2.90, 54/55) | xpEHH | win | WA | 54 | 2.900 | 2.439 |
| css1255 | 6B | 466 | 485 | 19000 | eas2 (Rsb 4.08, 53/54) | Rsb | win | eas2 | 53 | 4.076 | 2.955 |
| css1256 | 6B | 466 | 497 | 31000 | nor2 (xpEHH 2.37, 64/65) | xpEHH | both | nor2 | 64 | 2.367 | 2.117 |
| css1257 | 6B | 466 | 488 | 22000 | pnw2 (Rsb 5.11, 55/55) | Rsb | both | pnw2 | 55 | 5.107 | 3.119 |
| css1258 | 6B | 466 | 483 | 17000 | SC (xpEHH 2.40, 46/47) | xpEHH | win | SC | 46 | 2.401 | 2.225 |
| css1259 | 6B | 468 | 483 | 15000 | pnw (xpEHH 2.88, 43/44) | xpEHH | win | pnw | 43 | 2.879 | 2.423 |
| css1260 | 6B | 470 | 483 | 13000 | MT (xpEHH 3.13, 40/41) | xpEHH | both | MT | 40 | 3.127 | 2.645 |
| css1261 | 6B | 470 | 483 | 13000 | SRW2 (Rsb 3.58, 40/42) | Rsb | win | SRW2 | 40 | 3.582 | 2.609 |
| css1262 | 6B | 473 | 483 | 10000 | NE (xpEHH 2.53, 33/34) | xpEHH | win | NE | 33 | 2.532 | 2.287 |
| css1263 | 6B | 474 | 483 | 9000 | CA (xpEHH 2.71, 27/27) | xpEHH | both | CA | 27 | 2.708 | 2.457 |
| css1264 | 6B | 474 | 482 | 8000 | IN (xpEHH 2.27, 27/28) | xpEHH | win | IN | 27 | 2.275 | 2.202 |
| css1265 | 6B | 475 | 482 | 7000 | OR (xpEHH 2.35, 21/23) | xpEHH | win | OR | 21 | 2.349 | 2.179 |
| css1266 | 6B | 475 | 497 | 22000 | pac2 (Rsb 3.36, 41/44) | Rsb | both | pac2 | 41 | 3.358 | 2.200 |
| css1267 | 6B | 476 | 483 | 7000 | MN (Rsb 3.18, 27/29) | Rsb | both | MN | 27 | 3.183 | 2.444 |
| css1268 | 6B | 477 | 483 | 6000 | ND (xpEHH 2.65, 26/28) | xpEHH | both | ND | 26 | 2.647 | 2.364 |
| css1269 | 6B | 477 | 485 | 8000 | NE (xpEHH 2.25, 32/35) | xpEHH | both | NE | 32 | 2.249 | 2.140 |
| css1270 | 6B | 477 | 482 | 5000 | SWW (xpEHH 2.32, 23/25) | xpEHH | win | SWW | 23 | 2.318 | 2.165 |
| css1271 | 6B | 480 | 488 | 8000 | nor2 (Rsb 2.80, 11/11) | Rsb | both | nor2 | 11 | 2.798 | 2.308 |
| css1272 | 6B | 486 | 497 | 11000 | pac2 (Rsb 3.37, 10/10) | Rsb | spr | pac2 | 10 | 3.369 | 2.165 |
| css1273 | 6B | 486 | 497 | 11000 | WA (xpEHH 2.20, 10/10) | xpEHH | win | WA | 10 | 2.204 | 2.126 |
| css1274 | 6B | 489 | 497 | 8000 | eas2 (Rsb 2.29, 9/10) | Rsb | both | eas2 | 9 | 2.293 | 2.108 |
| css1275 | 6B | 489 | 497 | 8000 | eas2 (Rsb 2.34, 9/10) | Rsb | win | eas2 | 9 | 2.342 | 2.145 |
| css1276 | 6B | 522 | 544 | 22000 | all2 (Rsb 4.95, 35/35) | Rsb | both | all2 | 35 | 4.948 | 3.032 |
| css1277 | 6B | 522 | 544 | 22000 | all2 (xpEHH 2.93, 35/35) | xpEHH | both | all2 | 35 | 2.927 | 2.522 |
| css1278 | 6B | 522 | 544 | 22000 | MN (xpEHH 2.46, 34/34) | xpEHH | both | MN | 34 | 2.459 | 2.234 |
| css1279 | 6B | 522 | 544 | 22000 | nor2 (xpEHH 2.64, 34/34) | xpEHH | both | nor2 | 34 | 2.641 | 2.507 |
| css1280 | 6B | 522 | 539 | 17000 | pac2 (Rsb 3.02, 25/26) | Rsb | both | pac2 | 25 | 3.023 | 2.234 |
| css1281 | 6B | 522 | 544 | 22000 | pac2 (xpEHH 2.87, 33/33) | xpEHH | both | pac2 | 33 | 2.868 | 2.529 |
| css1282 | 6B | 522 | 544 | 22000 | pac2 (xpEHH 2.88, 33/33) | xpEHH | spr | pac2 | 33 | 2.881 | 2.558 |
| css1283 | 6B | 522 | 544 | 22000 | pnw2 (Rsb 3.27, 34/34) | Rsb | spr | pnw2 | 34 | 3.267 | 2.710 |
| css1284 | 6B | 522 | 544 | 22000 | pnw2 (xpEHH 3.20, 34/34) | xpEHH | spr | pnw2 | 34 | 3.201 | 2.943 |
| css1285 | 6B | 522 | 544 | 22000 | spr2 (Rsb 3.74, 35/35) | Rsb | spr | spr2 | 35 | 3.737 | 2.768 |
| css1286 | 6B | 522 | 544 | 22000 | spr2 (xpEHH 3.68, 35/35) | xpEHH | spr | spr2 | 35 | 3.678 | 3.339 |
| css1287 | 6B | 523 | 532 | 9000 | pnw2 (xpEHH 2.54, 15/16) | xpEHH | both | pnw2 | 15 | 2.541 | 2.453 |
| css1288 | 6B | 532 | 538 | 6000 | pnw2 (Rsb 2.06, 6/6) | Rsb | both | pnw2 | 6 | 2.061 | 2.032 |
| css1289 | 6B | 538 | 544 | 6000 | HRS2 (xpEHH 2.08, 10/11) | xpEHH | spr | HRS2 | 10 | 2.083 | 2.049 |
| css1290 | 6B | 560 | 584 | 24000 | pac2 (Rsb 2.58, 62/62) | Rsb | spr | pac2 | 62 | 2.584 | 2.311 |
| css1291 | 6B | 560 | 584 | 24000 | pac2 (Rsb 2.87, 62/62) | Rsb | both | pac2 | 62 | 2.867 | 2.417 |
| css1292 | 6B | 560 | 590 | 30000 | pac2 (xpEHH 2.98, 78/79) | xpEHH | spr | pac2 | 78 | 2.983 | 2.733 |
| css1293 | 6B | 560 | 590 | 30000 | pac2 (xpEHH 3.15, 79/79) | xpEHH | both | pac2 | 79 | 3.151 | 2.791 |
| css1294 | 6B | 560 | 590 | 30000 | pnw2 (Rsb 4.20, 78/78) | Rsb | spr | pnw2 | 78 | 4.204 | 2.863 |
| css1295 | 6B | 560 | 590 | 30000 | pnw2 (xpEHH 3.22, 78/78) | xpEHH | spr | pnw2 | 78 | 3.215 | 3.055 |
| css1296 | 6B | 560 | 590 | 30000 | pnw2 (xpEHH 3.22, 79/79) | xpEHH | both | pnw2 | 79 | 3.219 | 2.760 |
| css1297 | 6B | 560 | 590 | 30000 | spr2 (Rsb 4.36, 79/79) | Rsb | spr | spr2 | 79 | 4.356 | 2.914 |
| css1298 | 6B | 560 | 590 | 30000 | spr2 (xpEHH 3.62, 79/79) | xpEHH | spr | spr2 | 79 | 3.618 | 3.207 |
| css1299 | 6B | 560 | 590 | 30000 | WA (xpEHH 3.37, 78/78) | xpEHH | spr | WA | 78 | 3.365 | 2.770 |
| css1300 | 6B | 561 | 570 | 9000 | MN (xpEHH 2.24, 19/19) | xpEHH | both | MN | 19 | 2.241 | 2.174 |
| css1301 | 6B | 561 | 575 | 14000 | NY (xpEHH 2.10, 30/31) | xpEHH | win | NY | 30 | 2.095 | 2.035 |
| css1302 | 6B | 565 | 575 | 10000 | all2 (Rsb 3.81, 30/31) | Rsb | both | all2 | 30 | 3.814 | 2.442 |
| css1303 | 6B | 570 | 575 | 5000 | pnw2 (Fst 0.65, 16/16) | Fst | spr | pnw2 | 16 | 0.650 | 0.494 |
| css1304 | 6B | 570 | 590 | 20000 | WA (Rsb 3.55, 58/58) | Rsb | spr | WA | 58 | 3.548 | 2.702 |
| css1305 | 6B | 576 | 590 | 14000 | HRS2 (xpEHH 2.13, 39/40) | xpEHH | spr | HRS2 | 39 | 2.127 | 2.053 |
| css1306 | 6B | 580 | 585 | 5000 | pnw2 (Fst 0.53, 18/20) | Fst | spr | pnw2 | 18 | 0.532 | 0.405 |
| css1307 | 6B | 583 | 590 | 7000 | all2 (Rsb 3.78, 17/18) | Rsb | both | all2 | 17 | 3.779 | 2.873 |
| css1308 | 6B | 583 | 590 | 7000 | NE (xpEHH 2.61, 17/18) | xpEHH | both | NE | 17 | 2.608 | 2.270 |
| css1309 | 6B | 583 | 590 | 7000 | pnw2 (Rsb 4.36, 18/18) | Rsb | both | pnw2 | 18 | 4.356 | 3.453 |
| css1310 | 6B | 599 | 607 | 8000 | NE (xpEHH 2.82, 19/20) | xpEHH | both | NE | 19 | 2.823 | 2.125 |
| css1311 | 6B | 599 | 626 | 27000 | pnw (xpEHH 2.53, 40/41) | xpEHH | spr | pnw | 40 | 2.528 | 2.108 |
| css1312 | 6B | 599 | 619 | 20000 | pnw2 (Fst 0.58, 34/35) | Fst | spr | pnw2 | 34 | 0.576 | 0.484 |
| css1313 | 6B | 599 | 619 | 20000 | pnw2 (Rsb 3.43, 35/35) | Rsb | spr | pnw2 | 35 | 3.428 | 3.061 |
| css1314 | 6B | 599 | 619 | 20000 | pnw2 (xpEHH 3.37, 35/35) | xpEHH | spr | pnw2 | 35 | 3.370 | 2.504 |
| css1315 | 6B | 599 | 626 | 27000 | spr2 (Rsb 3.56, 40/41) | Rsb | spr | spr2 | 40 | 3.555 | 2.809 |
| css1316 | 6B | 599 | 609 | 10000 | spr2 (xpEHH 2.80, 23/23) | xpEHH | spr | spr2 | 23 | 2.803 | 2.137 |
| css1317 | 6B | 599 | 619 | 20000 | WA (Rsb 3.75, 35/35) | Rsb | spr | WA | 35 | 3.752 | 3.006 |
| css1318 | 6B | 599 | 619 | 20000 | WA (xpEHH 3.66, 35/35) | xpEHH | spr | WA | 35 | 3.660 | 3.127 |
| css1319 | 6B | 600 | 612 | 12000 | gpl2 (Fst 0.16, 22/23) | Fst | win | gpl2 | 22 | 0.158 | 0.136 |
| css1320 | 6B | 602 | 616 | 14000 | all2 (Rsb 2.96, 29/31) | Rsb | both | all2 | 29 | 2.959 | 2.729 |
| css1321 | 6B | 602 | 609 | 7000 | gpl (Rsb 2.34, 19/20) | Rsb | both | gpl | 19 | 2.338 | 2.225 |
| css1322 | 6B | 602 | 607 | 5000 | gpl (Rsb 2.60, 16/17) | Rsb | win | gpl | 16 | 2.605 | 2.543 |
| css1323 | 6B | 602 | 607 | 5000 | KS (Rsb 2.29, 16/17) | Rsb | win | KS | 16 | 2.289 | 2.265 |
| css1324 | 6B | 602 | 607 | 5000 | MN (Rsb 2.32, 16/17) | Rsb | both | MN | 16 | 2.319 | 2.291 |
| css1325 | 6B | 602 | 607 | 5000 | pnw2 (Rsb 3.58, 16/17) | Rsb | both | pnw2 | 16 | 3.579 | 3.504 |
| css1326 | 6B | 610 | 617 | 7000 | pnw2 (xpEHH 2.47, 11/11) | xpEHH | both | pnw2 | 11 | 2.472 | 2.122 |
| css1327 | 6B | 611 | 616 | 5000 | pnw2 (Rsb 2.69, 9/10) | Rsb | both | pnw2 | 9 | 2.686 | 2.591 |
| css1328 | 6B | 618 | 626 | 8000 | pac2 (xpEHH 2.14, 6/6) | xpEHH | both | pac2 | 6 | 2.143 | 2.084 |
| css1329 | 6B | 618 | 626 | 8000 | spr2 (xpEHH 2.81, 6/6) | xpEHH | spr | spr2 | 6 | 2.812 | 2.676 |
| css1330 | 6B | 636 | 643 | 7000 | pac2 (xpEHH 2.16, 15/15) | xpEHH | spr | pac2 | 15 | 2.155 | 2.084 |
| css1331 | 6B | 636 | 648 | 12000 | pac2 (xpEHH 2.79, 22/22) | xpEHH | both | pac2 | 22 | 2.794 | 2.192 |
| css1332 | 6B | 636 | 648 | 12000 | pnw (xpEHH 2.89, 22/22) | xpEHH | spr | pnw | 22 | 2.889 | 2.693 |
| css1333 | 6B | 636 | 652 | 16000 | pnw2 (Rsb 4.08, 34/34) | Rsb | spr | pnw2 | 34 | 4.082 | 2.724 |
| css1334 | 6B | 636 | 648 | 12000 | pnw2 (xpEHH 3.26, 21/21) | xpEHH | spr | pnw2 | 21 | 3.264 | 2.827 |
| css1335 | 6B | 636 | 647 | 11000 | spr2 (xpEHH 3.12, 20/21) | xpEHH | spr | spr2 | 20 | 3.124 | 2.533 |
| css1336 | 6B | 636 | 680 | 44000 | WA (Rsb 5.44, 90/91) | Rsb | spr | WA | 90 | 5.441 | 3.472 |
| css1337 | 6B | 636 | 672 | 36000 | WA (xpEHH 4.78, 74/74) | xpEHH | spr | WA | 74 | 4.778 | 3.556 |
| css1338 | 6B | 639 | 648 | 9000 | spr2 (Rsb 4.10, 20/20) | Rsb | spr | spr2 | 20 | 4.101 | 2.850 |
| css1339 | 6B | 640 | 648 | 8000 | pnw2 (Rsb 4.09, 18/19) | Rsb | both | pnw2 | 18 | 4.086 | 3.020 |
| css1340 | 6B | 641 | 648 | 7000 | pnw2 (xpEHH 3.11, 16/17) | xpEHH | both | pnw2 | 16 | 3.110 | 2.543 |
| css1341 | 6B | 642 | 647 | 5000 | pnw (Rsb 2.90, 14/15) | Rsb | spr | pnw | 14 | 2.902 | 2.176 |
| css1342 | 6B | 645 | 665 | 20000 | gpl (Rsb 4.45, 36/37) | Rsb | both | gpl | 36 | 4.452 | 2.891 |
| css1343 | 6B | 645 | 661 | 16000 | gpl (xpEHH 3.97, 35/36) | xpEHH | win | gpl | 35 | 3.971 | 2.556 |
| css1344 | 6B | 645 | 665 | 20000 | NE (Rsb 4.17, 35/35) | Rsb | both | NE | 35 | 4.166 | 2.914 |
| css1345 | 6B | 645 | 665 | 20000 | NE (xpEHH 4.11, 34/35) | xpEHH | both | NE | 34 | 4.108 | 2.879 |
| css1346 | 6B | 645 | 652 | 7000 | SC (Rsb 3.13, 12/13) | Rsb | both | SC | 12 | 3.130 | 2.630 |
| css1347 | 6B | 646 | 656 | 10000 | TX (xpEHH 3.22, 22/23) | xpEHH | both | TX | 22 | 3.223 | 2.555 |
| css1348 | 6B | 648 | 657 | 9000 | gpl (xpEHH 4.06, 26/28) | xpEHH | both | gpl | 26 | 4.060 | 2.773 |
| css1349 | 6B | 648 | 665 | 17000 | NE (Rsb 3.78, 33/34) | Rsb | win | NE | 33 | 3.783 | 2.720 |
| css1350 | 6B | 650 | 658 | 8000 | eas2 (Rsb 4.30, 16/16) | Rsb | win | eas2 | 16 | 4.296 | 2.907 |
| css1351 | 6B | 650 | 658 | 8000 | eas2 (Rsb 4.45, 16/16) | Rsb | both | eas2 | 16 | 4.449 | 2.979 |
| css1352 | 6B | 650 | 665 | 15000 | gpl (Rsb 4.28, 22/24) | Rsb | win | gpl | 22 | 4.280 | 3.038 |
| css1353 | 6B | 650 | 665 | 15000 | NE (xpEHH 3.90, 22/22) | xpEHH | win | NE | 22 | 3.902 | 2.999 |
| css1354 | 6B | 650 | 656 | 6000 | TX (Rsb 3.37, 14/14) | Rsb | both | TX | 14 | 3.373 | 2.700 |
| css1355 | 6B | 651 | 656 | 5000 | KS (Rsb 3.30, 12/13) | Rsb | both | KS | 12 | 3.303 | 2.759 |
| css1356 | 6B | 651 | 656 | 5000 | win2 (Rsb 4.84, 12/13) | Rsb | win | win2 | 12 | 4.843 | 3.012 |
| css1357 | 6B | 653 | 658 | 5000 | SC (Rsb 3.18, 9/9) | Rsb | both | SC | 9 | 3.182 | 2.471 |
| css1358 | 6B | 653 | 658 | 5000 | SRW2 (Rsb 3.37, 9/9) | Rsb | win | SRW2 | 9 | 3.370 | 3.023 |
| css1359 | 6B | 655 | 665 | 10000 | gpl2 (xpEHH 2.58, 10/10) | xpEHH | both | gpl2 | 10 | 2.581 | 2.475 |
| css1360 | 6B | 655 | 665 | 10000 | HRW2 (xpEHH 2.87, 10/10) | xpEHH | win | HRW2 | 10 | 2.875 | 2.737 |
| css1361 | 6B | 655 | 661 | 6000 | IN (Rsb 2.99, 9/9) | Rsb | both | IN | 9 | 2.985 | 2.286 |
| css1362 | 6B | 655 | 661 | 6000 | win2 (xpEHH 2.55, 9/9) | xpEHH | win | win2 | 9 | 2.546 | 2.367 |
| css1363 | 6B | 665 | 670 | 5000 | HRS (Fst 0.40, 9/10) | Fst | spr | HRS | 9 | 0.402 | 0.385 |
| css1364 | 6B | 665 | 670 | 5000 | spr2 (Fst 0.17, 9/10) | Fst | spr | spr2 | 9 | 0.171 | 0.169 |
| css1365 | 6B | 665 | 670 | 5000 | SWS (Fst 0.37, 9/10) | Fst | spr | SWS | 9 | 0.372 | 0.369 |
| css1366 | 6B | 684 | 693 | 9000 | SWW2 (xpEHH 3.14, 16/17) | xpEHH | win | SWW2 | 16 | 3.137 | 2.679 |
| css1367 | 7A | 21 | 26 | 5000 | pnw (xpEHH 3.73, 30/32) | xpEHH | win | pnw | 30 | 3.734 | 2.511 |
| css1368 | 7A | 71 | 77 | 6000 | KS (Fst 0.21, 11/12) | Fst | win | KS | 11 | 0.209 | 0.207 |
| css1369 | 7A | 71 | 77 | 6000 | NY (Fst 0.46, 11/12) | Fst | win | NY | 11 | 0.462 | 0.456 |
| css1370 | 7A | 75 | 81 | 6000 | WA (Fst 0.34, 5/5) | Fst | win | WA | 5 | 0.341 | 0.321 |
| css1371 | 7A | 229 | 236 | 7000 | pnw2 (Fst 0.25, 6/6) | Fst | both | pnw2 | 6 | 0.252 | 0.237 |
| css1372 | 7A | 229 | 234 | 5000 | SWS2 (Fst 0.38, 5/5) | Fst | spr | SWS2 | 5 | 0.382 | 0.382 |
| css1373 | 7A | 233 | 242 | 9000 | CA (Fst 0.28, 18/19) | Fst | spr | CA | 18 | 0.279 | 0.239 |
| css1374 | 7A | 233 | 242 | 9000 | CO (Fst 0.21, 17/19) | Fst | both | CO | 17 | 0.212 | 0.165 |
| css1375 | 7A | 233 | 242 | 9000 | pnw2 (Fst 0.23, 19/19) | Fst | win | pnw2 | 19 | 0.232 | 0.174 |
| css1376 | 7A | 237 | 242 | 5000 | SWW2 (Fst 0.18, 14/15) | Fst | win | SWW2 | 14 | 0.185 | 0.185 |
| css1377 | 7A | 239 | 245 | 6000 | pnw (Fst 0.15, 5/5) | Fst | both | pnw | 5 | 0.147 | 0.119 |
| css1378 | 7A | 239 | 245 | 6000 | WA (Fst 0.19, 5/5) | Fst | both | WA | 5 | 0.186 | 0.179 |
| css1379 | 7A | 502 | 515 | 13000 | HRW (Fst 0.39, 19/21) | Fst | win | HRW | 19 | 0.395 | 0.375 |
| css1380 | 7A | 502 | 508 | 6000 | SRW (Fst 0.29, 5/5) | Fst | win | SRW | 5 | 0.294 | 0.283 |
| css1381 | 7A | 514 | 528 | 14000 | KS (xpEHH 3.89, 24/25) | xpEHH | both | KS | 24 | 3.892 | 3.177 |
| css1382 | 7A | 516 | 521 | 5000 | KS (Rsb 4.58, 13/14) | Rsb | both | KS | 13 | 4.584 | 2.845 |
| css1383 | 7A | 516 | 528 | 12000 | KS (xpEHH 3.33, 23/23) | xpEHH | win | KS | 23 | 3.334 | 2.957 |
| css1384 | 7A | 516 | 521 | 5000 | TX (xpEHH 2.46, 9/9) | xpEHH | both | TX | 9 | 2.461 | 2.393 |
| css1385 | 7A | 538 | 544 | 6000 | KS (Rsb 2.68, 9/9) | Rsb | both | KS | 9 | 2.677 | 2.458 |
| css1386 | 7A | 538 | 548 | 10000 | KS (Rsb 3.03, 12/12) | Rsb | win | KS | 12 | 3.031 | 2.531 |
| css1387 | 7A | 538 | 548 | 10000 | KS (xpEHH 3.16, 12/12) | xpEHH | both | KS | 12 | 3.156 | 2.927 |
| css1388 | 7A | 538 | 548 | 10000 | KS (xpEHH 3.64, 12/12) | xpEHH | win | KS | 12 | 3.639 | 3.288 |
| css1389 | 7A | 562 | 570 | 8000 | KS (Rsb 2.36, 6/6) | Rsb | both | KS | 6 | 2.362 | 2.325 |
| css1390 | 7A | 562 | 576 | 14000 | KS (Rsb 2.52, 15/15) | Rsb | win | KS | 15 | 2.522 | 2.325 |
| css1391 | 7A | 562 | 573 | 11000 | KS (xpEHH 2.58, 13/13) | xpEHH | win | KS | 13 | 2.575 | 2.341 |
| css1392 | 7A | 580 | 585 | 5000 | KS (xpEHH 2.30, 7/7) | xpEHH | win | KS | 7 | 2.299 | 2.209 |
| css1393 | 7A | 599 | 607 | 8000 | ID (Fst 0.25, 19/19) | Fst | win | ID | 19 | 0.254 | 0.249 |
| css1394 | 7A | 599 | 607 | 8000 | MT (Fst 0.26, 19/19) | Fst | both | MT | 19 | 0.264 | 0.255 |
| css1395 | 7A | 599 | 607 | 8000 | SRW (Fst 0.29, 19/19) | Fst | win | SRW | 19 | 0.290 | 0.281 |
| css1396 | 7A | 610 | 632 | 22000 | MT (xpEHH 3.01, 46/47) | xpEHH | both | MT | 46 | 3.014 | 2.563 |
| css1397 | 7A | 613 | 631 | 18000 | KS (Rsb 3.94, 46/49) | Rsb | win | KS | 46 | 3.938 | 2.668 |
| css1398 | 7A | 613 | 622 | 9000 | nor (xpEHH 2.45, 27/28) | xpEHH | win | nor | 27 | 2.453 | 2.271 |
| css1399 | 7A | 614 | 632 | 18000 | KS (xpEHH 2.78, 45/46) | xpEHH | win | KS | 45 | 2.782 | 2.438 |
| css1400 | 7A | 635 | 656 | 21000 | nor (xpEHH 3.15, 25/25) | xpEHH | win | nor | 25 | 3.154 | 2.699 |
| css1401 | 7A | 643 | 656 | 13000 | MT (xpEHH 3.19, 17/17) | xpEHH | both | MT | 17 | 3.190 | 2.852 |
| css1402 | 7A | 648 | 656 | 8000 | nor (Rsb 3.33, 12/12) | Rsb | win | nor | 12 | 3.329 | 2.816 |
| css1403 | 7A | 671 | 676 | 5000 | MT (Rsb 2.92, 5/5) | Rsb | both | MT | 5 | 2.915 | 2.654 |
| css1404 | 7A | 674 | 681 | 7000 | MT (xpEHH 2.77, 15/17) | xpEHH | both | MT | 15 | 2.768 | 2.540 |
| css1405 | 7A | 715 | 720 | 5000 | gpl (xpEHH 2.60, 5/5) | xpEHH | win | gpl | 5 | 2.601 | 2.324 |
| css1406 | 7B | 32 | 37 | 5000 | MT (xpEHH 2.33, 5/5) | xpEHH | both | MT | 5 | 2.328 | 2.242 |
| css1407 | 7B | 32 | 50 | 18000 | SWW2 (xpEHH 2.78, 33/34) | xpEHH | win | SWW2 | 33 | 2.782 | 2.324 |
| css1408 | 7B | 35 | 42 | 7000 | pnw2 (Rsb 2.73, 6/6) | Rsb | win | pnw2 | 6 | 2.733 | 2.423 |
| css1409 | 7B | 40 | 49 | 9000 | MT (Rsb 3.42, 13/14) | Rsb | both | MT | 13 | 3.416 | 3.141 |
| css1410 | 7B | 40 | 46 | 6000 | SWW2 (Rsb 3.04, 6/6) | Rsb | win | SWW2 | 6 | 3.043 | 2.289 |
| css1411 | 7B | 40 | 49 | 9000 | TX (Rsb 4.02, 18/19) | Rsb | both | TX | 18 | 4.023 | 2.803 |
| css1412 | 7B | 44 | 49 | 5000 | MT (xpEHH 2.33, 11/12) | xpEHH | both | MT | 11 | 2.331 | 2.200 |
| css1413 | 7B | 45 | 50 | 5000 | win2 (xpEHH 2.78, 13/14) | xpEHH | win | win2 | 13 | 2.777 | 2.531 |
| css1414 | 7B | 65 | 72 | 7000 | TX (xpEHH 3.16, 14/14) | xpEHH | both | TX | 14 | 3.164 | 2.323 |
| css1415 | 7B | 72 | 77 | 5000 | ND (Fst 0.46, 5/5) | Fst | spr | ND | 5 | 0.464 | 0.389 |
| css1416 | 7B | 110 | 120 | 10000 | HRW (Rsb 2.62, 11/11) | Rsb | win | HRW | 11 | 2.615 | 2.365 |
| css1417 | 7B | 110 | 120 | 10000 | nor (Rsb 2.46, 11/11) | Rsb | win | nor | 11 | 2.457 | 2.273 |
| css1418 | 7B | 114 | 120 | 6000 | IN (Fst 0.41, 8/8) | Fst | both | IN | 8 | 0.412 | 0.381 |
| css1419 | 7B | 136 | 141 | 5000 | nor (Rsb 2.23, 5/5) | Rsb | win | nor | 5 | 2.227 | 2.197 |
| css1420 | 7B | 136 | 143 | 7000 | WA (Fst 0.22, 6/6) | Fst | spr | WA | 6 | 0.217 | 0.199 |
| css1421 | 7B | 142 | 148 | 6000 | gpl (Fst 0.27, 9/10) | Fst | spr | gpl | 9 | 0.270 | 0.222 |
| css1422 | 7B | 455 | 460 | 5000 | eas2 (Fst 0.17, 7/7) | Fst | both | eas2 | 7 | 0.174 | 0.149 |
| css1423 | 7B | 489 | 505 | 16000 | TX (xpEHH 2.90, 17/17) | xpEHH | both | TX | 17 | 2.899 | 2.604 |
| css1424 | 7B | 492 | 501 | 9000 | gpl2 (Fst 0.22, 13/14) | Fst | both | gpl2 | 13 | 0.217 | 0.204 |
| css1425 | 7B | 492 | 503 | 11000 | HRS2 (xpEHH 2.25, 17/18) | xpEHH | spr | HRS2 | 17 | 2.252 | 2.129 |
| css1426 | 7B | 492 | 515 | 23000 | KS (xpEHH 2.38, 23/24) | xpEHH | both | KS | 23 | 2.376 | 2.087 |
| css1427 | 7B | 493 | 501 | 8000 | HRS2 (Fst 0.15, 12/13) | Fst | spr | HRS2 | 12 | 0.148 | 0.141 |
| css1428 | 7B | 495 | 501 | 6000 | all2 (Fst 0.09, 7/7) | Fst | both | all2 | 7 | 0.087 | 0.080 |
| css1429 | 7B | 495 | 507 | 12000 | eas2 (Fst 0.42, 12/12) | Fst | win | eas2 | 12 | 0.425 | 0.302 |
| css1430 | 7B | 495 | 507 | 12000 | eas2 (Fst 0.44, 12/12) | Fst | both | eas2 | 12 | 0.440 | 0.280 |
| css1431 | 7B | 495 | 500 | 5000 | gpl (xpEHH 2.00, 6/6) | xpEHH | both | gpl | 6 | 2.002 | 2.002 |
| css1432 | 7B | 495 | 501 | 6000 | gpl2 (Fst 0.14, 7/7) | Fst | win | gpl2 | 7 | 0.136 | 0.130 |
| css1433 | 7B | 495 | 503 | 8000 | IN (Fst 0.68, 11/11) | Fst | win | IN | 11 | 0.682 | 0.564 |
| css1434 | 7B | 495 | 501 | 6000 | pnw (Fst 0.17, 7/7) | Fst | win | pnw | 7 | 0.166 | 0.164 |
| css1435 | 7B | 495 | 503 | 8000 | SRW (Fst 0.33, 11/11) | Fst | win | SRW | 11 | 0.332 | 0.308 |
| css1436 | 7B | 495 | 507 | 12000 | SRW2 (Fst 0.50, 12/12) | Fst | win | SRW2 | 12 | 0.500 | 0.409 |
| css1437 | 7B | 495 | 503 | 8000 | win2 (Fst 0.11, 11/11) | Fst | win | win2 | 11 | 0.109 | 0.089 |
| css1438 | 7B | 507 | 515 | 8000 | CA (Fst 0.44, 5/5) | Fst | both | CA | 5 | 0.438 | 0.438 |
| css1439 | 7B | 507 | 515 | 8000 | gpl (xpEHH 2.00, 5/5) | xpEHH | both | gpl | 5 | 2.003 | 2.003 |
| css1440 | 7B | 507 | 515 | 8000 | HRS2 (Fst 0.13, 5/5) | Fst | spr | HRS2 | 5 | 0.135 | 0.135 |
| css1441 | 7B | 507 | 515 | 8000 | pac (Fst 0.47, 5/5) | Fst | both | pac | 5 | 0.467 | 0.467 |
| css1442 | 7B | 507 | 515 | 8000 | TX (Rsb 2.10, 5/5) | Rsb | both | TX | 5 | 2.100 | 2.100 |
| css1443 | 7B | 507 | 515 | 8000 | TX (xpEHH 2.57, 5/5) | xpEHH | both | TX | 5 | 2.574 | 2.574 |
| css1444 | 7B | 517 | 526 | 9000 | HRS2 (xpEHH 2.33, 12/12) | xpEHH | spr | HRS2 | 12 | 2.330 | 2.153 |
| css1445 | 7B | 518 | 526 | 8000 | TX (xpEHH 2.52, 8/8) | xpEHH | both | TX | 8 | 2.524 | 2.294 |
| css1446 | 7B | 529 | 538 | 9000 | HRS2 (xpEHH 2.14, 9/9) | xpEHH | spr | HRS2 | 9 | 2.143 | 2.142 |
| css1447 | 7B | 533 | 538 | 5000 | pnw (Fst 0.25, 7/7) | Fst | win | pnw | 7 | 0.250 | 0.248 |
| css1448 | 7B | 533 | 538 | 5000 | WA (Fst 0.41, 7/7) | Fst | win | WA | 7 | 0.412 | 0.410 |
| css1449 | 7B | 536 | 542 | 6000 | gpl (Fst 0.26, 5/5) | Fst | spr | gpl | 5 | 0.261 | 0.261 |
| css1450 | 7B | 536 | 542 | 6000 | HRS2 (Fst 0.13, 5/5) | Fst | spr | HRS2 | 5 | 0.134 | 0.134 |
| css1451 | 7B | 536 | 542 | 6000 | MN (Fst 0.51, 5/5) | Fst | both | MN | 5 | 0.514 | 0.510 |
| css1452 | 7B | 536 | 543 | 7000 | TX (Rsb 2.19, 8/8) | Rsb | both | TX | 8 | 2.189 | 2.091 |
| css1453 | 7B | 543 | 548 | 5000 | gpl2 (Fst 0.17, 5/5) | Fst | both | gpl2 | 5 | 0.174 | 0.148 |
| css1454 | 7B | 543 | 553 | 10000 | TX (xpEHH 2.16, 11/11) | xpEHH | both | TX | 11 | 2.162 | 2.115 |
| css1455 | 7B | 553 | 558 | 5000 | NY (Fst 0.49, 6/6) | Fst | win | NY | 6 | 0.488 | 0.464 |
| css1456 | 7B | 553 | 558 | 5000 | SC (Fst 0.61, 6/6) | Fst | both | SC | 6 | 0.613 | 0.602 |
| css1457 | 7B | 553 | 558 | 5000 | SC (Fst 0.67, 6/6) | Fst | win | SC | 6 | 0.667 | 0.651 |
| css1458 | 7B | 557 | 580 | 23000 | TX (xpEHH 2.73, 55/55) | xpEHH | both | TX | 55 | 2.730 | 2.407 |
| css1459 | 7B | 561 | 570 | 9000 | HRS2 (Fst 0.19, 16/17) | Fst | spr | HRS2 | 16 | 0.194 | 0.185 |
| css1460 | 7B | 567 | 574 | 7000 | HRS2 (xpEHH 2.15, 21/25) | xpEHH | spr | HRS2 | 21 | 2.147 | 2.139 |
| css1461 | 7B | 584 | 595 | 11000 | gpl2 (Fst 0.21, 26/27) | Fst | both | gpl2 | 26 | 0.210 | 0.166 |
| css1462 | 7B | 584 | 597 | 13000 | nor (Rsb 3.17, 27/27) | Rsb | win | nor | 27 | 3.167 | 2.536 |
| css1463 | 7B | 584 | 592 | 8000 | TX (xpEHH 2.26, 20/20) | xpEHH | both | TX | 20 | 2.257 | 2.223 |
| css1464 | 7B | 608 | 621 | 13000 | nor (xpEHH 3.15, 20/20) | xpEHH | win | nor | 20 | 3.148 | 2.681 |
| css1465 | 7B | 611 | 621 | 10000 | nor (Rsb 3.78, 14/15) | Rsb | win | nor | 14 | 3.777 | 2.953 |
| css1466 | 7B | 612 | 617 | 5000 | HRW (Rsb 3.58, 11/12) | Rsb | win | HRW | 11 | 3.582 | 2.576 |
| css1467 | 7B | 612 | 621 | 9000 | MT (Rsb 2.87, 13/14) | Rsb | both | MT | 13 | 2.870 | 2.405 |
| css1468 | 7B | 612 | 621 | 9000 | MT (xpEHH 2.61, 13/14) | xpEHH | both | MT | 13 | 2.608 | 2.411 |
| css1469 | 7B | 615 | 621 | 6000 | ID (Rsb 2.93, 5/5) | Rsb | spr | ID | 5 | 2.928 | 2.364 |
| css1470 | 7B | 635 | 640 | 5000 | ID (Rsb 2.47, 5/5) | Rsb | spr | ID | 5 | 2.471 | 2.312 |
| css1471 | 7B | 686 | 691 | 5000 | nor (Fst 0.25, 9/10) | Fst | both | nor | 9 | 0.249 | 0.211 |
| css1472 | 7B | 692 | 700 | 8000 | pnw (Fst 0.21, 9/9) | Fst | win | pnw | 9 | 0.211 | 0.194 |
| css1473 | 7B | 710 | 716 | 6000 | pnw (Rsb 4.89, 75/81) | Rsb | win | pnw | 75 | 4.888 | 2.974 |
| css1474 | 7B | 715 | 720 | 5000 | pnw2 (Rsb 3.07, 9/10) | Rsb | spr | pnw2 | 9 | 3.071 | 2.658 |
| css1475 | 7D | 5 | 10 | 5000 | pnw (Fst 0.23, 5/5) | Fst | both | pnw | 5 | 0.226 | 0.176 |
| css1476 | 7D | 103 | 109 | 6000 | CO (Fst 0.49, 12/13) | Fst | win | CO | 12 | 0.486 | 0.303 |
| css1477 | 7D | 103 | 108 | 5000 | MN (Fst 0.31, 12/13) | Fst | spr | MN | 12 | 0.315 | 0.296 |
| css1478 | 7D | 103 | 108 | 5000 | MN (Fst 0.44, 12/13) | Fst | both | MN | 12 | 0.440 | 0.390 |
| css1479 | 7D | 104 | 109 | 5000 | TX (Fst 0.20, 5/5) | Fst | both | TX | 5 | 0.196 | 0.163 |
