# Supplementary material for: Candidate selective sweeps in US wheat populations
Source: Plant Genome. 2024 Sep 25;17(4):e20513. doi: 10.1002/tpg2.20513 (PMC11628914; doi:10.1002/tpg2.20513)
Supplement: Supplementary file 2 — Supplemental Figure S1. Comparison of standard Gaussian distribution against the observed distribution of genomewide Rsb and xpEHH values for all 63 population pairs compared. Supplemental Figure S2. Normal Q‐Q plots of observed distribution of genomewide Rsb and xpEHH values for all 63 population pairs compared. [file TPG2-17-e20513-s002.pdf]

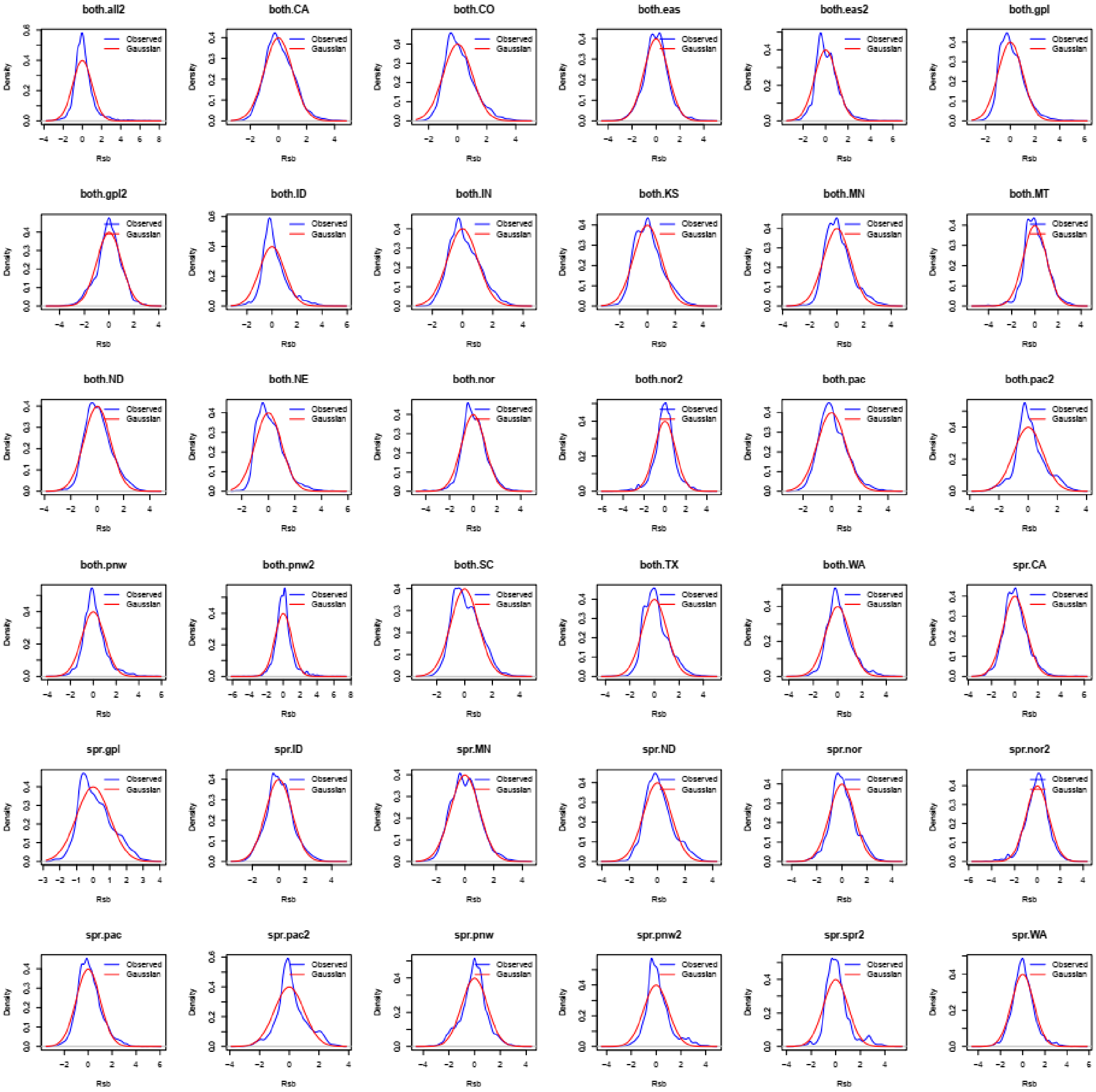
both.all2
both.CA
both.CO
both.eas
both.eas2
both.gpl
both.gpl2
both.ID
both.IN
both.KS
both.MN
both.MT
both.ND
both.NE
both.nor
both.nor2
both.pac
both.pac2
both.pnw
both.pnw2
both.SC
both.TX
both.WA
spr.CA
spr.gpl
spr.ID
spr.MN
spr.ND
spr.nor
spr.nor2
spr.pac
spr.pac2
spr.pnw
spr.pnw2
spr.spr2
spr.WA
Observed
Gaussian
Density
Rsb

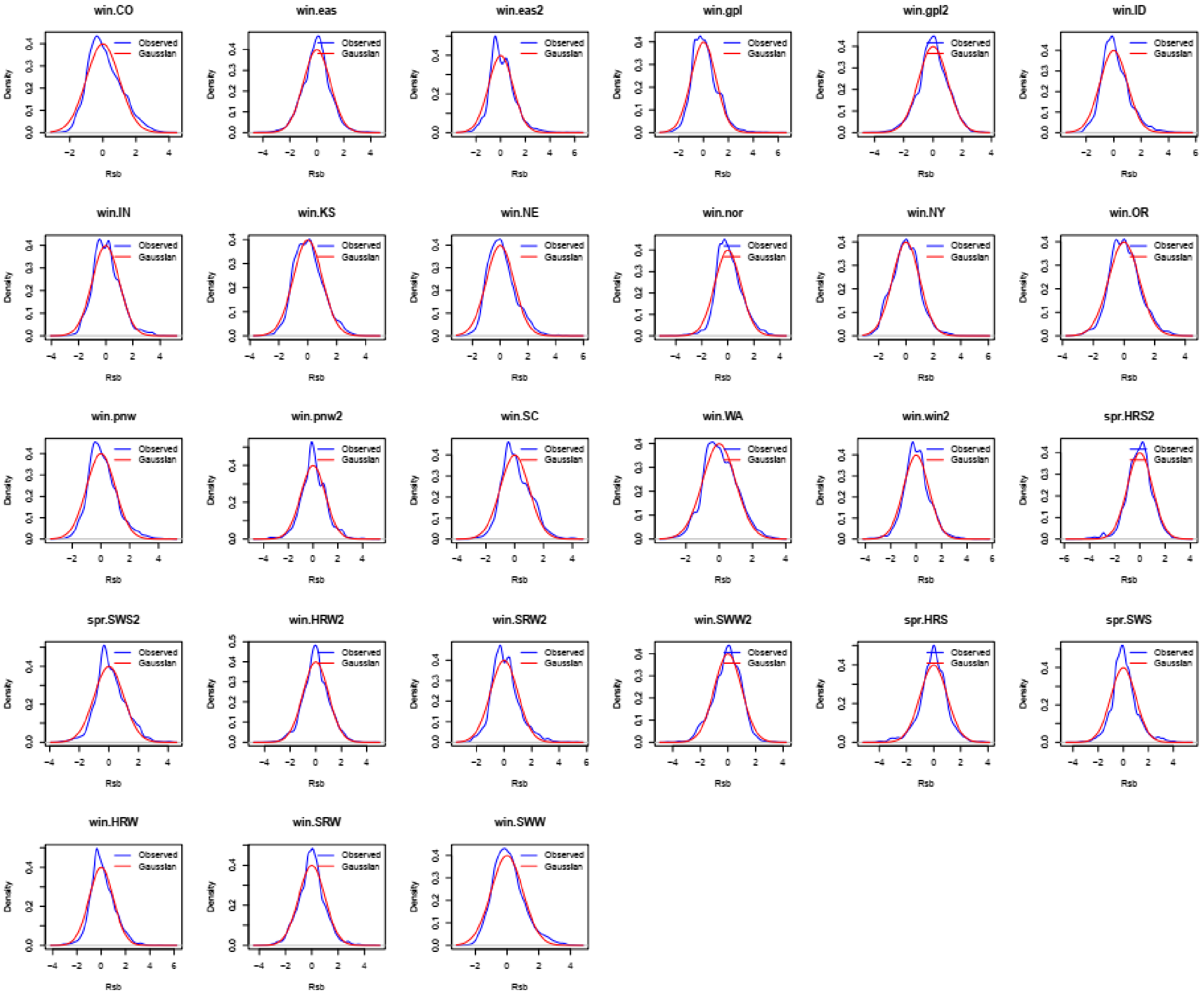
win.CO
win.eas
win.eas2
win.gpl
win.gpl2
win.ID
win.IN
win.KS
win.NE
win.nor
win.NY
win.OR
win.pnw
win.pnw2
win.SC
win.WA
win.win2
spr.HRS2
spr.SWS2
win.HRW2
win.SRW2
win.SWW2
spr.HRS
spr.SWS
win.HRW
win.SRW
win.SWW
Observed
Gaussian
Density
Rsb

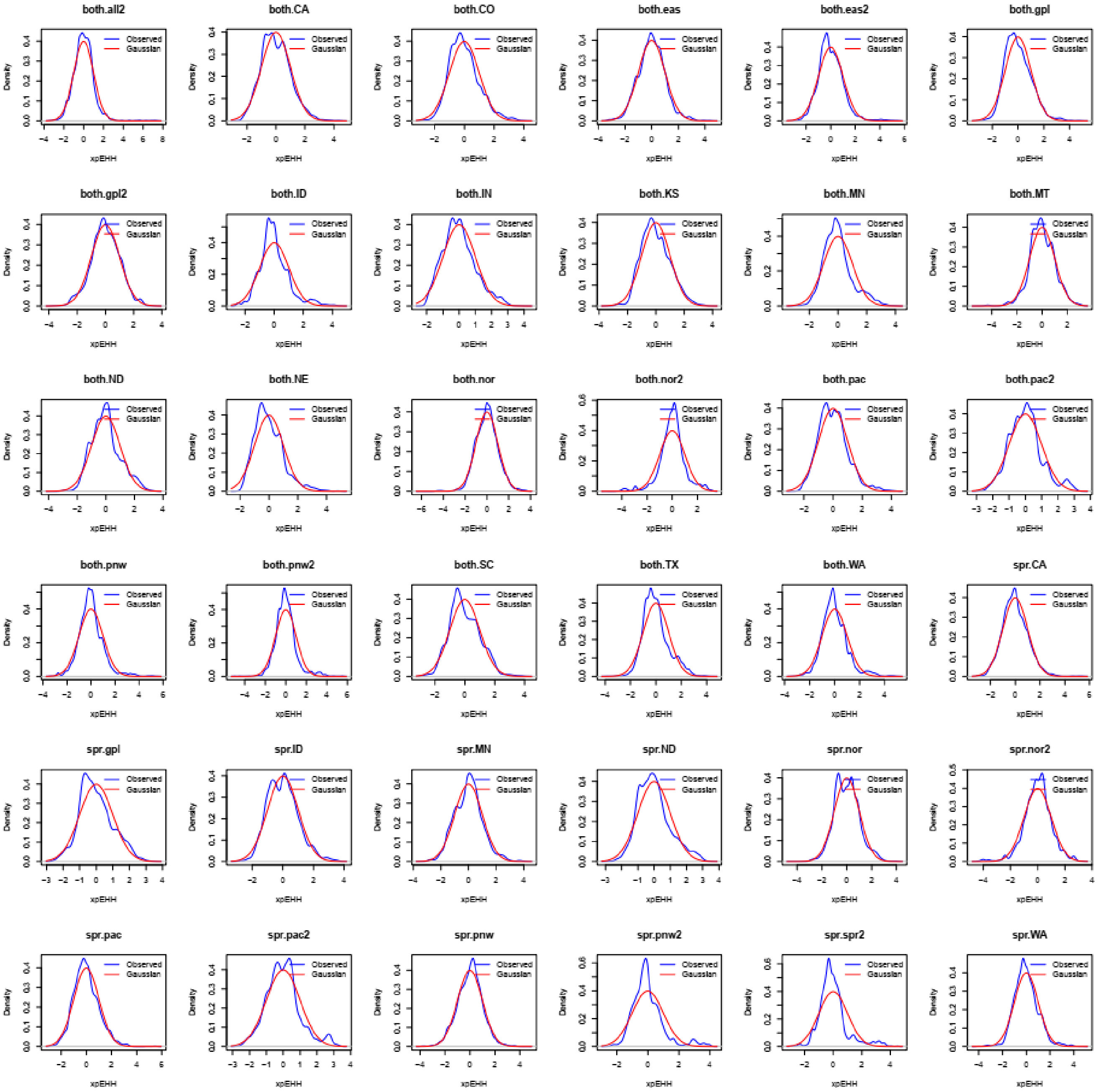
both.all2
both.CA
both.CO
both.eas
both.eas2
both.gpl
both.gpl2
both.ID
both.IN
both.KS
both.MN
both.MT
both.ND
both.NE
both.nor
both.nor2
both.pac
both.pac2
both.pnw
both.pnw2
both.SC
both.TX
both.WA
spr.CA
spr.gpl
spr.ID
spr.MN
spr.ND
spr.nor
spr.nor2
spr.pac
spr.pac2
spr.pnw
spr.pnw2
spr.spr2
spr.WA
Observed
Gaussian
Density
xpEHH

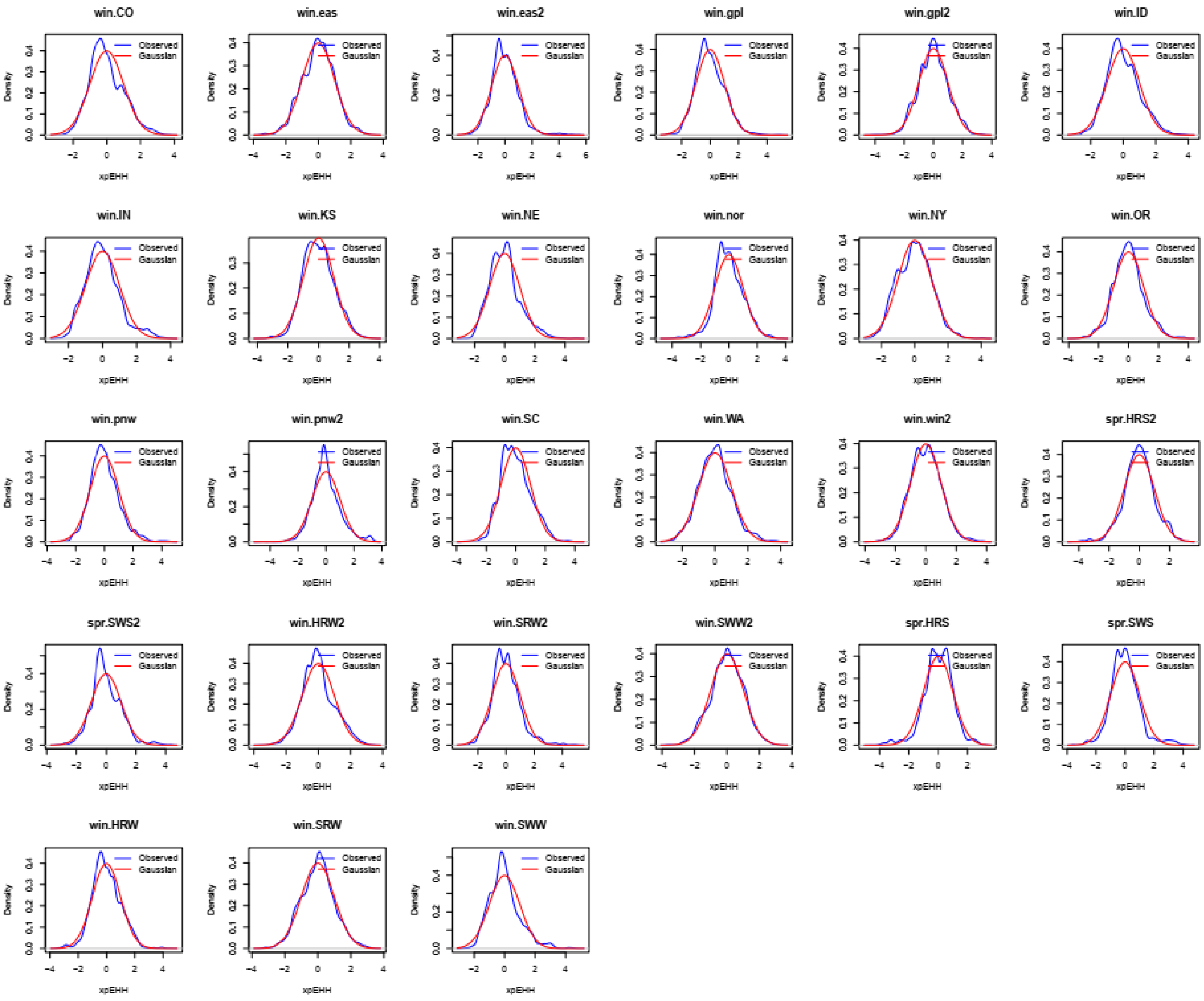

Supplemental Figure S1. Comparison of standard Gaussian distribution against the observed distribution of genomewide Rsb and xpEHH values.

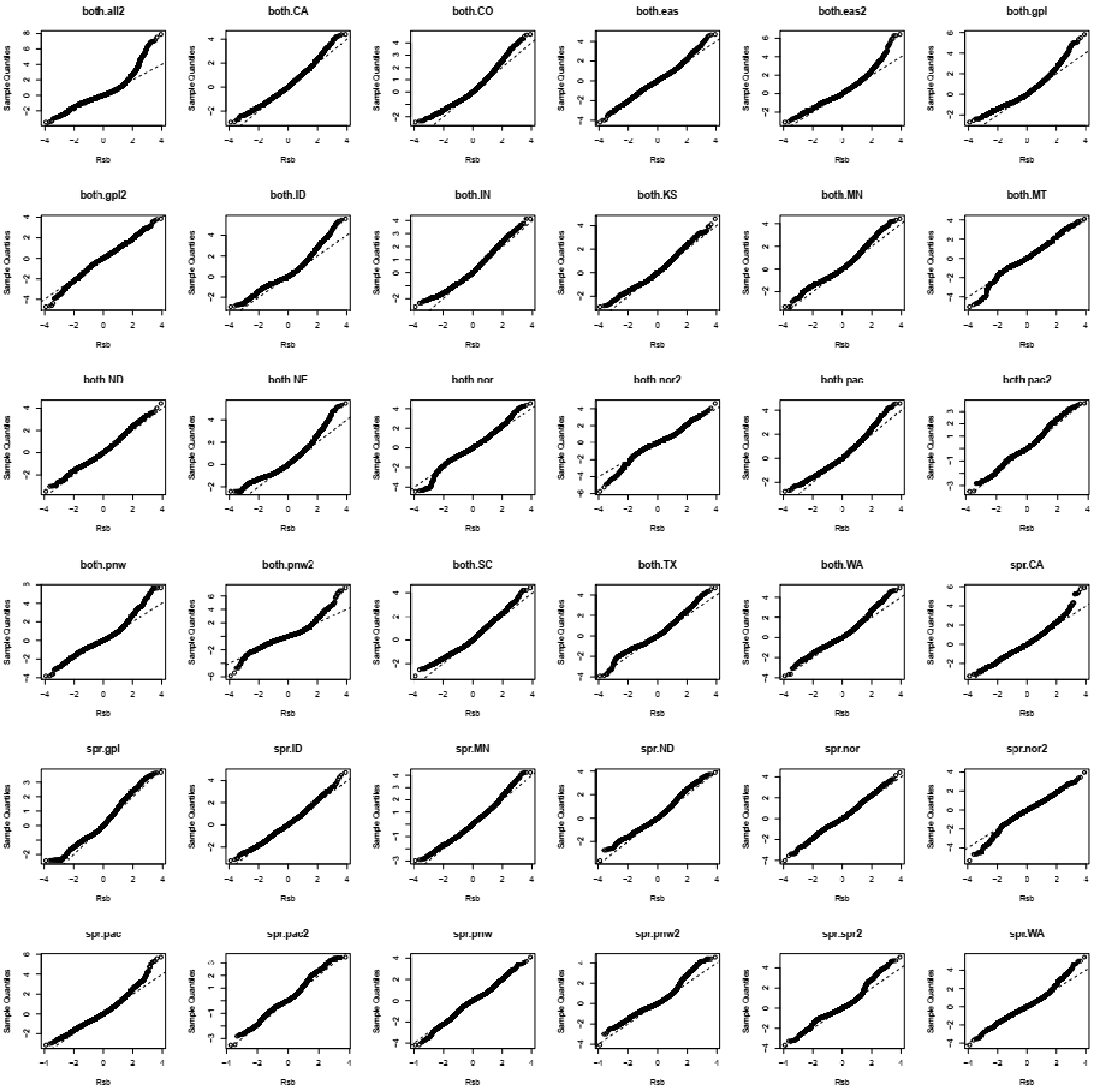
both.all2
both.CA
both.CO
both.eas
both.eas2
both.gpl
both.gpl2
both.ID
both.IN
both.KS
both.MN
both.MT
both.ND
both.NE
both.nor
both.nor2
both.pac
both.pac2
both.pnw
both.pnw2
both.SC
both.TX
both.WA
spr.CA
spr.gpl
spr.ID
spr.MN
spr.ND
spr.nor
spr.nor2
spr.pac
spr.pac2
spr.pnw
spr.pnw2
spr.spr2
spr.WA
Sample Quantiles
Rsb

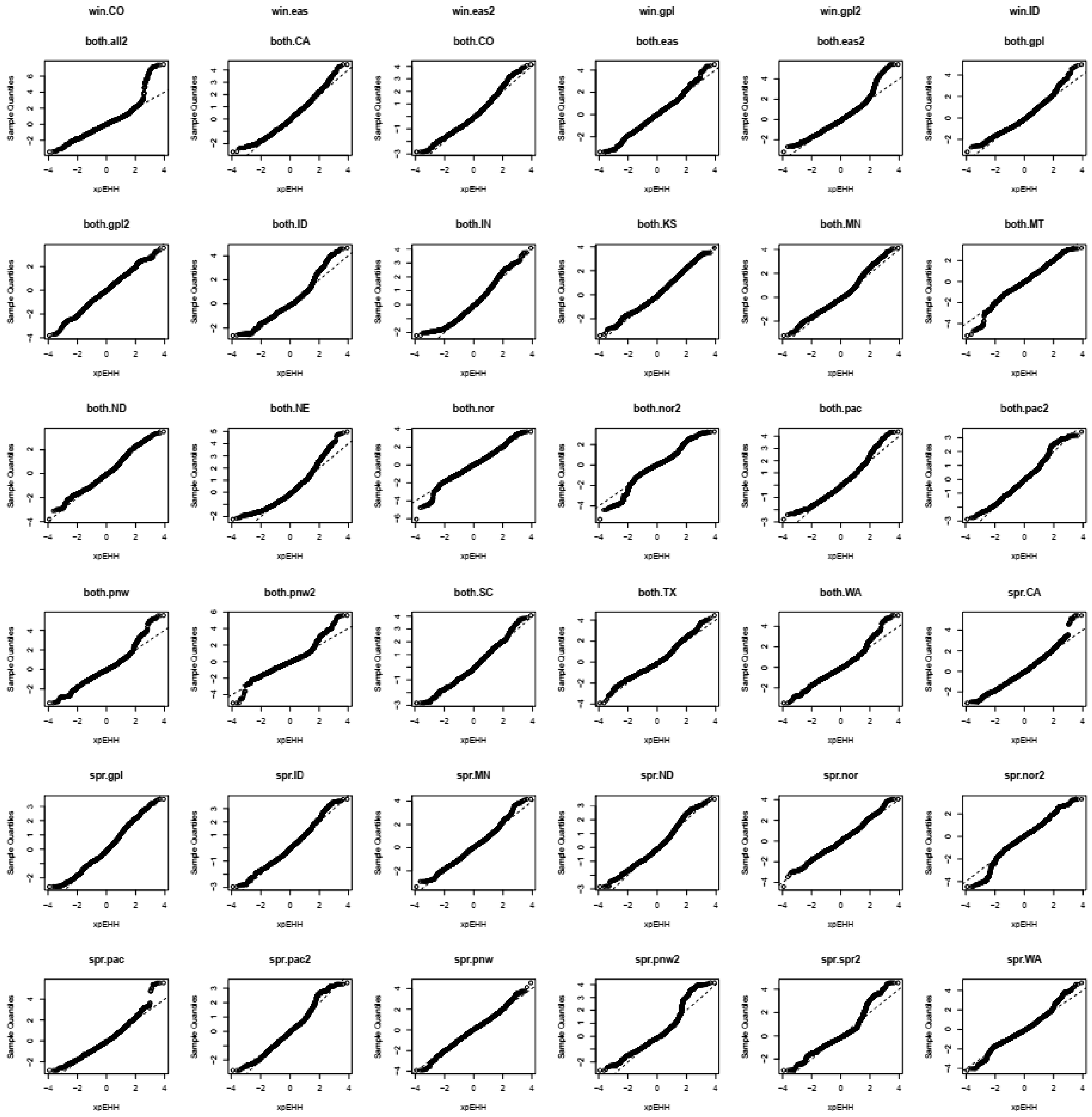
win.CO
win.eas
win.eas2
win.gpl
win.gpl2
win.ID
both.all2
both.CA
both.CO
both.eas
both.eas2
both.gpl
both.gpl2
both.ID
both.IN
both.KS
both.MN
both.MT
both.ND
both.NE
both.nor
both.nor2
both.pac
both.pac2
both.pnw
both.pnw2
both.SC
both.TX
both.WA
spr.CA
spr.gpl
spr.ID
spr.MN
spr.ND
spr.nor
spr.nor2
spr.pac
spr.pac2
spr.pnw
spr.pnw2
spr.spr2
spr.WA
Sample Quantiles
xpEHH

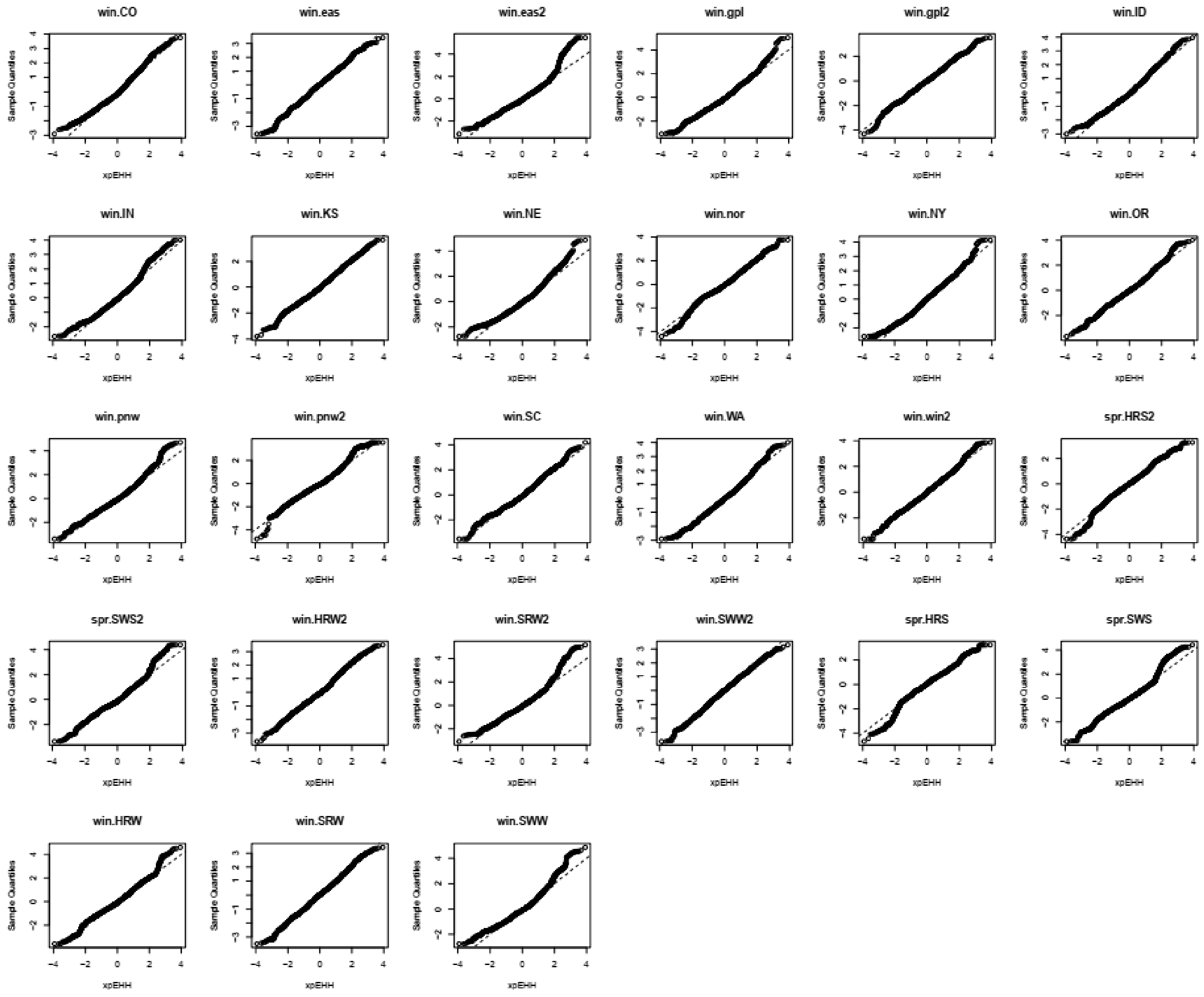

Supplemental Figure S2. Normal Q-Q plots of observed distribution of genomewide Rsb and xpEHH values for all 63 population pairs compared.
