# Supplementary material for: Candidate selective sweeps in US wheat populations
Source: Plant Genome. 2024 Sep 25;17(4):e20513. doi: 10.1002/tpg2.20513 (PMC11628914; doi:10.1002/tpg2.20513)
Supplement: Supplementary file 3 — Supplemental Figure S3. Map of candidate selective sweeps (CSS) in U.S. wheat populations due to selection over time. Each population pair was obtained by splitting a population (e.g., HRS with 150 varieties) into two halves, one of older varieties (e.g., HRS1 with 75 oldest varieties) and the other of newer varieties (e.g., HRS2 with 75 newest varieties). Fst, Rsb, and xpEHH were computed for these pairs. The linkage blocks from left to right show CSS in population pairs with a) both spring and winter varieties, b) just spring varieties, and c) just winter varieties. Physical positions in Mbp for the start of the sweep are on the left side of the bar. The right side includes end position of the CSS, name of the population selected in (spr, spring; win, winter; eas, Eastern; gpl, the Great Plains; nor, Northern, pac, the Pacific; pnw, the Pacific Northwest; HRS, hard red spring; HRW, hard red winter; SRW, soft red winter; SWS, soft white spring; SWW, soft white winter), growth habit (B, S, and W for both, spring, and winter), statistic and its maximum value, PIC values in target and reference population, major allele frequencies in the target and reference population, and CSS serial number. Red, green, and blue color of the label indicate CSS detected using Fst, Rsb, and xpEHH, respectively. Size of the label corresponds with the size of the CSS. Location of known genes are indicated by (***) and F and L refer to the physical positions of the first and last SNP genotyped on the chromosome. [file TPG2-17-e20513-s005.pdf]

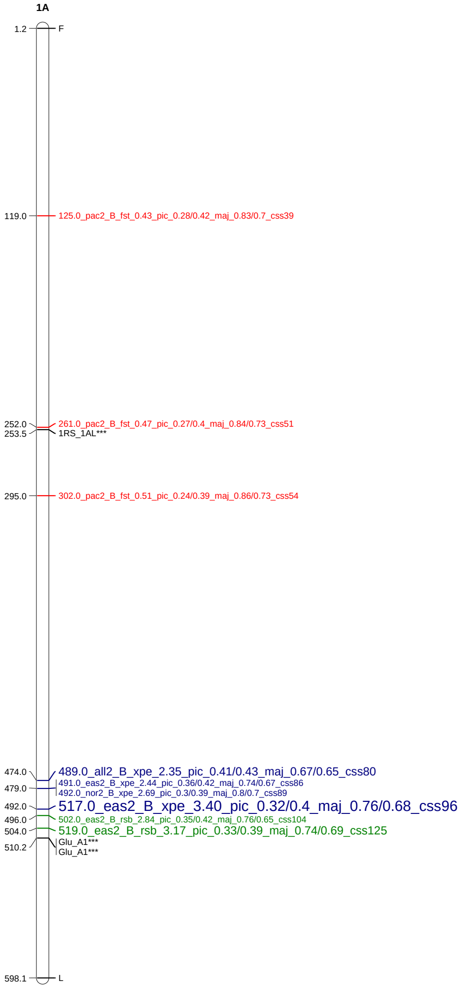
1A
1.2
F
119.0
125.0_pac2_B_fst_0.43_pic_0.28/0.42_maj_0.83/0.7_css39
252.0
261.0_pac2_B_fst_0.47_pic_0.27/0.4_maj_0.84/0.73_css51
253.5
1RS_1AL***
295.0
302.0_pac2_B_fst_0.51_pic_0.24/0.39_maj_0.86/0.73_css54
474.0
489.0_all2_B_xpe_2.35_pic_0.41/0.43_maj_0.67/0.65_css80
479.0
491.0_eas2_B_xpe_2.44_pic_0.36/0.42_maj_0.74/0.67_css86
492.0_nor2_B_xpe_2.69_pic_0.3/0.39_maj_0.8/0.7_css89
492.0
517.0_eas2_B_xpe_3.40_pic_0.32/0.4_maj_0.76/0.68_css96
496.0
502.0_eas2_B_rsb_2.84_pic_0.35/0.42_maj_0.76/0.65_css104
504.0
519.0_eas2_B_rsb_3.17_pic_0.33/0.39_maj_0.74/0.69_css125
510.2
Glu_A1***
Glu_A1***
598.1
L

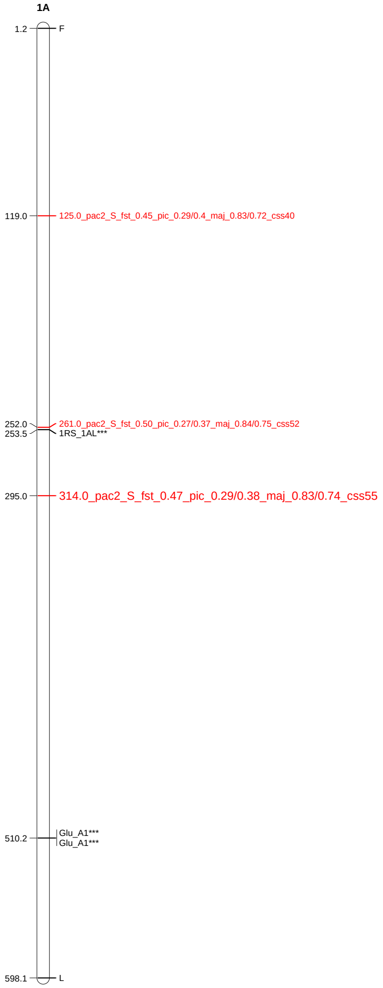
1A
1.2
F
119.0
125.0_pac2_S_fst_0.45_pic_0.29/0.4_maj_0.83/0.72_css40
252.0
261.0_pac2_S_fst_0.50_pic_0.27/0.37_maj_0.84/0.75_css52
253.5
1RS_1AL***
295.0
314.0_pac2_S_fst_0.47_pic_0.29/0.38_maj_0.83/0.74_css55
510.2
Glu_A1***
Glu_A1***
598.1
L

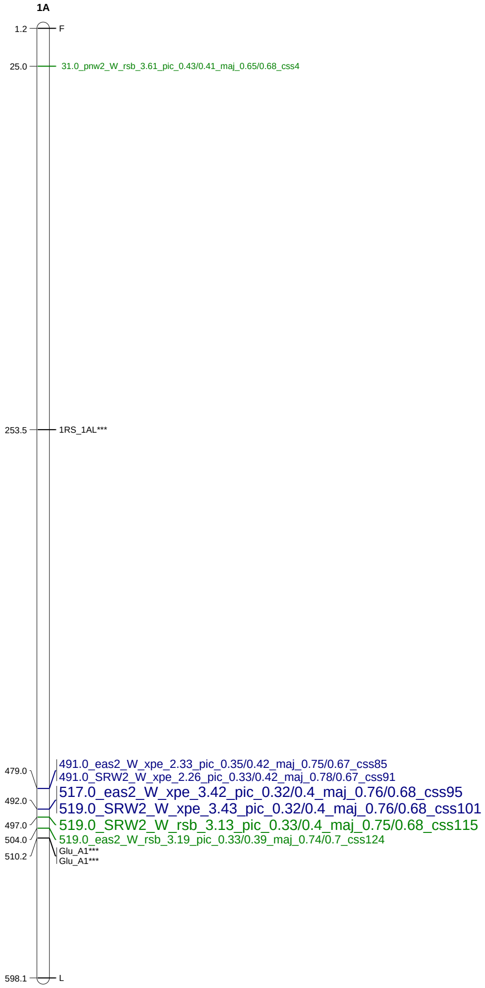
1A
1.2
F
25.0
31.0_pnw2_W_rsb_3.61_pic_0.43/0.41_maj_0.65/0.68_css4
253.5
1RS_1AL***
479.0
491.0_eas2_W_xpe_2.33_pic_0.35/0.42_maj_0.75/0.67_css85
491.0_SRW2_W_xpe_2.26_pic_0.33/0.42_maj_0.78/0.67_css91
492.0
517.0_eas2_W_xpe_3.42_pic_0.32/0.4_maj_0.76/0.68_css95
519.0_SRW2_W_xpe_3.43_pic_0.32/0.4_maj_0.76/0.68_css101
497.0
519.0_SRW2_W_rsb_3.13_pic_0.33/0.4_maj_0.75/0.68_css115
504.0
519.0_eas2_W_rsb_3.19_pic_0.33/0.39_maj_0.74/0.7_css124
510.2
Glu_A1***
Glu_A1***
598.1
L

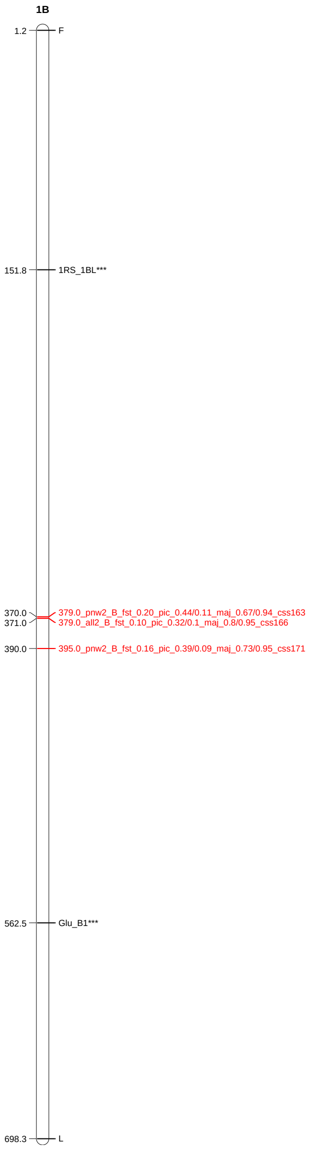

1B
1.2
F
151.8
1RS_1BL***
370.0
371.0
379.0_pnw2_B_fst_0.20_pic_0.44/0.11_maj_0.67/0.94_css163
379.0_all2_B_fst_0.10_pic_0.32/0.1_maj_0.8/0.95_css166
390.0
395.0_pnw2_B_fst_0.16_pic_0.39/0.09_maj_0.73/0.95_css171
562.5
Glu_B1***
698.3
L

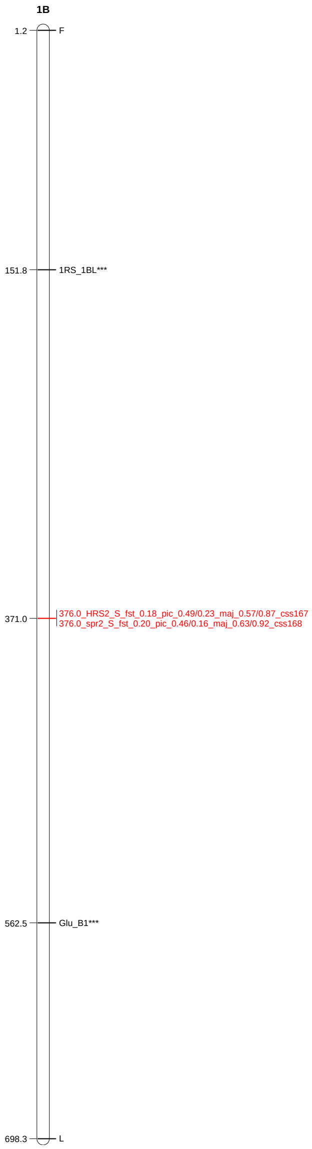

1B
1.2
F
151.8
1RS_1BL***
371.0
376.0_HRS2_S_fst_0.18_pic_0.49/0.23_maj_0.57/0.87_css167
376.0_spr2_S_fst_0.20_pic_0.46/0.16_maj_0.63/0.92_css168
562.5
Glu_B1***
698.3
L

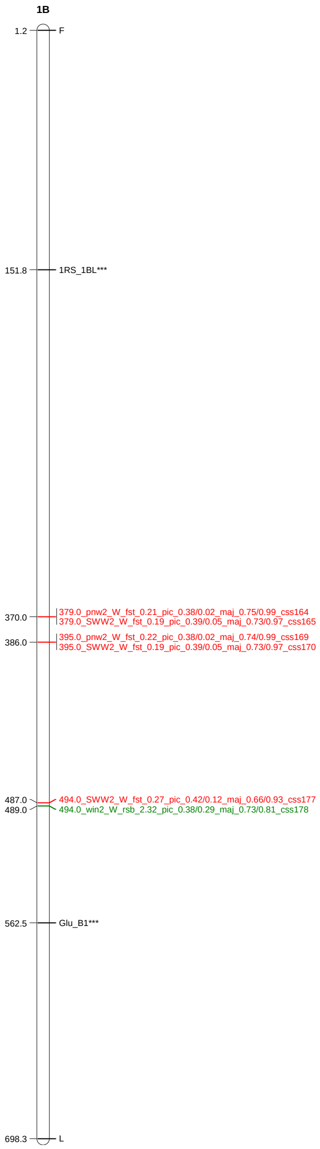

1B
1.2
F
151.8
1RS_1BL***
370.0
379.0_pnw2_W_fst_0.21_pic_0.38/0.02_maj_0.75/0.99_css164
379.0_SWW2_W_fst_0.19_pic_0.39/0.05_maj_0.73/0.97_css165
386.0
395.0_pnw2_W_fst_0.22_pic_0.38/0.02_maj_0.74/0.99_css169
395.0_SWW2_W_fst_0.19_pic_0.39/0.05_maj_0.73/0.97_css170
487.0
489.0
494.0_SWW2_W_fst_0.27_pic_0.42/0.12_maj_0.66/0.93_css177
494.0_win2_W_rsb_2.32_pic_0.38/0.29_maj_0.73/0.81_css178
562.5
Glu_B1***
698.3
L

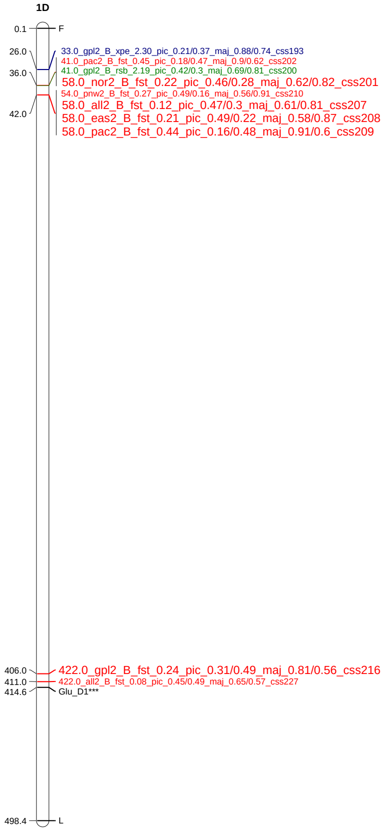
1D
0.1
F
26.0
36.0
42.0
33.0_gpl2_B_xpe_2.30_pic_0.21/0.37_maj_0.88/0.74_css193
41.0_pac2_B_fst_0.45_pic_0.18/0.47_maj_0.9/0.62_css202
41.0_gpl2_B_rsb_2.19_pic_0.42/0.3_maj_0.69/0.81_css200
58.0_nor2_B_fst_0.22_pic_0.46/0.28_maj_0.62/0.82_css201
54.0_pnw2_B_fst_0.27_pic_0.49/0.16_maj_0.56/0.91_css210
58.0_all2_B_fst_0.12_pic_0.47/0.3_maj_0.61/0.81_css207
58.0_eas2_B_fst_0.21_pic_0.49/0.22_maj_0.58/0.87_css208
58.0_pac2_B_fst_0.44_pic_0.16/0.48_maj_0.91/0.6_css209
406.0
411.0
414.6
422.0_gpl2_B_fst_0.24_pic_0.31/0.49_maj_0.81/0.56_css216
422.0_all2_B_fst_0.08_pic_0.45/0.49_maj_0.65/0.57_css227
Glu_D1***
498.4
L

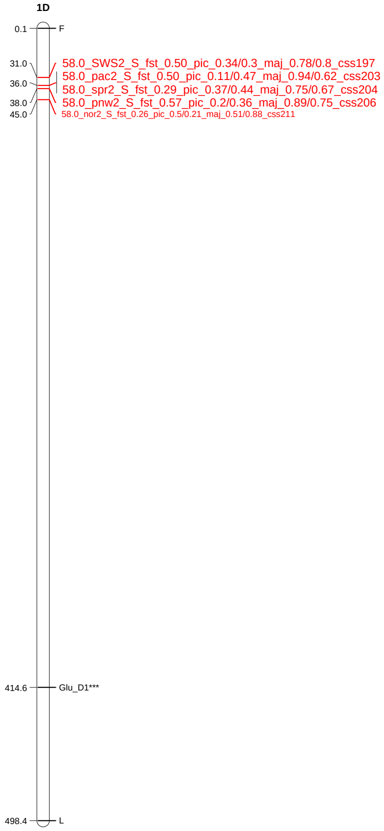
1D
0.1
F
31.0
36.0
38.0
45.0
58.0_SWS2_S_fst_0.50_pic_0.34/0.3_maj_0.78/0.8_css197
58.0_pac2_S_fst_0.50_pic_0.11/0.47_maj_0.94/0.62_css203
58.0_spr2_S_fst_0.29_pic_0.37/0.44_maj_0.75/0.67_css204
58.0_pnw2_S_fst_0.57_pic_0.2/0.36_maj_0.89/0.75_css206
58.0_nor2_S_fst_0.26_pic_0.5/0.21_maj_0.51/0.88_css211
414.6
Glu_D1***
498.4
L

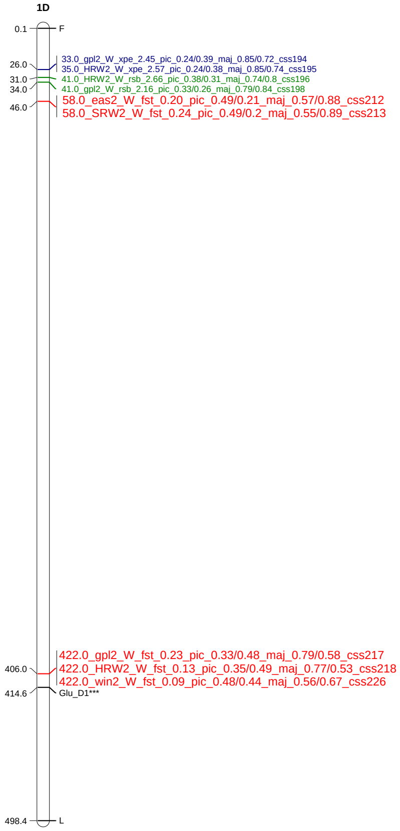
1D
0.1
F
26.0
31.0
34.0
46.0
33.0_gpl2_W_xpe_2.45_pic_0.24/0.39_maj_0.85/0.72_css194
35.0_HRW2_W_xpe_2.57_pic_0.24/0.38_maj_0.85/0.74_css195
41.0_HRW2_W_rsb_2.66_pic_0.38/0.31_maj_0.74/0.8_css196
41.0_gpl2_W_rsb_2.16_pic_0.33/0.26_maj_0.79/0.84_css198
58.0_eas2_W_fst_0.20_pic_0.49/0.21_maj_0.57/0.88_css212
58.0_SRW2_W_fst_0.24_pic_0.49/0.2_maj_0.55/0.89_css213
406.0
414.6
422.0_gpl2_W_fst_0.23_pic_0.33/0.48_maj_0.79/0.58_css217
422.0_HRW2_W_fst_0.13_pic_0.35/0.49_maj_0.77/0.53_css218
422.0_win2_W_fst_0.09_pic_0.48/0.44_maj_0.56/0.67_css226
Glu_D1***
498.4
L

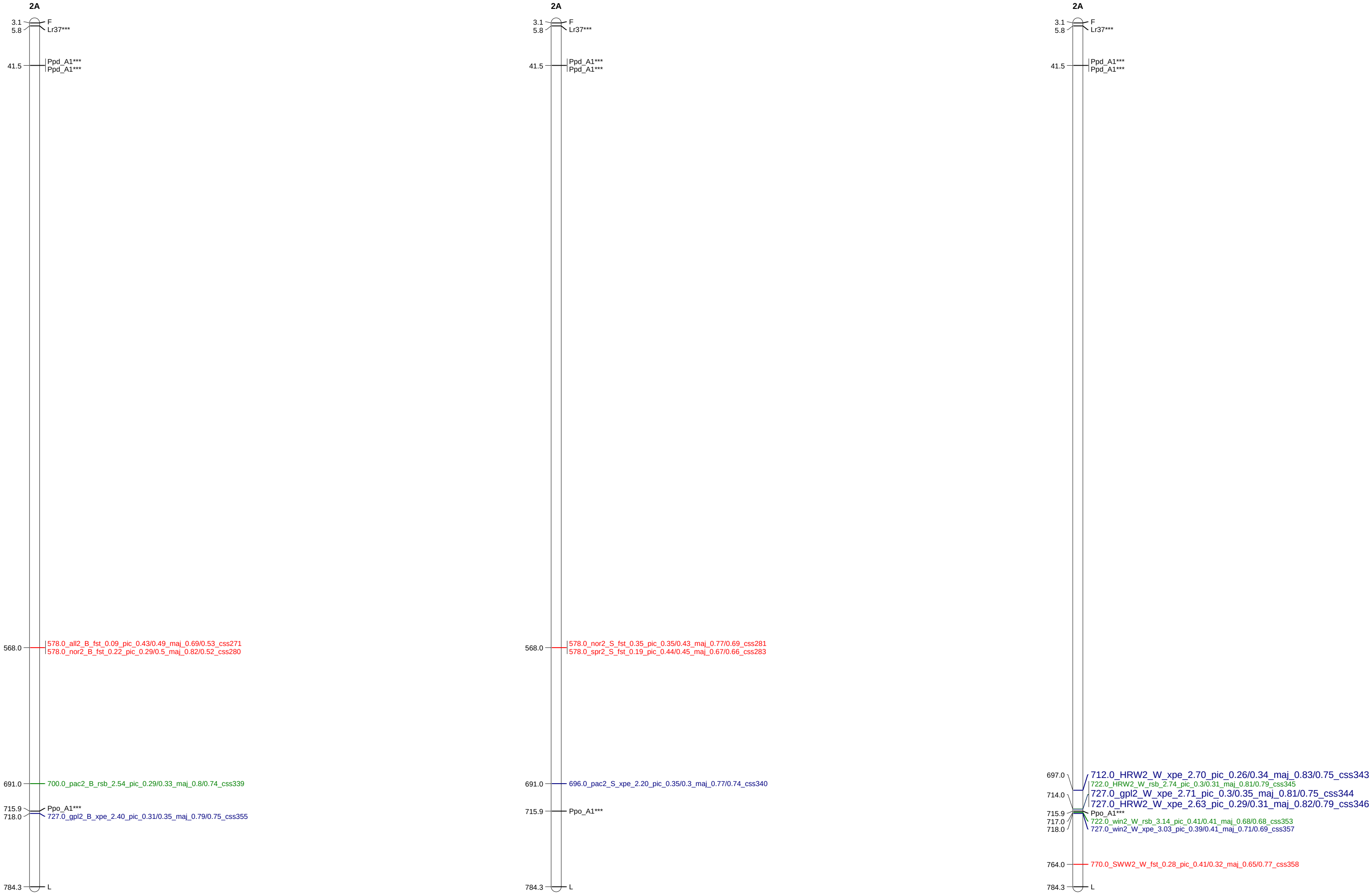
2A
3.1
F
5.8
Lr37***
41.5
Ppd_A1***
Ppd_A1***
568.0
578.0_all2_B_fst_0.09_pic_0.43/0.49_maj_0.69/0.53_css271
578.0_nor2_B_fst_0.22_pic_0.29/0.5_maj_0.82/0.52_css280
691.0
700.0_pac2_B_rsb_2.54_pic_0.29/0.33_maj_0.8/0.74_css339
715.9
Ppo_A1***
718.0
727.0_gpl2_B_xpe_2.40_pic_0.31/0.35_maj_0.79/0.75_css355
784.3
L
2A
3.1
F
5.8
Lr37***
41.5
Ppd_A1***
Ppd_A1***
568.0
578.0_nor2_S_fst_0.35_pic_0.35/0.43_maj_0.77/0.69_css281
578.0_spr2_S_fst_0.19_pic_0.44/0.45_maj_0.67/0.66_css283
691.0
696.0_pac2_S_xpe_2.20_pic_0.35/0.3_maj_0.77/0.74_css340
715.9
Ppo_A1***
784.3
L
2A
3.1
F
5.8
Lr37***
41.5
Ppd_A1***
Ppd_A1***
697.0
712.0_HRW2_W_xpe_2.70_pic_0.26/0.34_maj_0.83/0.75_css343
722.0_HRW2_W_rsb_2.74_pic_0.3/0.31_maj_0.81/0.79_css345
714.0
727.0_gpl2_W_xpe_2.71_pic_0.3/0.35_maj_0.81/0.75_css344
727.0_HRW2_W_xpe_2.63_pic_0.29/0.31_maj_0.82/0.79_css346
715.9
Ppo_A1***
717.0
722.0_win2_W_rsb_3.14_pic_0.41/0.41_maj_0.68/0.68_css353
718.0
727.0_win2_W_xpe_3.03_pic_0.39/0.41_maj_0.71/0.69_css357
764.0
770.0_SWW2_W_fst_0.28_pic_0.41/0.32_maj_0.65/0.77_css358
784.3
L

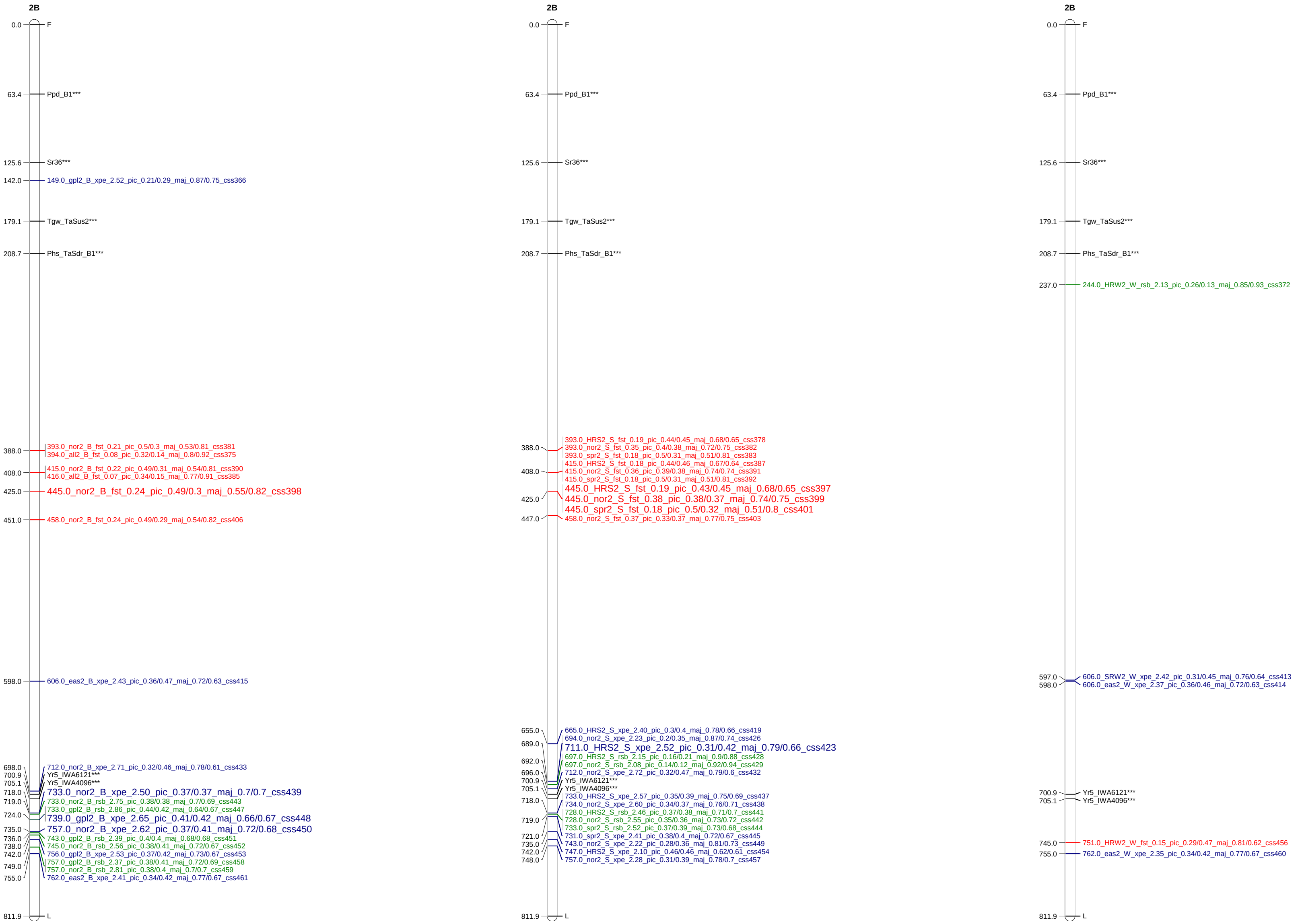

2B
0.0
63.4
125.6
142.0
179.1
208.7
388.0
408.0
425.0
451.0
598.0
698.0
700.9
705.1
718.0
719.0
724.0
735.0
736.0
738.0
742.0
749.0
755.0
811.9
F
Ppd_B1***
Sr36***
149.0_gpl2_B_xpe_2.52_pic_0.21/0.29_maj_0.87/0.75_css366
Tgw_TaSus2***
Phs_TaSdr_B1***
393.0_nor2_B_fst_0.21_pic_0.5/0.3_maj_0.53/0.81_css381
394.0_all2_B_fst_0.08_pic_0.32/0.14_maj_0.8/0.92_css375
415.0_nor2_B_fst_0.22_pic_0.49/0.31_maj_0.54/0.81_css390
416.0_all2_B_fst_0.07_pic_0.34/0.15_maj_0.77/0.91_css385
445.0_nor2_B_fst_0.24_pic_0.49/0.3_maj_0.55/0.82_css398
458.0_nor2_B_fst_0.24_pic_0.49/0.29_maj_0.54/0.82_css406
606.0_eas2_B_xpe_2.43_pic_0.36/0.47_maj_0.72/0.63_css415
712.0_nor2_B_xpe_2.71_pic_0.32/0.46_maj_0.78/0.61_css433
Yr5_IWA6121***
Yr5_IWA4096***
733.0_nor2_B_xpe_2.50_pic_0.37/0.37_maj_0.7/0.7_css439
733.0_nor2_B_rsb_2.75_pic_0.38/0.38_maj_0.7/0.69_css443
733.0_gpl2_B_rsb_2.86_pic_0.44/0.42_maj_0.64/0.67_css447
739.0_gpl2_B_xpe_2.65_pic_0.41/0.42_maj_0.66/0.67_css448
757.0_nor2_B_xpe_2.62_pic_0.37/0.41_maj_0.72/0.68_css450
743.0_gpl2_B_rsb_2.39_pic_0.4/0.4_maj_0.68/0.68_css451
745.0_nor2_B_rsb_2.56_pic_0.38/0.41_maj_0.72/0.67_css452
756.0_gpl2_B_xpe_2.53_pic_0.37/0.42_maj_0.73/0.67_css453
757.0_gpl2_B_rsb_2.37_pic_0.38/0.41_maj_0.72/0.69_css458
757.0_nor2_B_rsb_2.81_pic_0.38/0.4_maj_0.7/0.7_css459
762.0_eas2_B_xpe_2.41_pic_0.34/0.42_maj_0.77/0.67_css461
L
2B
0.0
63.4
125.6
179.1
208.7
388.0
408.0
425.0
447.0
655.0
689.0
692.0
696.0
700.9
705.1
718.0
719.0
721.0
735.0
742.0
748.0
811.9
F
Ppd_B1***
Sr36***
Tgw_TaSus2***
Phs_TaSdr_B1***
393.0_HRS2_S_fst_0.19_pic_0.44/0.45_maj_0.68/0.65_css378
393.0_nor2_S_fst_0.35_pic_0.4/0.38_maj_0.72/0.75_css382
393.0_spr2_S_fst_0.18_pic_0.5/0.31_maj_0.51/0.81_css383
415.0_HRS2_S_fst_0.18_pic_0.44/0.46_maj_0.67/0.64_css387
415.0_nor2_S_fst_0.36_pic_0.39/0.38_maj_0.74/0.74_css391
415.0_spr2_S_fst_0.18_pic_0.5/0.31_maj_0.51/0.81_css392
445.0_HRS2_S_fst_0.19_pic_0.43/0.45_maj_0.68/0.65_css397
445.0_nor2_S_fst_0.38_pic_0.38/0.37_maj_0.74/0.75_css399
445.0_spr2_S_fst_0.18_pic_0.5/0.32_maj_0.51/0.8_css401
458.0_nor2_S_fst_0.37_pic_0.33/0.37_maj_0.77/0.75_css403
665.0_HRS2_S_xpe_2.40_pic_0.3/0.4_maj_0.78/0.66_css419
694.0_nor2_S_xpe_2.23_pic_0.2/0.35_maj_0.87/0.74_css426
711.0_HRS2_S_xpe_2.52_pic_0.31/0.42_maj_0.79/0.66_css423
697.0_HRS2_S_rsb_2.15_pic_0.16/0.21_maj_0.9/0.88_css428
697.0_nor2_S_rsb_2.08_pic_0.14/0.12_maj_0.92/0.94_css429
712.0_nor2_S_xpe_2.72_pic_0.32/0.47_maj_0.79/0.6_css432
Yr5_IWA6121***
Yr5_IWA4096***
733.0_HRS2_S_xpe_2.57_pic_0.35/0.39_maj_0.75/0.69_css437
734.0_nor2_S_xpe_2.60_pic_0.34/0.37_maj_0.76/0.71_css438
728.0_HRS2_S_rsb_2.46_pic_0.37/0.38_maj_0.71/0.7_css441
728.0_nor2_S_rsb_2.55_pic_0.35/0.36_maj_0.73/0.72_css442
733.0_spr2_S_rsb_2.52_pic_0.37/0.39_maj_0.73/0.68_css444
731.0_spr2_S_xpe_2.41_pic_0.38/0.4_maj_0.72/0.67_css445
743.0_nor2_S_xpe_2.22_pic_0.28/0.36_maj_0.81/0.73_css449
747.0_HRS2_S_xpe_2.10_pic_0.46/0.46_maj_0.62/0.61_css454
757.0_nor2_S_xpe_2.28_pic_0.31/0.39_maj_0.78/0.7_css457
L
2B
0.0
63.4
125.6
179.1
208.7
237.0
597.0
598.0
700.9
705.1
745.0
755.0
811.9
F
Ppd_B1***
Sr36***
Tgw_TaSus2***
Phs_TaSdr_B1***
244.0_HRW2_W_rsb_2.13_pic_0.26/0.13_maj_0.85/0.93_css372
606.0_SRW2_W_xpe_2.42_pic_0.31/0.45_maj_0.76/0.64_css413
606.0_eas2_W_xpe_2.37_pic_0.36/0.46_maj_0.72/0.63_css414
Yr5_IWA6121***
Yr5_IWA4096***
751.0_HRW2_W_fst_0.15_pic_0.29/0.47_maj_0.81/0.62_css456
762.0_eas2_W_xpe_2.35_pic_0.34/0.42_maj_0.77/0.67_css460
L

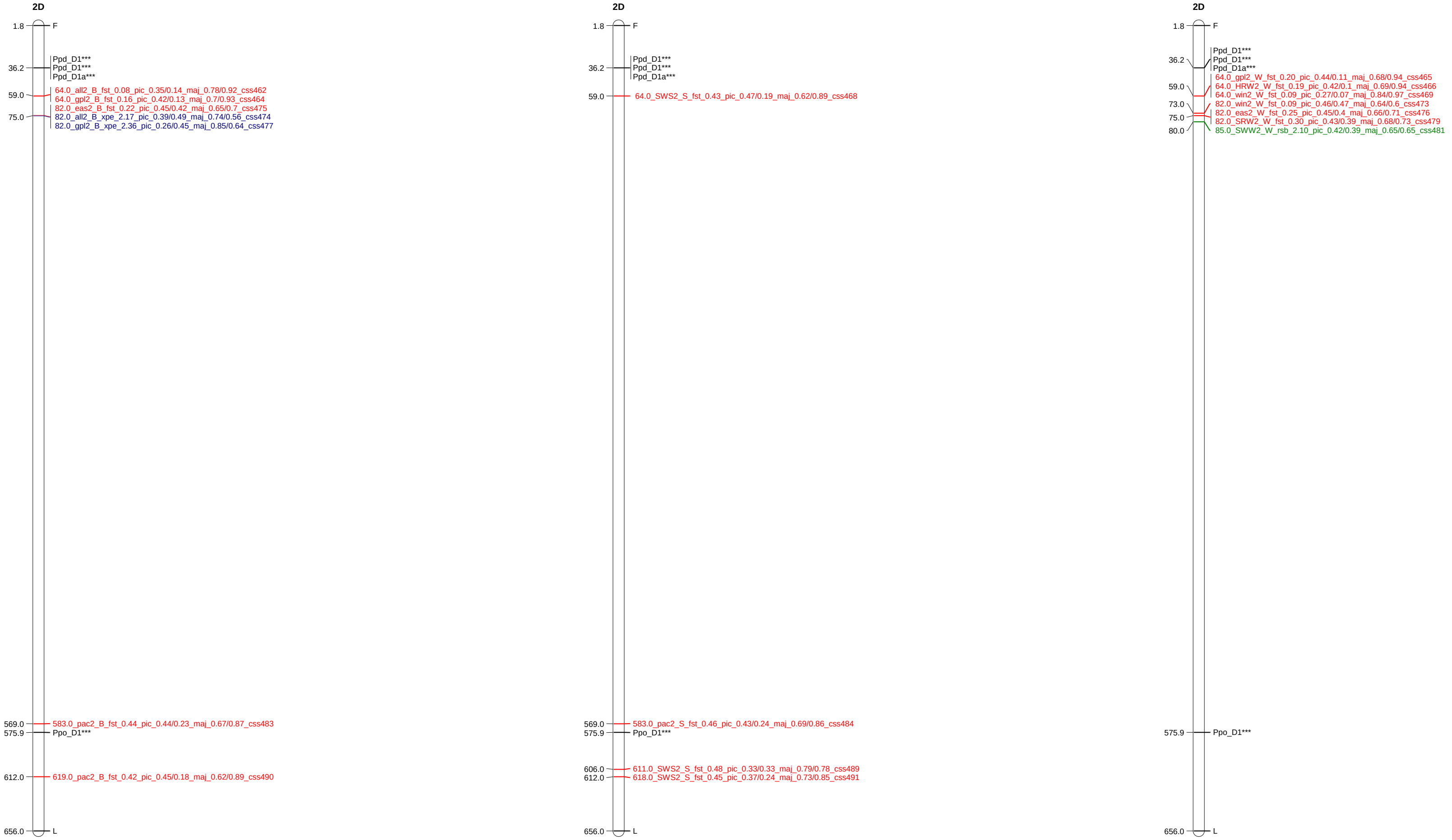

2D
1.8
F
36.2
Ppd_D1***
Ppd_D1***
Ppd_D1a***
59.0
64.0_all2_B_fst_0.08_pic_0.35/0.14_maj_0.78/0.92_css462
64.0_gpl2_B_fst_0.16_pic_0.42/0.13_maj_0.7/0.93_css464
75.0
82.0_eas2_B_fst_0.22_pic_0.45/0.42_maj_0.65/0.7_css475
82.0_all2_B_xpe_2.17_pic_0.39/0.49_maj_0.74/0.56_css474
82.0_gpl2_B_xpe_2.36_pic_0.26/0.45_maj_0.85/0.64_css477
569.0
583.0_pac2_B_fst_0.44_pic_0.44/0.23_maj_0.67/0.87_css483
575.9
Ppo_D1***
612.0
619.0_pac2_B_fst_0.42_pic_0.45/0.18_maj_0.62/0.89_css490
656.0
L
2D
1.8
F
36.2
Ppd_D1***
Ppd_D1***
Ppd_D1a***
59.0
64.0_SWS2_S_fst_0.43_pic_0.47/0.19_maj_0.62/0.89_css468
569.0
583.0_pac2_S_fst_0.46_pic_0.43/0.24_maj_0.69/0.86_css484
575.9
Ppo_D1***
606.0
611.0_SWS2_S_fst_0.48_pic_0.33/0.33_maj_0.79/0.78_css489
612.0
618.0_SWS2_S_fst_0.45_pic_0.37/0.24_maj_0.73/0.85_css491
656.0
L
2D
1.8
F
36.2
Ppd_D1***
Ppd_D1***
Ppd_D1a***
59.0
64.0_gpl2_W_fst_0.20_pic_0.44/0.11_maj_0.68/0.94_css465
64.0_HRW2_W_fst_0.19_pic_0.42/0.1_maj_0.69/0.94_css466
64.0_win2_W_fst_0.09_pic_0.27/0.07_maj_0.84/0.97_css469
73.0
82.0_win2_W_fst_0.09_pic_0.46/0.47_maj_0.64/0.6_css473
75.0
82.0_eas2_W_fst_0.25_pic_0.45/0.4_maj_0.66/0.71_css476
82.0_SRW2_W_fst_0.30_pic_0.43/0.39_maj_0.68/0.73_css479
80.0
85.0_SWW2_W_rsb_2.10_pic_0.42/0.39_maj_0.65/0.65_css481
575.9
Ppo_D1***
656.0
L

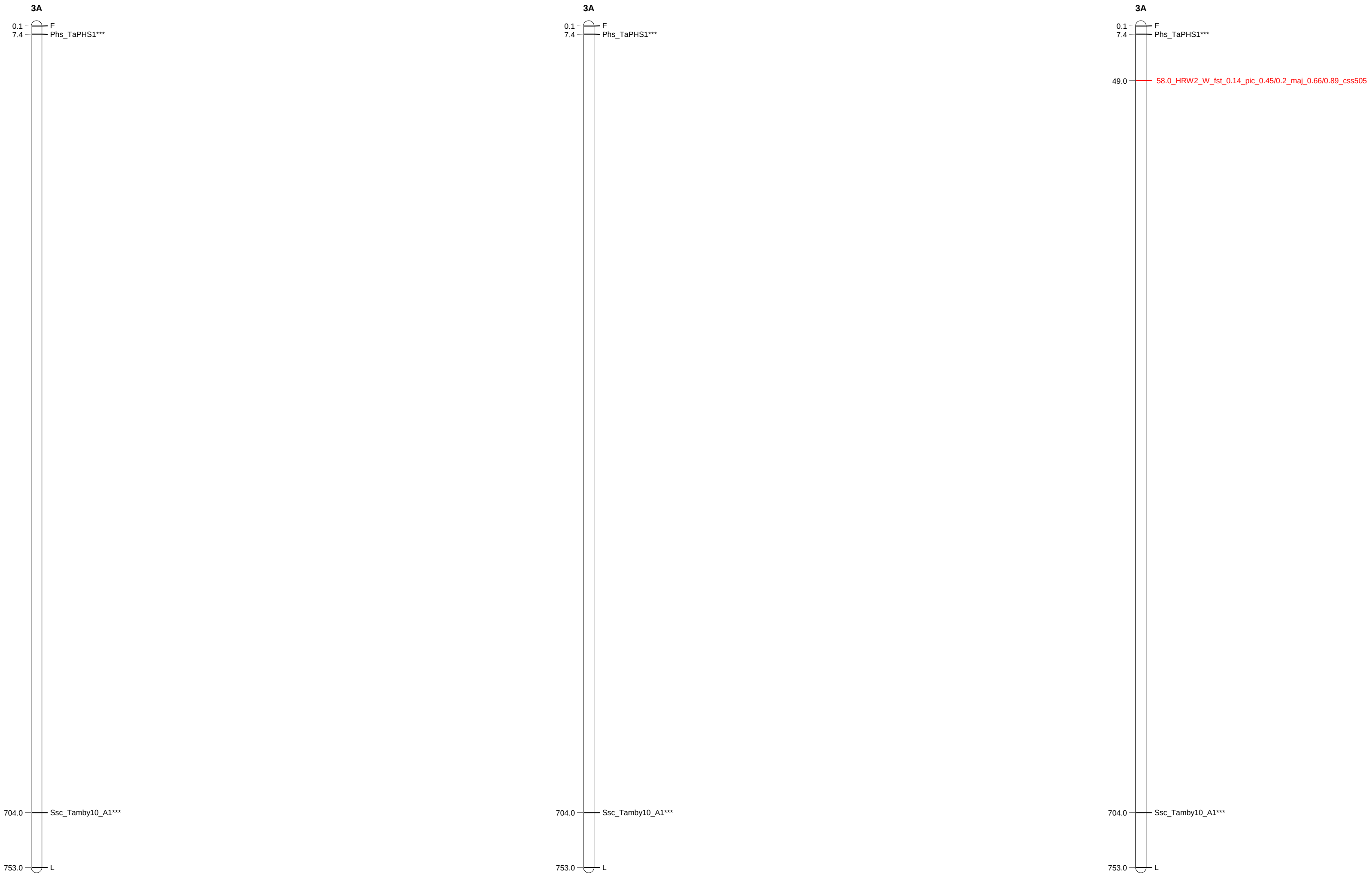
3A
0.1 F
7.4 Phs_TaPHS1***
704.0 Ssc_Tamby10_A1***
753.0 L
3A
0.1 F
7.4 Phs_TaPHS1***
704.0 Ssc_Tamby10_A1***
753.0 L
3A
0.1 F
7.4 Phs_TaPHS1***
49.0 58.0_HRW2_W_fst_0.14_pic_0.45/0.2_maj_0.66/0.89_css505
704.0 Ssc_Tamby10_A1***
753.0 L

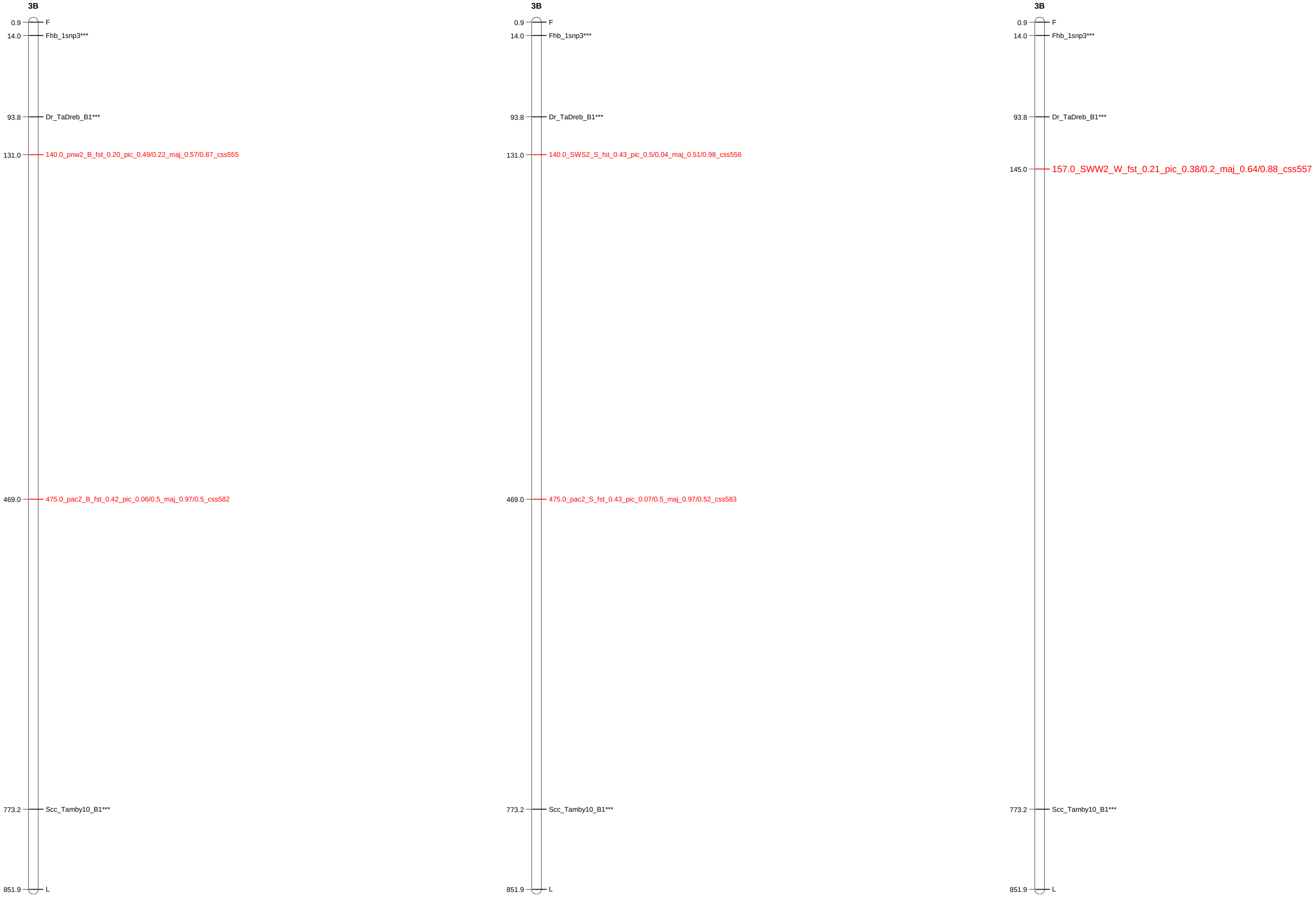
3B
0.9 F
14.0 Fhb_1snp3***
93.8 Dr_TaDreb_B1***
131.0 140.0_pnw2_B_fst_0.20_pic_0.49/0.22_maj_0.57/0.87_css555
469.0 475.0_pac2_B_fst_0.42_pic_0.06/0.5_maj_0.97/0.5_css582
773.2 Scc_Tamby10_B1***
851.9 L
3B
0.9 F
14.0 Fhb_1snp3***
93.8 Dr_TaDreb_B1***
131.0 140.0_SWS2_S_fst_0.43_pic_0.5/0.04_maj_0.51/0.98_css556
469.0 475.0_pac2_S_fst_0.43_pic_0.07/0.5_maj_0.97/0.52_css583
773.2 Scc_Tamby10_B1***
851.9 L
3B
0.9 F
14.0 Fhb_1snp3***
93.8 Dr_TaDreb_B1***
145.0 157.0_SWW2_W_fst_0.21_pic_0.38/0.2_maj_0.64/0.88_css557
773.2 Scc_Tamby10_B1***
851.9 L

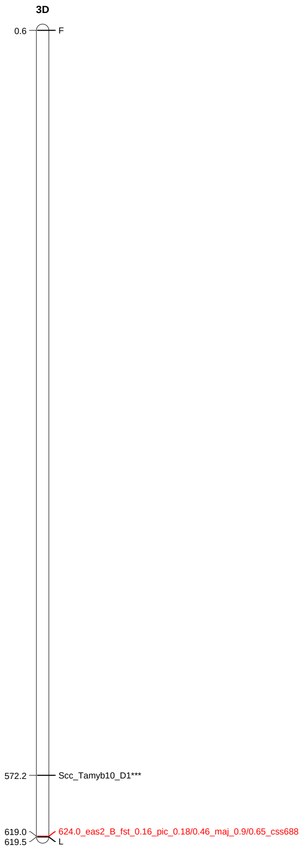
3D
0.6
F
572.2
Scc_Tamyb10_D1***
619.0
619.5
624.0_eas2_B_fst_0.16_pic_0.18/0.46_maj_0.9/0.65_css688
L

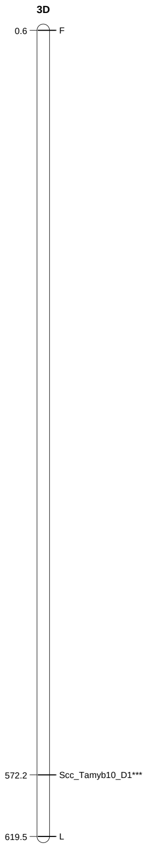
3D
0.6
F
572.2
Scc_Tamyb10_D1***
619.5
L

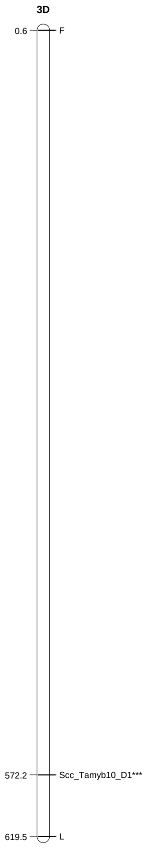
3D
0.6
F
572.2
Scc_Tamyb10_D1***
619.5
L

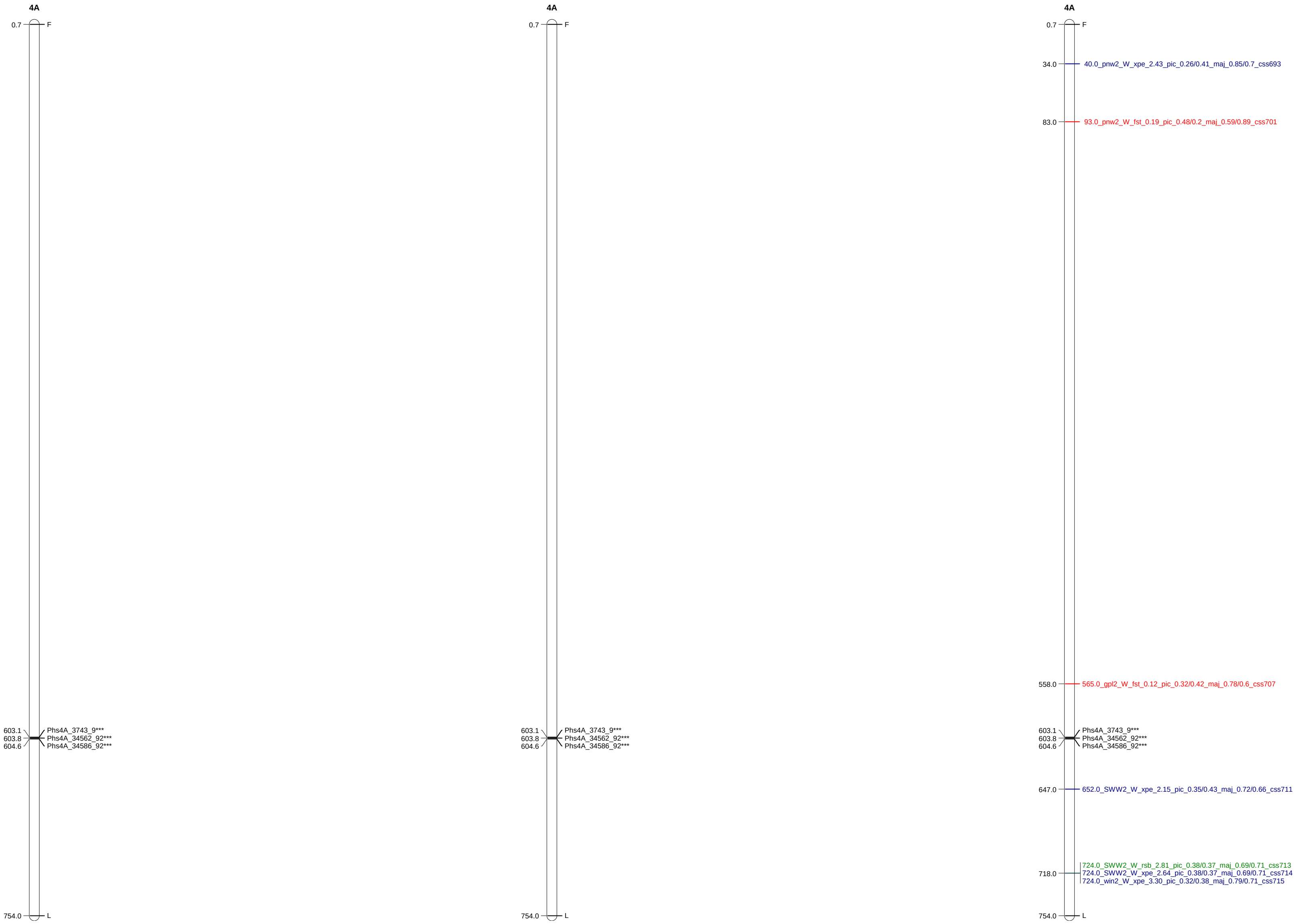
4A
0.7
F
603.1
603.8
604.6
Phs4A_3743_9***
Phs4A_34562_92***
Phs4A_34586_92***
754.0
L
4A
0.7
F
603.1
603.8
604.6
Phs4A_3743_9***
Phs4A_34562_92***
Phs4A_34586_92***
754.0
L
4A
0.7
F
34.0
40.0_pnw2_W_xpe_2.43_pic_0.26/0.41_maj_0.85/0.7_css693
83.0
93.0_pnw2_W_fst_0.19_pic_0.48/0.2_maj_0.59/0.89_css701
558.0
565.0_gpl2_W_fst_0.12_pic_0.32/0.42_maj_0.78/0.6_css707
603.1
603.8
604.6
Phs4A_3743_9***
Phs4A_34562_92***
Phs4A_34586_92***
647.0
652.0_SWW2_W_xpe_2.15_pic_0.35/0.43_maj_0.72/0.66_css711
718.0
724.0_SWW2_W_rsb_2.81_pic_0.38/0.37_maj_0.69/0.71_css713
724.0_SWW2_W_xpe_2.64_pic_0.38/0.37_maj_0.69/0.71_css714
724.0_win2_W_xpe_3.30_pic_0.32/0.38_maj_0.79/0.71_css715
754.0
L

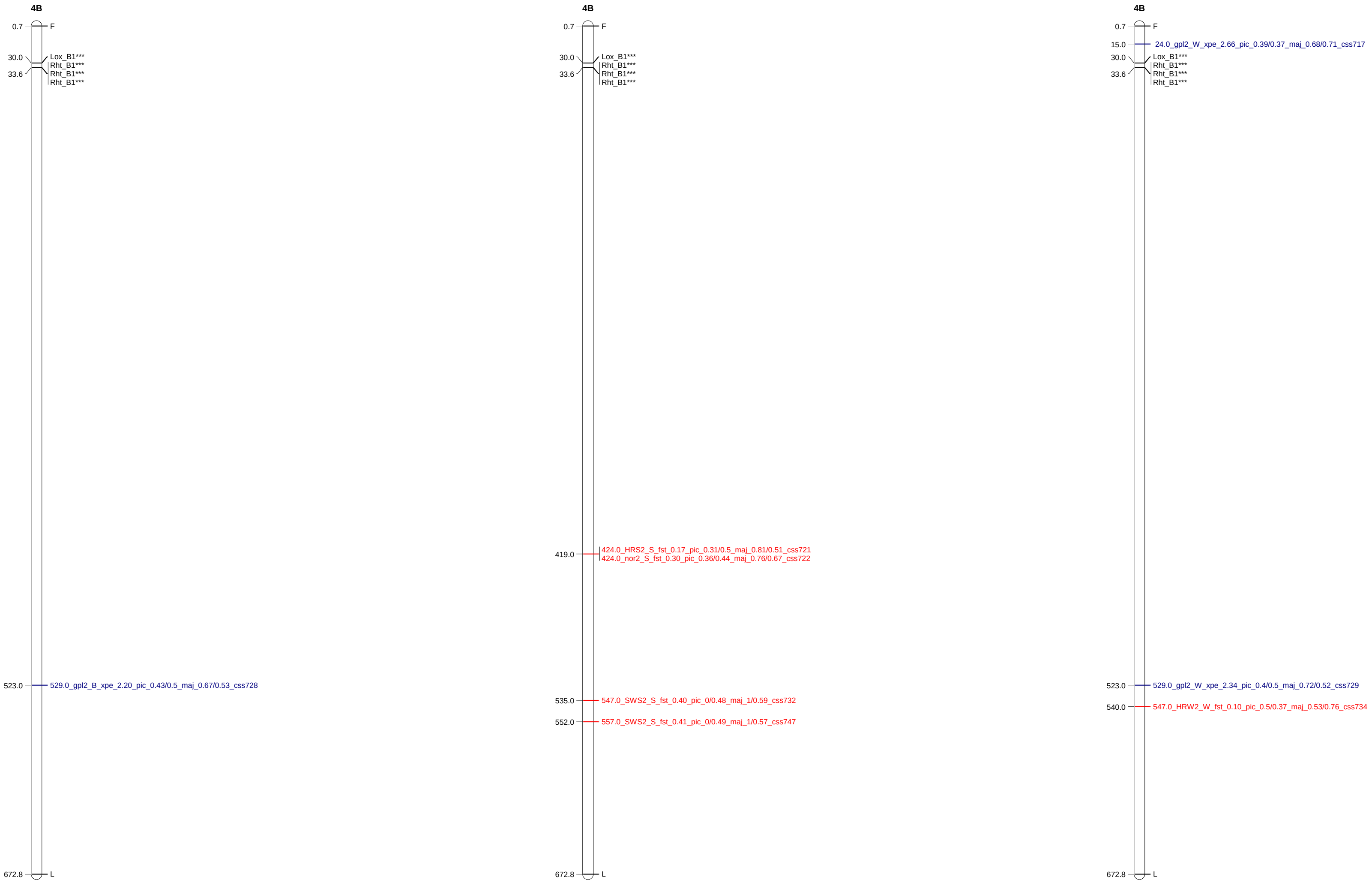
4B
0.7
F
30.0
33.6
Lox_B1***
Rht_B1***
Rht_B1***
Rht_B1***
523.0
529.0_gpl2_B_xpe_2.20_pic_0.43/0.5_maj_0.67/0.53_css728
672.8
L
4B
0.7
F
30.0
33.6
Lox_B1***
Rht_B1***
Rht_B1***
Rht_B1***
419.0
424.0_HRS2_S_fst_0.17_pic_0.31/0.5_maj_0.81/0.51_css721
424.0_nor2_S_fst_0.30_pic_0.36/0.44_maj_0.76/0.67_css722
535.0
547.0_SWS2_S_fst_0.40_pic_0/0.48_maj_1/0.59_css732
552.0
557.0_SWS2_S_fst_0.41_pic_0/0.49_maj_1/0.57_css747
672.8
L
4B
0.7
F
15.0
24.0_gpl2_W_xpe_2.66_pic_0.39/0.37_maj_0.68/0.71_css717
30.0
33.6
Lox_B1***
Rht_B1***
Rht_B1***
Rht_B1***
523.0
529.0_gpl2_W_xpe_2.34_pic_0.4/0.5_maj_0.72/0.52_css729
540.0
547.0_HRW2_W_fst_0.10_pic_0.5/0.37_maj_0.53/0.76_css734
672.8
L

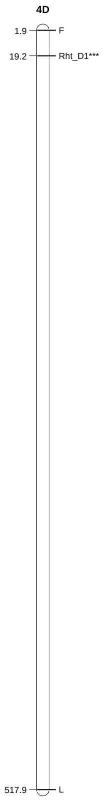
4D
1.9
F
19.2
Rht_D1***
517.9
L

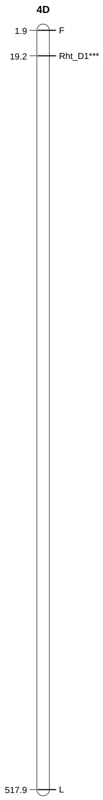
4D
1.9
F
19.2
Rht_D1***
517.9
L

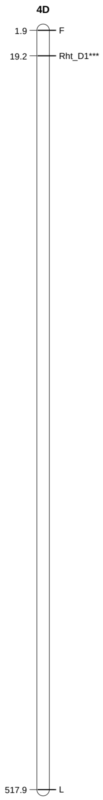
4D
1.9
F
19.2
Rht_D1***
517.9
L

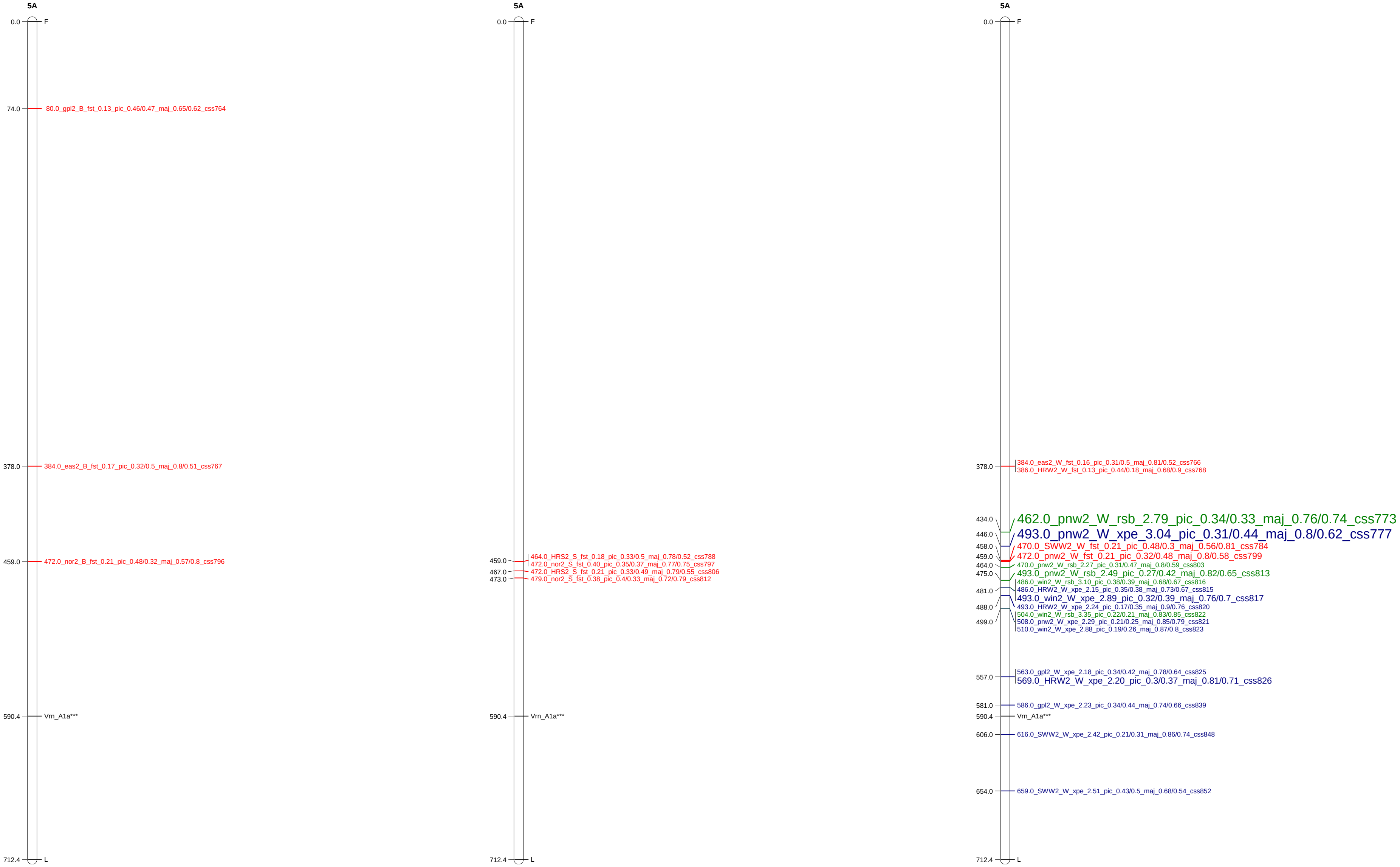

5A
0.0
F
74.0
80.0_gpl2_B_fst_0.13_pic_0.46/0.47_maj_0.65/0.62_css764
378.0
384.0_eas2_B_fst_0.17_pic_0.32/0.5_maj_0.8/0.51_css767
459.0
472.0_nor2_B_fst_0.21_pic_0.48/0.32_maj_0.57/0.8_css796
590.4
Vrn_A1a***
712.4
L
5A
0.0
F
459.0
467.0
473.0
464.0_HRS2_S_fst_0.18_pic_0.33/0.5_maj_0.78/0.52_css788
472.0_nor2_S_fst_0.40_pic_0.35/0.37_maj_0.77/0.75_css797
472.0_HRS2_S_fst_0.21_pic_0.33/0.49_maj_0.79/0.55_css806
479.0_nor2_S_fst_0.38_pic_0.4/0.33_maj_0.72/0.79_css812
590.4
Vrn_A1a***
712.4
L
5A
0.0
F
378.0
384.0_eas2_W_fst_0.16_pic_0.31/0.5_maj_0.81/0.52_css766
386.0_HRW2_W_fst_0.13_pic_0.44/0.18_maj_0.68/0.9_css768
434.0
446.0
458.0
459.0
464.0
475.0
481.0
488.0
499.0
462.0_pnw2_W_rsb_2.79_pic_0.34/0.33_maj_0.76/0.74_css773
493.0_pnw2_W_xpe_3.04_pic_0.31/0.44_maj_0.8/0.62_css777
470.0_SWW2_W_fst_0.21_pic_0.48/0.3_maj_0.56/0.81_css784
472.0_pnw2_W_fst_0.21_pic_0.32/0.48_maj_0.8/0.58_css799
470.0_pnw2_W_rsb_2.27_pic_0.31/0.47_maj_0.8/0.59_css803
493.0_pnw2_W_rsb_2.49_pic_0.27/0.42_maj_0.82/0.65_css813
486.0_win2_W_rsb_3.10_pic_0.38/0.39_maj_0.68/0.67_css816
486.0_HRW2_W_xpe_2.15_pic_0.35/0.38_maj_0.73/0.67_css815
493.0_win2_W_xpe_2.89_pic_0.32/0.39_maj_0.76/0.7_css817
493.0_HRW2_W_xpe_2.24_pic_0.17/0.35_maj_0.9/0.76_css820
504.0_win2_W_rsb_3.35_pic_0.22/0.21_maj_0.83/0.85_css822
508.0_pnw2_W_xpe_2.29_pic_0.21/0.25_maj_0.85/0.79_css821
510.0_win2_W_xpe_2.88_pic_0.19/0.26_maj_0.87/0.8_css823
557.0
563.0_gpl2_W_xpe_2.18_pic_0.34/0.42_maj_0.78/0.64_css825
569.0_HRW2_W_xpe_2.20_pic_0.3/0.37_maj_0.81/0.71_css826
581.0
586.0_gpl2_W_xpe_2.23_pic_0.34/0.44_maj_0.74/0.66_css839
590.4
Vrn_A1a***
606.0
616.0_SWW2_W_xpe_2.42_pic_0.21/0.31_maj_0.86/0.74_css848
654.0
659.0_SWW2_W_xpe_2.51_pic_0.43/0.5_maj_0.68/0.54_css852
712.4
L

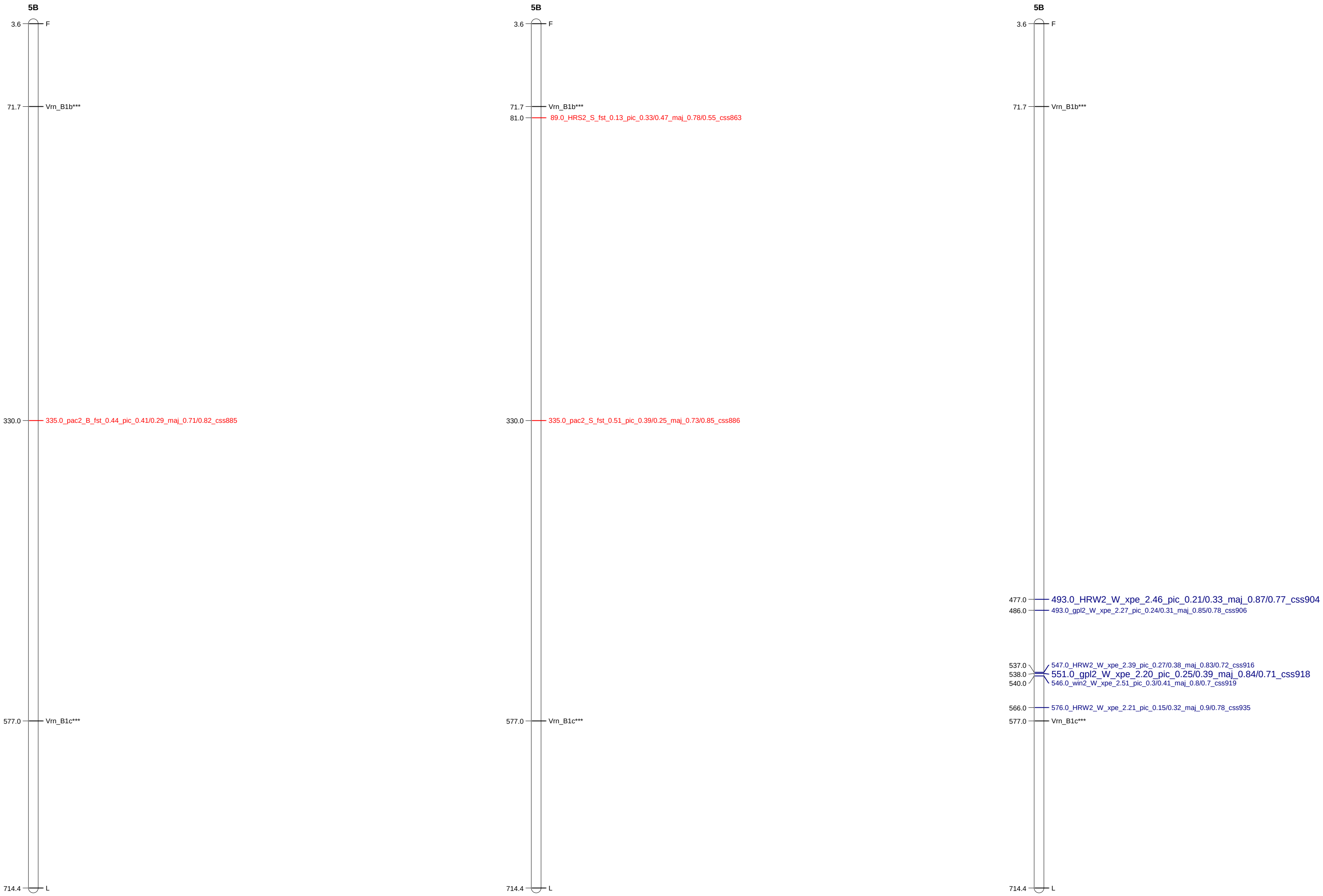
5B
3.6
F
71.7
Vrn_B1b***
330.0
335.0_pac2_B_fst_0.44_pic_0.41/0.29_maj_0.71/0.82_css885
577.0
Vrn_B1c***
714.4
L
5B
3.6
F
71.7
Vrn_B1b***
81.0
89.0_HRS2_S_fst_0.13_pic_0.33/0.47_maj_0.78/0.55_css863
330.0
335.0_pac2_S_fst_0.51_pic_0.39/0.25_maj_0.73/0.85_css886
577.0
Vrn_B1c***
714.4
L
5B
3.6
F
71.7
Vrn_B1b***
477.0
493.0_HRW2_W_xpe_2.46_pic_0.21/0.33_maj_0.87/0.77_css904
486.0
493.0_gpl2_W_xpe_2.27_pic_0.24/0.31_maj_0.85/0.78_css906
537.0
547.0_HRW2_W_xpe_2.39_pic_0.27/0.38_maj_0.83/0.72_css916
538.0
551.0_gpl2_W_xpe_2.20_pic_0.25/0.39_maj_0.84/0.71_css918
540.0
546.0_win2_W_xpe_2.51_pic_0.3/0.41_maj_0.8/0.7_css919
566.0
576.0_HRW2_W_xpe_2.21_pic_0.15/0.32_maj_0.9/0.78_css935
577.0
Vrn_B1c***
714.4
L

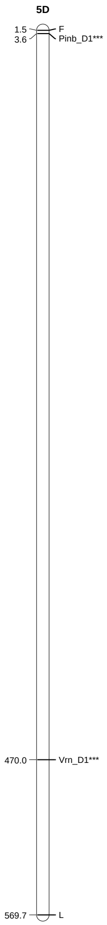
5D
1.5
3.6
F
Pinb_D1***
470.0
Vrn_D1***
569.7
L

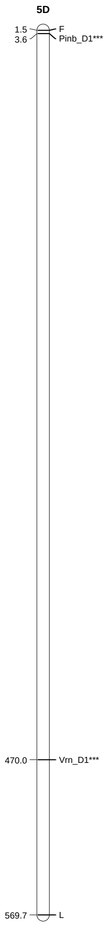
5D
1.5
3.6
F
Pinb_D1***
470.0
Vrn_D1***
569.7
L

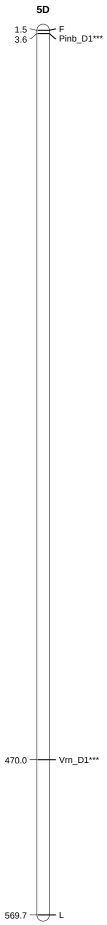
5D
1.5
3.6
F
Pinb_D1***
470.0
Vrn_D1***
569.7
L

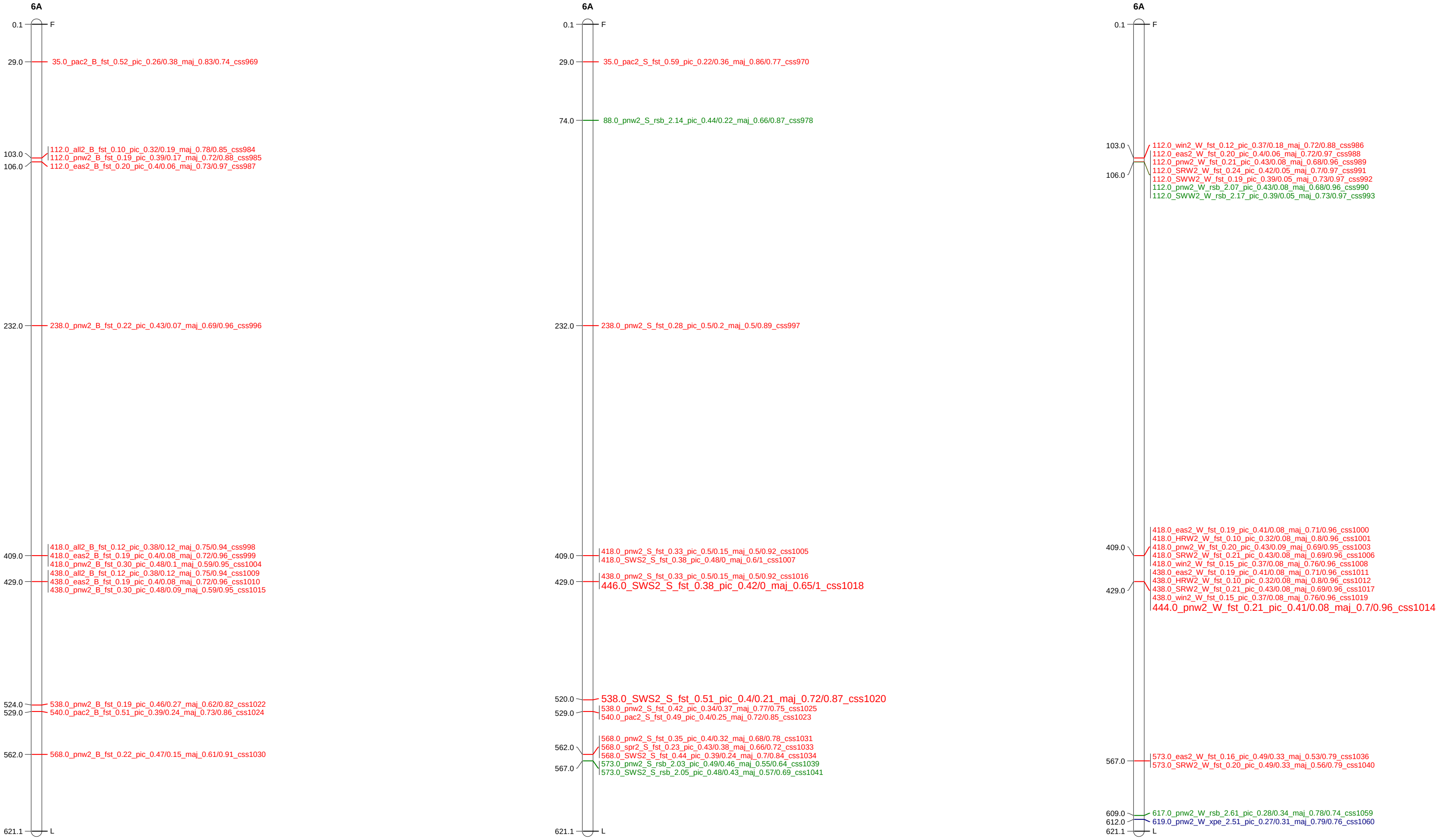
6A
0.1 F
29.0 35.0_pac2_B_fst_0.52_pic_0.26/0.38_maj_0.83/0.74_css969
103.0
106.0
112.0_all2_B_fst_0.10_pic_0.32/0.19_maj_0.78/0.85_css984
112.0_pnw2_B_fst_0.19_pic_0.39/0.17_maj_0.72/0.88_css985
112.0_eas2_B_fst_0.20_pic_0.4/0.06_maj_0.73/0.97_css987
232.0 238.0_pnw2_B_fst_0.22_pic_0.43/0.07_maj_0.69/0.96_css996
409.0
418.0_all2_B_fst_0.12_pic_0.38/0.12_maj_0.75/0.94_css998
418.0_eas2_B_fst_0.19_pic_0.4/0.08_maj_0.72/0.96_css999
418.0_pnw2_B_fst_0.30_pic_0.48/0.1_maj_0.59/0.95_css1004
429.0
438.0_all2_B_fst_0.12_pic_0.38/0.12_maj_0.75/0.94_css1009
438.0_eas2_B_fst_0.19_pic_0.4/0.08_maj_0.72/0.96_css1010
438.0_pnw2_B_fst_0.30_pic_0.48/0.09_maj_0.59/0.95_css1015
524.0 538.0_pnw2_B_fst_0.19_pic_0.46/0.27_maj_0.62/0.82_css1022
529.0 540.0_pac2_B_fst_0.51_pic_0.39/0.24_maj_0.73/0.86_css1024
562.0 568.0_pnw2_B_fst_0.22_pic_0.47/0.15_maj_0.61/0.91_css1030
621.1 L
6A
0.1 F
29.0 35.0_pac2_S_fst_0.59_pic_0.22/0.36_maj_0.86/0.77_css970
74.0 88.0_pnw2_S_rsb_2.14_pic_0.44/0.22_maj_0.66/0.87_css978
232.0 238.0_pnw2_S_fst_0.28_pic_0.5/0.2_maj_0.5/0.89_css997
409.0
418.0_pnw2_S_fst_0.33_pic_0.5/0.15_maj_0.5/0.92_css1005
418.0_SWS2_S_fst_0.38_pic_0.48/0_maj_0.6/1_css1007
429.0
438.0_pnw2_S_fst_0.33_pic_0.5/0.15_maj_0.5/0.92_css1016
446.0_SWS2_S_fst_0.38_pic_0.42/0_maj_0.65/1_css1018
520.0 538.0_SWS2_S_fst_0.51_pic_0.4/0.21_maj_0.72/0.87_css1020
529.0
538.0_pnw2_S_fst_0.42_pic_0.34/0.37_maj_0.77/0.75_css1025
540.0_pac2_S_fst_0.49_pic_0.4/0.25_maj_0.72/0.85_css1023
562.0
567.0
568.0_pnw2_S_fst_0.35_pic_0.4/0.32_maj_0.68/0.78_css1031
568.0_spr2_S_fst_0.23_pic_0.43/0.38_maj_0.66/0.72_css1033
568.0_SWS2_S_fst_0.44_pic_0.39/0.24_maj_0.7/0.84_css1034
573.0_pnw2_S_rsb_2.03_pic_0.49/0.46_maj_0.55/0.64_css1039
573.0_SWS2_S_rsb_2.05_pic_0.48/0.43_maj_0.57/0.69_css1041
621.1 L
6A
0.1 F
103.0
106.0
112.0_win2_W_fst_0.12_pic_0.37/0.18_maj_0.72/0.88_css986
112.0_eas2_W_fst_0.20_pic_0.4/0.06_maj_0.72/0.97_css988
112.0_pnw2_W_fst_0.21_pic_0.43/0.08_maj_0.68/0.96_css989
112.0_SRW2_W_fst_0.24_pic_0.42/0.05_maj_0.7/0.97_css991
112.0_SWW2_W_fst_0.19_pic_0.39/0.05_maj_0.73/0.97_css992
112.0_pnw2_W_rsb_2.07_pic_0.43/0.08_maj_0.68/0.96_css990
112.0_SWW2_W_rsb_2.17_pic_0.39/0.05_maj_0.73/0.97_css993
409.0
429.0
418.0_eas2_W_fst_0.19_pic_0.41/0.08_maj_0.71/0.96_css1000
418.0_HRW2_W_fst_0.10_pic_0.32/0.08_maj_0.8/0.96_css1001
418.0_pnw2_W_fst_0.20_pic_0.43/0.09_maj_0.69/0.95_css1003
418.0_SRW2_W_fst_0.21_pic_0.43/0.08_maj_0.69/0.96_css1006
418.0_win2_W_fst_0.15_pic_0.37/0.08_maj_0.76/0.96_css1008
438.0_eas2_W_fst_0.19_pic_0.41/0.08_maj_0.71/0.96_css1011
438.0_HRW2_W_fst_0.10_pic_0.32/0.08_maj_0.8/0.96_css1012
438.0_SRW2_W_fst_0.21_pic_0.43/0.08_maj_0.69/0.96_css1017
438.0_win2_W_fst_0.15_pic_0.37/0.08_maj_0.76/0.96_css1019
444.0_pnw2_W_fst_0.21_pic_0.41/0.08_maj_0.7/0.96_css1014
567.0
573.0_eas2_W_fst_0.16_pic_0.49/0.33_maj_0.53/0.79_css1036
573.0_SRW2_W_fst_0.20_pic_0.49/0.33_maj_0.56/0.79_css1040
609.0 617.0_pnw2_W_rsb_2.61_pic_0.28/0.34_maj_0.78/0.74_css1059
612.0 619.0_pnw2_W_xpe_2.51_pic_0.27/0.31_maj_0.79/0.76_css1060
621.1 L

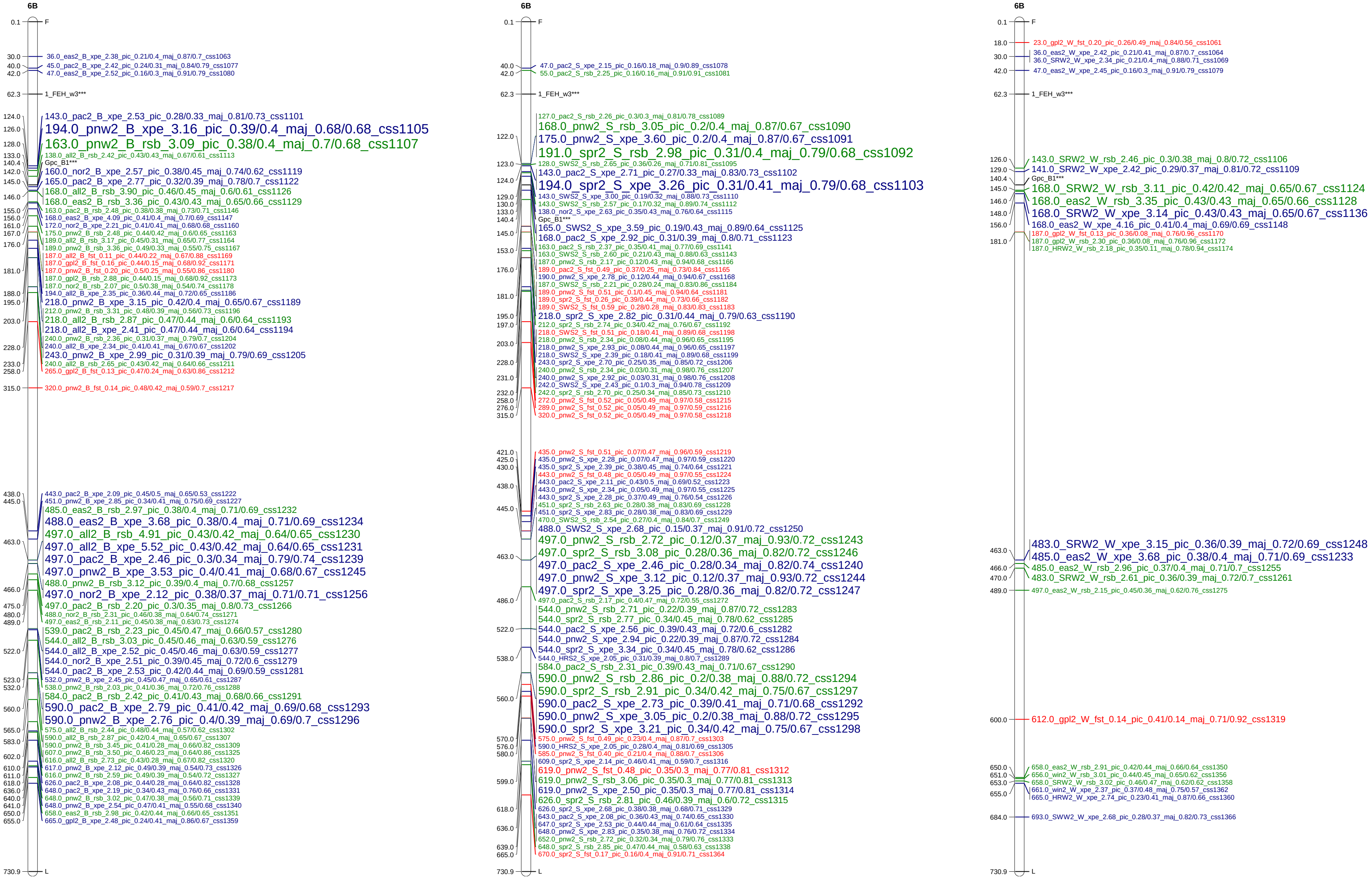
6B
1_FEH_w3***
Gpc_B1***
194.0_pnw2_B_xpe_3.16_pic_0.39/0.4_maj_0.68/0.68_css1105
163.0_pnw2_B_rsb_3.09_pic_0.38/0.4_maj_0.7/0.68_css1107
488.0_eas2_B_xpe_3.68_pic_0.38/0.4_maj_0.71/0.69_css1234
497.0_all2_B_rsb_4.91_pic_0.43/0.42_maj_0.64/0.65_css1230
497.0_all2_B_xpe_5.52_pic_0.43/0.42_maj_0.64/0.65_css1231
497.0_pac2_B_xpe_2.46_pic_0.3/0.34_maj_0.79/0.74_css1239
497.0_pnw2_B_xpe_3.53_pic_0.4/0.41_maj_0.68/0.67_css1245
497.0_nor2_B_xpe_2.12_pic_0.38/0.37_maj_0.71/0.71_css1256
590.0_pac2_B_xpe_2.79_pic_0.41/0.42_maj_0.69/0.68_css1293
590.0_pnw2_B_xpe_2.76_pic_0.4/0.39_maj_0.69/0.7_css1296
6B
1_FEH_w3***
168.0_pnw2_S_rsb_3.05_pic_0.2/0.4_maj_0.87/0.67_css1090
175.0_pnw2_S_xpe_3.60_pic_0.2/0.4_maj_0.87/0.67_css1091
191.0_spr2_S_rsb_2.98_pic_0.31/0.4_maj_0.79/0.68_css1092
194.0_spr2_S_xpe_3.26_pic_0.31/0.41_maj_0.79/0.68_css1103
497.0_pnw2_S_rsb_2.72_pic_0.12/0.37_maj_0.93/0.72_css1243
497.0_spr2_S_rsb_3.08_pic_0.28/0.36_maj_0.82/0.72_css1246
497.0_pac2_S_xpe_2.46_pic_0.28/0.34_maj_0.82/0.74_css1240
497.0_pnw2_S_xpe_3.12_pic_0.12/0.37_maj_0.93/0.72_css1244
497.0_spr2_S_xpe_3.25_pic_0.28/0.36_maj_0.82/0.72_css1247
590.0_pnw2_S_rsb_2.86_pic_0.2/0.38_maj_0.88/0.72_css1294
590.0_spr2_S_rsb_2.91_pic_0.34/0.42_maj_0.75/0.67_css1297
590.0_pac2_S_xpe_2.73_pic_0.39/0.41_maj_0.71/0.68_css1292
590.0_pnw2_S_xpe_3.05_pic_0.2/0.38_maj_0.88/0.72_css1295
590.0_spr2_S_xpe_3.21_pic_0.34/0.42_maj_0.75/0.67_css1298
6B
1_FEH_w3***
Gpc_B1***
168.0_SRW2_W_rsb_3.11_pic_0.42/0.42_maj_0.65/0.67_css1124
168.0_eas2_W_rsb_3.35_pic_0.43/0.43_maj_0.65/0.66_css1128
168.0_SRW2_W_xpe_3.14_pic_0.43/0.43_maj_0.65/0.67_css1136
483.0_SRW2_W_xpe_3.15_pic_0.36/0.39_maj_0.72/0.69_css1248
485.0_eas2_W_xpe_3.68_pic_0.38/0.4_maj_0.71/0.69_css1233
612.0_gpl2_W_fst_0.14_pic_0.41/0.14_maj_0.71/0.92_css1319

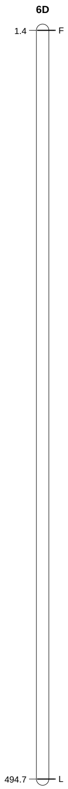
6D
1.4
F
494.7
L

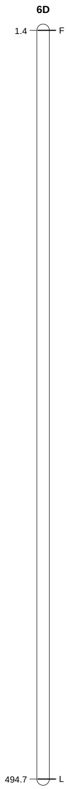
6D
1.4
F
494.7
L

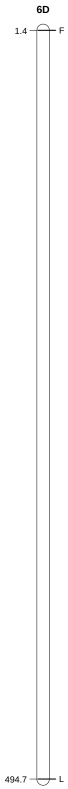
6D
1.4
F
494.7
L

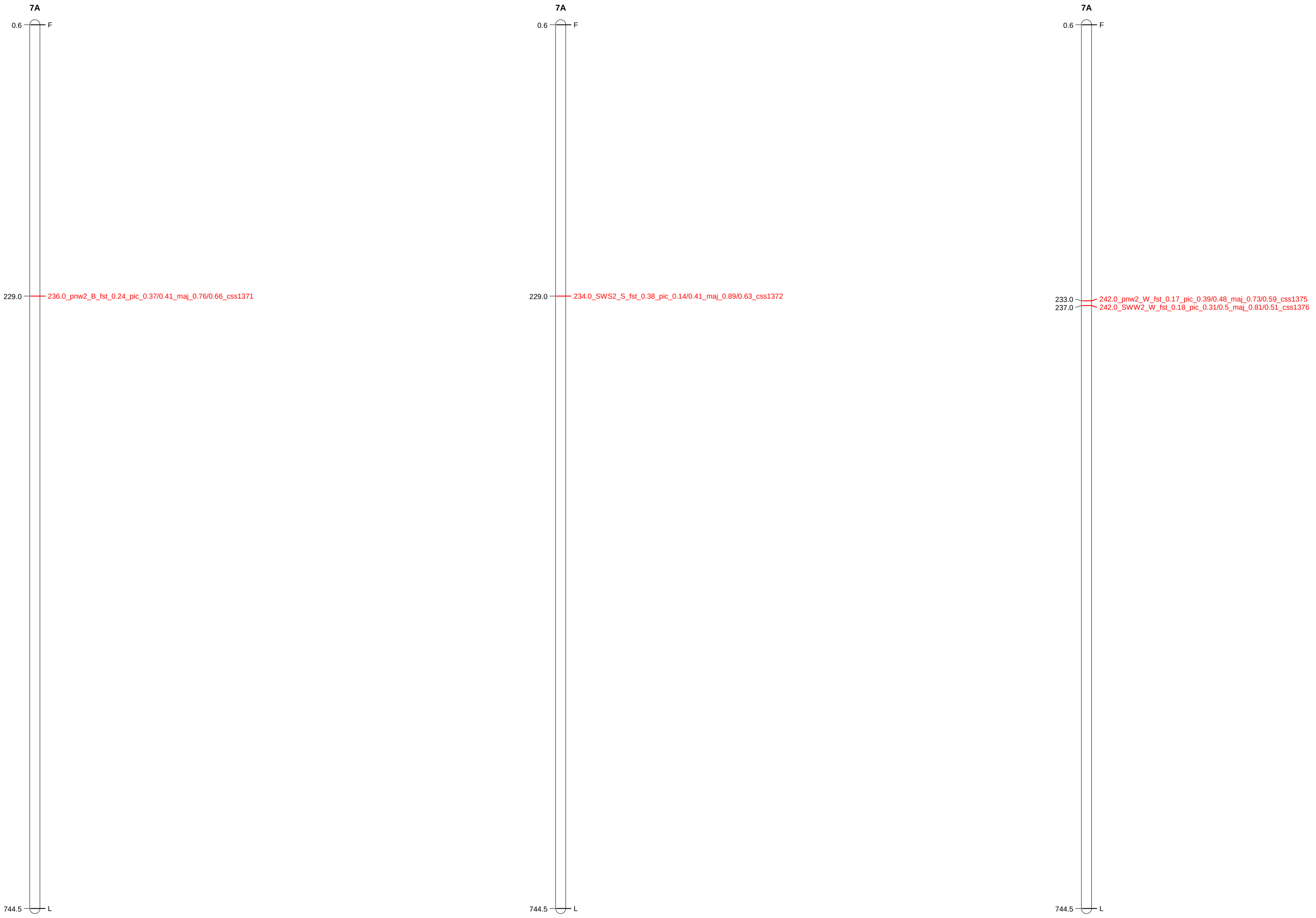
7A
0.6
F
229.0
236.0_pnw2_B_fst_0.24_pic_0.37/0.41_maj_0.76/0.66_css1371
744.5
L
7A
0.6
F
229.0
234.0_SWS2_S_fst_0.38_pic_0.14/0.41_maj_0.89/0.63_css1372
744.5
L
7A
0.6
F
233.0
242.0_pnw2_W_fst_0.17_pic_0.39/0.48_maj_0.73/0.59_css1375
237.0
242.0_SWW2_W_fst_0.18_pic_0.31/0.5_maj_0.81/0.51_css1376
744.5
L

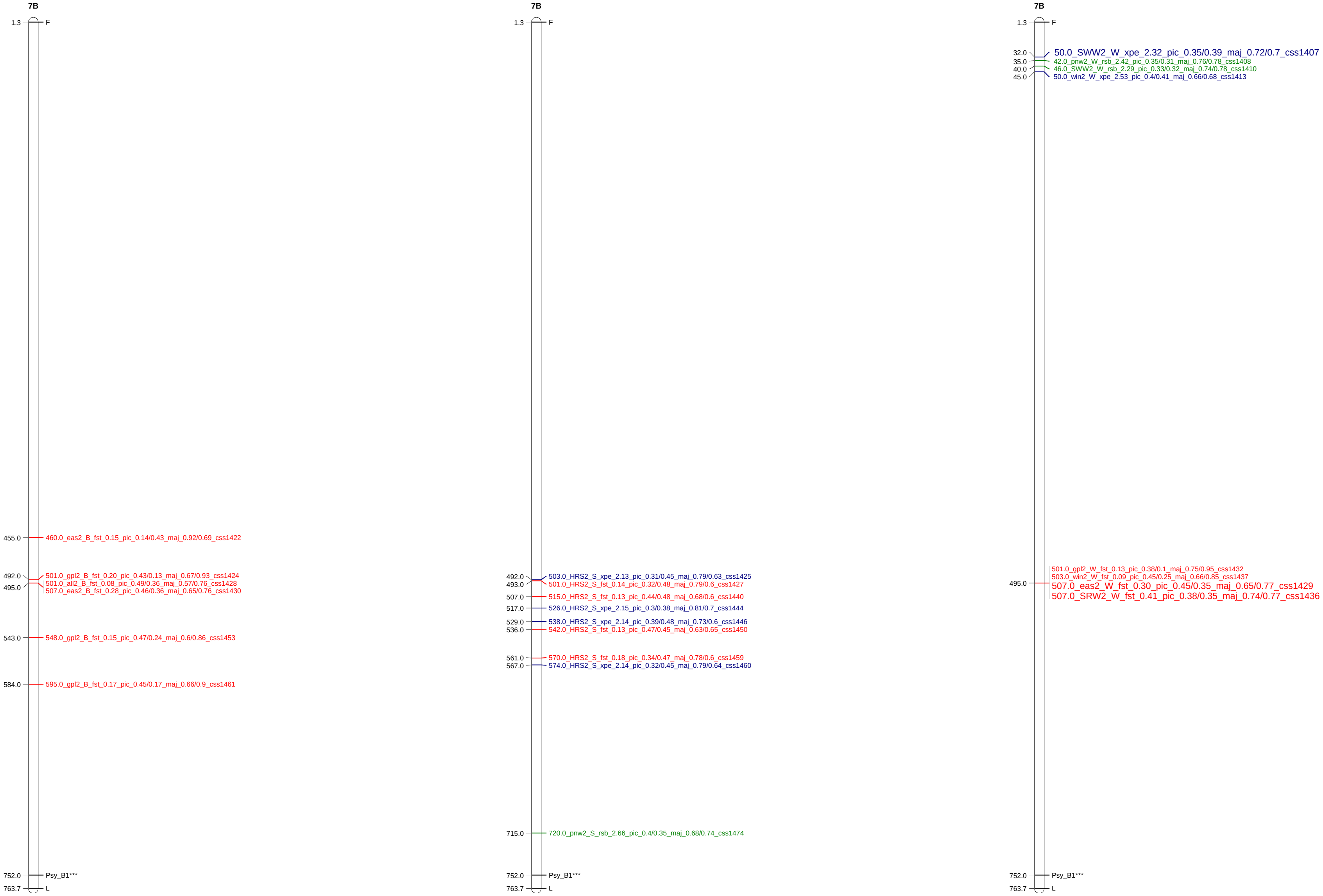
7B
1.3 F
455.0 460.0_eas2_B_fst_0.15_pic_0.14/0.43_maj_0.92/0.69_css1422
492.0 501.0_gpl2_B_fst_0.20_pic_0.43/0.13_maj_0.67/0.93_css1424
501.0_all2_B_fst_0.08_pic_0.49/0.36_maj_0.57/0.76_css1428
495.0 507.0_eas2_B_fst_0.28_pic_0.46/0.36_maj_0.65/0.76_css1430
543.0 548.0_gpl2_B_fst_0.15_pic_0.47/0.24_maj_0.6/0.86_css1453
584.0 595.0_gpl2_B_fst_0.17_pic_0.45/0.17_maj_0.66/0.9_css1461
752.0 Psy_B1***
763.7 L
7B
1.3 F
492.0 503.0_HRS2_S_xpe_2.13_pic_0.31/0.45_maj_0.79/0.63_css1425
493.0 501.0_HRS2_S_fst_0.14_pic_0.32/0.48_maj_0.79/0.6_css1427
507.0 515.0_HRS2_S_fst_0.13_pic_0.44/0.48_maj_0.68/0.6_css1440
517.0 526.0_HRS2_S_xpe_2.15_pic_0.3/0.38_maj_0.81/0.7_css1444
529.0 538.0_HRS2_S_xpe_2.14_pic_0.39/0.48_maj_0.73/0.6_css1446
536.0 542.0_HRS2_S_fst_0.13_pic_0.47/0.45_maj_0.63/0.65_css1450
561.0 570.0_HRS2_S_fst_0.18_pic_0.34/0.47_maj_0.78/0.6_css1459
567.0 574.0_HRS2_S_xpe_2.14_pic_0.32/0.45_maj_0.79/0.64_css1460
715.0 720.0_pnw2_S_rsb_2.66_pic_0.4/0.35_maj_0.68/0.74_css1474
752.0 Psy_B1***
763.7 L
7B
1.3 F
32.0 50.0_SWW2_W_xpe_2.32_pic_0.35/0.39_maj_0.72/0.7_css1407
35.0 42.0_pnw2_W_rsb_2.42_pic_0.35/0.31_maj_0.76/0.78_css1408
40.0 46.0_SWW2_W_rsb_2.29_pic_0.33/0.32_maj_0.74/0.78_css1410
45.0 50.0_win2_W_xpe_2.53_pic_0.4/0.41_maj_0.66/0.68_css1413
501.0_gpl2_W_fst_0.13_pic_0.38/0.1_maj_0.75/0.95_css1432
503.0_win2_W_fst_0.09_pic_0.45/0.25_maj_0.66/0.85_css1437
495.0 507.0_eas2_W_fst_0.30_pic_0.45/0.35_maj_0.65/0.77_css1429
507.0_SRW2_W_fst_0.41_pic_0.38/0.35_maj_0.74/0.77_css1436
752.0 Psy_B1***
763.7 L

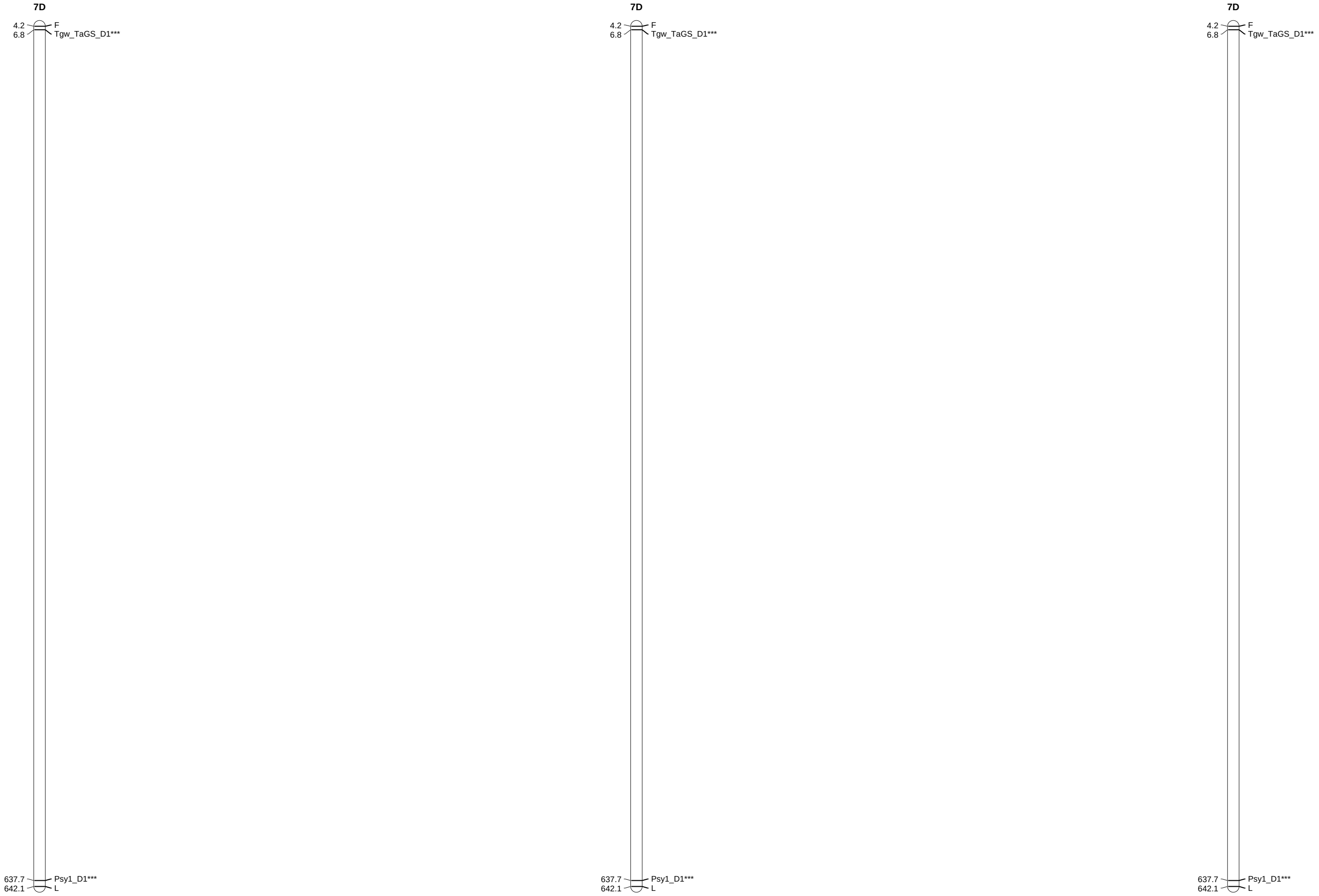

Supplemental Figure S3. Map of candidate selective sweeps (CSS) in U.S. wheat populations due to selection over time. Each population pair was obtained by splitting a population (e.g., HRS with 150 varieties) into two halves, one of older varieties (e.g., HRS1 with 75 oldest varieties) and the other of newer varieties (e.g., HRS2 with 75 newest varieties). F_{st}, Rsb, and xpEHH were computed for these pairs. The linkage blocks from left to right show CSS in population pairs with a) both spring and winter varieties, b) just spring varieties, and c) just winter varieties. Physical positions in Mbp for the start of the sweep are on the left side of the bar. The right side includes end position of the CSS, name of the population selected in (spr, spring; win, winter; eas, Eastern; gpl, the Great Plains; nor, Northern, pac, the Pacific; pnw, the Pacific Northwest; HRS, hard red spring; HRW, hard red winter; SRW, soft red winter; SWS, soft white spring; SWW, soft white winter), growth habit (B, S, and W for both, spring, and winter), statistic and its maximum value, PIC values in target and reference population, major allele frequencies in the target and reference population, and CSS serial number. Red, green, and blue color of the label indicate CSS detected using F_{st}, Rsb, and xpEHH respectively. Size of the label corresponds with the size of the CSS. Location of known genes are indicated by (***) and F and L refer to the physical positions of the first and last SNP genotyped on the chromosome.
