# Supplementary material for: Candidate selective sweeps in US wheat populations
Source: Plant Genome. 2024 Sep 25;17(4):e20513. doi: 10.1002/tpg2.20513 (PMC11628914; doi:10.1002/tpg2.20513)
Supplement: Supplementary file 5 — Supplemental Figure S5. Distribution of 24,033 loci used in the analysis across the wheat chromosomes. Each bin size is 2.5Mbp and the dashed line represents the best estimated location of centromere (IWGSC et al., 2018). [file TPG2-17-e20513-s003.pdf]

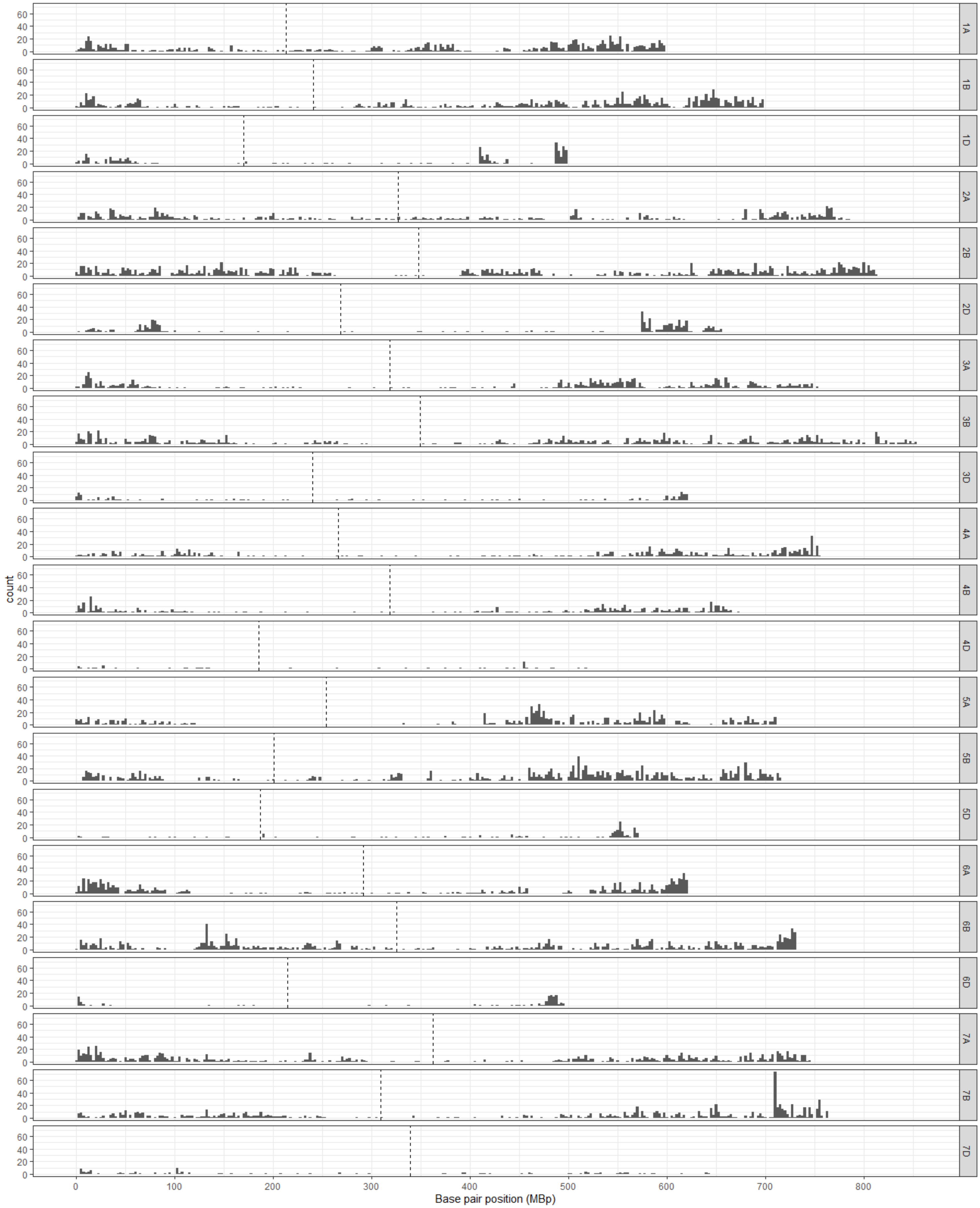

Supplemental Figure S5. Distribution of 24,033 loci used in the analysis across the wheat chromosomes. Each bin size is 2.5Mbp and the dashed line represents the best estimated location of centromere (IWGSC et al., 2018).
